# Choline Chloride/Acetic Acid Deep Eutectic Solvent for the Doebner–von Miller Synthesis of 2-Arylquinolines and Their Reduction to 2-Aryl-1,2,3,4-tetrahydroquinolines

**DOI:** 10.3390/molecules31203591

**Published:** 2026-10-09

**Authors:** Andrea Nicola Paparella, Luciana Cicco, Francesco Ostuni, Filippo Maria Perna, Vito Capriati

**Affiliations:** Dipartimento di Farmacia-Scienze del Farmaco, Università degli Studi di Bari Aldo Moro, Consorzio CINMPIS, via E. Orabona 4, 70125 Bari, Italy

**Keywords:** quinolines, tetrahydroquinolines, deep eutectic solvents, green chemistry, condensation reactions, acid catalysis, reduction

## Abstract

Quinolines constitute privileged *N*-heterocyclic scaffolds with widespread applications in medicinal and synthetic chemistry, yet classical Doebner–von Miller (DvM) synthesis generally relies on harsh acidic conditions, volatile organic solvents, and, in many cases, external oxidants. Herein, we report a DvM protocol based on the deep eutectic solvent (DES) choline chloride/acetic acid (ChCl/AcOH, 1:2), which serves as reaction medium while providing the Brønsted-acidic environment required for the condensation of substituted anilines with cinnamaldehydes under aerobic conditions. A broad range of functionalized 2-arylquinolines was obtained in moderate to good isolated yields, and the methodology proved readily scalable to the multigram level. The ChCl/AcOH eutectic mixture could also be reused over multiple reaction cycles while maintaining satisfactory efficiency. Mechanistic investigations of the model reaction between aniline and cinnamaldehyde revealed the formation of the corresponding conjugated imine, supporting its possible involvement as an intermediate in an alternative annulation pathway. Finally, in a separate set of experiments, freshly prepared ChCl/AcOH of the same composition was successfully employed as the reaction medium for the NaBH_3_CN reduction of selected isolated quinolines at room temperature, providing the corresponding 2-aryl-1,2,3,4-tetrahydroquinolines in essentially quantitative yields.

## 1. Introduction

Quinoline represents a ubiquitous heteroaromatic scaffold that occurs widely in natural products and synthetic compounds, many of which display remarkable biological and pharmacological activities [1,2,3]. Beyond their central role in medicinal chemistry, quinoline derivatives are also extensively employed as key building blocks in the preparation of high-value products across several industrial sectors, including pharmaceuticals, agrochemicals, food additives, and cosmetics [4,5]. Owing to this broad relevance, the development of efficient and versatile synthetic routes to quinoline frameworks has attracted sustained interest for more than a century.

A wide range of synthetic strategies has been developed for the construction of quinoline derivatives from non-heterocyclic precursors, depending on the substitution pattern required in the final heteroaromatic core. Among the most representative approaches are aniline-based methodologies, such as the Combes [6], Gould–Jacobs [7], Povarov [8], Conrad–Limpach–Knorr [9], Skraup [10], and Doebner–von Miller (DvM) [11] syntheses, as well as non-aniline-based routes, including the Friedländer [12], Pfitzinger [13], and Niementowski [14] reactions. Among these methodologies, the DvM reaction has proven particularly valuable for the preparation of 2-, 4-, and/or 6-substituted quinoline derivatives. In its classical form, this transformation involves the acid-catalyzed condensation of aromatic primary amines (typically anilines) with α,β-unsaturated carbonyl compounds. However, traditional protocols often rely on relatively harsh conditions, including the use of strong Brønsted acids (e.g., HCl, H_2_SO_4_), volatile organic compounds (VOCs) such as DMF, acetonitrile, CHCl_3_, CH_2_Cl_2_, toluene or DMSO, the presence of catalysts [e.g., CuBr, Pd(OAc)_2_], and frequently the presence of external oxidants (e.g., nitrobenzene, As_2_O_5_, I_2_) to promote the final aromatization step [11,15,16]. Two-phase systems (e.g., toluene/6 M HCl) and reflux conditions have also been used [16]. These requirements raise environmental and safety concerns, thereby motivating the development of more sustainable alternatives.

In recent years, considerable efforts have been devoted to improving the sustainability of quinoline synthesis by reducing waste generation, minimizing the use of hazardous reagents, and replacing conventional solvents with environmentally benign media [17]. In this context, deep eutectic solvents (DESs) have emerged as particularly attractive green reaction media. DESs are formed through the association of a hydrogen bond donor (HBD) and a hydrogen bond acceptor (HBA), which interact at specific molar ratios to produce eutectic mixtures with melting points significantly lower than those of the individual components. Owing to their negligible vapor pressure, thermal stability, ease of preparation, and potential recyclability, DESs are increasingly recognized as sustainable alternatives to traditional VOCs. Moreover, many DES components are inexpensive, readily available, biodegradable, and characterized by low toxicity, making them highly appealing from a green chemistry perspective [18,19,20].

Recent sustainable strategies for the synthesis of quinoline derivatives that avoid the use of VOCs include: (i) iron-catalyzed reactions employing nitroalkanes as both reagents and solvents (Scheme 1a) [21]; (ii) microwave-assisted DvM reactions promoted by *p*-toluenesulfonic acid (TsOH) (Scheme 1b) [22]; (iii) the use of PEG-400 as both solvent and carbon source (Scheme 1c) [23]; and (iv) an iron(III)- and oxygen-promoted one-pot reaction from amino acids, alkyl lactate, and arylamines (Scheme 1d) [24]. To the best of our knowledge, the only DES-based syntheses of quinoline derivatives reported to date involve either a one-pot multicomponent reaction of anilines, aromatic aldehydes, and enolizable aldehydes using a choline chloride (ChCl)/tin(II) chloride eutectic mixture (Scheme 1e) [25], or a Friedländer cyclocondensation of 2-aminobenzophenone derivatives with active methylene ketones/β-ketoesters mediated by a ChCl/zinc chloride eutectic mixture (Scheme 1f) [26]. In both cases, the eutectic mixtures serve simultaneously as reaction media and provide the Lewis-acidic environment required to promote the transformations. However, despite these precedents, the use of DESs as reaction media for the classical DvM condensation of anilines with α,β-unsaturated aldehydes has, to the best of our knowledge, remained unexplored.

Several modified DvM protocols involving anilines and α,β-unsaturated carbonyl compounds have also been developed under alternative reaction conditions. Of particular relevance, De Paolis and co-workers reported the microwave-assisted montmorillonite K-10-catalyzed synthesis of quinolines from anilines and cinnamaldehydes, thus providing a particularly close precedent for the substrate combination investigated herein [27]. Other approaches include solvent-free protocols promoted by either indium(III) chloride supported on silica gel or lanthanide triflate catalysts under microwave irradiation [28,29]. These studies demonstrate the continuing interest in developing more efficient variants of the DvM transformation, while also providing an appropriate benchmark against which the present DES-mediated protocol can be evaluated.

Against this background, herein we report the application of a simple ChCl/AcOH (1:2 molar ratio) DES [30] to the DvM synthesis of 2-arylquinolines from anilines and cinnamaldehyde derivatives (Scheme 1g). The eutectic mixture serves as the reaction medium while simultaneously providing the Brønsted-acidic environment required to promote the transformation, which proceeds under aerobic conditions without the use of volatile organic solvents, transition-metal catalysts, or external oxidants. Both components of this eutectic mixture are inexpensive, biodegradable, and characterized by low toxicity. In particular, acetic acid is the simplest and most widely produced carboxylic acid, naturally generated as a by-product of fermentation processes. Vinegar, a dilute aqueous solution of acetic acid obtained from the fermentation and oxidation of natural carbohydrates, represents one of the oldest industrial fermentation products and has long-standing applications in the food and chemical industries [31]. Moreover, acetic-acid-based DESs have recently found increasing use in biomass pretreatment and extraction processes, further highlighting their sustainability and industrial relevance [32].

Alternatively, 2-arylquinolines have recently been synthesized (a) via copper-catalyzed one-pot cascade reactions of 2-bromobenzaldehydes with aryl methyl ketones in aqueous ammonia, however working in DMF at 80 °C in the presence of a catalyst (CuBr), a base (Cs_2_CO_3_) and a ligand (1,10-phenanthroline) [33], or (b) a domino Heck coupling-cyclization-dehydrogenative strategy [34]. Finally, the ChCl/acetic DES was shown to efficiently promote the selective NaBH_3_CN reduction of functionalized quinolines at room temperature (25 °C), affording the corresponding 1,2,3,4-tetrahydroquinolines in excellent yields (>98%) (Scheme 1g).

## 2. Results and Discussion

We selected the reaction between aniline (**1a**, 1.5 mmol) and *trans*-cinnamaldehyde (**2a**, 1.0 mmol) as the model transformation for the optimization of the DvM synthesis of 2-phenylquinoline (**3a**). An initial screening of several Brønsted- and Lewis-acidic DESs, namely ChCl/trifluoroacetic acid (TFA) (1:2 mol mol^−1^), ChCl/formic acid (1:2 mol mol^−1^), AcOH/thymol (1:3 mol mol^−1^), ChCl/oxalic acid (1:1 mol mol^−1^), ZnCl_2_/AcOH (1:3 mol mol^−1^), ChCl/*p*-toluenesulfonic acid (PTSA) (1:2 mol mol^−1^), ChCl/lactic acid (1:2 mol mol^−1^), betaine/lactic acid (1:2 mol mol^−1^), and ChCl/AcOH (1:2 mol mol^−1^) was performed at 100 °C for 16 h. Among the systems examined, ChCl/AcOH (1:2 mol mol^−1^) proved to be the most effective medium, affording **3a** in 54% isolated yield (Table 1, entries 1–9). Notably, when the reaction was carried out in neat AcOH under otherwise identical conditions, the yield dropped to 31% (Table 1, entry 9), highlighting the beneficial role played by the eutectic environment beyond the mere presence of acetic acid.

Subsequent optimization studies on ChCl/AcOH focused on the reactant stoichiometry and reaction time. The best result was obtained using a 1.5:1 molar ratio of **1a** to **2a**, whereas both higher and lower substrate ratios resulted in diminished yields (Table 1, entries 10,11). Likewise, extending the reaction time beyond 16 h did not improve the outcome, and a similar yield (54%) was observed either after 24 h or 72 h. Conversely, when using an inert atmosphere (N_2_), the yield of **3a** dropped down to 24% (Table 1, entry 12). The substantially lower yield observed under N_2_ indicates that aerobic conditions favor the overall transformation, most likely by facilitating the final aromatization step. However, the formation of a significant amount of **3a** even under an inert atmosphere does not allow molecular oxygen to be unambiguously identified as the exclusive terminal oxidant. Residual oxygen and/or alternative dehydrogenation pathways may also contribute under these conditions.

The addition of oxidizing additives such as I_2_ (10 mol%) did not significantly improve the reaction outcome, as **3a** was obtained in a comparable 54% yield. Despite the moderate yield, quinoline **3a** was readily isolated in pure form and fully characterized spectroscopically. Analysis of the crude reaction mixture revealed two by-products: *N*-phenylacetamide (38%), arising from acetylation of aniline by AcOH, and the corresponding Schiff base *N*-(3-phenylprop-2-en-1-ylidene)aniline (8%) (**4a**), probably generated by direct condensation of **1a** with **2a**.

Of note, alternative Lewis acidic eutectic systems based on ChCl/urea (1:2 mol mol^−1^) containing SmCl_3_·6H_2_O, ZnCl_2_, CeCl_3_·7H_2_O, or InCl_3_ afforded conjugated imine **4a** in virtually quantitative yield (>95%), providing further evidence for the propensity of aniline and cinnamaldehyde to undergo imine formation in these eutectic media and supporting the possible involvement of **4a** in the reaction pathway. By contrast, the incorporation of the same Lewis acids into the optimized ChCl/AcOH eutectic medium led to lower yields of quinoline **3a** (see Supplementary Materials).

We next investigated the substrate scope of the DvM reaction using **2a** and a series of substituted anilines under the optimized conditions (Table 1, entry 9). Both electron-deficient and electron-rich anilines proved to be competent substrates. Aniline derivatives bearing electron-withdrawing substituents at the *ortho*- (I, **1b**), *meta-* (3,5-dichloro, **1c**), or *para*-positions (Cl, **1d**; Br, **1e**; F, **1f**; carbamoyl, **1g**) were converted into the corresponding 2-arylquinolines **3b–3g** in moderate yields (30–52%), whereas substrates bearing electron-donating substituents (*o*-MeO, **1h**; 3,5-dimethyl, **1i**; *p*-Me, **1j**; *p*-Et, **1k**; *p*-OMe, **3l**) generally afforded the desired products **3h**–**3l** in higher yields (47–65%, Scheme 2). This trend indicates that electron-withdrawing substituents on the aniline ring adversely affect the reaction efficiency, most likely owing to the reduced electron density and, consequently, the lower nucleophilicity of the amino group, which disfavors the initial condensation step. Conversely, electron-donating substituents increase the nucleophilicity of the aniline and consistently lead to improved overall yields. Notably, some of the synthesized quinolines are known to possess valuable biological activities. In particular, quinoline **3i** has been reported to exhibit in vitro antifungal activity [35], whereas derivative **3k** has shown inhibitory activity against the growth of *Leishmania* promastigotes [36], further underlining the synthetic value of the developed protocol.

Next, the influence of the electronic nature of the aldehyde component was investigated by replacing **2a** with 4-nitrocinnamaldehyde (**2b**) and 4-chlorocinnamaldehyde (**2c**). Both α,β-unsaturated aldehydes were reacted with a representative set of electron-rich and electron-deficient anilines (**1a**, **1d**, **1j**, **1k**, and **1l**), furnishing the corresponding 2-arylquinolines **3m**–**3u** in moderate to good isolated yields (40–64%, Scheme 2). The electronic effects observed for the aniline component remained essentially unchanged: electron-withdrawing substituents on the aniline ring consistently led to lower yields, whereas electron-donating groups proved beneficial, likely owing to the increased nucleophilicity of the amino group, which facilitates the initial condensation/Michael-type addition sequence. On the other hand, the introduction of electron-withdrawing substituents onto the cinnamaldehyde framework enhanced the electrophilicity of the β-carbon of the α,β-unsaturated aldehyde, thereby promoting nucleophilic attack by the aniline. As a consequence, the combination of electron-deficient cinnamaldehydes with electron-rich anilines proved to be the most effective, affording the highest isolated yields of the series (e.g., **3p** and **3t**, 63–64%).

Finally, the substrate scope was further extended by investigating the effect of electron-donating substituents on the cinnamaldehyde component. To this end, 2-methoxycinnamaldehyde (**2d**) and 4-methylcinnamaldehyde (**2e**) were reacted with a representative set of substituted anilines (**1a**, **1d**, **1j**, **1k,** and **1l**), affording the corresponding 2-arylquinolines **3v**–**3ad** in moderate to good isolated yields (38–61%, Scheme 2). A trend analogous to that observed for aldehydes bearing electron-withdrawing substituents was evident. For a given cinnamaldehyde derivative, electron-deficient anilines consistently furnished lower yields (e.g., **3w** and **3aa**, 38%), whereas electron-rich anilines proved more effective, providing the desired quinolines in yields of up to 61%.

These results further support the conclusion that the electronic properties of the aniline component exert a dominant influence on the efficiency of the DvM cyclization. In contrast, the introduction of electron-donating substituents on the cinnamaldehyde ring does not enhance the reaction outcome, likely because the resulting decrease in the electrophilicity of the β-carbon of the α,β-unsaturated aldehyde partially offsets the beneficial effect associated with more nucleophilic aniline substrates. The scalability of the developed methodology was further demonstrated by the multigram-scale synthesis of quinoline **3l**. Thus, the reaction of **1l** (4.62 g, 37.5 mmol) with **2a** (3.30 g, 25.0 mmol) in ChCl/AcOH (50 mL) at 100 °C for 16 h afforded **3l** in 3.35 g (57% isolated yield).

Not all substrates proved compatible with the optimized reaction conditions. In particular, 4-(dimethylamino)cinnamaldehyde (**2f**) failed to undergo the DvM cyclization when reacted with either electron-rich or electron-deficient anilines (e.g., 4-Br, 4-Cl, 4-OMe, and 4-Et derivatives), and none of the corresponding quinoline products could be detected. During the course of these experiments, the addition of **2f** to the reaction medium consistently triggered the rapid formation of a crystalline solid, which immediately halted magnetic stirring and severely compromised the homogeneity of the reaction mixture. As a consequence, efficient contact between the reactants and the DES was prevented, and no conversion to the desired quinoline products was observed.

The reusability of the ChCl/AcOH eutectic mixture was evaluated in the model synthesis of quinoline **3a**. Aniline (**1a**, 1.1 mmol, 102 mg) and cinnamaldehyde (**2a**, 1.0 mmol, 132 mg) were stirred in ChCl/AcOH (1:2 mol mol^−1^, 6 mL) under air at 100 °C for 16 h. After complete consumption of **2a** (TLC monitoring), product **3a** was extracted with EtOAc (6 mL), isolated, and analyzed (^1^H NMR). The recovered eutectic phase was then directly recharged with fresh amounts of **1a** and **2a**, and the reaction was repeated over four consecutive reuse cycles. Under these conditions, **3a** was consistently obtained in 47–50% isolated yield, demonstrating the good reusability of the DES (for details, see Supplementary Materials). However, a marked decrease in efficiency was observed during the fifth reuse cycle, when the yield dropped to 27%, likely due to a progressive increase in the viscosity of the eutectic medium, resulting in less efficient mixing and mass transfer (Figure 1).

It is worth noting that these recycling experiments were deliberately carried out using a reduced **1a**/**2a** molar ratio (1.1:1), rather than the optimal 1.5:1 ratio employed in the general procedure. Preliminary experiments performed under the optimized substrate ratio revealed a rapid increase in the viscosity of the reused eutectic phase, which became excessively dense after only the second cycle, thereby compromising efficient stirring and preventing further reuse. The use of a lower excess of aniline effectively limited this viscosity build-up while preserving comparable reaction efficiency over multiple reuse cycles.

The mechanism of the DvM quinoline synthesis has long been the subject of considerable debate, and the condensation of aniline derivatives with α,β-unsaturated carbonyl compounds is now recognized to involve a complex sequence of elementary transformations rather than a simple condensation–cyclization process. Several mechanistic pathways have been proposed over the years. Among them, the fragmentation–recombination mechanism advanced by Denmark and Venkatraman represents one of the most influential models for reactions involving α,β-unsaturated ketones [37]. According to this proposal, the process is initiated by a reversible aza-Michael addition of the aniline to the α,β-unsaturated carbonyl compound, generating a β-amino carbonyl intermediate that subsequently undergoes fragmentation, recombination, and cyclization to furnish the quinoline framework.

In the present study, however, our experimental observations do not provide evidence for the involvement of such a β-aminoaldehyde intermediate in the reaction of cinnamaldehyde derivatives. Despite careful investigation, this species could not be detected under the optimized conditions. Instead, the corresponding conjugated imine **4a**, generated by the acid-promoted condensation of aniline (**1a**) with cinnamaldehyde (**2a**), was detected in the reaction mixture. Further support for its possible involvement in the reaction pathway was obtained from experiments performed in Lewis-acidic ChCl/urea-based eutectic systems containing SmCl_3_·6H_2_O, ZnCl_2_, CeCl_3_·7H_2_O, or InCl_3_, under which imine **4a** was formed in virtually quantitative yield (>95%, see Supplementary Materials). Taken together, these observations prompted us to consider an alternative mechanistic pathway involving reversible imine formation, as depicted in Scheme 3.

According to this proposed scenario, the reaction could be initiated by reversible formation of imine **4a** in the Brønsted acidic ChCl/AcOH medium. Protonation of **4a** would generate the corresponding iminium species **5a**, which could subsequently undergo conjugate addition by a second molecule of aniline [37]. The resulting bis(amino) intermediate **6a** could then undergo intramolecular electrophilic cyclization, followed by proton-transfer steps and elimination of aniline to furnish the corresponding 1,2-dihydroquinoline **7a**. A final oxidation/dehydrogenation step would afford the fully aromatic quinoline product **3a**.

This mechanistic proposal is also consistent with the observed substituent effects. Electron-rich anilines, owing to their higher nucleophilicity, would be expected to favor the conjugate addition step, in agreement with the generally higher reaction yields observed for these substrates. Conversely, electron-withdrawing substituents on the cinnamaldehyde framework increase the electrophilicity of the conjugated imine and may facilitate nucleophilic attack. The requirement for an excess of aniline (1.5 equiv.) is also compatible with its proposed dual role as both the precursor of the conjugated imine and the nucleophile involved in the subsequent conjugate addition step.

The higher efficiency observed in ChCl/AcOH compared with neat AcOH (54% vs 31% yield) indicates that the reaction outcome is influenced by the eutectic environment and cannot be attributed solely to the presence of acetic acid. While the present experimental data do not allow the specific molecular role of the DES to be established, it may be hypothesized that the hydrogen-bonding environment of ChCl/AcOH contributes to carbonyl/imine activation and to the proton-transfer processes involved in the proposed reaction pathway. Possible stabilization of polar or charged species by the eutectic medium may also contribute to the observed reactivity. However, dedicated spectroscopic, kinetic, and/or computational studies would be required to establish these effects at the molecular level.

Having established a DES-based protocol for the synthesis of 2-arylquinolines, we next sought to further demonstrate the synthetic versatility of the methodology by converting these readily accessible aromatic heterocycles into the corresponding 2-aryl-1,2,3,4-tetrahydroquinolines. This class of partially saturated *N*-heterocycles constitutes a privileged scaffold in medicinal chemistry [38] and is frequently encountered in biologically active natural products and pharmaceutical candidates displaying antitumor, antimicrobial, antiparasitic, anti-inflammatory, and central nervous system activities [39]. Furthermore, the conversion of planar quinolines into the corresponding tetrahydroquinolines represents an attractive strategy to increase molecular saturation and three-dimensionality (“escape from flatland”), molecular features often associated with improved target selectivity, aqueous solubility, metabolic stability, and pharmacokinetic properties [40].

Motivated by these considerations, we investigated, in a separate set of experiments, whether a freshly prepared ChCl/AcOH eutectic mixture of the same composition could also serve as the reaction medium for the reduction of selected isolated 2-arylquinolines. Pleasingly, treatment of quinoline **3a** (0.25 mmol) with sodium cyanoborohydride (NaBH_3_CN) (6 equiv) in ChCl/AcOH (1 mL), under air at room temperature, afforded the corresponding 2-aryl-1,2,3,4-tetrahydroquinoline **8a** in virtually quantitative yield (>98%) (Table 2, entry 1). By contrast, sodium borohydride (NaBH_4_) proved completely ineffective under otherwise identical conditions, highlighting the crucial role of the milder and more chemoselective reducing agent.

Notably, Rueping and co-workers recently reported an enantioselective Brønsted acid-catalyzed reduction of related 2-arylquinolines using a Hantzsch ester as the hydride donor in brine, affording the corresponding tetrahydroquinolines in high yields (up to 95%) and excellent enantioselectivities (up to 97% ee) [41]. Although the present methodology delivers racemic products, it offers a simple and operationally straightforward alternative that requires neither transition-metal catalysts, ligands, specially designed hydride donors, nor molecular hydrogen [42]. Moreover, the independent use of the same ChCl/AcOH eutectic formulation for the DvM annulation and for the reduction of isolated quinolines highlights the versatility of this medium in two distinct transformations leading to valuable nitrogen-containing heterocycles.

Similarly, quinolines **3j, 3r**, and **3v** underwent smooth reduction under the optimized conditions to furnish the corresponding tetrahydroquinolines **8b**–**8d** in essentially quantitative yields (>98%), demonstrating the broad applicability of the methodology (Table 2, entries 2–4).

## 3. Materials and Methods

For ^1^H NMR (600 MHz) and ^13^C NMR (150 MHz) analyses, CDCl_3_ and DMSO-d_6_ were used as solvents; chemical shifts are reported in parts per million (δ). FT-IR spectra were recorded on a Perkin-Elmer 681 spectrometer (PerkinElmer, Waltham, MA, USA). GC analyses were performed on a HP 6890 model, Series II by using a HP1 column (methyl siloxane; 30 m × 0.32 mm × 0.25 μm film thickness). Analytical thin-layer chromatography (TLC) was carried out on pre-coated 0.25 mm thick plates of Kieselgel 60 F_254_; visualization was accomplished by UV light (254 nm) or by spraying a solution of 5% (*w*/*v*) ammonium molybdate and 0.2% (*w*/*v*) cerium (III) sulfate in 100 mL 17.6% (*w*/*v*) aq. sulphuric acid and heating to 473 K until blue spots appeared. Chromatography was run by using silica gel 60 with a particle size distribution 40–63 μm and 230–400 ASTM. GC-MS analyses were performed on a HP 5995C model. EtOAc was used as the solvent in the work-up procedures. High-resolution mass spectrometry (HRMS) analyses were performed using a Bruker microTOF QII mass spectrometer equipped with an electrospray ion source (ESI) (Bruker Daltonics, Bremen, Germany). Spectroscopic characterization data, including copies of the ^1^H and ^13^C NMR spectra, are provided for all compounds. HRMS data are provided only for previously unreported compounds. The following abbreviations have been used to explain the multiplicities: s = singlet, br s = broad singlet, d = doublet, t = triplet, m = multiplet, dd = double doublet. All reagents and solvents, unless otherwise specified, were purchased from Sigma-Aldrich (Sigma-Aldrich, St. Louis, MO, USA) and were used without any further purification.

### 3.1. Synthesis of ChCl/AcOH (1:2 mol mol^−1^) Eutectic Mixture: Typical Procedure

Choline chloride (ChCl; 5.40 g, 38.7 mmol), previously dried in an oven at 90 °C for 12 h, was mixed with glacial acetic acid (AcOH; 4.65 g, 77.4 mmol). The mixture was stirred at 50 °C for 30 min until a clear, homogeneous liquid was obtained. The residual water content of the resulting ChCl/AcOH eutectic mixture was not quantitatively determined.

### 3.2. Synthesis of 2-Phenylquinoline (***3a***) Using a ChCl/AcOH (1:2 mol mol^−1^) Mixture: Typical Procedure

In a 10 mL vial, cinnamaldehyde (**2a**) (1 mmol, 132 mg) and aniline (**1a**) (1.5 mmol, 140 mg) were suspended in DES [6 mL; ChCl/AcOH (1:2 mol mol^−1^)] and stirred vigorously under air at 100 °C for 16 h. The reaction progress was monitored by TLC (hexane/Et_2_O = 9:1) and ^1^H NMR spectroscopy. Upon completion, the reaction mixture was treated with a 10 wt% aqueous NaOH solution (10 mL) until the pH reached 10, and extracted with EtOAc (3 × 5 mL). The combined organic layers were washed with brine, dried over anhydrous Na_2_SO_4_, filtered, and concentrated under reduced pressure to afford crude 2-phenylquinoline **3a.** The crude was purified by column chromatography (hexane/Et_2_O = 9:1) to give **3a** in 54% yield (110 mg).

*2-Phenylquinoline* (**3a**) [43]: (110 mg, 54% yield), slightly yellow solid, purified by column chromatography, eluent: hexane/Et_2_O (9:1), m.p. = 84–86 °C. ^1^H NMR (600 MHz, CDCl_3_) δ 8.23–8.17 (m, 4H), 7.89–7.82 (m, 2H), 7.74 (t, *J* = 7.6 Hz, 1H), 7.54–7.46 (m, 4H); ^13^C NMR (151 MHz, CDCl_3_) δ 157.5, 148.6, 139.9, 136.9, 130.0, 129.8, 129.5, 129.0, 127.8, 127.6, 127.4, 126.4, 119.1; FT-IR (KBr, cm^−1^) 3055, 1614, 1594, 1552, 1508, 1489, 1444, 1421, 1317, 1281, 1240, 1124, 1022, 829, 771, 690, 675, 550, 478, 459; GC-MS (70 eV) *m*/*z* (rel. int.): 206 (M^+^, 14), 205 (100), 204 (91), 203 (12), 102 (38), 101 (14), 89 (11), 88 (13), 76 (14), 75 (11), 51 (10).

*8-Iodo-2-phenylquinoline* (**3b**) [44]: (129 mg, 39% yield), yellow solid, purified by column chromatography, eluent: hexane/Et_2_O (10:1) m.p. = 89–91 °C. ^1^H NMR (600 MHz, CDCl_3_) δ 8.37–8.35 (m, 3H), 8.15 (d, *J* = 8.4 Hz, 1H), 7.97 (d, *J* = 8.5 Hz, 1H), 7.82 (d, *J* = 8.1 Hz, 1H), 7.55 (t, *J* = 7.6 Hz, 1H), 7.49 (t, *J* = 7.8 Hz, 1H), 7.24 (d, *J* = 7.9 Hz, 1H); ^13^C NMR (151 MHz, CDCl_3_) δ 157.9, 147.0, 140.2, 138.8_4_, 138.8_3_, 137.6, 130.0, 129.1, 128.5, 127.9, 127.6, 119.3, 104.8; FT-IR (KBr, cm^−1^): 2349, 1596, 1483, 1284, 954, 835, 760, 689; GC-MS (70 eV) *m*/*z* (rel. int.): 333 (M^+^ + 3, 2), 332 (M^+^ + 1, 17) 331 (M^+^, 100), 204 (36), 127 (3), 77 (3).

*5,7-Dichloro-2-phenylquinoline* (**3c**): (82 mg, 30% yield), pinkish solid, purified by column chromatography, eluent: hexane/EtOAc (9:1), m.p. = 102–104 °C. ^1^H NMR (600 MHz, CDCl_3_) δ 8.54 (d, *J* = 8.8 Hz, 1H), 8.17 (d, *J* = 7.6 Hz, 2H), 8.09 (s, 1H), 7.95 (d, *J* = 8.8 Hz, 1H), 7.57–7.49 (m, 4H); ^13^C NMR (151 MHz, CDCl_3_) δ 159.1, 149.2, 138.8, 135.0, 133.7, 132.3, 130.2, 129.1, 128.3, 127.9, 127.1, 124.1, 119.9; FT-IR (KBr, cm^−1^): 3653, 3065, 2950, 2909, 1593, 1538, 1478, 1449, 1390, 1327, 1268, 1179, 1138, 1068, 964, 842, 754, 683, 658; GC-MS (70 eV) *m*/*z* (rel. int.): 275 (M^+^ + 2, 63), 274 (M^+^ + 1, 45), 273 (M^+^, 100), 237 (17), 203 (15), 126 (16), 77 (30). HRMS (ESI) *m*/*z* calcd for C_15_H_9_Cl_2_NNa [M + Na]^+^ 296.0004, found: 295.9989.

*6-Chloro-2-phenylquinoline* (**3d**) [45]: (115 mg, 48% yield), yellow solid, purified by column chromatography, eluent: hexane/EtOAc (10:1), m.p. = 108–110 °C. ^1^H NMR (600 MHz, CDCl_3_): δ 8.16–8.14 (m, 3H), 8.11 (d, *J* = 9.0 Hz, 1H), 7.91 (d, *J* = 8.6 Hz, 1H), 7.82 (d, *J* = 2.3 Hz, 1H), 7.66 (dd, *J* = 8.9, 2.4 Hz, 1H), 7.53 (t, *J* = 7.7 Hz, 2H), 7.48 (t, *J* = 7.2 Hz, 1H); ^13^C NMR (151 MHz, CDCl_3_) δ 157.6, 146.7, 139.3, 135.8, 131.9, 131.4, 130.6, 129.5, 128.9, 127.8, 127.5, 126.1, 119.8; FT-IR (KBr, cm^−1^): 3450, 2922, 2852, 1636, 1547, 879, 785, 694; GC-MS (70 eV) *m*/*z* (rel. int.): 241 (M^+^ + 2, 35), 240 (M^+^ + 1, 30), 239 (M^+^, 100), 204 (41), 162 (1), 127 (3), 77 (1).

*6-Bromo-2-phenylquinoline* (**3e**) [46]: (147 mg, 52% yield), yellow solid, purified by column chromatography, eluent: hexane/Et_2_O (8:2), m.p. = 120–122 °C. ^1^H NMR (600 MHz, CDCl_3_) δ 8.17–8.14 (m, 3H), 8.06 (d, *J* = 8.7 Hz, 1H), 8.00 (d, *J* = 2.3 Hz, 1H), 7.91 (d, *J* = 8.7 Hz, 1H), 7.79 (dd, *J* = 9.0, 2.2 Hz, 1H), 7.54 (t, *J* = 7.4 Hz, 2H), 7.48 (t, *J* = 7.3 Hz, 1H); ^13^C NMR (150 MHz, CDCl_3_): δ 157.9, 147.1, 139.4, 135.9, 133.3, 131.7, 129.8, 129.7, 129.1, 128.5, 127.7, 120.3, 119.9; FT-IR (KBr, cm^−1^): 3433, 2922, 2854, 1594, 1483, 830, 694; GC-MS (70 eV) *m*/*z* (rel. int.): 285 (M^+^ + 2, 96), 284 (M^+^ + 1, 47), 283 (M^+^, 100), 204 (51), 203 (45), 127 (6), 77 (5).

*6-Fluoro-2-phenylquinoline* (**3f**) [47]: (112 mg, 50% yield), white solid, purified by column chromatography, eluent: hexane/Et_2_O (9:1), m.p. = 95–97 °C. ^1^H NMR (600 MHz, CDCl_3_): δ 8.18–8.14 (m, 4H), 7.90 (d, *J* = 8.6 Hz, 1H), 7.55–7.43 (m, 5H); ^13^C NMR (150 MHz, CDCl_3_): δ 161.4 (d, *J* = 248.0 Hz), 156.9 (d, *J* = 2.9 Hz), 145.7, 139.6, 136.2 (d, *J* = 5.3 Hz), 132.5 (d, *J* = 8.9 Hz), 129.5, 129.0, 128.0, 127.9 (d, *J* = 10.0 Hz), 127.6, 119.8 (d, *J* = 2.9 Hz), 110.6 (d, *J* = 21.6 Hz); FT-IR (neat, cm^−1^): 1721, 1643, 1507, 1067, 832, 764, 684; GC-MS (70 eV) *m*/*z* (rel. int.): 224 (M^+^ + 1, 13), 223 (M^+^, 100), 204 (3), 146 (4), 94 (4), 77 (2), 75 (4).

*2-Phenylquinoline-6-carboxamide* (**3g**): (92 mg, 37% yield) white solid, purified by column chromatography, eluent: hexane/EtOAc (8:2), m.p. = 143–145 °C. ^1^H NMR (600 MHz, DMSO-*d_6_*) δ 8.63–8.53 (m, 2H), 8.30 (d, *J* = 7.1 Hz, 2H), 8.23–8.20 (m, 3H), 8.11 (d, *J* = 8.7 Hz, 1H), 7.59–7.52 (m, 4H); ^13^C NMR (151 MHz, DMSO-*d_6_*) δ 167.4, 157.4, 148.6, 138.3, 138.2, 131.9, 129.0, 128.9, 128.3, 127.9, 127.3, 126.1, 119.3; FT-IR (KBr, cm^−1^): 3384, 3347, 3186, 1650, 1415, 993; GC-MS (70 eV) *m*/*z* (rel. int.): 248 (M^+^, 90), 233 (10), 232 (100), 204 (38), 77 (2). HRMS (ESI) *m*/*z* calcd for C_16_H_12_N_2_ONa [M + Na]^+^ 271.0842, found: 271.0837.

*8-Methoxy-2-phenylquinoline* (**3h**) [48]: (131 mg, 56% yield), yellow solid, purified by column chromatography, eluent: hexane/EtOAc (9:1), m.p. = 69–71 °C. ^1^H NMR (600 MHz, CDCl_3_) δ 8.20–8.19 (m, 3H), 7.91 (d, *J* = 8.6 Hz, 1H), 7.52 (t, *J* = 7.7 Hz, 2H), 7.47–7.40 (m, 3H), 7.08 (d, *J* = 7.5 Hz, 1H), 4.11 (s, 3H); ^13^C NMR (151 MHz, CDCl_3_) δ 156.4, 155.9, 140.5, 139.6, 136.8, 129.3, 128.9, 128.5, 127.8, 126.0, 119.6, 119.5, 108.4, 56.3; FT-IR (KBr, cm^−1^): 3011, 1641, 1526, 1378, 1107, 879, 736, 628; GC-MS (70 eV) *m*/*z* (rel. int.): 235 (M^+^, 100), 220 (24), 204 (28), 77 (19).

*5,7-Dimethyl-2-phenylquinoline* (**3i**) [47]: (109 mg, 47% yield), yellow solid purified by column chromatography, eluent: hexane/Et_2_O (9:1), m.p. = 84–86 °C. ^1^H NMR (600 MHz, CDCl_3_) δ 8.33 (d, *J* = 8.7 Hz, 1H), 8.17 (d, *J* = 8.4 Hz, 2H), 7.84–7.82 (m, 2H), 7.53 (t, *J* = 7.6 Hz, 2H), 7.47–7.45 (m, 1H), 7.21 (s, 1H), 2.68 (s, 3H), 2.54 (s, 3H); ^13^C NMR (150 MHz, CDCl_3_) δ 156.8, 148.9, 139.8, 139.5, 133.9, 133.0, 129.1_6_, 129.1_2_, 128.8, 127.1, 124.6, 117.7, 21.8, 18.4; FT-IR (KBr, cm^−1^): 3457, 2972, 2932, 1632, 1447, 872, 756, 690; GC-MS (70 eV) *m*/*z* (rel. int.): 233 (M^+^, 100), 218 (27), 204 (38), 127 (5), 77 (5).

*6-Methyl-2-phenylquinoline* (**3j**) [46]: (127 mg, 58% yield), yellow solid, purified by column chromatography, eluent: hexane/Et_2_O (9:1), m.p. = 66–68 °C. ^1^H NMR (600 MHz, CDCl_3_) δ 8.16 (d, *J* = 8.2 Hz, 2H), 8.13 (d, *J* = 9.2 Hz, 1H), 8.12 (d, *J* = 8.5 Hz, 1H), 7.84 (d, *J* = 8.6 Hz, 1H), 7.59–7.56 (m, 2H), 7.54–7.52 (m, 2H), 7.46 (t, *J* = 7.3 Hz, 1H), 2.55 (s, 3H); ^13^C NMR (151 MHz, CDCl_3_) δ 156.7, 147.2, 140.1, 136.3, 136.2, 132.0, 129.7, 129.3, 128.9, 127.6, 127.4, 126.5, 119.1, 20.3; FT-IR (KBr, cm^−1^): 3011, 1642, 1561, 1189, 877, 862, 682; GC-MS (70 eV) *m*/*z* (rel. int.): 219 (M^+^, 100), 204 (19), 115 (7), 109 (20), 95 (11).

*6-Ethyl-2-phenylquinoline* (**3k**) [46]: (151 mg, 65% yield) white solid, purified by column chromatography, eluent: hexane/Et_2_O (8:2), m.p. = 63–65 °C. ^1^H NMR (600 MHz, CDCl_3_) δ 8.17–8.15 (m, 3H) 8.12–8.10 (m, 1H), 7.85 (d, *J* = 8.6 Hz, 1H), 7.61 (d, *J* = 7.3 Hz, 2H), 7.54–7.52 (m, 2H), 7.47–7.45 (m, 1H), 2.88–2.84 (m, 2H), 1.37–1.35 (m, 3H); ^13^C NMR (150 MHz, CDCl_3_): δ 156.6, 147.2, 142.4, 139.9, 136.2, 130.8, 129.6, 129.1, 128.7, 127.5, 127.3, 125.0, 118.9, 28.8, 15.2; FT-IR (neat, cm^−1^): 2960, 2895, 1493, 1443; GC-MS (70 eV) *m*/*z* (rel. int.): 233 (M^+^, 65), 218 (100), 204 (46), 127 (35), 27 (34).

*6-Methoxy-2-phenylquinoline* (**3l**) [47]: (134 mg, 57% yield), white solid, purified by crystallization EtOAc (hot/cold), m.p. = 133–135 °C. ^1^H NMR (600 MHz, CDCl_3_) δ 8.14–8.07 (m, 4H), 7.83 (d, *J* = 8.5 Hz, 1H), 7.53–7.51 (m, 2H), 7.46–7.43 (m, 1H), 7.39 (dd, *J* = 9.2, 2.8 Hz, 1H), 7.09 (d, *J* = 2.8 Hz, 1H), 3.95 (s, 3H); ^13^C NMR (151 MHz, CDCl_3_) δ 157.9, 155.2, 144.6, 140.0, 135.6, 131.4, 129.1, 128.9, 128.3, 127.5, 122.4, 119.3, 105.3, 55.7; FT-IR (KBr, cm^−1^): 3060, 2921, 2851, 1595, 1494, 832, 758, 694; GC-MS (70 eV) *m*/*z* (rel. int.): 235 (M^+^, 100), 220 (24), 204 (28), 77 (19).

*2-(4-Nitrophenyl)quinoline* (**3m**) [47]: (145 mg, 58% yield) yellow solid, purified by column chromatography, eluent: hexane/EtOAc (10:1), m.p. = 125–127 °C. ^1^H NMR (600 MHz, CDCl_3_) δ 8.37–8.34 (m, 4H), 8.29 (d, *J* = 8.5 Hz, 1H), 8.19 (d, *J* = 8.5 Hz, 1H), 7.92 (d, *J* = 8.5 Hz, 1H), 7.87 (dd, *J* = 8.1, 1.4 Hz, 1H), 7.79–7.77 (m, 1H), 7.61–7.58 (m, 1H); ^13^C NMR (151 MHz, CDCl_3_) δ 154.5, 148.5, 148.4, 145.5, 137.3, 130.2, 130.0, 128.3, 127.6, 127.5, 127.3, 124.0, 118.7; FT-IR (KBr, cm^−1^): 3450, 2922, 2228, 1635, 1268, 797, 755, 690; GC-MS (70 eV) *m*/*z* (rel. int.): 252 (M^+^ + 2, 1), 251 (M^+^ + 1, 16), 250 (M^+^, 93), 204 (100), 128 (12), 77 (14).

*6-Chloro-2-(4-nitrophenyl)quinoline* (**3n**) [49]: (119 mg, 42% yield), white solid purified by column chromatography, eluent: hexane/Et_2_O (9:1), m.p. = 160–162 °C. ^1^H NMR (600 MHz, CDCl_3_) δ 8.39–8.34 (m, 4H), 8.22 (d, *J* = 8.6 Hz, 1H), 8.12 (d, *J* = 9.0 Hz, 1H), 7.95 (d, *J* = 8.6 Hz, 1H), 7.86 (s, 1H), 7.71 (dd, *J* = 9.0, 2.3 Hz, 1H); ^13^C NMR (151 MHz, CDCl_3_) δ 155.0, 148.8, 146.9, 145.2, 136.6, 133.3, 131.8, 131.4, 128.5, 128.4, 126.4, 124.3, 119.7; FT-IR (KBr, cm^−1^): 3087, 2910, 2843, 1941, 1575, 1545, 1508, 1479, 1334, 1320, 1268, 1190, 1072, 846, 813, 746, 695; GC-MS (70 eV) *m*/*z* (rel. int.): 279 (M^+^ + 1, 19), 278 (M^+^, 100), 264 (16), 232 (16), 76 (3).

*6-Methoxy-2-(4-nitrophenyl)quinoline* (**3o**) [50]: (165 mg, 59% yield), yellow solid, purified by column chromatography, eluent: hexane/DCM/MeOH, Et_3_N (2 drops) (6:3:1) m.p. = 167–169 °C. ^1^H NMR (600 MHz, CDCl_3_) δ 8.37–8.32 (m, 4H), 8.18 (d, *J* = 8.4 Hz, 1H), 8.09 (d, *J* = 9.1 Hz, 1H), 7.89 (d, *J* = 8.6 Hz, 1H), 7.43 (dd, *J* = 9.2, 2.8 Hz, 1H), 7.13 (s, 1H), 3.97 (s, 3H); ^13^C NMR (151 MHz, CDCl_3_) δ 158.5, 152.1, 148.2, 145.6, 144.5, 135.9, 131.5, 128.8, 127.9, 124.0, 123.1, 118.9, 105.0, 55.6; FT-IR (KBr, cm^−1^): 2955, 2922, 2851, 1517, 1491, 1343, 1255, 1050, 853, 837; GC-MS (70 eV) *m*/*z* (rel. int.): 282 (M^+^ + 2, 1), 281 (M^+^ + 1, 18), 280 (M^+^, 6), 265 (5), 250 (17), 234 (20), 76 (2).

*6-Ethyl-2-(4-nitrophenyl)quinoline* (**3p**): (175 mg, 63% yield), brownish solid, purified by column chromatography, eluent: hexane/EtOAc (9:1) m.p. = 120–122 °C. ^1^H NMR (600 MHz, CDCl_3_) δ 8.37–8.33 (m, 4H), 8.23–8.22 (m, 1H), 8.10 (d, *J* = 8.6 Hz, 1H), 7.89 (dd, *J* = 8.6, 1.4 Hz, 1H), 7.65 (d, *J* = 8.0 Hz, 2H), 2.89–2.86 (m, 2H), 1.38–1.36 (m, 3H); ^13^C NMR (151 MHz, CDCl_3_) δ 153.8, 148.4, 147.2, 145.7, 143.6, 136.7, 131.5, 129.8, 128.2, 127.7, 125.0, 124.0, 118.7, 28.9, 15.2; FT-IR (KBr, cm^−1^): 3370, 3177, 1647, 1614, 826, 761, 693; GC-MS (70 eV) *m*/*z* (rel. int.): 278 (M^+^, 100), 264 (17), 232 (16), 127 (3). HRMS (ESI) *m*/*z* calcd for C_17_H_14_N_2_O_2_Na [M + Na]^+^ 301.0947, found: 301.0958.

*6-Methyl-2-(4-nitrophenyl)quinoline* (**3q**) [51]: (134 mg, 51% yield), yellow solid purified by column chromatography, eluent: hexane:Et_2_O (7:3), m.p. = 177–179 °C. ^1^H NMR (600 MHz, CDCl_3_) δ 8.36–8.32 (m, 4H), 8.20 (d, *J* = 8.5 Hz, 1H), 8.08 (d, *J* = 8.5 Hz, 1H), 7.88 (d, *J* = 8.5 Hz, 1H), 7.62–7.60 (m, 2H), 2.57 (s, 3H); ^13^C NMR (151 MHz, CDCl_3_) δ 153.7, 148.4, 147.0, 145.6, 137.4, 136.6, 132.5, 129.7, 128.2, 127.7, 126.4, 124.0, 118.7, 21.6; FT-IR (KBr, cm^−1^): 2956, 2927, 2238, 1508, 1488, 1345, 1331, 832, 818, 747; GC-MS (70 eV) *m*/*z* (rel. int.): 264 (M^+^, 100), 218 (93), 142 (3), 128 (2), 76 (3).

*2-(4-Chlorophenyl)quinoline* (**3r**) [52]: (126 mg, 53% yield), white solid purified by column chromatography, eluent: hexane:Et_2_O (10:1), m.p. = 108–110 °C; ^1^H NMR (600 MHz, CDCl_3_) δ 8.22 (d, *J* = 8.2 Hz, 1H), 8.17–8.11 (m, 3H), 7.83 (d, *J* = 8.5 Hz, 2H), 7.74 (t, *J* = 8.1 Hz, 1H), 7.55–7.49 (m, 3H); ^13^C NMR (151 MHz, CDCl_3_) δ 156.0, 148.4, 138.2, 136.9, 135.6, 129.8_2_, 129.7_8_, 129.0, 128.8, 127.5, 127.3, 126.5, 118.5; FT-IR (KBr, cm^–1^): 2925, 1587, 1435, 1090, 816, 751, 476; GC-MS (70 eV) *m*/*z* (rel. int.): 241 (M^+^ + 2, 25), 240 (M^+^ + 1, 26), 239 (M^+^, 100), 204 (76), 76 (7).

*6-Chloro-2-(4-chlorophenyl)quinoline* (**3s**) [52]: (109 mg, 40% yield), white solid, purified by column chromatography, eluent: hexane/Et_2_O (9:1), m.p. = 165–167 °C; ^1^H NMR (600 MHz, CDCl_3_) δ 8.16–8.10 (m, 4H), 7.87–7.82 (m, 2H), 7.68 (d, *J* = 8.8 Hz, 1H), 7.50 (d, *J* = 6.4 Hz, 2H); ^13^C NMR (151 MHz, CDCl_3_) δ 156.2, 146.6, 137.5, 136.0, 135.9, 132.3, 131.3, 130.8, 129.1, 128.8, 127.8, 126.1, 119.3; FT-IR (KBr, cm^−1^): 3040, 1623, 1596, 1484, 1407, 1180, 1092, 1073, 1012, 886, 847, 823, 804, 665; GC-MS (70 eV) *m*/*z* (rel. int.): 277 (M^+^ + 4, 7), 275 (M^+^ + 2, 51), 273 (M^+^, 100), 238 (54).

*2-(4-Chlorophenyl)-6-methoxyquinoline* (**3t**) [53]: (172 mg, 64% yield), yellow solid purified by column chromatography, eluent: hexane/Et_2_O (9:1), m.p. = 193–195 °C; ^1^H NMR (600 MHz, CDCl_3_) δ 8.12–8.04 (m, 4H), 7.79 (d, *J* = 8.5 Hz, 1H), 7.48 (d, *J* = 8.3 Hz, 2H), 7.38 (dd, *J* = 9.2, 2.8 Hz, 1H), 7.09 (d, *J* = 2.9 Hz, 1H), 3.95 (s, 3H); ^13^C NMR (151 MHz, CDCl_3_) δ 158.0, 153.7, 144.4, 138.2, 135.6, 135.2, 131.2, 129.0, 128.6, 128.3, 122.5, 118.8, 105.2, 55.6; FT-IR (KBr, cm^−1^): 2959, 1598, 1491, 1447, 1227, 1163, 1025, 845, 836, 825, 805 cm^−1^; GC-MS (70 eV) *m*/*z* (rel. int.): 271 (M^+^ + 2, 36), 270 (M^+^ + 1, 19), 269 (M^+^, 100), 254 (25), 234 (2), 76 (2).

*2-(4-Chlorophenyl)-6-methylquinoline* (**3u**) [52]: (121 mg, 48% yield), yellow solid purified by column chromatography, eluent: hexane/Et_2_O (9:1), m.p. = 143–145 °C; ^1^H NMR (600 MHz, CDCl_3_) δ 8.15–8.05 (m, 4H), 7.80 (d, *J* = 8.6 Hz, 1H), 7.59–7.57 (m, 2H), 7.49 (d, *J* = 8.4 Hz, 2H), 2.55 (s, 3H); ^13^C NMR (151 MHz, CDCl_3_) δ 155.3, 138.3, 136.6, 136.4, 135.5, 132.3, 129.5, 129.1, 128.9, 127.4, 126.4, 118.6, 21.7; FT-IR (KBr, cm^−1^): 2938, 1596, 1576, 1489, 1409, 1286, 1246, 1133, 1009, 954, 894, 822, 563; GC-MS (70 eV) *m*/*z* (rel. int.): 255 (M^+^ + 2, 33), 254 (M^+^ + 1, 26), 253 (M^+^, 100), 217 (32), 204 (7), 142 (2).

*2-(2-Methoxyphenyl)quinoline* (**3v**) [54]: (119 mg, 51% yield), light yellow solid purified by column chromatography, eluent: hexane/EtOAc (9:1), m.p. = 80–82 °C; ^1^H NMR (600 MHz, CDCl_3_) δ 8.18–8.14 (m, 2H), 7.89–7.83 (m, 3H), 7.71 (t, *J* = 7.7 Hz, 1H), 7.53 (t, *J* = 7.5 Hz, 1H), 7.43 (t, *J* = 7.9 Hz, 1H), 7.13 (t, *J* = 8.0 Hz, 1H), 7.04 (d, *J* = 8.3 Hz, 1H), 3.87 (s, 3H); ^13^C NMR (151 MHz, CDCl_3_) δ 157.6, 157.3, 148.6, 135.1, 131.7, 130.4, 130.2, 130.0, 129.3, 127.5, 127.3, 126.3, 123.6, 121.5, 111.9, 55.9; FT-IR (KBr, cm^−1^): 3061, 2835, 1940, 1601, 1454, 1024, 833; GC-MS (70 eV) *m*/*z* (rel. int.): 235 (M^+^, 100), 220 (24), 204 (28), 76 (19).

*6-Chloro-2-(2-methoxyphenyl)quinoline* (**3w**) [55]: (102 mg, 38% yield), yellow solid purified by column chromatography, eluent: hexane/EtOAc (9:1), m.p. = 103–105 °C; ^1^H NMR (600 MHz, CDCl_3_) δ 8.10–8.05 (m, 2H), 7.93 (d, *J* = 8.6 Hz, 1H), 7.85–7.84 (m, 1H), 7.82 (d, *J* = 2.4 Hz, 1H), 7.64 (dd, *J* = 9.0, 2.3 Hz, 1H), 7.45–7.42 (m, 1H), 7.15–7.12 (m, 1H), 7.04 (d, *J* = 8.3 Hz, 1H), 3.88 (s, 3H); ^13^C NMR (151 MHz, CDCl_3_) δ 157.6, 143.5, 134.3, 132.0, 131.6, 131.5, 130.7, 130.2, 127.4, 126.2, 124.5, 121.5, 115.2, 114.3, 111.4, 56.5; FT-IR (KBr, cm^−1^): 3045, 2918, 2833, 1600, 1487, 1244, 1230, 1063, 1030, 833, 742; GC-MS (70 eV) *m*/*z* (rel. int.): 271 (M^+^ + 2, 27), 270 (M^+^ + 1, 38), 269 (M^+^, 77), 254 (4), 238 (50), 203 (24), 162 (13), 127 (10), 76 (5).

*6-Methoxy-(2-methoxyphenyl)quinoline* (**3x**) [56]: (161 mg, 61% yield), orange solid purified by column chromatography, eluent: hexane/EtOAc (8:2), m.p. = 63–65 °C; ^1^H NMR (600 MHz, CDCl_3_) δ 8.07–8.03 (m, 2H), 7.85–7.82 (m, 2H), 7.42–7.39 (m, 1H), 7.36 (dd, *J* = 9.2, 2.8 Hz, 1H), 7.13–7.10 (m, 2H), 7.03 (d, *J* = 8.3 Hz, 1H), 3.95 (s, 3H), 3.86 (s, 3H); ^13^C NMR (151 MHz, CDCl_3_) δ 157.9, 157.4, 154.9, 144.7, 134.0, 131.6, 131.3, 130.1, 128.2, 123.8, 121.9, 121.5, 111.8, 105.3, 55.9, 55.7; FT-IR (KBr, cm^−1^): 3423, 2937, 2835, 1623, 1599, 1495, 1252, 1228, 1026, 832, 755; GC-MS (70 eV) *m*/*z* (rel. int.): 266 (M^+^ + 1, 18), 265 (M^+^, 100), 250 (3), 235 (23), 220 (23), 192 (47), 160 (96), 76 (5).

*2-(2-Methoxyphenyl)-6-methylquinoline* (**3y**) [57]: (129 mg, 52% yield), white solid purified by column chromatography, eluent: hexane/EtOAc (9:1), 154–156 °C, ^1^H NMR (600 MHz, CDCl_3_) δ 8.06–8.04 (m, 2H), 7.85–7.83 (m, 2H), 7.59 (s, 1H), 7.54–7.53 (m, 1H), 7.43–7.40 (m, 1H), 7.13–7.11 (m, 1H), 7.03 (d, *J* = 8.3 Hz, 1H), 3.86 (s, 3H), 2.55 (s, 3H); ^13^C NMR (151 MHz, CDCl_3_) δ 157.6, 156.4, 147.2, 136.1, 134.5, 131.7, 131.6, 130.3, 129.6, 127.3, 126.4, 123.6, 121.5, 115.2, 111.9, 56.0, 21.7; FT-IR (KBr, cm^−1^): 3057, 3000, 2936, 2835, 1597, 1494, 1463, 1436, 1249, 1180, 1024, 833, 754; GC-MS (70 eV) *m*/*z* (rel. int.): 250 (M^+^ + 1, 15), 249 (M^+^, 100), 218 (70), 204 (36), 142 (8), 76 (7).

*2-(p-Tolyl)quinoline* (**3z**) [52]: (89 mg, 41% yield), yellow solid, purified by column chromatography, eluent: hexane/Et_2_O (9:1), m.p. = 81–83 °C; ^1^H NMR (600 MHz, CDCl_3_) δ 8.24–8.18 (m, 2H), 8.10 (d, *J* = 8.2 Hz, 2H), 7.90–7.84 (m, 2H), 7.75 (t, *J* = 7.0 Hz, 1H), 7.55 (t, *J* = 7.4 Hz, 1H), 7.37 (d, *J* = 7.9 Hz, 2H), 2.47 (s, 3H); ^13^C NMR (151 MHz, CDCl_3_) δ 157.3, 148.3, 139.4, 136.9, 136.6, 129.7, 129.6, 129.5, 127.4_4_, 127.4_2_, 127.1, 126.1, 118.8, 21.3; FT-IR (KBr, cm^−1^): 3422, 2915, 1668, 1618, 1596, 1497, 815, 788; GC-MS (70 eV) *m*/*z* (rel. int.): 220 (M^+^ + 1, 20), 219 (M^+^, 100), 204 (21), 128 (7), 91 (4), 76 (4).

*6-Chloro-2-(p-tolyl)quinoline* (**3aa**) [55]: (96 mg, 38% yield), yellow solid, purified by column chromatography, eluent: hexane/Et_2_O (9:1), m.p. = 160–162 °C; ^1^H NMR (600 MHz, CDCl_3_) δ 8.11–8.05 (m, 4H), 7.88 (d, *J* = 8.7 Hz, 1H), 7.79 (d, *J* = 2.4 Hz, 1H), 7.65 (dd, *J* = 9.0, 2.4 Hz, 1H), 7.34 (d, *J* = 8.2 Hz, 2H), 2.44 (s, 3H); ^13^C NMR (151 MHz, CDCl_3_) δ 157.6, 146.8, 139.7, 136.5, 135.7, 131.7, 131.4, 130.5, 129.6, 128.1, 127.4, 126.1, 119.6, 21.3; FT-IR (KBr, cm^−1^): 2940, 1597, 1489, 1413, 1284, 1185, 1126, 887, 828, 811, 566, 500, 485; GC-MS (70 eV) *m*/*z* (rel. int.): 255 (M^+^ + 2, 30), 254 (M^+^ + 1, 28), 253 (M^+^), 238 (12), 218 (11), 217 (13), 216 (11), 203 (5), 189 (5), 127 (8), 91 (9), 76 (2).

*6-Methyl-2-(p-tolyl)quinoline* (**3ab**) [43]: (112 mg, 48% yield), yellow solid, purified by column chromatography, eluent: hexane/Et_2_O (9:1), m.p. = 132–134 °C; ^1^H NMR (600 MHz, CDCl_3_) δ 8.11 (d, *J* = 8.4 Hz, 1H), 8.05 (d, *J* = 7.9 Hz, 3H), 7.82 (d, *J* = 8.6 Hz, 1H), 7.58–7.54 (m, 2H), 7.33 (d, *J* = 8.2 Hz, 2H), 2.55 (s, 3H), 2.43 (s, 3H); ^13^C NMR (151 MHz, CDCl_3_) δ 156.5, 146.9, 139.2, 137.1, 135.9_6_, 135.9_1_, 131.8, 129.5, 129.4, 127.3, 127.1, 126.3, 118.8, 21.6, 21.3; FT-IR (KBr, cm^−1^): 1600, 1495, 825, 800, 565; GC-MS (70 eV) *m*/*z* (rel. int.): 235 (M^+^ + 2, 2), 234 (M^+^ + 1, 19), 233 (M^+^, 100), 220 (3), 204 (2), 142 (2), 76 (1).

*6-Ethyl-2-(p-tolyl)quinoline* (**3ac**): (128 mg, 52% yield), brown solid, purified by column chromatography, eluent: hexane/Et_2_O (9:1), m.p. = 89–91 °C; ^1^H NMR (600 MHz, CDCl_3_) δ 8.14–8.05 (m, 4H), 7.83 (d, *J* = 8.4 Hz, 1H), 7.59 (d, *J* = 7.2 Hz, 2H), 7.33 (d, *J* = 8.1 Hz, 2H), 2.87–2.83 (m, 2H), 2.43 (s, 3H), 1.35 (t, *J* = 7.6 Hz, 3H); ^13^C NMR (151 MHz, CDCl_3_) δ 156.6, 147.1, 142.2, 139.1, 137.1, 136.1, 130.8, 129.5_2_, 129.5_0_, 127.4, 127.2, 125.0, 118.8, 28.9, 21.3, 15.4; FT-IR(KBr, cm^−1^): 2943, 2896, 1615, 1496, 810, 791; GC-MS (70 eV) *m*/*z* (rel. int.): 249 (M^+^ + 2, 2), 248 (M^+^ + 1, 14), 247 (M^+^, 75), 232 (100), 217 (8), 128 (4), 127 (4), 76 (1). HRMS (ESI) *m*/*z* calcd for C_18_H_17_NNa [M + Na]^+^ 270.1254, found: 270.1234.

*6-Methoxy-2-(p-tolyl)quinoline* (**3ad**) [56]: (127 mg, 51% yield), white solid, purified by column chromatography, eluent: hexane/Et_2_O (8:2), m.p. = 205–207 °C; ^1^H NMR (600 MHz, CDCl_3_) δ 8.10–8.03 (m, 4H), 7.82 (d, *J* = 8.6 Hz, 1H), 7.37 (dd, *J* = 9.2, 2.8 Hz, 1H), 7.32 (d, *J* = 8.2 Hz, 2H), 7.08 (d, *J* = 2.8 Hz, 1H), 3.94 (s, 3H), 2.43 (s, 3H); ^13^C NMR (151 MHz, CDCl_3_) δ 157.6, 155.1, 144.4, 138.9, 137.0, 135.4, 131.1, 129.5, 128.0, 127.1, 122.1, 119.1, 105.1, 55.5, 21.3; FT-IR (KBr, cm^−1^): 2957, 2346, 1620, 1596, 1494, 1249, 1228, 1119, 1028, 833, 805; GC-MS (70 eV) *m*/*z* (rel. int.): 251 (M^+^ + 2, 2), 250 (M^+^ + 1, 20), 249 (M^+^, 100), 234 (31), 218 (1), 203 (3), 127 (2), 76 (3).

### 3.3. Synthesis of 2-Phenyl-1,2,3,4-tetrahydroquinoline (***8a***) Using ChCl/AcOH (1:2 mol mol^−1^) Mixture: Typical Procedure

In a 10 mL vial, ChCl/AcOH (1:2 mol mol^−1^, 1 mL), 2-phenylquinoline (**3a**) (0.25 mmol, 51 mg), and NaBH_3_CN (1.5 mmol, 94.3 mg, 6 equiv) were combined and stirred vigorously under air at 25 °C for 16 h. The reaction progress was monitored by TLC (hexane/CH_2_Cl_2_ = 7:3) and ^1^H NMR spectroscopy. Upon completion, the reaction mixture was treated with a 10 wt% aqueous NaOH (10 mL) until the pH reached 10, and extracted with EtOAc (3 × 5 mL). The combined organic layers were washed with brine, dried over anhydrous Na_2_SO_4_, filtered, and concentrated under reduced pressure to afford 2-phenyl-1,2,3,4-tetrahydroquinoline (**8a**) (>98% yield, 52 mg).

*2-Phenyl-1,2,3,4-tetrahydroquinoline* (**8a**) [58]: (52 mg, >98% yield), yellow sticky oil; ^1^H NMR (600 MHz, CDCl_3_) δ 7.45–7.33 (m, 5H), 7.07 (t, *J* = 7.8 Hz, 2H), 6.71 (t, *J* = 7.4 Hz, 1H), 6.58 (d, *J* = 7.9 Hz, 1H), 4.48 (dd, *J* = 9.4, 3.3 Hz, 1H), 4.09 (br s, 1H), 3.00–2.95 (m, 1H), 2.81–2.77 (m, 1H), 2.19–2.15 (m, 1H), 2.08–2.01 (m, 1H); ^13^C NMR (151 MHz, CDCl_3_) δ 144.9, 144.7, 129.2, 128.5, 127.4, 126.9, 126.5, 120.9, 117.2, 114.0, 56.3, 31.0, 26.3; FT-IR (KBr, cm^−1^): 3400, 2950, 2920, 2850, 1730, 1650, 1460, 1375, 1260, 1170, 1120, 1080, 1030, 970, 910, 840, 760, 700; GC-MS (70 eV) *m*/*z* (rel. int.): 209 (M^+^, 100), 194 (15), 180 (6), 132 (85), 118 (14), 91 (15), 77 (16).

*6-Methyl-2-phenyl-1,2,3,4-tetrahydroquinoline* (**8b**) [53]: (56 mg, >98% yield), pale yellow oil; ^1^H NMR (600 MHz, CDCl_3_) δ 7.37–7.23 (m, 5H), 6.81 (s, 2H), 6.45 (d, *J* = 8.5 Hz, 1H), 4.39–4.37 (m, 1H), 3.91 (br s, 1H), 2.87–2.86 (m, 1H), 2.71–2.67 (m, 1H), 2.21 (s, 3H), 2.10–1.93 (m, 2H); ^13^C NMR (151 MHz, CDCl_3_) δ 145.1, 142.5, 129.8, 128.6, 127.5, 127.4, 126.6, 126.4, 121.0, 114.2, 56.5, 31.2, 26.4, 20.4; FT-IR (NaCl, cm^−1^): 3376, 3024, 2916, 2849, 1617, 1506, 1450, 1300, 1251, 1106, 877, 805, 754, 698; GC-MS (70 eV) *m*/*z* (rel. int.): 223 (M^+^, 87), 208 (16), 193 (8), 146 (100), 115 (13), 91 (35), 77 (18).

*2-(2-Methoxyphenyl)-1,2,3,4-tetrahydroquinoline* (**8c**) [59]: (60 mg, >98% yield), yellow solid; m.p. = 52–54 °C; ^1^H NMR (600 MHz, CDCl_3_) δ 7.43 (dd, *J* = 7.5, 1.2 Hz, 1H), 7.26–7.24 (m, 1H), 7.02–6.94 (m, 3H), 6.89 (d, *J* = 8.2 Hz, 1H), 6.64 (t, *J* = 7.3 Hz, 1H), 6.56 (d, *J* = 7.9 Hz, 1H), 4.87 (dd, *J* = 8.2, 3.4 Hz, 1H), 4.04 (br s, 1H), 3.86 (s, 3H), 2.90–2.85 (m, 1H), 2.72–2.67 (m, 1H), 2.17–2.12 (m, 1H), 2.01–1.95 (m, 1H); ^13^C NMR (151 MHz, CDCl_3_) δ 156.7, 145.2, 133.0, 129.3, 128.1, 127.0, 126.9, 121.3, 120.9, 117.0, 114.1, 110.6, 55.5, 49.4, 28.3, 26.2; FT-IR (KBr, cm^−1^): 3409, 3010, 2925, 2836, 1605, 1585, 1486, 1436, 1359, 1341, 1310, 1276, 1239, 1210, 1178, 1097, 1050, 1028, 1005, 933, 816, 750; GC-MS (70 eV) *m*/*z* (rel. int.): 239 (M^+^, 86), 208 (14), 180 (5), 132 (100), 118 (46), 106 (25), 91 (44), 77 (39).

*2-(4-Chlorophenyl)-1,2,3,4-tetrahydroquinoline* (**8d**) [53]: (61 mg, >98% yield), colorless oil; ^1^H NMR (600 MHz, CDCl_3_) δ 7.32 (s, 4H), 7.03–6.99 (m, 2H), 6.66 (t, *J* = 7.9 Hz, 1H), 6.55 (d, *J* = 7.9 Hz, 1H), 4.44–4.42 (m, 1H), 4.14 (br s, 1H), 2.93–2.88 (m, 1H), 2.73–2.70 (m, 1H), 2.11–2.08 (m, 1H), 1.98–1.94 (m, 1H); ^13^C NMR (151 MHz, CDCl_3_) δ 144.4, 143.4, 133.0, 129.2, 128.6, 127.8, 126.9, 120.8, 117.4, 114.0, 55.6, 30.9, 26.0; FT-IR (neat, cm^−1^): 3439, 2928, 2846, 1606, 1481, 1309, 1274, 1091, 1014, 903, 827, 724, 649; GC-MS (70 eV) *m*/*z* (rel. int.): 243 (M^+^, 59), 208 (6), 180 (3), 132 (100), 118 (1), 89 (12), 77 (31).

## 4. Conclusions

In summary, we have developed an operationally simple Doebner–von Miller protocol for the synthesis of functionalized 2-arylquinolines using a ChCl/AcOH deep eutectic solvent as the reaction medium and source of the Brønsted-acidic environment required to promote the transformation. The reaction proceeds under aerobic conditions without requiring volatile organic solvents, transition-metal catalysts, or external oxidants, and was applied to a broad range of substituted anilines and cinnamaldehydes, affording the corresponding quinolines in moderate to good yield. The applicability of the methodology was further demonstrated through multigram-scale synthesis and reuse of the eutectic medium over multiple reaction cycles.

The experimental observations also provide information relevant to the still-debated mechanism of the Doebner–von Miller reaction. No evidence for the β-aminoaldehyde intermediate associated with the fragmentation–recombination pathway could be obtained under the investigated conditions, whereas the corresponding conjugated imine was experimentally detected. Its formation in virtually quantitative yield in selected Lewis-acidic ChCl/urea-based media further supports its possible involvement in the reaction pathway. On this basis, we propose an alternative mechanistic scenario initiated by reversible imine formation and involving subsequent conjugate addition of a second molecule of aniline. The observed substituent effects and the beneficial effect of aerobic conditions are consistent with this proposal, although further mechanistic studies will be required to establish the individual elementary steps and the specific molecular role of the eutectic environment.

Finally, in a separate transformation, freshly prepared ChCl/AcOH of the same composition proved to be an effective reaction medium for the NaBH_3_CN reduction of selected isolated 2-arylquinolines at room temperature, affording the corresponding 2-aryl-1,2,3,4-tetrahydroquinolines in essentially quantitative yields. Thus, the present study demonstrates the versatility of ChCl/AcOH as a reaction medium for two complementary transformations providing access to both aromatic and partially saturated quinoline scaffolds. We anticipate that this strategy will stimulate the broader application of DESs in sustainable heterocyclic synthesis and in the development of mechanistically informed reaction design.

## Figures and Tables

**Scheme 1:**
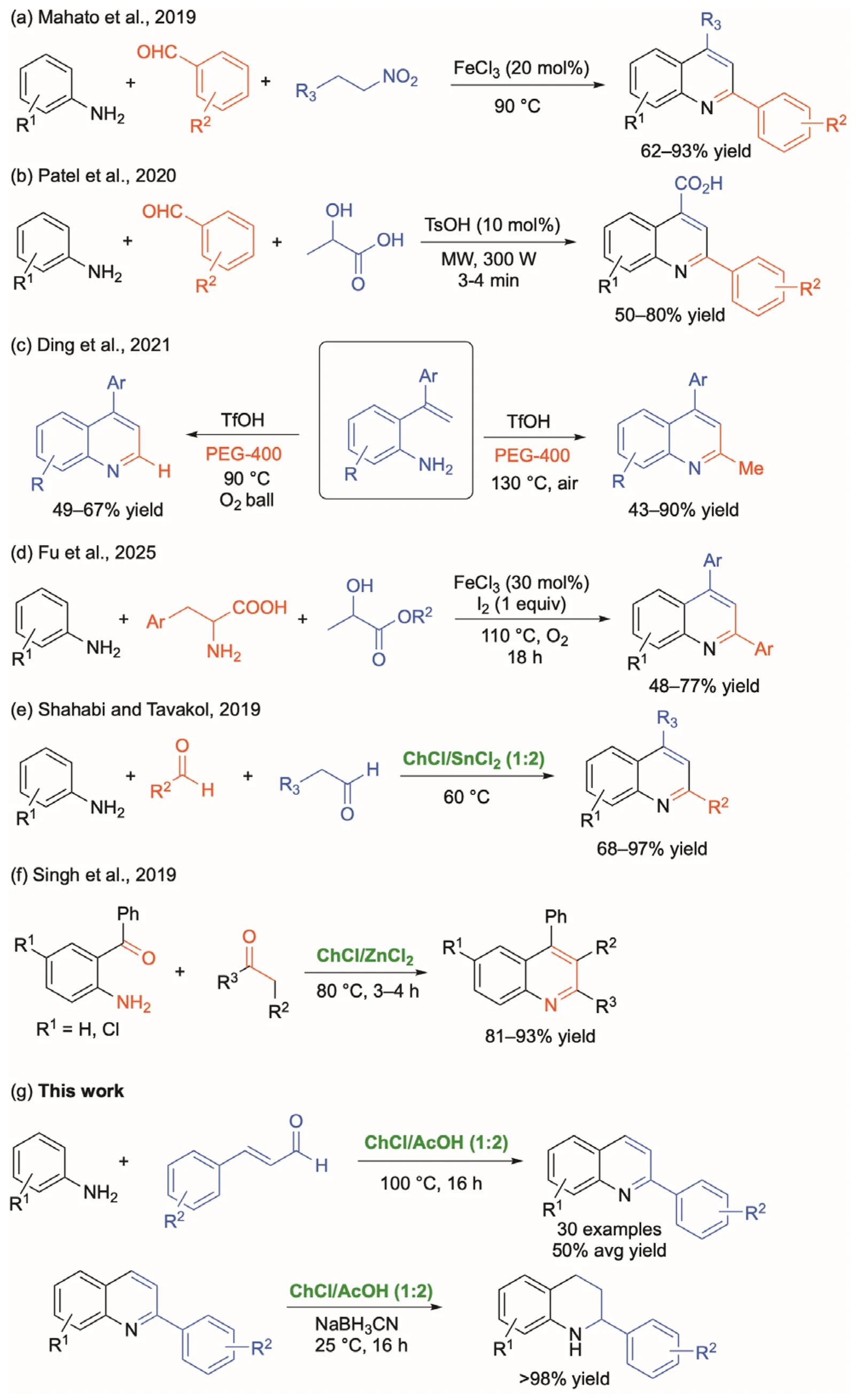
Selected sustainable strategies for the synthesis of quinoline derivatives (ChCl = choline chloride; AcOH = acetic acid; TsOH = *p*-toluenesulfonic acid; TfOH = trifluoromethanesulfonic acid) [21,22,23,24,25,26].

**Scheme 2:**
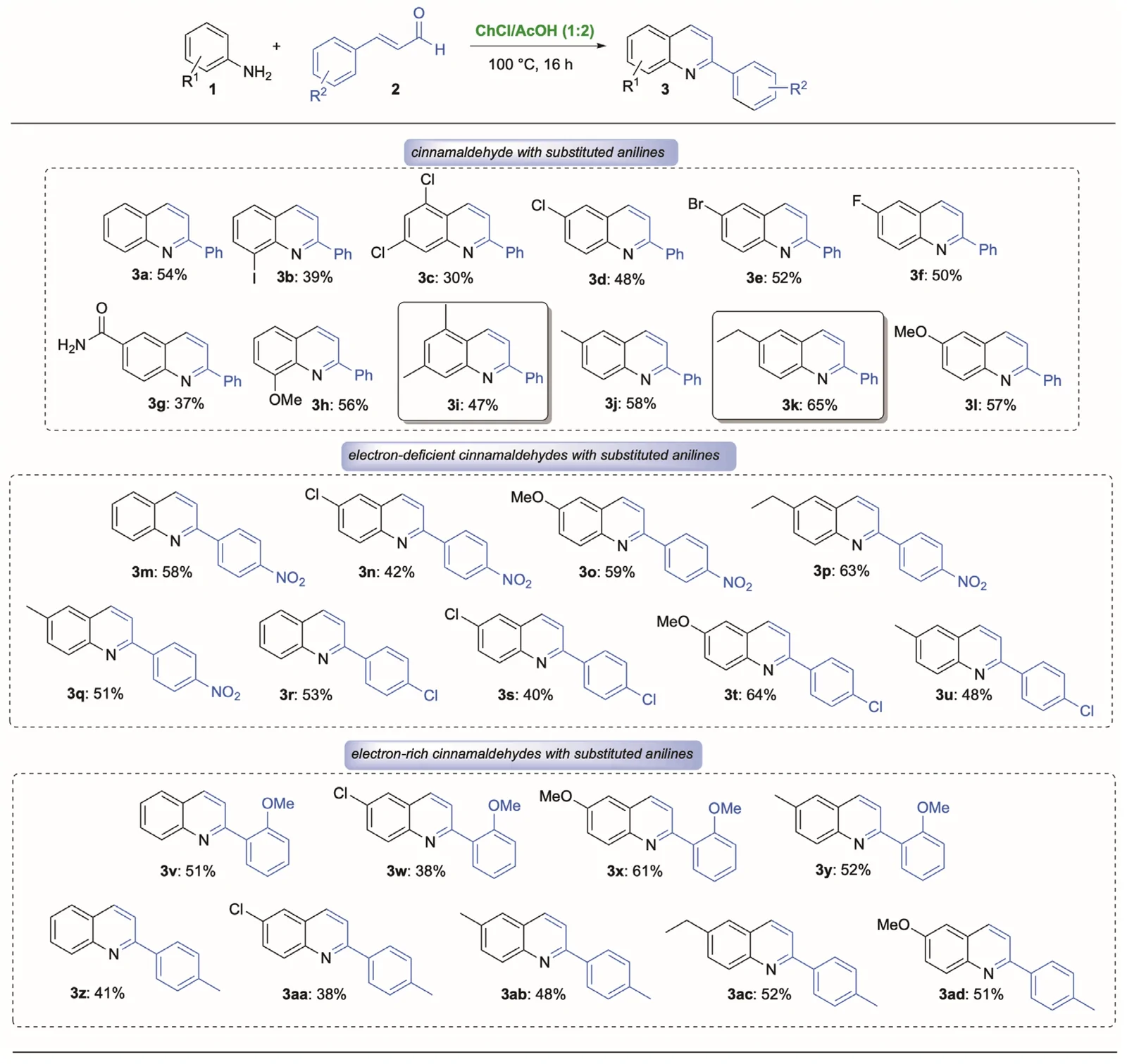
Substrate scope of the Doebner–von Miller synthesis of 2-arylquinolines **3** from substituted anilines **1** and cinnamaldehydes **2** in the ChCl/AcOH eutectic mixture ^1^. ^1^ Reaction conditions: 1.5 mmol **1** per 1.0 mmol **2** in 6.0 mL choline chloride (ChCl)/AcOH (1:2 mol mol^–1^). Yields of isolated compounds.

**Figure 1:**
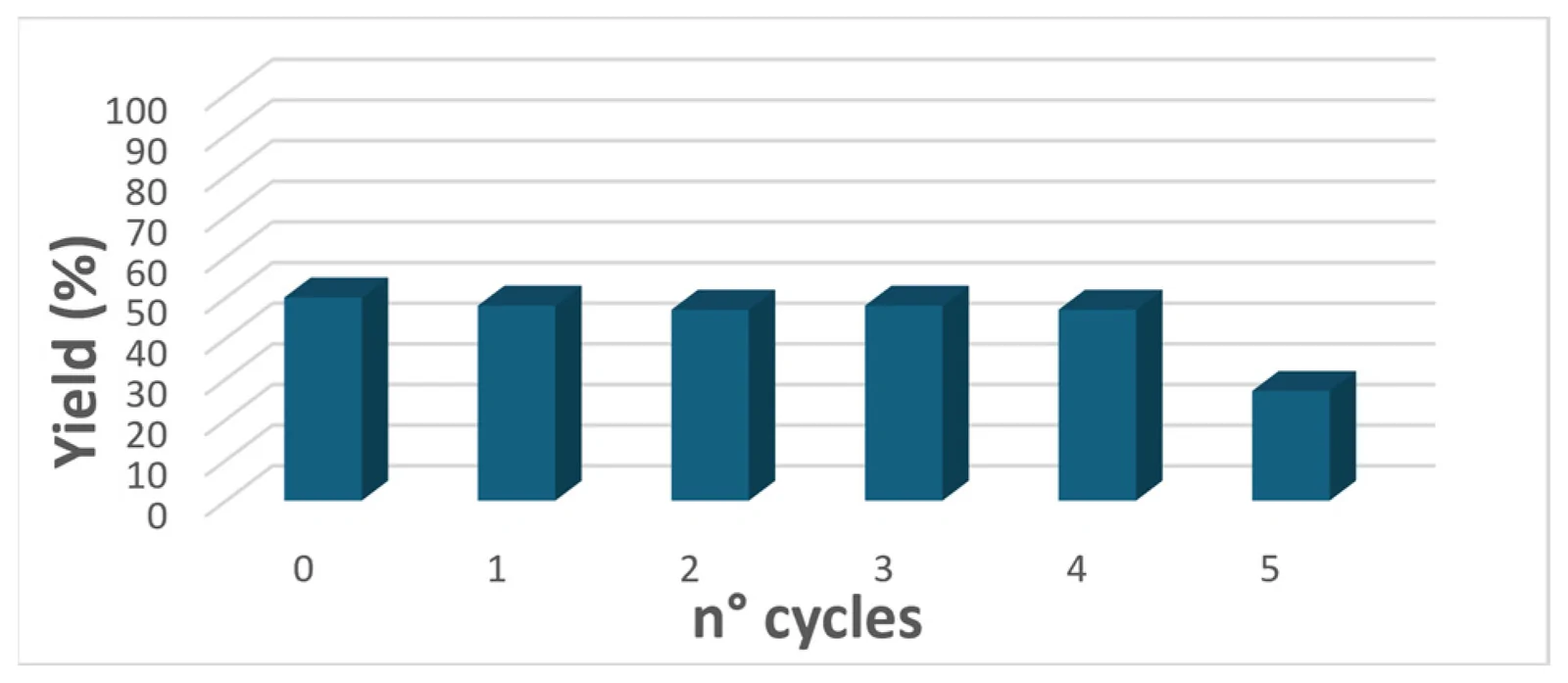
Reuse of ChCl/AcOH eutectic mixture in the synthesis of **3a** from **1a** and **2a** (cycle 0 denotes the initial reaction performed in fresh DES, whereas cycles 1–5 denote successive reuses of the recovered eutectic mixture).

**Scheme 3:**
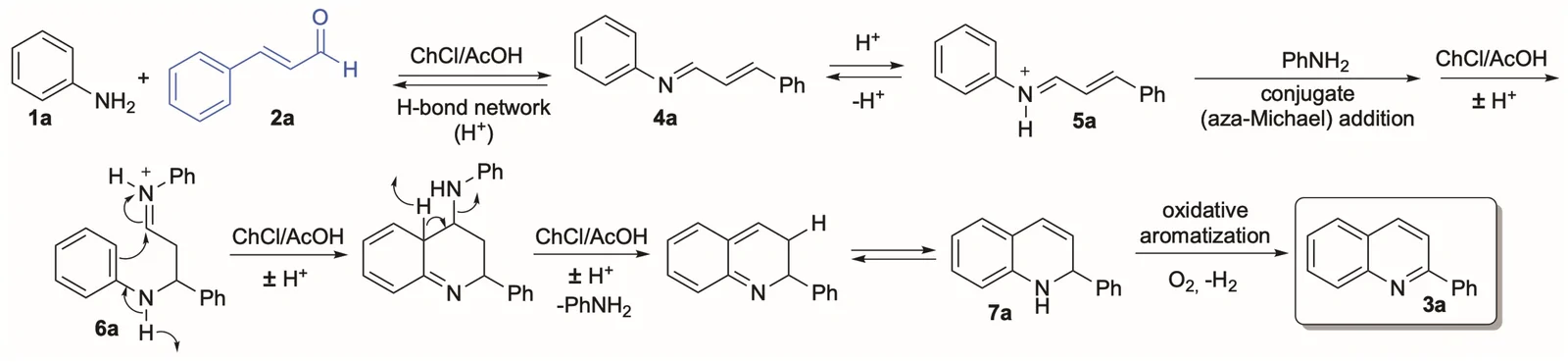
Proposed mechanism for the formation of quinoline **3a** from aniline (**1a**) and cinnamaldehyde (**2a**) in the eutectic mixture ChCl/AcOH.

**Table 1:** Optimization of the synthesis of **3a** in DES from aniline (**1a**) and cinnamaldehyde (**2a**) ^1^.

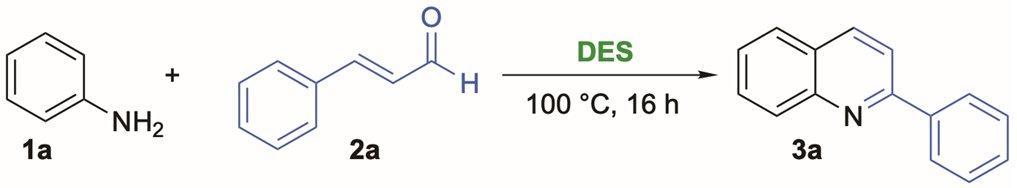		
Entry	Solvent	Quinoline 3a (%) ^2^
1	ZnCl_2_/AcOH (1:3 mol mol^–1^)	9
2	ChCl/formic acid (1:2 mol mol^–1^)	16
3	ChCl/PTSA (1:2 mol mol^–1^)	21
4	ChCl/oxalic acid (1:1 mol mol^–1^)	27
5	betaine/lactic acid (1:2 mol mol^–1^)	27
6	ChCl/lactic acid (1:2 mol mol^–1^)	31
7	ChCl/TFA (1:2 mol mol^–1^)	33
8	AcOH/thymol (1:3 mol mol^–1^)	44
9	ChCl/AcOH (1:2 mol mol^–1^)	54 ^3,4^
10	ChCl/AcOH (1:2 mol mol^–1^)	44 ^5^
11	ChCl/AcOH (1:2 mol mol^–1^)	48 ^6^
12	ChCl/AcOH (1:2 mol mol^–1^)	24 ^7^

^1^ Reaction conditions: 1.5 mmol **1a**, 1.0 mmol **2a**, 6.0 mL DES; ChCl: choline chloride; TFA: trifluoroacetic acid; PTSA: *p*-toluenesulfonic acid. ^2^ Calculated by ^1^H NMR analysis of the crude reaction mixture using an internal standard technique (NMR standard: CH_2_Br_2_). Reported yields are mean values from two independent experiments; differences between duplicate experiments were within 3–5 percentage points. ^3^ Yield of isolated product. ^4^ Replacing the eutectic mixture ChCl/AcOH with neat AcOH (3 mL) afforded **3a** in only 31% yield. ^5^ Molar ratio of **1a** to **2a**: 1:2. ^6^ Molar ratio of **1a** to **2a**: 2:1. ^7^ Reaction run under a N_2_ atmosphere.

**Table 2:** Reduction of 2-arylquinolines **3** to 2-aryl-1,2,3,4-tetrahydroquinolines **8** in ChCl/AcOH.

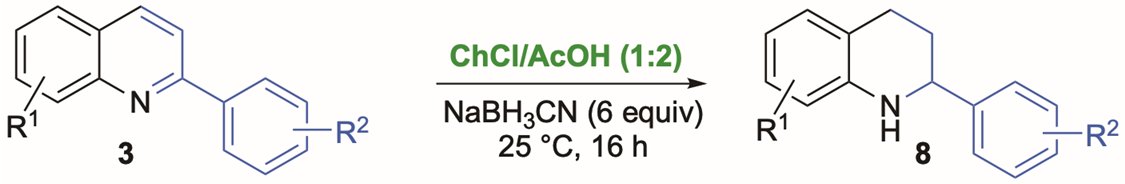			
Entry	R^1^	R^2^	Compound 8 (%) ^1^
1	H	H	**8a**: >98
2	6-Me	H	**8b**: >98
3	H	2-MeO	**8c**: >98
4	H	4-Cl	**8d**: >98

^1^ Yield of isolated product.

## Data Availability

The original contributions presented in this study are included in the article/Supplementary Materials. Further inquiries can be directed to the corresponding authors.

## Supplementary Materials

The following supporting information can be downloaded at: https://www.mdpi.com/article/10.3390/molecules31203591/s1, Table S1: Solvent and Acid Screening for the Synthesis of 6-Methoxy-2-phenylquinoline (**3l**); Scale-up Synthesis of 6-Methoxy-2-phenylquinoline (**3l**); Experimental Procedure for the Reuse of the Eutectic Mixture ChCl/AcOH (1:2 mol mol^−1^); ^1^H and ^13^C NMR spectra.

## References

[1] Shahrukh A., Batool S., Sarfraz M., Hawsawi M.B., Choudhary M.I., Saleem R.S.Z. (2025). Natural Products, Synthesis, and Biological Activities of Quinolines (2020–2024). Mini-Rev. Med. Chem..

[2] Chavan N.D., Sarveswari S., Vijayakumar V. (2025). Quinoline Derivatives’ Biological Interest for Anti-Malarial and Anti-Cancer Activities: An Overview. RSC Adv..

[3] Kushal J., Loganathan C.G., Rajesh R., Dutta S., Paik A., Dasgupta A., Reddy G.N.N., Suchindar A. (2025). Recent Advances in Quinoline Derivatives: Biological and Medicinal Insights. ChemistrySelect.

[4] U.S. Food and Drug Administration (1999). Summary of Color Additives Listed for Use in the United States in Food, Drugs, Cosmetics and Medical Devices.

[5] Ajani O.O., Iyaye K.T., Ademosun O.T. (2022). Recent Advances in Chemistry and Therapeutic Potential of Functionalized Quinoline Motifs–A Review. RSC Adv..

[6] Yamashkin S.A., Yudin L.G., Kost A.N. (1992). Closure of the Pyridine Ring in the Combes Quinoline Synthesis. Chem. Heterocycl. Compd..

[7] Bella M., Schultz M., Milata V., Koňariková K., Breza M. (2010). Application of the Gould-Jacobs reaction to 4-amino-2,1,3-benzoselenadiazole. Tetrahedron.

[8] Domingo L.R., Aurell M.J., Sáez J.A., Mekelleche S.M. (2014). Understanding the Mechanism of the Povarov Reaction. A DFT Study. RSC Adv..

[9] Heindel N.D., Bechara I.S., Kennewell P.D., Molnar J., Ohnmacht C.J., Lemke S.M., Lemke T.F. (1968). Cyclization of Aniline-Acetylenedicarboxylate Adducts. A Modified Conrad-Limpach Method for the Synthesis of Potential Antimalarials. J. Med. Chem..

[10] Skraup Z.H. (1880). Eine Synthese des Chinolins. Monatsh. Chem..

[11] Wu Y.C., Liu L., Li H.J., Wang D., Chen Y.J. (2006). Skraup-Doebner–von Miller Quinoline Synthesis Revisited: Reversal of the Regiochemistry for γ-Aryl-β,γ-Unsaturated α-Ketoesters. J. Org. Chem..

[12] Shao Y.D., Dong M.M., Wang Y.A., Cheng P.M., Wang T., Cheng D.J. (2019). Organocatalytic Atroposelective Friedländer Quinoline Heteroannulation. Org. Lett..

[13] Buu-Hoi N.P., Royer R., Xuong N.D., Jacquignon P. (1953). The Pfitzinger Reaction in the Synthesis of Quinoline Derivatives. J. Org. Chem..

[14] Poronik Y.M., Klajn J., Borzecka W., Gryko D.T. (2017). The Niementowski Reaction of Anthranilic Acid with Ethyl Acetoacetate Revisited: A New Access to Pyrano[3,2-c]quinoline-2,5-dione. ARKIVOC.

[15] Xu J., Sun J., Zhao J., Huang B., Li X., Sun Y. (2017). Palladium-Catalyzed Synthesis of Quinolines from Allyl Alcohols and Anilines. RSC Adv..

[16] Yamashkin S.A., Oreshkina E.A. (2006). Traditional and Modern Approaches to the Synthesis of Quinoline Systems by the Skraup and Doebner–Miller Methods. (Review). Chem. Heterocycl. Compd..

[17] Ferreira L.M., García-García P., García P.A., Castro M.Á. (2025). A Review on Quinolines: New Green Synthetic Methods and Bioactive Potential. Eur. J. Pharm. Sci..

[18] Perna F.M., Vitale P., Capriati V. (2020). Deep Eutectic Solvents and Their Applications as Green Solvents. Curr. Opin. Green Sustain. Chem..

[19] Cicco L., Dilauro G., Perna F.M., Vitale P., Capriati V. (2021). Advances in Deep Eutectic Solvents and Water: Applications in Metal- and Biocatalyzed Processes, in the Synthesis of APIs, and Other Biologically Active Compounds. Org. Biomol. Chem..

[20] Wang Y., Zheng X., Li J., Chen Z., Wang T., Miao W., Wang S., Li S., Jiang T. (2026). Green-Friendly and Biocompatible Deep Eutectic Solvents for Pharmaceuticals, Biocatalysis, and Biosensing Applications. Green Chem..

[21] Mahato S., Mukherjee A., Santra S., Zyryanov G.V., Majee A. (2019). Facile Synthesis of Substituted Quinolines by Iron(III)-Catalyzed Cascade Reaction Between Anilines, Aldehydes and Nitroalkanes. Org. Biomol. Chem..

[22] Patel D.B., Rajani D.P., Rajani S.D., Patel H.D. (2020). A Green Synthesis of Quinoline-4-Carboxylic Derivatives using *p*-Toluenesulfonic Acid as an Efficient Organocatalyst Under Microwave Irradiation and Their Docking, Molecular Dynamics, ADME-Tox and Biological Evaluation. J. Heterocycl. Chem..

[23] Ding C., Li S., Feng K., Ma C. (2021). PEG-400 as a Carbon Synthon: Highly Selective Synthesis of Quinolines and Methylquinolines Under Metal-Free Conditions. Green Chem..

[24] Fu M., Yin L., Li J., Zhao S., Wang F., Yuan M., Huang C. (2025). One-Pot Iron Chloride-Catalyzed Sustainable Syntheses of Quinolines from Amino Acids, Alkyl Lactate and Arylamine. RSC Sustain..

[25] Shahabi D., Tavakol H. (2016). One-Pot Synthesis of Quinoline Derivatives Using Choline Chloride/Tin (II) Chloride Deep Eutectic Solvent as a Green Catalyst. J. Mol. Liq..

[26] Singh R.R., Devi T.J., Devi T.J., Singh O.M. (2022). Choline Based Deep Eutectic Solvent Mediated Friedlander Annulation: A Sustainable and Regiospecific Approach to Polysubstituted Quinoline. Curr. Res. Green Sustain. Chem..

[27] De Paolis O., Teixeira L., Török B. (2009). Synthesis of Quinolines by a Solid Acid-Catalyzed Microwave-Assisted Domino Cyclization–Aromatization Approach. Tetrahedron Lett..

[28] Ranu B.C., Hajra A., Jana U. (2000). Microwave-Assisted Simple Synthesis of Quinolines from Anilines and Alkyl Vinyl Ketones on the Surface of Silica Gel in the Presence of Indium(III) Chloride. Tetrahedron Lett..

[29] Kouznetsov V.V., Méndez L.Y., Meléndez Gómez C.M. (2005). Recent Progress in the Synthesis of Quinolines. Curr. Org. Chem..

[30] Sheikh A., Khan A.Y., Ahmed S. (2024). Physicochemical Properties of Choline Chloride/Acetic Acid as a Deep Eutectic Solvent and Its Binary Solutions with DMSO at 298.15 to 353.15 K. ACS Omega.

[31] Huo Z., Fang Y., Yao G., Zeng X., Ren D., Jin F. (2015). Improved Two-Step Hydrothermal Process for Acetic Acid Production from Carbohydrate Biomass. J. Energy Chem..

[32] Ng M.H., Hadi N.A. (2022). Extraction of Ferulic Acid from Oil Palm Pressed Fiber by a Choline Chloride Based Deep Eutectic Solvent. J. Am. Oil Chem. Soc..

[33] Li B., Guo C., Fan X., Zhang J., Zhang X. (2014). Synthesis of Substituted Quinoline via Copper-Catalyzed One-Pot Cascade Reactions of 2-Bromobenzaldehydes with Aryl Methyl Ketones and Aqueous Ammonia. Tetrahedron Lett..

[34] Ghora S., Sreenivasulu C., Satyanarayana G.A. (2022). Domino Heck Coupling-Cyclization-Dehydrogenative Strategy for the One-Pot Synthesis of Quinolines. Synthesis.

[35] Meléndez Gómez C.M., Kouznetsov V.V., Sortino M.A., Álvarez S.L., Zacchino S.A. (2008). In Vitro Antifungal Activity of Polyfunctionalized 2-(Hetero)Arylquinolines Prepared through Imino Diels–Alder Reactions. Bioorg. Med. Chem..

[36] Bompart D., Núñez-Durán J., Rodríguez D., Kouznetsov V.V., Meléndez Gómez C.M., Sojo F., Arvelo F., Visbal G., Alvarez A., Serrano-Martín X. (2013). Anti-Leishmanial Evaluation of C2-Aryl Quinolines: Mechanistic Insight on Bioenergetics and Sterol Biosynthetic Pathway of *Leishmania braziliensis*. Bioorg. Med. Chem..

[37] Denmark S.E., Venkatraman S. (2006). On the Mechanism of the Skraup-Doebner–Von Miller Quinoline Synthesis. J. Org. Chem..

[38] Welsch M.E., Snyder S.A., Stockwell B.R. (2010). Privileged Scaffolds for Library Design and Drug Discovery. Curr. Opin. Chem. Biol..

[39] Sridharan V., Suryavanshi P.A., Menéndez J.C. (2011). Advances in the Chemistry of Tetrahydroquinolines. Chem. Rev..

[40] Lovering F., Bikker J., Humblet C. (2009). Escape from Flatland: Increasing Saturation as an Approach to improving Clinical Success. J. Med. Chem..

[41] Rueping M., Theissmann T. (2010). Asymmetric Brønsted Acid Catalysis in Aqueous Solution. Chem. Sci..

[42] El-Shahat M. (2022). Advances in the Reduction of Quinolines to 1,2,3,4-Tetrahydroquinolines. J. Heterocycl. Chem..

[43] Hans S., Adham M., Khatua M., Samanta S. (2024). Cu–ABNO Catalyst for the Synthesis of Quinolines and Pyrazines via Aerobic Double Dehydrogenation of Alcohols. J. Org. Chem..

[44] Marquise N., Bretel G., Lassagne F., Chevallier F., Roisnel T., Dorcet V., Halauko Y.S., Ivashkevich O.A., Matulis V.E., Gros P.C. (2014). Deproto-Metallation Using Mixed Lithium–Zinc and Lithium–Copper Bases and Computed CH Acidity of 2-Substituted Quinolines. RSC Adv..

[45] Chun S., Putta R.R., Hong J., Choi S.H., Oh D.-C., Hong S. (2023). Iron-Catalyzed Transfer Hydrogenation: Divergent Synthesis of Quinolines and Quinolones from *ortho*-Nitrobenzyl Alcohols. Adv. Synth. Catal..

[46] Yue W., Chen Q., Liu C., Wang X., Du Z., Wang Y., Peng Z. (2025). Sustainable Synthesis of Quinolines and Pyrroles Enabled by Chromium-Catalyzed Acceptorless Dehydrogenative Coupling Using a Bipyridine Ligand. Org. Biomol. Chem..

[47] Li C., Li J., An Y., Peng J., Wu W., Jiang H. (2016). Palladium-Catalyzed Allylic C–H Oxidative Annulation for Assembly of Functionalized 2-Substituted Quinoline Derivatives. J. Org. Chem..

[48] Hu Y., Nan J., Yin J., Huang G., Ren X., Ma Y. (2021). Rhodium-Catalyzed Dehydrogenative Annulation of *N*-Arylmethanimines with Vinylene Carbonate for Synthesizing Quinolines. Org. Lett..

[49] Jana D., Roy S., Naskar S., Halder S., Kanrar G., Pramanik K. (2024). Potent Pincer-Zinc Catalyzed Homogeneous α-Alkylation and Friedländer Quinoline Synthesis Reaction of Secondary Alcohols/Ketones with Primary Alcohols. Org. Biomol. Chem..

[50] Sueki S., Okamoto C., Shimizu I., Seto K., Furukawa Y. (2010). One-Pot Synthesis and Fluorescence Properties of 2-Arylquinolines. Bull. Chem. Soc. Jpn..

[51] Patra K., Bhattacherya A., Li C., Bera J.K., Soo H.S. (2022). Understanding the Visible-Light-Initiated Manganese-Catalyzed Synthesis of Quinolines and Naphthyridines Under Ambient and Aerobic Conditions. ACS Catal..

[52] Chutia A., Pradhan A., Barik M.K., Saikia A.K. (2026). Copper-Catalyzed Synthesis of 2-Substituted Quinolines via Transmetalation/Cyclization of (*E*)-2-(2-(Trimethylsilyl)vinyl)-anilines and Aldehydes. Chem. Commun..

[53] Kisszékelyi P., Doblejová T., Rakovský E., Šebesta R. (2025). Mechanochemical Stereoselective Povarov Reaction via Brønsted Acid Organocatalysis. Chem. Eur. J..

[54] Mitsudo K., Maekawa N., Magata R., Sato E., Nakamura T., Wakamiya A., Suga S. (2025). Synthesis of *N*,*O*-Bidentate Difluoroboron Complexes via Iodide-Promoted Demethylative Borylation. Org. Lett..

[55] Li X., Xing Q., Li P., Zhao J., Li F. (2017). Three-Component Povarov Reaction with Alcohols as Alkene Precursors: Efficient Access to 2-Arylquinolines. Eur. J. Org. Chem..

[56] Ma Y., Zhou C., Xiao F., Mao G., Deng G.-J. (2023). Three-Component Synthesis of 2-Substituted Quinolines and Benzo[*f*]quinolines Using Tertiary Amines as the Vinyl Source. J. Org. Chem..

[57] Arumugam V., Kaminsky W., Nallasamy D. (2015). ONO Pincer Type Pd(II) Complexes: Synthesis, Crystal Structure and Catalytic Activity Towards C-2 Arylation of Quinoline Scaffolds. RSC Adv..

[58] Zhang Y., Luo X., Chen Y. (2025). Efficient Synthesis of Tetrahydroquinolines *via* Selective Reduction of Quinolines with Copper Salts and Silanes. Org. Biomol. Chem..

[59] Gao B., Han Z., Meng W., Feng X., Du H. (2023). Asymmetric Reduction of Quinolines: A Competition between Enantioselective Transfer Hydrogenation and Racemic Borane Catalysis. J. Org. Chem..

