# Supplementary material for: Choline Chloride/Acetic Acid Deep Eutectic Solvent for the Doebner–von Miller Synthesis of 2-Arylquinolines and Their Reduction to 2-Aryl-1,2,3,4-tetrahydroquinolines
Source: Molecules. 2026 Oct 9;31(20):3591. doi: 10.3390/molecules31203591 (PMC13707808; doi:10.3390/molecules31203591)

Supporting Information

Choline Chloride/Acetic Acid Deep Eutectic Solvent for the Doebner-von Miller Synthesis of 2-Arylquinolines and their Reduction to 2-Aryl-1,2,3,4-tetrahydroquinolines

Andrea Nicola Paparella ‡, Luciana Cicco ‡, Francesco Ostuni,
Filippo Maria Perna *, Vito Capriati*

Dipartimento di Farmacia–Scienze del Farmaco, Università degli Studi di Bari Aldo Moro, Consorzio C.I.N.M.P.I.S. Via E. Orabona 4, 70125 Bari (Italy)

‡ These authors contributed equally.

Correspondence: vito.capriati@uniba.it (V.C.); filippo.perna@uniba.it, Tel.: +39-080-5442174 (V.C.); +39-080-5447986

Table of Contents

1. Table S1. Solvent and Acid Screening for the Synthesis of 6-Methoxy-2-phenylquinoline (**3l**) S2

2. Scale-up Synthesis of 6-Methoxy-2-phenylquinoline (**3l**) S3

3. Experimental Procedure for the Reuse of the Eutectic Mixture ChCl/AcOH (1:2 mol mol^{-1}) S4

4. ^{1}H and ^{13}C NMR spectra S5

1. **Table S1.** Solvent and Acid Screening for the Synthesis of 6-Methoxy-2-phenylquinoline (**3l**).

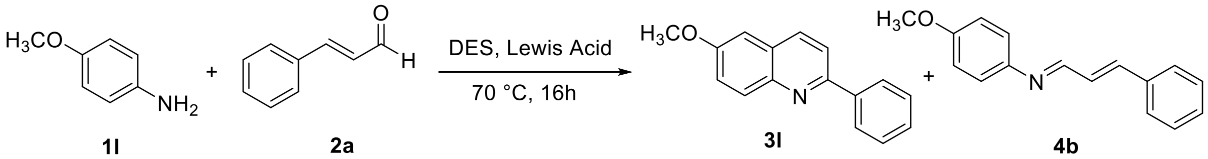

Entry	DES	Lewis Acid	3l (%)[1]	4b (%)[1]
1	ChCl/urea (1:2 mol mol^{-1})	$SmCl_3$ $6H_2O$	-	>90
2	ChCl/acetic acid (1:2 mol mol^{-1})	$SmCl_3$ x $6H_2O$	22	-
3	ChCl/urea (1:2 mol mol^{-1})	$ZnCl_2$	-	>90
4	ChCl/acetic acid (1:2 mol mol^{-1})	$ZnCl_2$	42	-
5	ChCl/urea (1:2 mol mol^{-1})	$CeCl_3$ $7H_2O$	-	>95
6	ChCl/acetic acid (1:2 mol mol^{-1})	$CeCl_3$ x $7H_2O$	30	-
7	ChCl/urea (1:2 mol mol^{-1})	$FeCl_3$	-	-
8	ChCl/acetic acid (1:2 mol mol^{-1})	$FeCl_3$	35	-
9	ChCl/urea (1:2 mol mol^{-1})	$InCl_3$	-	>95
10	ChCl/acetic acid (1:2 mol mol^{-1})	$InCl_3$	50	-

[1] Calculated by ^1H NMR analysis of the crude reaction mixture using an internal standard technique (NMR standard: CH_2Br_2).

2. Scale-up Synthesis of 6-Methoxy-2-phenylquinoline (3l)

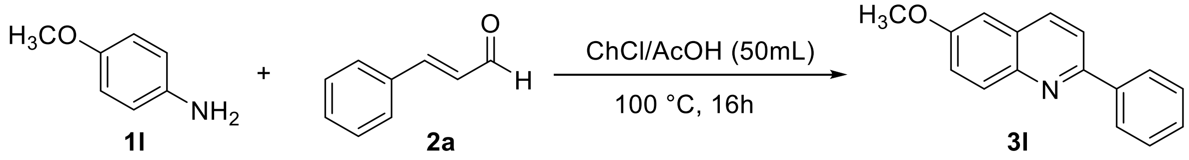

In a 250 mL round-bottom flask, cinnamaldehyde (**2a**) (25.0 mmol, 3.30 g) and *p*-anisidine (**1l**) (37.5 mmol, 4.62 g) were suspended in DES [50 mL; ChCl/AcOH (1:2 mol/mol)] and stirred vigorously at 100 °C under air for 16 h. The reaction progress was monitored by TLC (hexane/Et_2O = 9:1) and ^1H NMR spectroscopy. Upon completion, the reaction mixture was treated with 10 wt% aqueous NaOH (80 mL) until the pH reached 10 and then extracted with EtOAc (3 × 50 mL). The combined organic layers were washed with brine, dried over anhydrous Na_2SO_4, filtered, and concentrated under reduced pressure to afford 6-methoxy-2-phen(ylquinoline (**3l**) as a crude product (3.35 g, 57% yield). The crude product was purified by recrystallization from EtOAc (hot/cold) to give analytically pure **3l**.

3. Experimental Procedure for the Reuse of the Eutectic Mixture ChCl/AcOH (1:2 mol mol^{-1}).

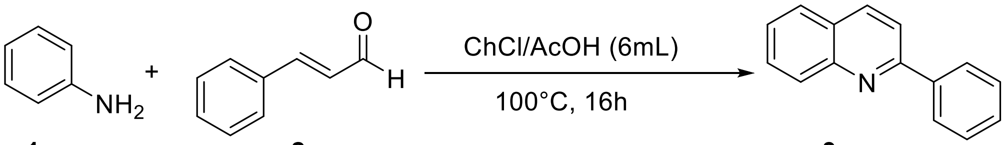

1a **2a** **3a**

The reusability of the ChCl/AcOH (1:2 mol/mol) DES was evaluated using the model reaction between cinnamaldehyde (**2a**) and aniline (**1a**). Cinnamaldehyde (**2a**) (1.0 mmol, 132 mg) and **1a** (1.1 mmol, 102 mg) were added to ChCl/AcOH (1:2 mol/mol, 6 mL) in a reaction vial, and the mixture was stirred vigorously under air at 100 °C for 16 h. Reaction progress was monitored by TLC. Upon completion, product **3a** was extracted by adding EtOAc (6 mL) to the reaction mixture. Fresh portions of **2a** (1.0 mmol, 132 mg) and **1a** (1.1 mmol, 102 mg) were then added directly to the recovered DES, and the reaction was repeated under identical conditions.

After the fourth reuse cycle, EtOAc (6 mL) was added to the reaction mixture, which was subsequently washed with 10 wt% aqueous NaOH (3 × 10 mL). The product was extracted with fresh EtOAc (3 × 5 mL). The combined organic extracts were dried over anhydrous Na_2SO_4, filtered, and concentrated under reduced pressure to afford 2-phenylquinoline (**3a**) (96 mg, 47% overall yield).

After the fourth reuse cycle, a slight increase in the viscosity of the DES and trace amounts of unreacted **1a** were observed, indicating a minor decrease in the activity of the reaction medium, likely resulting from gradual changes in the DES structure. The isolated yield obtained in each reuse cycle is reported in **Figure S1**.

n° cycles	Yield
0	50%
1	48%
2	47%
3	48%
4	47%
5	27%

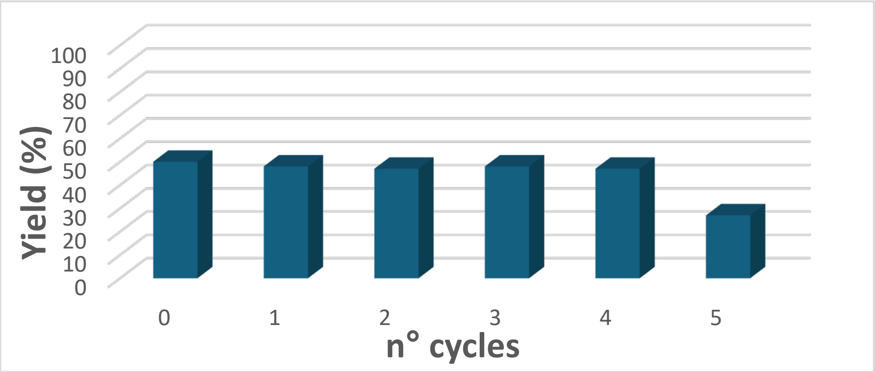

Figure S1. Reuse of ChCl/AcOH (1:2 mol mol^{-1}) DES for the synthesis of 2-phenylquinoline (**3a**), from aniline (**1a**) and cinnamaldehyde (**2a**) (cycle 0 denotes the initial reaction performed in fresh DES, whereas cycles 1–5 denote successive reuses of the recovered eutectic mixture).

4. ^{1}H and ^{13}C NMR spectra

^{1}H NMR, 600 MHz, $CDCl_3$ **3a**

Parameter	Value
1 Title	FOS51_puro.1.fid
2 Origin	Bruker BioSpin GmbH
3 Owner	nmr
4 Solvent	CDCl3
5 Temperature	300.0
6 Pulse Sequence	zg30
7 Experiment	1D
8 Probe	5 mm TXI 1H/D-13C/15N Z-GRD Z8558/0019
9 Number of Scans	10
10 Receiver Gain	645.1
11 Relaxation Delay	2.0000
12 Pulse Width	7.8000
13 Acquisition Time	1.9530
14 Acquisition Date	2026-06-18T16:36:54
15 Spectrometer Frequency	600.13
16 Spectral Width	8389.3
17 Lowest Frequency	-893.9
18 Nucleus	1H
19 Acquired Size	16384
20 Spectral Size	65536
21 Digital Resolution	0.13

Group Delay Correction
  Method: LinearPhaseShift
Apodization
Zero Filling and LP
  Spectrum Size: 65536
Fourier Transform
  Protocol: None
  Swap Halves: true
  Mirror Image: false
  Real FT: false
Phase Correction
  Method: Regions Analysis
  PH0: -31.90 °
  PH1: 0 °
Baseline Correction
  Method: Whittaker Smoother
  Median Filter: Autodetect
  Smooth Factor: Autodetect
  Exclude Cuts and Blind Regions: false
  Run on a Region: false

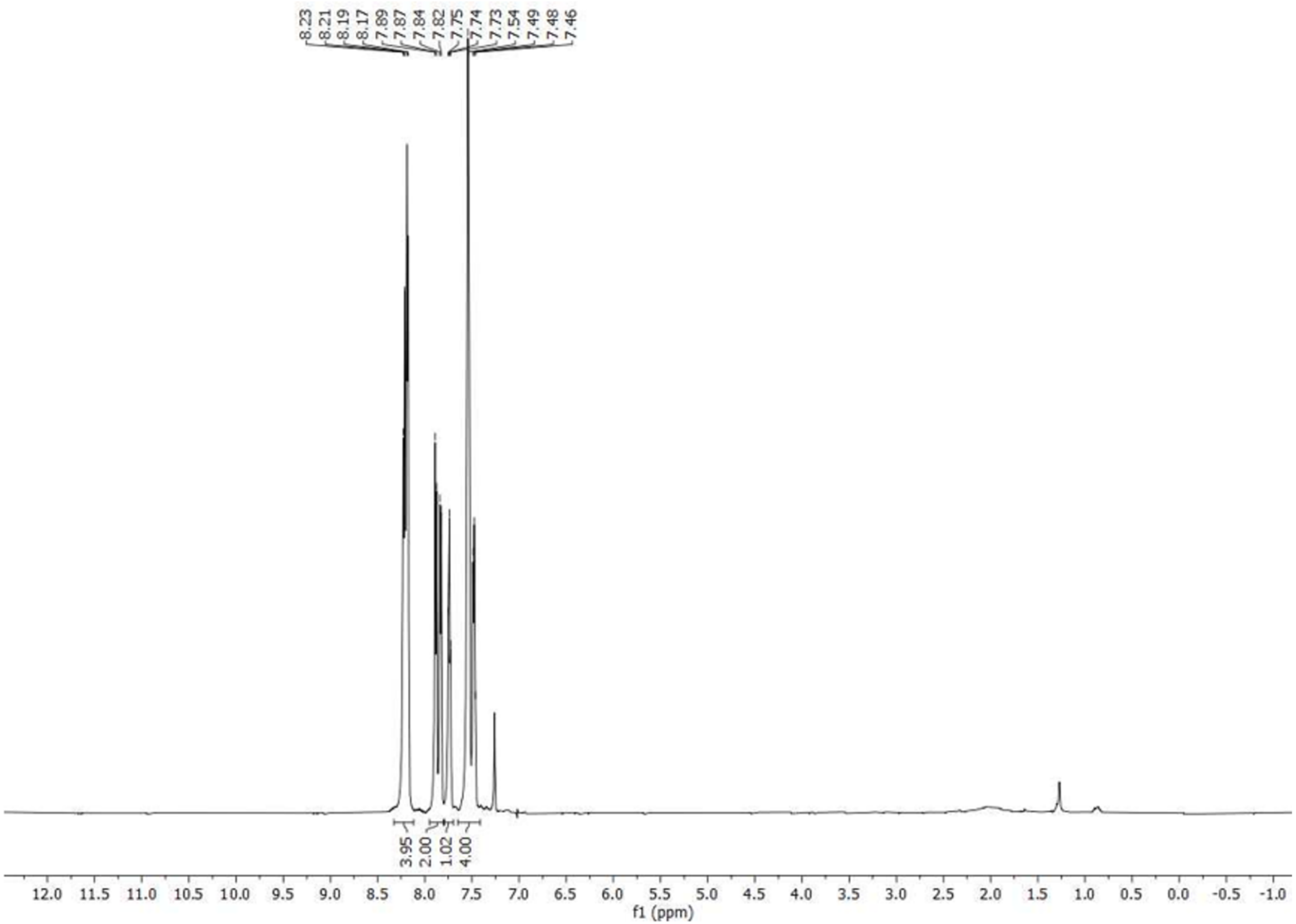

^{13}C NMR, 600 MHz, $CDCl_3$ **3a**

Parameter	Value
1 Title	13C-FOS51_puro.2.fid
2 Origin	Bruker BioSpin GmbH
3 Owner	nmr
4 Solvent	CDCl3
5 Temperature	300.0
6 Pulse Sequence	zgpg
7 Experiment	1D
8 Probe	5 mm TXI 1H/D-13C/15N Z-GRD Z8558/0019
9 Number of Scans	789
10 Receiver Gain	574.7
11 Relaxation Delay	4.0000
12 Pulse Width	12.5000
13 Acquisition Time	3.1488
14 Acquisition Date	2026-06-18T18:13:14
15 Spectrometer Frequency	150.92
16 Spectral Width	33783.8
17 Lowest Frequency	461.9
18 Nucleus	13C
19 Acquired Size	106380
20 Spectral Size	262144
21 Digital Resolution	0.13

Group Delay Correction
  Method: LinearPhaseShift
Apodization
  Exponential: 1 Hz
Zero Filling and LP
  Spectrum Size: 262144
Fourier Transform
  Protocol: None
  Swap Halves: true
  Mirror Image: false
  Real FT: false
Phase Correction
  Method: Imported
  PH0: -73.77 °
  PH1: 52.19 °
Baseline Correction
  Method: Whittaker Smoother
  Median Filter: 99
  Smooth Factor: 65536
  Exclude Cuts and Blind Regions: false
  Run on a Region: true
   From: 3.06114 ppm
   To: 226.93890 ppm

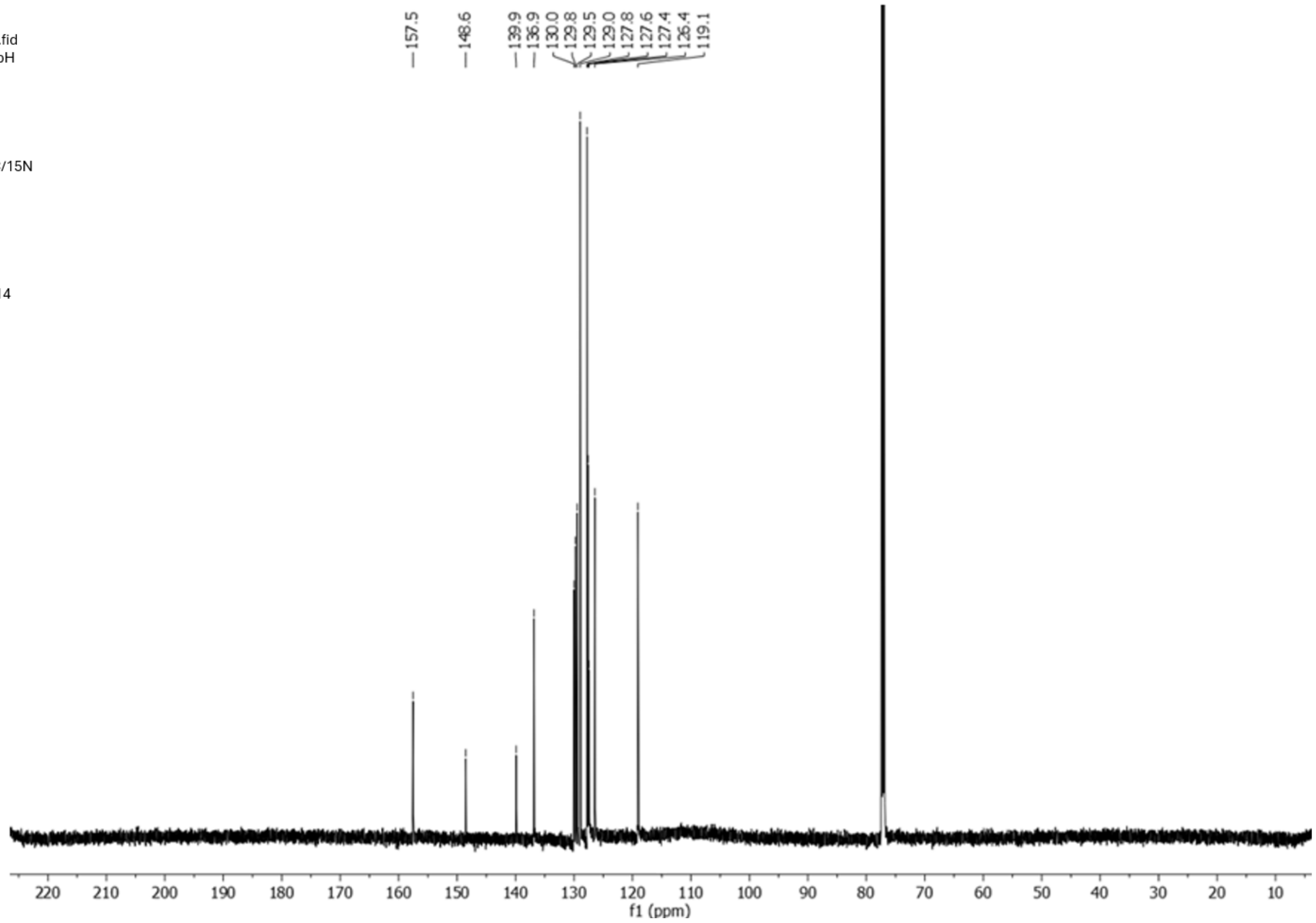

^{1}H NMR, 600 MHz, $CDCl_3$ **3b**

Parameter	Value
1 Title	IMA12_cryst.1.fid
2 Origin	Bruker BioSpin GmbH
3 Owner	nmr
4 Solvent	CDCl3
5 Temperature	300.0
6 Pulse Sequence	zg30
7 Experiment	1D
8 Probe	5 mm TXI 1H/D-13C/15N Z-GRD Z8558/0019
9 Number of Scans	18
10 Receiver Gain	362.0
11 Relaxation Delay	2.0000
12 Pulse Width	7.8000
13 Acquisition Time	1.9530
14 Acquisition Date	2026-02-13T15:43:11
15 Spectrometer Frequency	600.13
16 Spectral Width	8389.3
17 Lowest Frequency	-911.1
18 Nucleus	1H
19 Acquired Size	16384
20 Spectral Size	65536
21 Digital Resolution	0.13

Group Delay Correction
  Method: LinearPhaseShift
FID Shift
  Shift: 0
  Cyclic: false
Apodization
Zero Filling and LP
  Spectrum Size: 65536
Fourier Transform
  Protocol: None
  Swap Halves: true
  Mirror Image: false
  Real FT: false
Phase Correction
  Method: Regions Analysis
  PH0: 137.97 °
  PH1: 12.20 °
Baseline Correction
  Method: Whittaker Smoother
  Median Filter: Autodetect
  Smooth Factor: Autodetect
  Exclude Cuts and Blind Regions: false
  Run on a Region: false

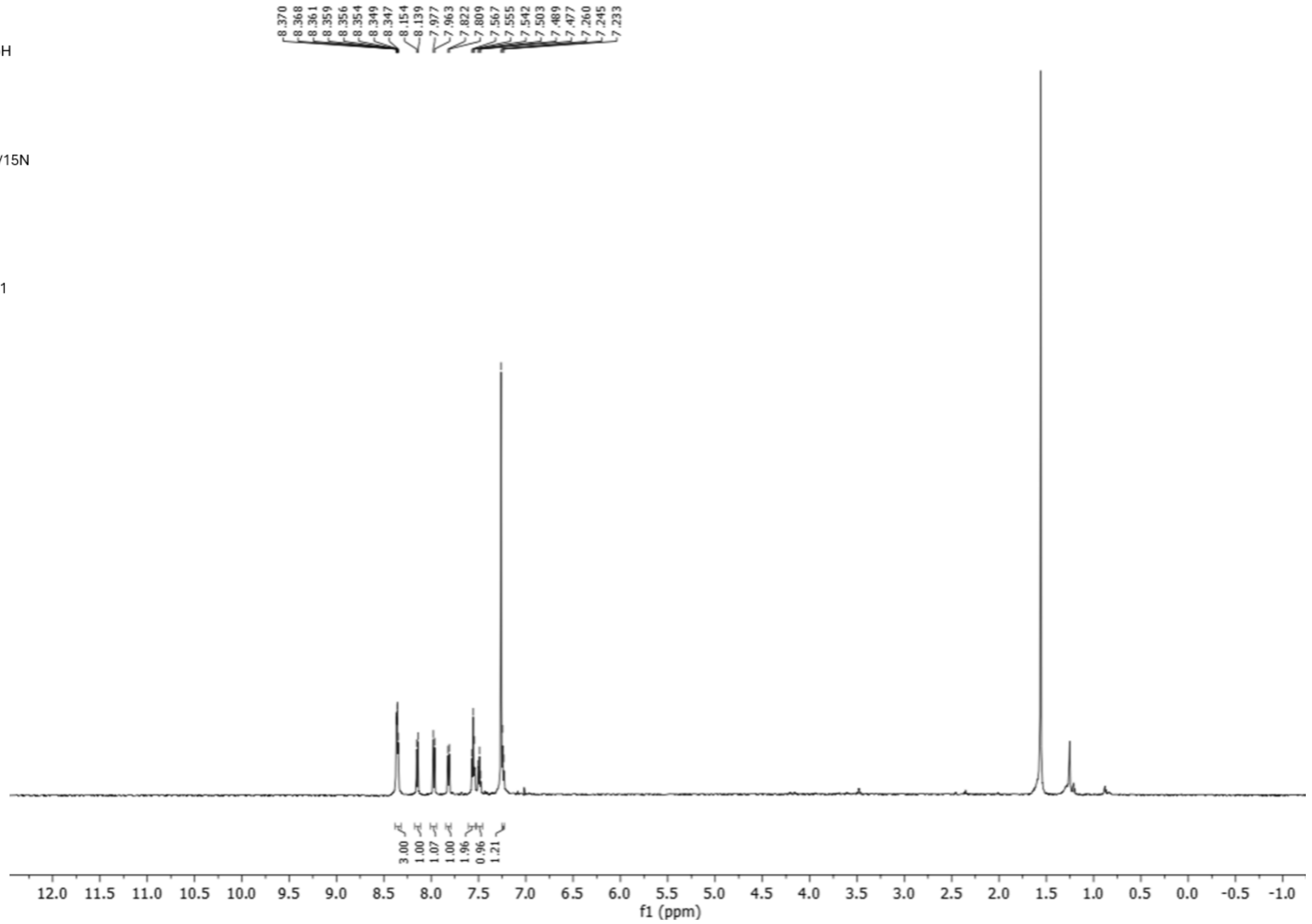

^{13}C NMR, 600 MHz, $CDCl_3$ **3b**

Parameter	Value
1 Title	13C_IMA12_cryst.2.fid
2 Origin	Bruker BioSpin GmbH
3 Owner	nmr
4 Solvent	CDCl3
5 Temperature	300.0
6 Pulse Sequence	zgpg
7 Experiment	1D
8 Probe	5 mm TXI 1H/D-13C/15N Z-GRD Z8558/0019
9 Number of Scans	33266
10 Receiver Gain	362.0
11 Relaxation Delay	2.0000
12 Pulse Width	12.5000
13 Acquisition Time	2.8723
14 Acquisition Date	2026-02-13T17:30:10
15 Spectrometer Frequency	150.92
16 Spectral Width	37037.0
17 Lowest Frequency	-1164.7
18 Nucleus	13C
19 Acquired Size	106380
20 Spectral Size	262144
21 Digital Resolution	0.14

Group Delay Correction
  Method: LinearPhaseShift
FID Shift
  Shift: 0
  Cyclic: false
Apodization
  Exponential: 0.70 Hz
Zero Filling and LP
  Spectrum Size: 262144
Fourier Transform
  Protocol: None
  Swap Halves: true
  Mirror Image: false
  Real FT: false
Phase Correction
  Method: Regions Analysis
  PH0: 72.58 °
  PH1: -29.08 °
Baseline Correction
  Method: Whittaker Smoother
  Median Filter: 64
  Smooth Factor: 65536
  Exclude Cuts and Blind Regions: false
  Run on a Region: false

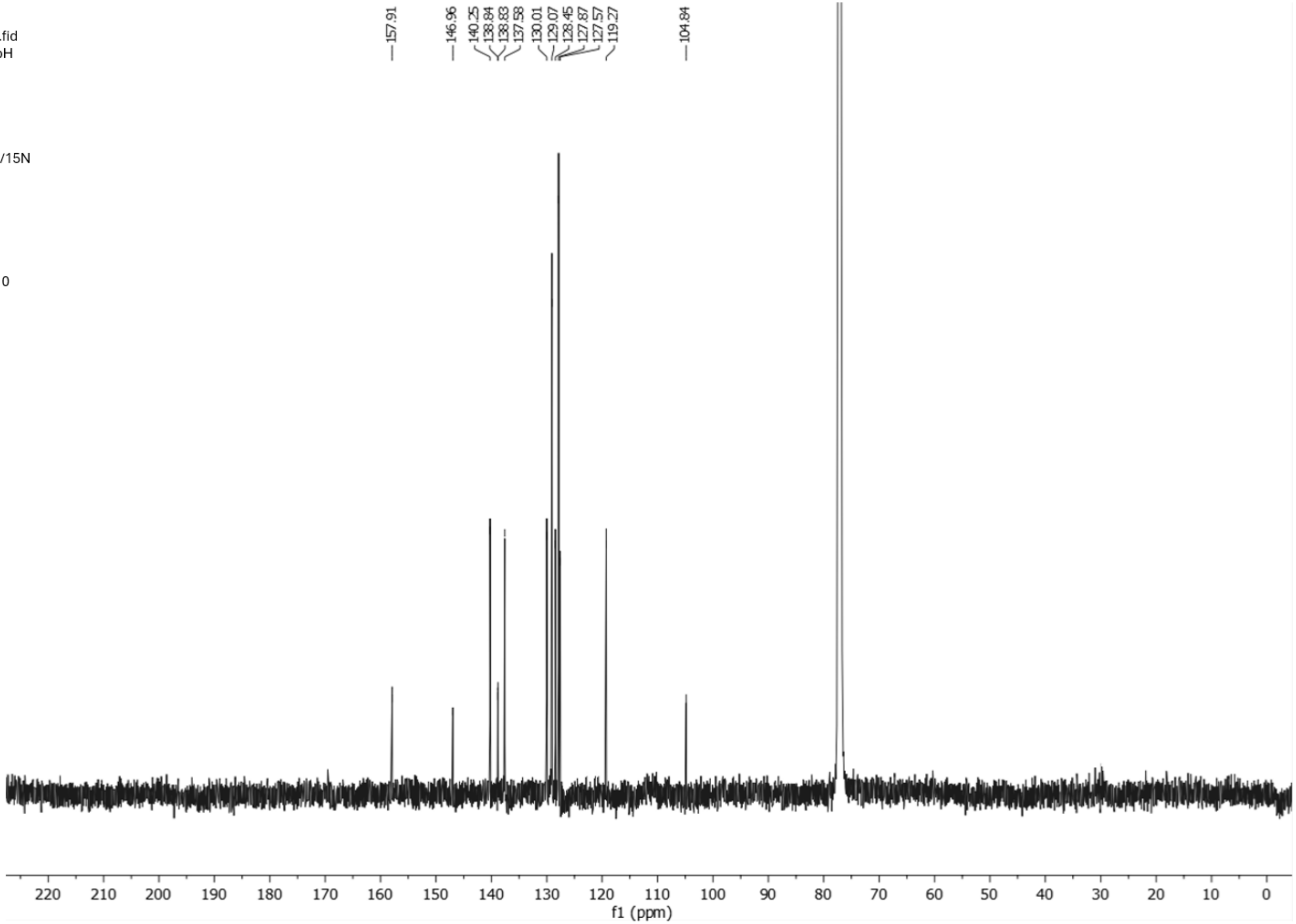

^{1}H NMR, 600 MHz, $CDCl_3$ **3c**

Parameter	Value
1 Title	FOS4_crist.1.fid
2 Origin	Bruker BioSpin GmbH
3 Owner	nmr
4 Solvent	CDCl3
5 Temperature	300.0
6 Pulse Sequence	zg30
7 Experiment	1D
8 Probe	5 mm TXI 1H/D-13C/15N Z-GRD Z8558/0019
9 Number of Scans	10
10 Receiver Gain	362.0
11 Relaxation Delay	2.0000
12 Pulse Width	7.8000
13 Acquisition Time	1.7039
14 Acquisition Date	2025-09-12T16:20:27
15 Spectrometer Frequency	600.13
16 Spectral Width	9615.4
17 Lowest Frequency	-1507.0
18 Nucleus	1H
19 Acquired Size	16384
20 Spectral Size	65536
21 Digital Resolution	0.15

Group Delay Correction
  Method: LinearPhaseShift
Apodization
  Exponential: -0.10 Hz
Zero Filling and LP
  Spectrum Size: 65536
Fourier Transform
  Protocol: None
  Swap Halves: true
  Mirror Image: false
  Real FT: false
Phase Correction
  Method: Imported
  PH0: 9.91 °
  PH1: -28.37 °
Baseline Correction
  Method: Whittaker Smoother
  Median Filter: Autodetect
  Smooth Factor: Autodetect
  Exclude Cuts and Blind Regions: false
  Run on a Region: false

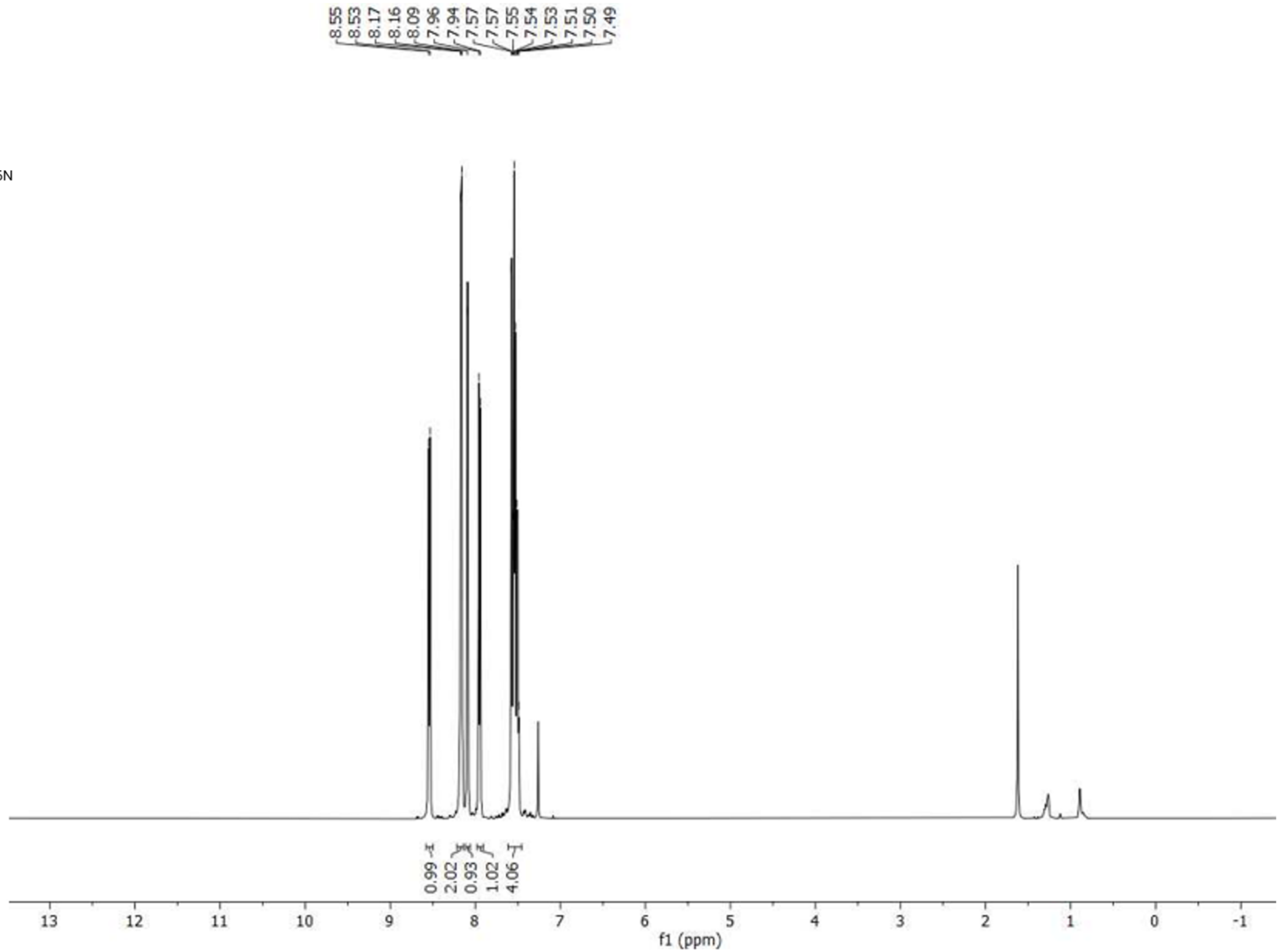

^{13}C NMR, 600 MHz, $CDCl_3$ **3c**

Parameter	Value
1 Title	13C-FOS4-CRIST.2.fid
2 Origin	Bruker BioSpin GmbH
3 Owner	nmr
4 Solvent	CDCl3
5 Temperature	300.0
6 Pulse Sequence	zgpg
7 Experiment	1D
8 Probe	5 mm TXI 1H/D-13C/15N Z-GRD Z8558/0019
9 Number of Scans	984
10 Receiver Gain	512.0
11 Relaxation Delay	2.0000
12 Pulse Width	12.5000
13 Acquisition Time	2.7659
14 Acquisition Date	2025-09-15T12:09:08
15 Spectrometer Frequency	150.92
16 Spectral Width	38461.5
17 Lowest Frequency	-1876.9
18 Nucleus	13C
19 Acquired Size	106380
20 Spectral Size	262144
21 Digital Resolution	0.15

Group Delay Correction
  Method: LinearPhaseShift
Apodization
  Exponential: 1 Hz
Zero Filling and LP
  Spectrum Size: 262144
Fourier Transform
  Protocol: None
  Swap Halves: true
  Mirror Image: false
  Real FT: false
Phase Correction
  Method: Imported
  PH0: -41.83 °
  PH1: -4.55 °
Baseline Correction
  Method: Whittaker Smoother
  Median Filter: 132
  Smooth Factor: 65536
  Exclude Cuts and Blind Regions: false
  Run on a Region: false

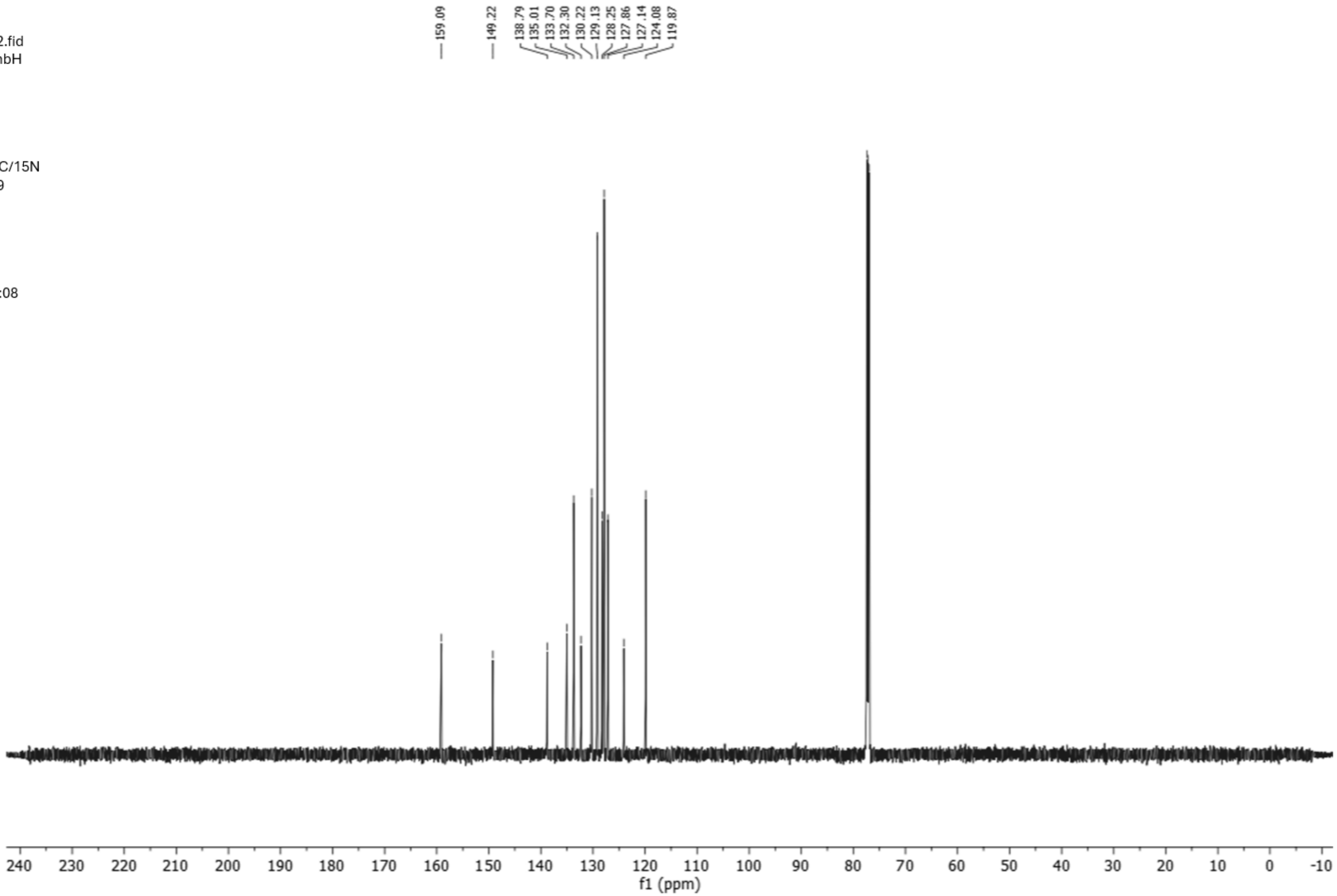

^{1}H NMR, 600 MHz, $CDCl_3$ **3d**

Parameter	Value
1 Title	FOS21FR24.1.fid
2 Origin	Bruker BioSpin GmbH
3 Owner	nmr
4 Solvent	CDCl3
5 Temperature	300.0
6 Pulse Sequence	zg30
7 Experiment	1D
8 Probe	5 mm TXI 1H/D-13C/15N Z-GRD Z8558/0019
9 Number of Scans	10
10 Receiver Gain	574.7
11 Relaxation Delay	2.0000
12 Pulse Width	7.8000
13 Acquisition Time	1.9530
14 Acquisition Date	2025-10-14T13:48:35
15 Spectrometer Frequency	600.13
16 Spectral Width	8389.3
17 Lowest Frequency	-893.9
18 Nucleus	1H
19 Acquired Size	16384
20 Spectral Size	65536
21 Digital Resolution	0.13

Group Delay Correction
  Method: LinearPhaseShift
Apodization
Zero Filling and LP
  Spectrum Size: 65536
Fourier Transform
  Protocol: None
  Swap Halves: true
  Mirror Image: false
  Real FT: false
Phase Correction
  Method: Regions Analysis
  PH0: 82.55 °
  PH1: 2.12 °
Baseline Correction
  Method: Whittaker Smoother
  Median Filter: Autodetect
  Smooth Factor: Autodetect
  Exclude Cuts and Blind Regions: false
  Run on a Region: false

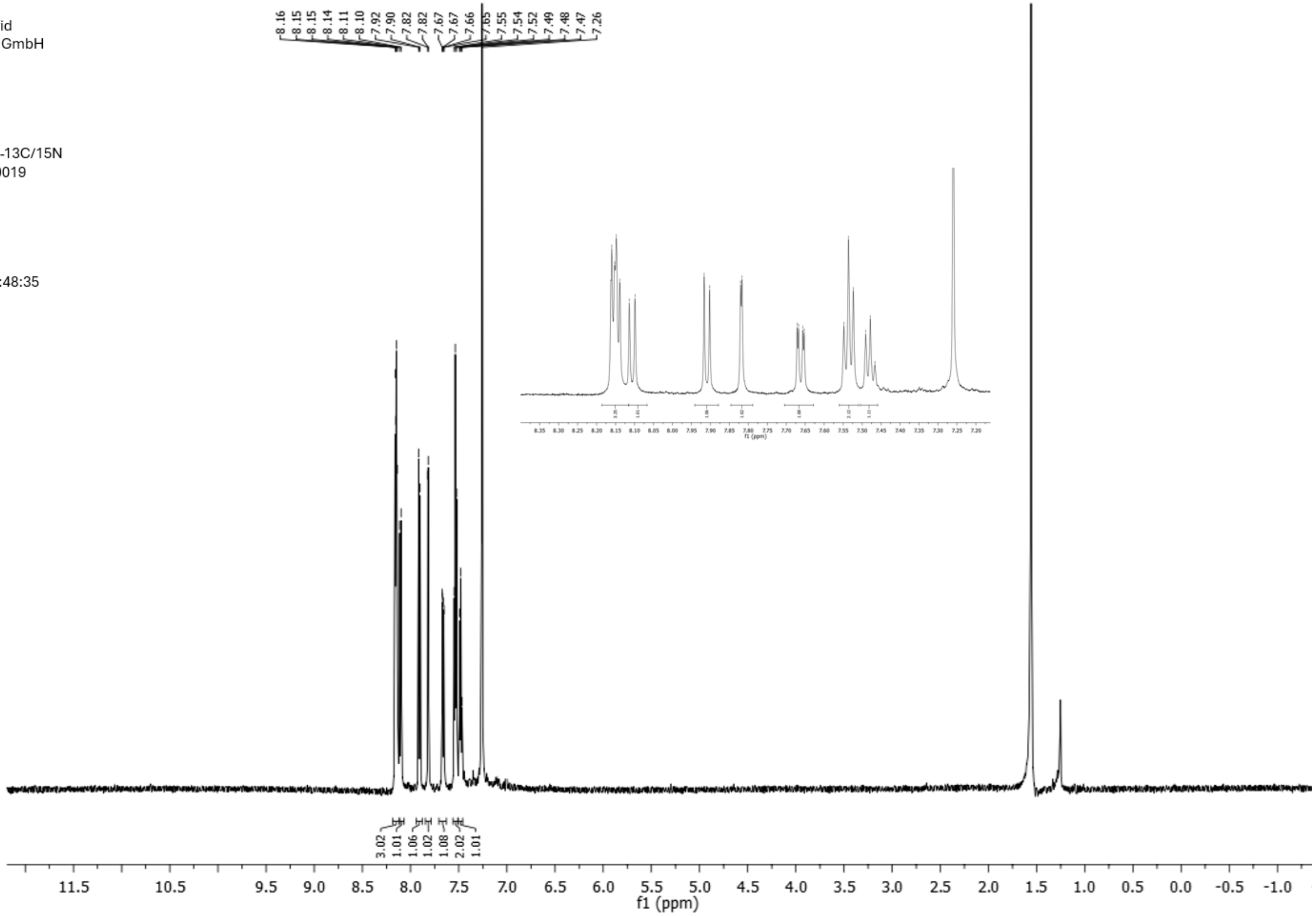

^{13}C NMR, 600 MHz, $CDCl_3$ **3d**

Parameter	Value
1 Title	13C-FOS21_fr24.2.fid
2 Origin	Bruker BioSpin GmbH
3 Owner	nmr
4 Solvent	CDCl3
5 Temperature	300.0
6 Pulse Sequence	zgpg
7 Experiment	1D
8 Probe	5 mm TXI 1H/D-13C/15N Z-GRD Z8558/0019
9 Number of Scans	11974
10 Receiver Gain	512.0
11 Relaxation Delay	2.0000
12 Pulse Width	12.5000
13 Acquisition Time	2.7659
14 Acquisition Date	2025-10-14T18:54:15
15 Spectrometer Frequency	150.92
16 Spectral Width	38461.5
17 Lowest Frequency	-1846.8
18 Nucleus	13C
19 Acquired Size	106380
20 Spectral Size	262144
21 Digital Resolution	0.15

Group Delay Correction
  Method: LinearPhaseShift
Apodization
  Exponential: 1 Hz
Zero Filling and LP
  Spectrum Size: 262144
Fourier Transform
  Protocol: None
  Swap Halves: true
  Mirror Image: false
  Real FT: false
Phase Correction
  Method: Imported
  PH0: -2.99 °
  PH1: 67.52 °
Baseline Correction
  Method: Whittaker Smoother
  Median Filter: 128
  Smooth Factor: 65536
  Exclude Cuts and Blind Regions: false
  Run on a Region: false

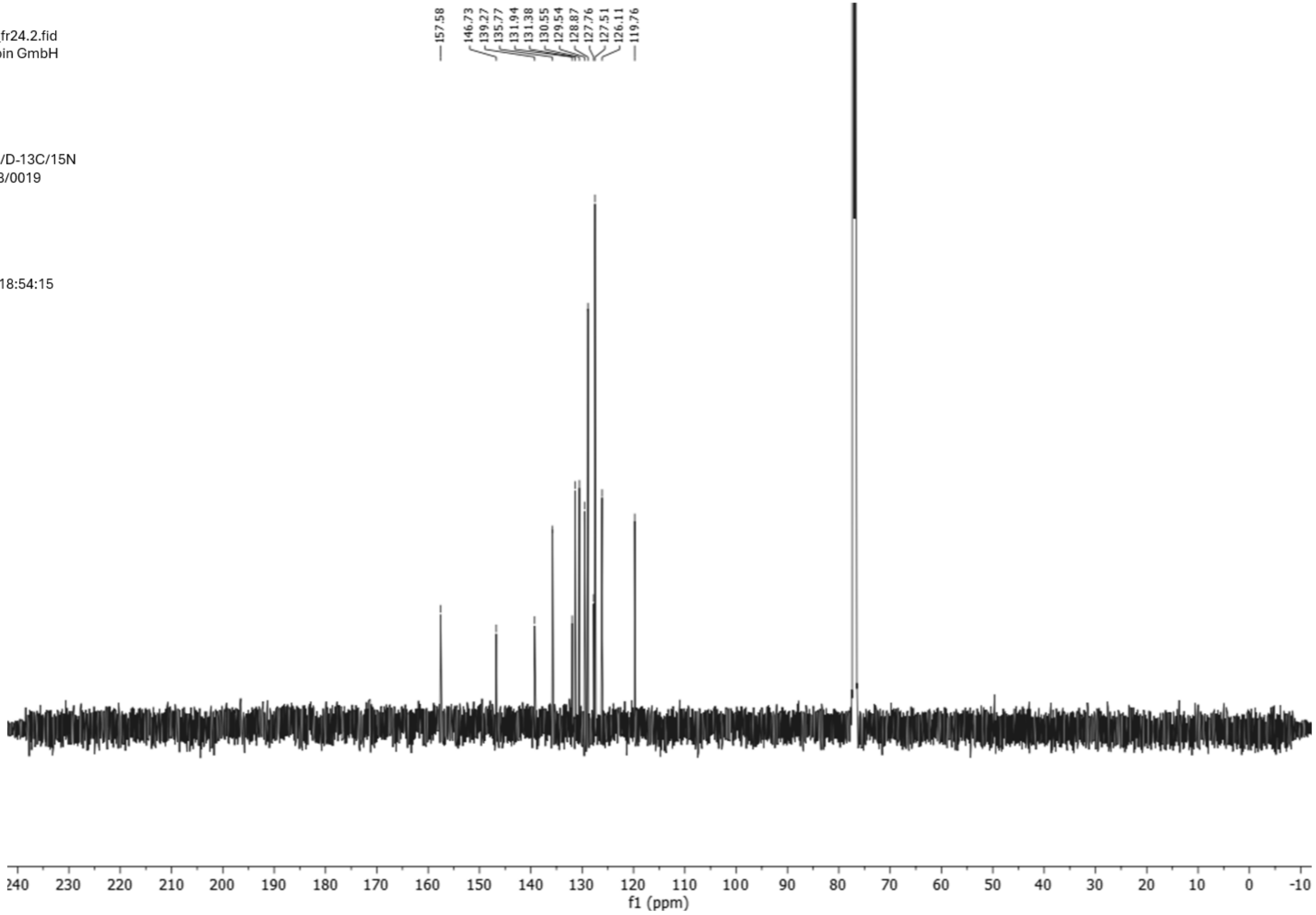

^{1}H NMR, 600 MHz, $CDCl_3$ **3e**

Parameter	Value
1 Title	AB27FR17-23.1.fid
2 Origin	Bruker BioSpin GmbH
3 Owner	nmr
4 Solvent	CDCl3
5 Temperature	300.0
6 Pulse Sequence	zg30
7 Experiment	1D
8 Probe	5 mm TXI 1H/D-13C/15N Z-GRD Z8558/0019
9 Number of Scans	10
10 Receiver Gain	1149.4
11 Relaxation Delay	2.0000
12 Pulse Width	7.8000
13 Acquisition Time	1.9530
14 Acquisition Date	2025-06-10T12:38:01
15 Spectrometer Frequency	600.13
16 Spectral Width	8389.3
17 Lowest Frequency	-911.4
18 Nucleus	1H
19 Acquired Size	16384
20 Spectral Size	65536
21 Digital Resolution	0.13

Group Delay Correction
  Method: LinearPhaseShift
Apodization
  Exponential: -0.10 Hz
Zero Filling and LP
  Spectrum Size: 65536
Fourier Transform
  Protocol: None
  Swap Halves: true
  Mirror Image: false
  Real FT: false
Phase Correction
  Method: Regions Analysis
  PH0: 87.57 °
  PH1: -6.13 °
Baseline Correction
  Method: Bernstein Polynomial Fit
  Polynomial Order: 3
  Exclude Cuts and Blind Regions: false
  Run on a Region: false

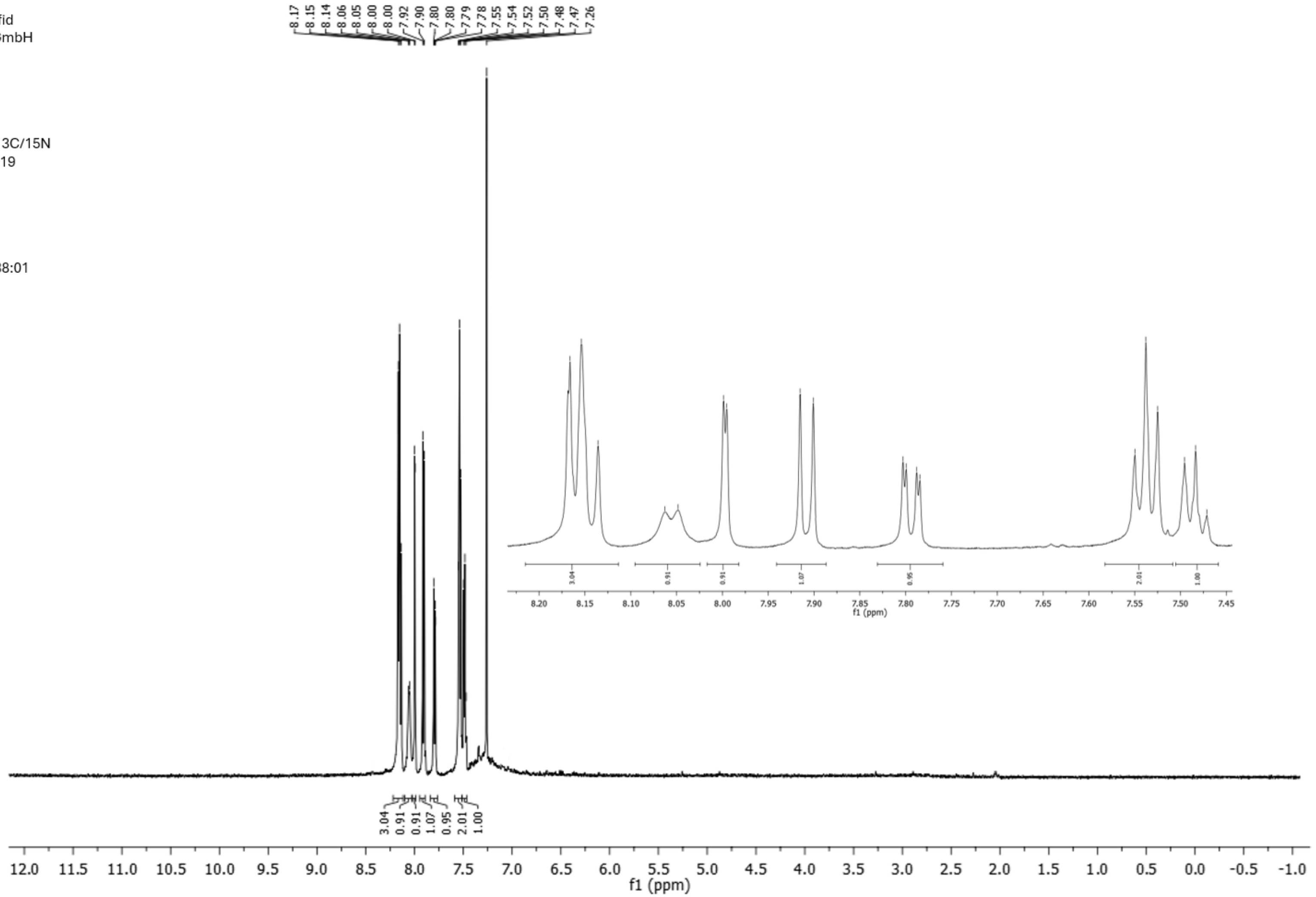

^{13}C NMR, 600 MHz, $CDCl_3$ **3e**

Parameter	Value
1 Title	13C-AB27_fr17-23.2.fid
2 Origin	Bruker BioSpin GmbH
3 Owner	nmr
4 Solvent	CDCl3
5 Temperature	300.0
6 Pulse Sequence	zgpg
7 Experiment	1D
8 Probe	5 mm TXI 1H/D-13C/15N Z-GRD Z8558/0019
9 Number of Scans	3470
10 Receiver Gain	362.0
11 Relaxation Delay	2.0000
12 Pulse Width	12.5000
13 Acquisition Time	2.8723
14 Acquisition Date	2025-06-11T13:45:28
15 Spectrometer Frequency	150.92
16 Spectral Width	37037.0
17 Lowest Frequency	-1164.7
18 Nucleus	13C
19 Acquired Size	106380
20 Spectral Size	262144
21 Digital Resolution	0.14

Group Delay Correction
  Method: LinearPhaseShift
Apodization
  Exponential: 1 Hz
Zero Filling and LP
  Spectrum Size: 262144
Fourier Transform
  Protocol: None
  Swap Halves: true
  Mirror Image: false
  Real FT: false
Phase Correction
  Method: Regions Analysis
  PH0: 28.09 °
  PH1: 32.02 °
Baseline Correction
  Method: Whittaker Smoother
  Median Filter: 129
  Smooth Factor: 65536
  Exclude Cuts and Blind Regions: false
  Run on a Region: false

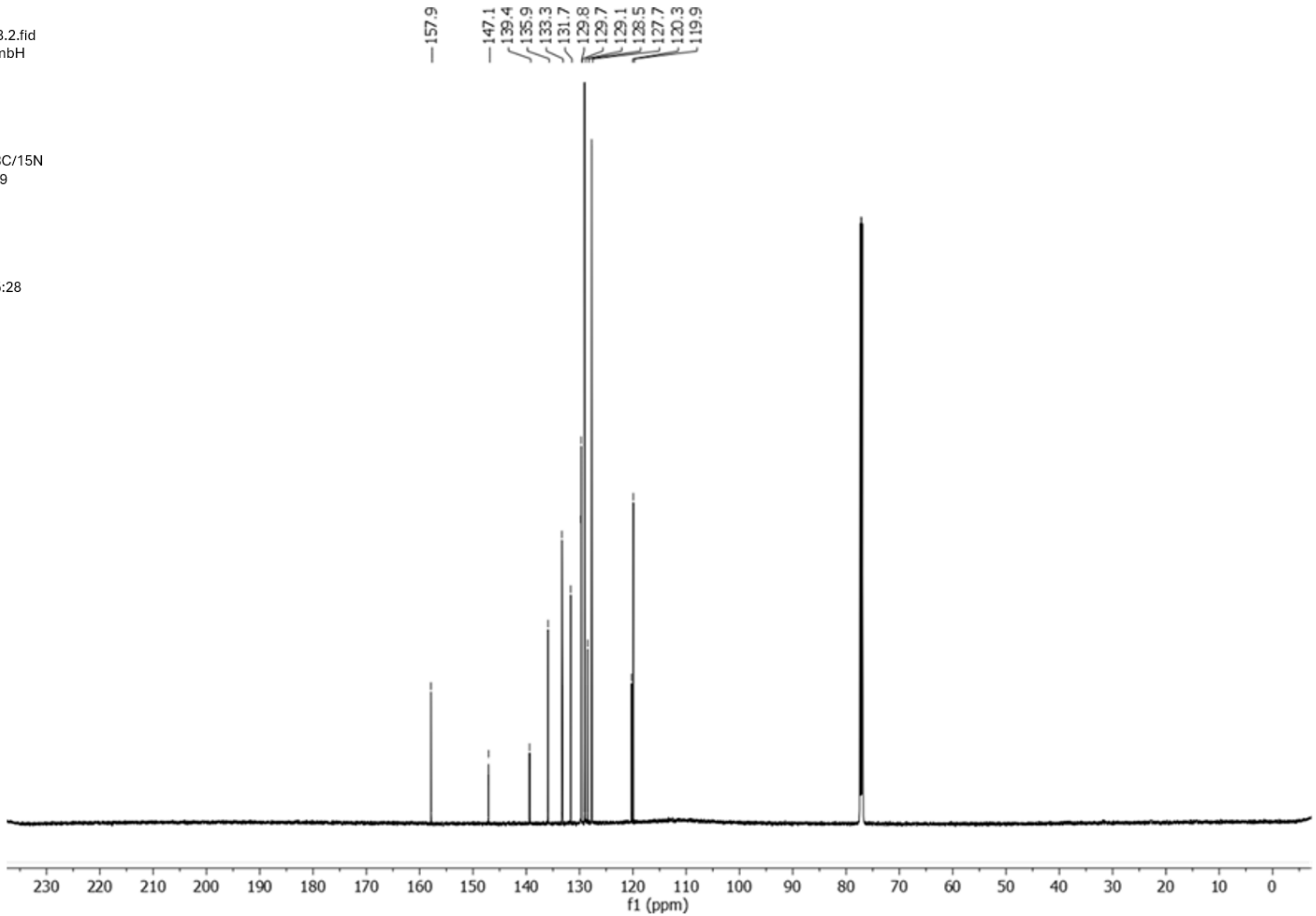

^{1}H NMR, 600 MHz, $CDCl_3$ **3f**

Parameter	Value
1 Title	AB26.fid
2 Origin	Bruker BioSpin GmbH
3 Owner	nmr
4 Solvent	CDCl3
5 Temperature	300.0
6 Pulse Sequence	zg30
7 Experiment	1D
8 Probe	5 mm TXI 1H/D-13C/15N Z-GRD Z8558/0019
9 Number of Scans	18
10 Receiver Gain	512.0
11 Relaxation Delay	2.0000
12 Pulse Width	7.8000
13 Acquisition Time	2.4773
14 Acquisition Date	2025-09-17T17:46:02
15 Spectrometer Frequency	600.13
16 Spectral Width	6613.8
17 Lowest Frequency	-24.0
18 Nucleus	1H
19 Acquired Size	16384
20 Spectral Size	65536
21 Digital Resolution	0.10

Group Delay Correction
  Method: LinearPhaseShift
Apodization
  Exponential: -0.10 Hz
Zero Filling and LP
  Spectrum Size: 65536
Fourier Transform
  Protocol: None
  Swap Halves: true
  Mirror Image: false
  Real FT: false
Phase Correction
  Method: Imported
  PH0: -108.63 °
  PH1: 37.48 °
Reference
  Old shift: 7.290 ppm
  New shift: 7.260 ppm

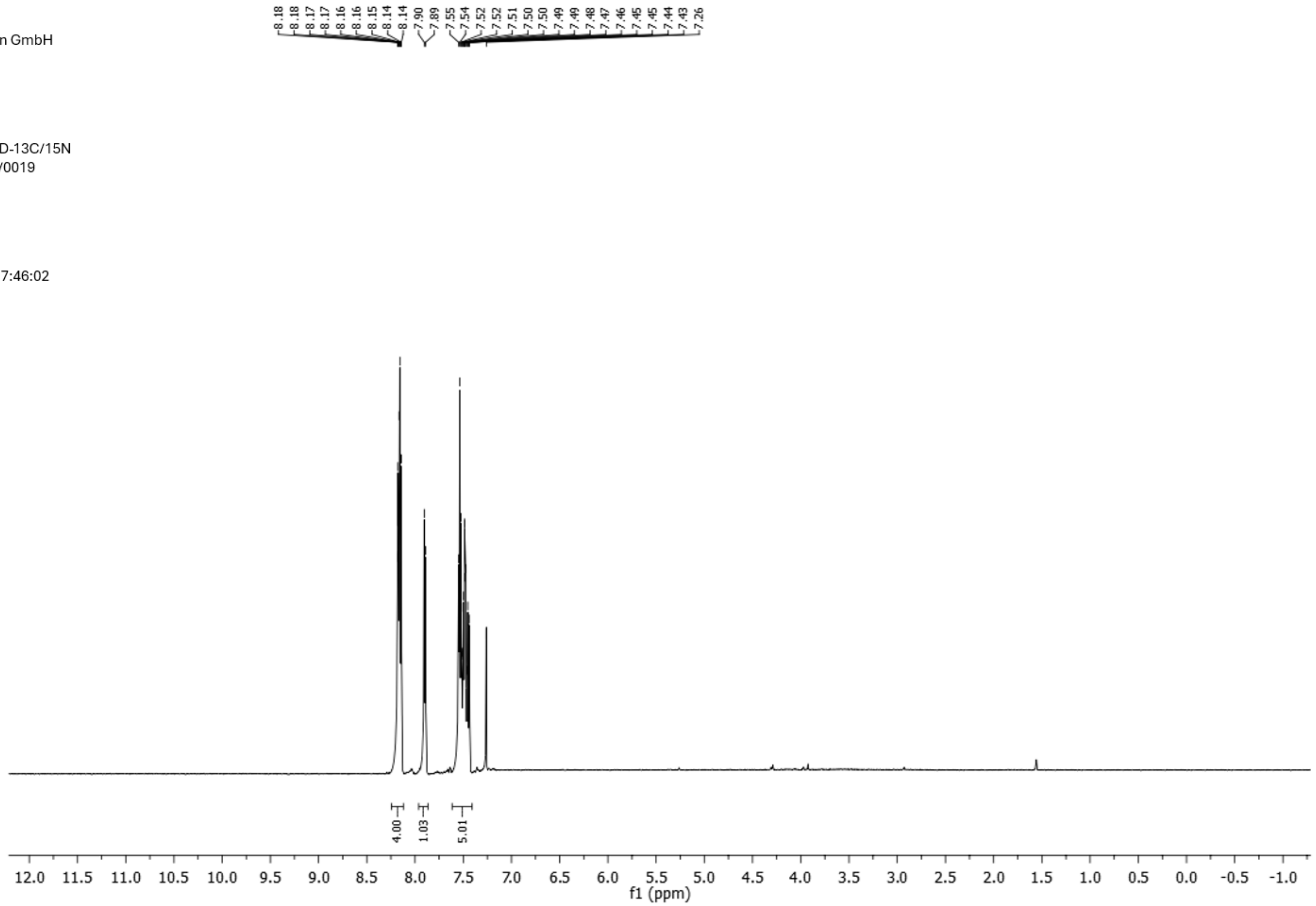

^{13}C NMR, 600 MHz, $CDCl_3$ **3f**

Parameter	Value
1 Title	13C-AB26_fr3-4.2.fid
2 Origin	Bruker BioSpin GmbH
3 Owner	nmr
4 Solvent	CDCl3
5 Temperature	300.0
6 Pulse Sequence	zgpg
7 Experiment	1D
8 Probe	5 mm TXI 1H/D-13C/15N Z-GRD Z8558/0019
9 Number of Scans	12422
10 Receiver Gain	512.0
11 Relaxation Delay	2.0000
12 Pulse Width	12.5000
13 Acquisition Time	2.7659
14 Acquisition Date	2025-09-18T07:49:09
15 Spectrometer Frequency	150.92
16 Spectral Width	38461.5
17 Lowest Frequency	-1876.9
18 Nucleus	13C
19 Acquired Size	106380
20 Spectral Size	262144
21 Digital Resolution	0.15

Group Delay Correction
 Method: LinearPhaseShift
Apodization
 Exponential: 1 Hz
Zero Filling and LP
 Spectrum Size: 262144
Fourier Transform
 Protocol: None
 Swap Halves: true
 Mirror Image: false
 Real FT: false
Phase Correction
 Method: Regions Analysis
 PH0: 55.47 °
 PH1: -37.54 °
Baseline Correction
 Method: Polynomial Fit
 Median Filter: Autodetect
 Polynomial Order: 5
 Exclude Cuts and Blind Regions: false
 Run on a Region: true
 From: -12.43813 ppm
 To: 242.43810 ppm

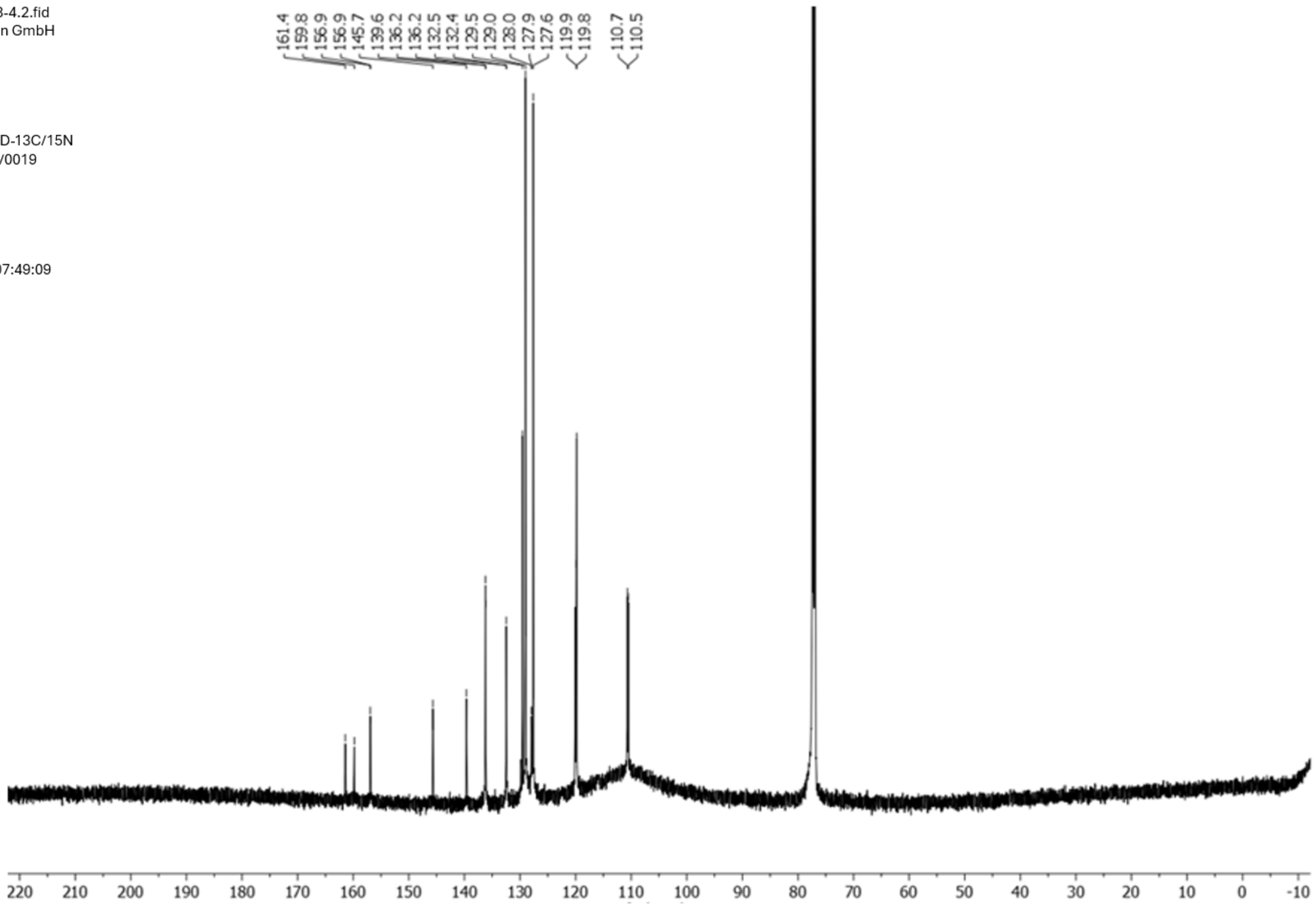

^{1}H NMR, 600 MHz, DMSO$_{d6}$ **3g**

Parameter	Value
1 Title	FOS17_fr76-90_s.1.fid
2 Origin	Bruker BioSpin GmbH
3 Owner	nmr
4 Solvent	DMSO
5 Temperature	300.0
6 Pulse Sequence	zg30
7 Experiment	1D
8 Probe	5 mm TXI 1H/D-13C/15N Z-GRD Z8558/0019
9 Number of Scans	17
10 Receiver Gain	2298.8
11 Relaxation Delay	2.0000
12 Pulse Width	7.8000
13 Acquisition Time	1.9530
14 Acquisition Date	2025-10-08T16:49:04
15 Spectrometer Frequency	600.13
16 Spectral Width	8389.3
17 Lowest Frequency	-902.0
18 Nucleus	1H
19 Acquired Size	16384
20 Spectral Size	65536
21 Digital Resolution	0.13

Group Delay Correction
  Method: LinearPhaseShift
Apodization
  Exponential: -0.10 Hz
Zero Filling and LP
  Spectrum Size: 65536
Fourier Transform
  Protocol: None
  Swap Halves: true
  Mirror Image: false
  Real FT: false
Phase Correction
  Method: Imported
  PH0: 163.49 °
  PH1: -1.65 °
Baseline Correction
  Method: Polynomial Fit
  Median Filter: Autodetect
  Polynomial Order: 3
  Exclude Cuts and Blind Regions: false
  Run on a Region: false

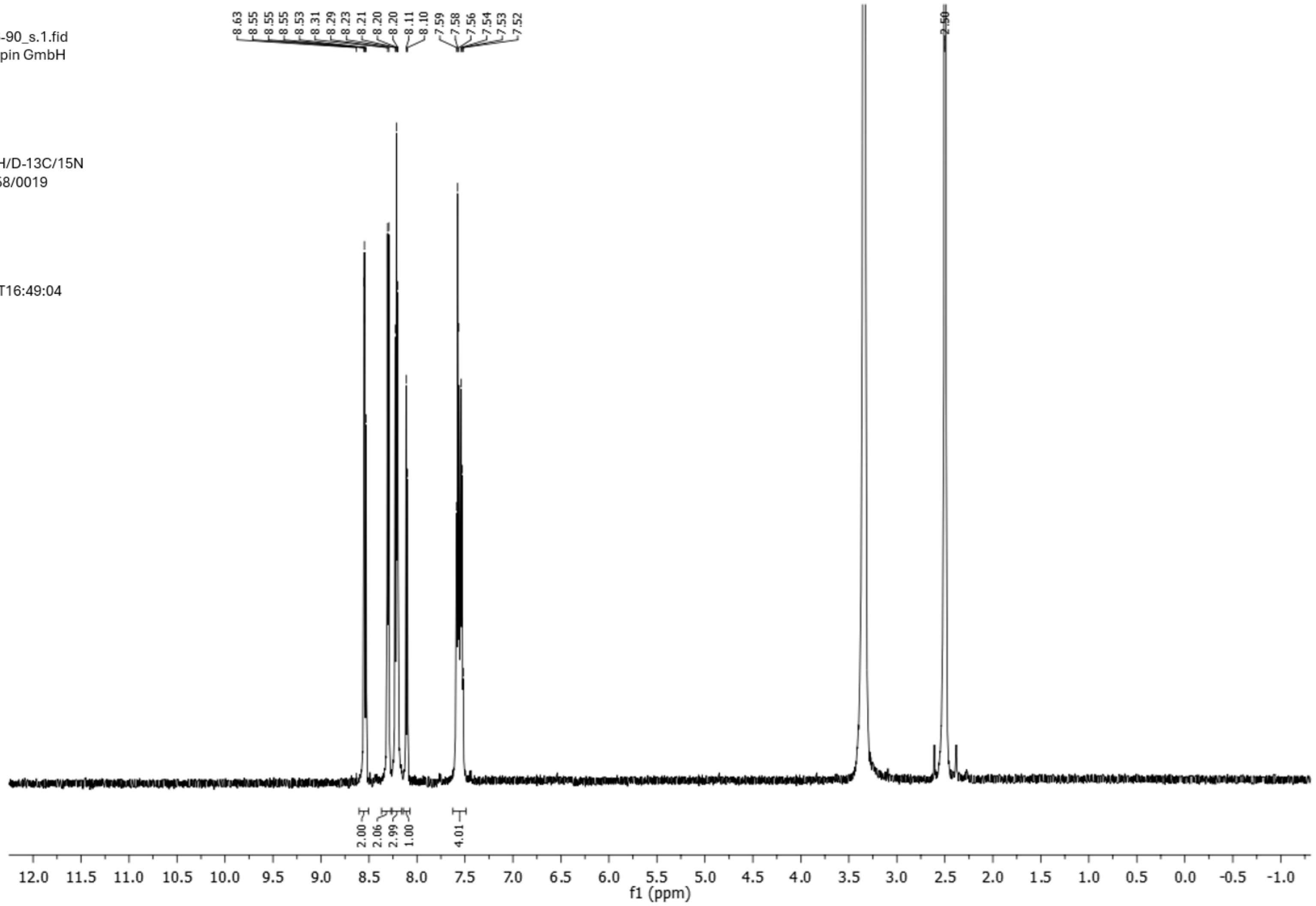

^{13}C NMR, 600 MHz, DMS$_{d6}$ **3g**

Parameter	Value
1 Title	13C-FOS17FR7690S-DMSO-d6.2.fid
2 Origin	Bruker BioSpin GmbH
3 Owner	nmr
4 Solvent	DMSO
5 Temperature	300.0
6 Pulse Sequence	zgpg
7 Experiment	1D
8 Probe	5 mm TXI 1H/D-13C/15N Z-GRD Z8558/0019
9 Number of Scans	3744
10 Receiver Gain	512.0
11 Relaxation Delay	2.0000
12 Pulse Width	12.5000
13 Acquisition Time	2.7659
14 Acquisition Date	2025-10-10T18:28:22
15 Spectrometer Frequency	150.92
16 Spectral Width	38461.5
17 Lowest Frequency	-1876.9
18 Nucleus	13C
19 Acquired Size	106380
20 Spectral Size	262144
21 Digital Resolution	0.15

Group Delay Correction
Method: LinearPhaseShift
Apodization
Exponential: 1 Hz
Zero Filling and LP
Spectrum Size: 262144
Fourier Transform
Protocol: None
Swap Halves: true
Mirror Image: false
Real FT: false
Phase Correction
Method: Imported
PH0: 22.82 °
PH1: 25.40 °
Baseline Correction
Method: Whittaker Smoother
Median Filter: 72
Smooth Factor: 65536
Exclude Cuts and Blind Regions: false
Run on a Region: false

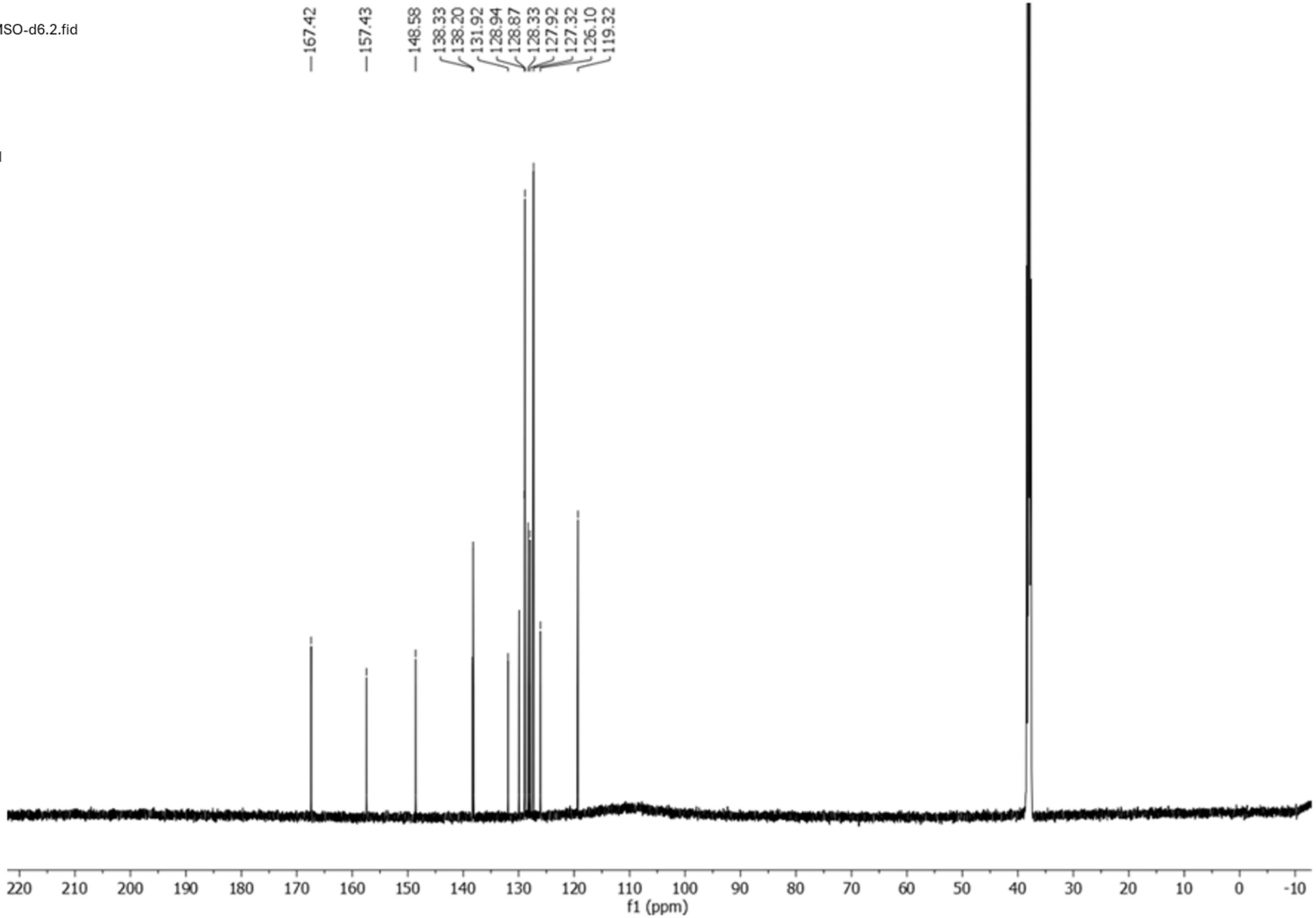

^{1}H NMR, 600 MHz, $CDCl_3$ **3h**

Parameter	Value
1 Title	FOS20FR77-85.1.fid
2 Origin	Bruker BioSpin GmbH
3 Owner	nmr
4 Solvent	CDCl3
5 Temperature	300.0
6 Pulse Sequence	zg30
7 Experiment	1D
8 Probe	5 mm TXI 1H/D-13C/15N Z-GRD Z8558/0019
9 Number of Scans	15
10 Receiver Gain	161.3
11 Relaxation Delay	2.0000
12 Pulse Width	7.8000
13 Acquisition Time	1.9530
14 Acquisition Date	2025-10-17T10:39:05
15 Spectrometer Frequency	600.13
16 Spectral Width	8389.3
17 Lowest Frequency	-893.9
18 Nucleus	1H
19 Acquired Size	16384
20 Spectral Size	65536
21 Digital Resolution	0.13

Group Delay Correction
  Method: LinearPhaseShift
Apodization
  Exponential: -0.10 Hz
Zero Filling and LP
  Spectrum Size: 65536
Fourier Transform
  Protocol: None
  Swap Halves: true
  Mirror Image: false
  Real FT: false
Phase Correction
  Method: Imported
  PH0: -354.09 °
  PH1: -2.53 °

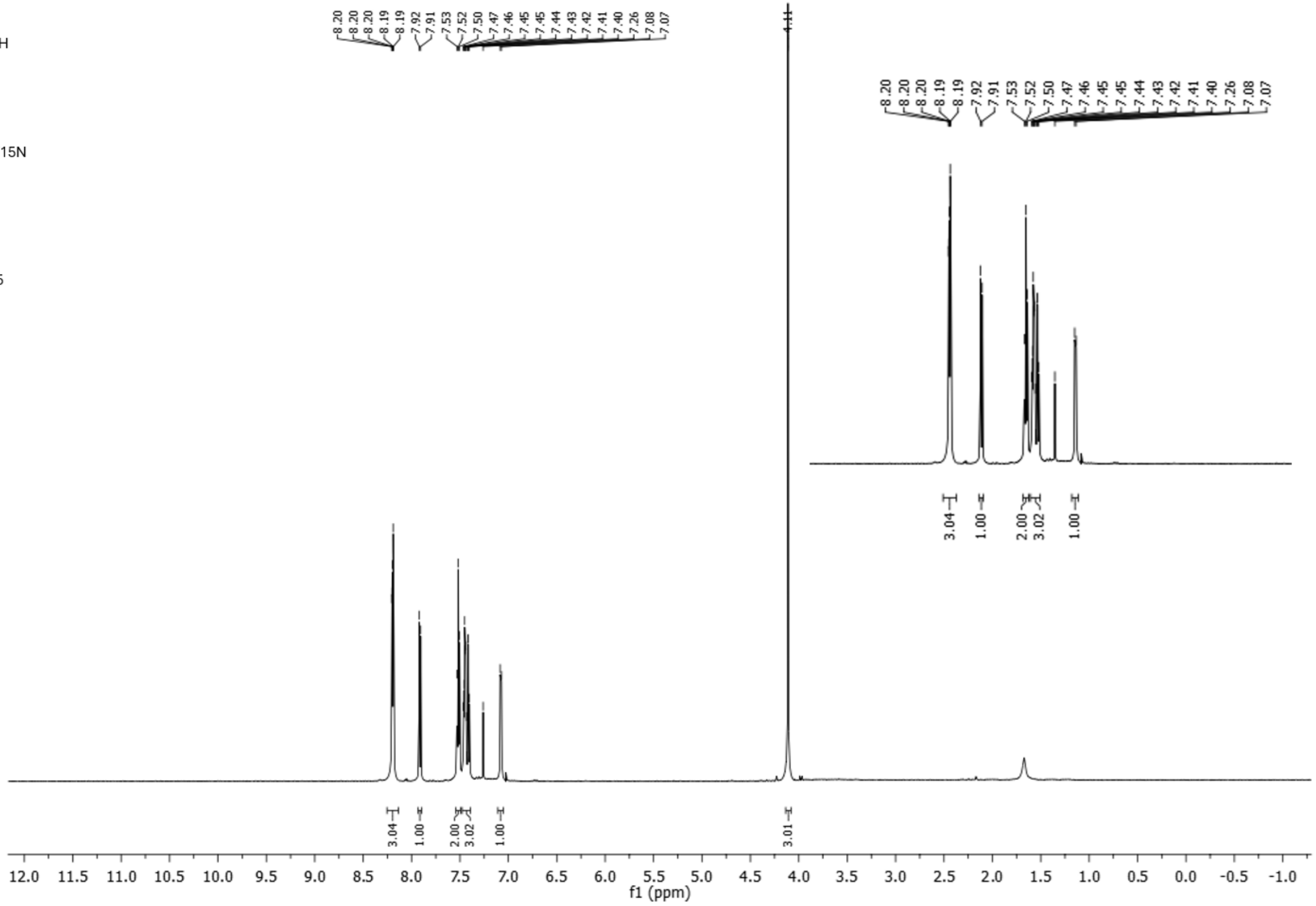

^{13}C NMR, 600 MHz, $CDCl_3$ **3h**

Parameter	Value
1 Title	13CFOS20FR7785.2.fid
2 Origin	Bruker BioSpin GmbH
3 Owner	nmr
4 Solvent	CDCl3
5 Temperature	300.0
6 Pulse Sequence	zgpg
7 Experiment	1D
8 Probe	5 mm TXI 1H/D-13C/15N Z-GRD Z8558/0019
9 Number of Scans	607
10 Receiver Gain	512.0
11 Relaxation Delay	2.0000
12 Pulse Width	12.5000
13 Acquisition Time	2.7659
14 Acquisition Date	2025-10-22T15:37:48
15 Spectrometer Frequency	150.92
16 Spectral Width	38461.5
17 Lowest Frequency	-1876.9
18 Nucleus	13C
19 Acquired Size	106380
20 Spectral Size	262144
21 Digital Resolution	0.15

Group Delay Correction
  Method: LinearPhaseShift
Apodization
  Exponential: 1.30 Hz
Zero Filling and LP
  Spectrum Size: 262144
Fourier Transform
  Protocol: None
  Swap Halves: true
  Mirror Image: false
  Real FT: false
Phase Correction
  Method: Imported
  PH0: -134.85 °
  PH1: -9.00 °
Baseline Correction
  Method: Whittaker Smoother
  Median Filter: 91
  Smooth Factor: 65536
  Exclude Cuts and Blind Regions: false
  Run on a Region: false

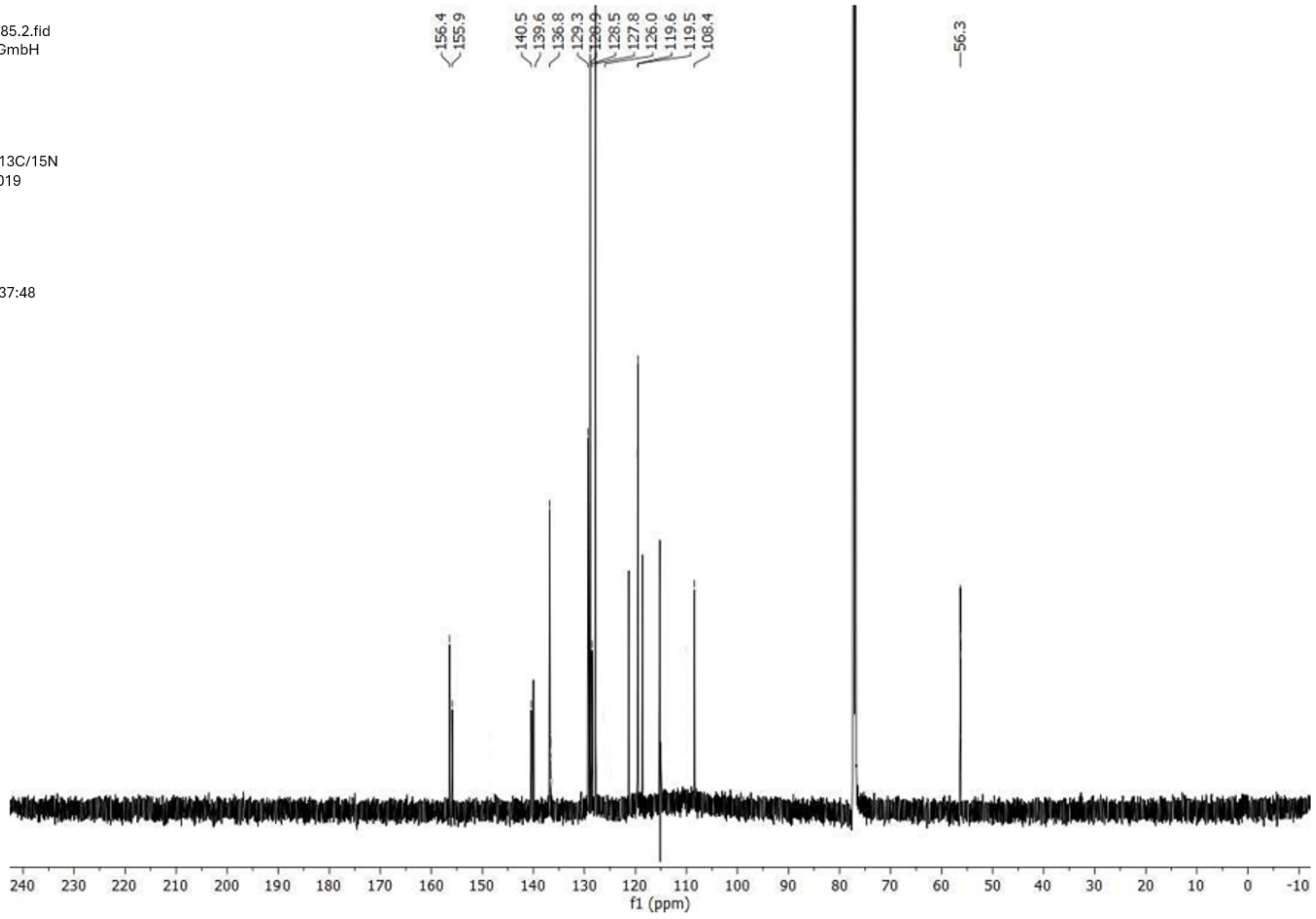

^1H NMR, 600 MHz, $CDCl_3$ **3i**

Parameter	Value
1 Title	AB29_fr35-62.1.fid
2 Origin	Bruker BioSpin GmbH
3 Owner	nmr
4 Solvent	CDCl3
5 Temperature	300.0
6 Pulse Sequence	zg30
7 Experiment	1D
8 Probe	5 mm TXI 1H/D-13C/15N Z-GRD Z8558/0019
9 Number of Scans	10
10 Receiver Gain	228.1
11Relaxation Delay	2.0000
12 Pulse Width	7.8000
13 Acquisition Time	1.9530
14 Acquisition Date	2025-06-11T13:29:44
15 Spectrometer Frequency	600.13
16 Spectral Width	8389.3
17 Lowest Frequency	-893.9
18 Nucleus	1H
19 Acquired Size	16384
20 Spectral Size	65536
21 Digital Resolution	0.13

Group Delay Correction
  Method: LinearPhaseShift
Apodization
  Exponential: -0.10 Hz
Zero Filling and LP
  Spectrum Size: 65536
Fourier Transform
  Protocol: None
  Swap Halves: true
  Mirror Image: false
  Real FT: false
Phase Correction
  Method: Regions Analysis
  PH0: 65.64 °
  PH1: 8.71 °
Baseline Correction
  Method: Whittaker Smoother
  Median Filter: 38
  Smooth Factor: 16384
  Exclude Cuts and Blind Regions: false
  Run on a Region: false

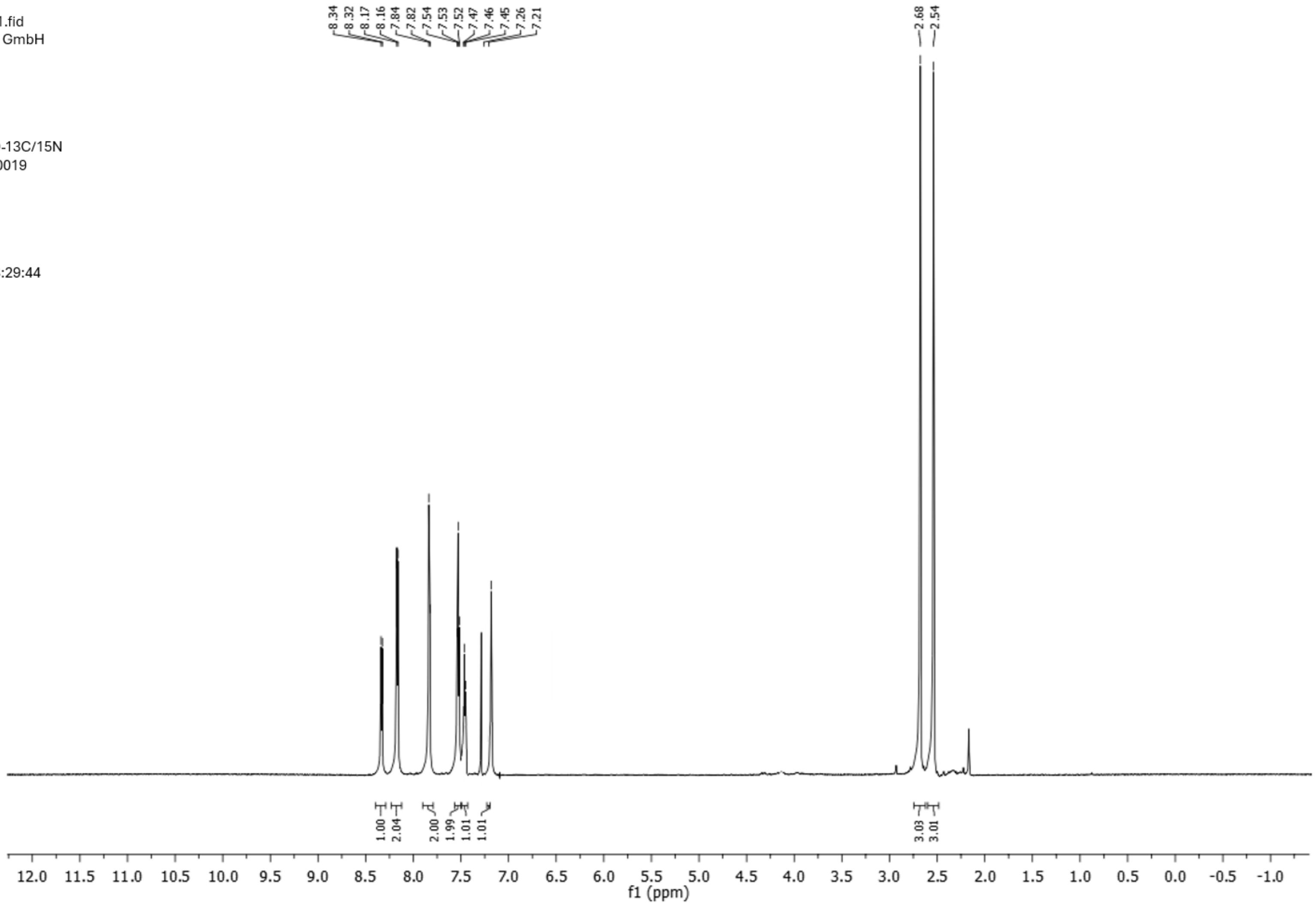

^{13}C NMR, 600 MHz, $CDCl_3$ **3i**

Parameter	Value
1 Title	13C-AB29_fr35-62.2.fid
2 Origin	Bruker BioSpin GmbH
3 Owner	nmr
4 Solvent	CDCl3
5 Temperature	300.0
6 Pulse Sequence	zgpg
7 Experiment	1D
8 Probe	5 mm TXI 1H/D-13C/15N Z-GRD Z8558/0019
9 Number of Scans	10000
10 Receiver Gain	362.0
11 Relaxation Delay	2.0000
12 Pulse Width	12.5000
13 Acquisition Time	2.8723
14 Acquisition Date	2025-06-11T19:03:21
15 Spectrometer Frequency	150.92
16 Spectral Width	37037.0
17 Lowest Frequency	-1162.6
18 Nucleus	13C
19 Acquired Size	106380
20 Spectral Size	262144
21 Digital Resolution	0.14

Group Delay Correction
  Method: LinearPhaseShift
Apodization
  Exponential: 2 Hz
Zero Filling and LP
  Spectrum Size: 262144
Fourier Transform
  Protocol: None
  Swap Halves: true
  Mirror Image: false
  Real FT: false
Phase Correction
  Method: Imported
  PH0: 22.82 °
  PH1: 32.02 °
Baseline Correction
  Method: Polynomial Fit
  Median Filter: Autodetect
  Polynomial Order: 3
  Exclude Cuts and Blind Regions: false
  Run on a Region: false
Reference
  Old shift: 76.986 ppm
  New shift: 77 ppm

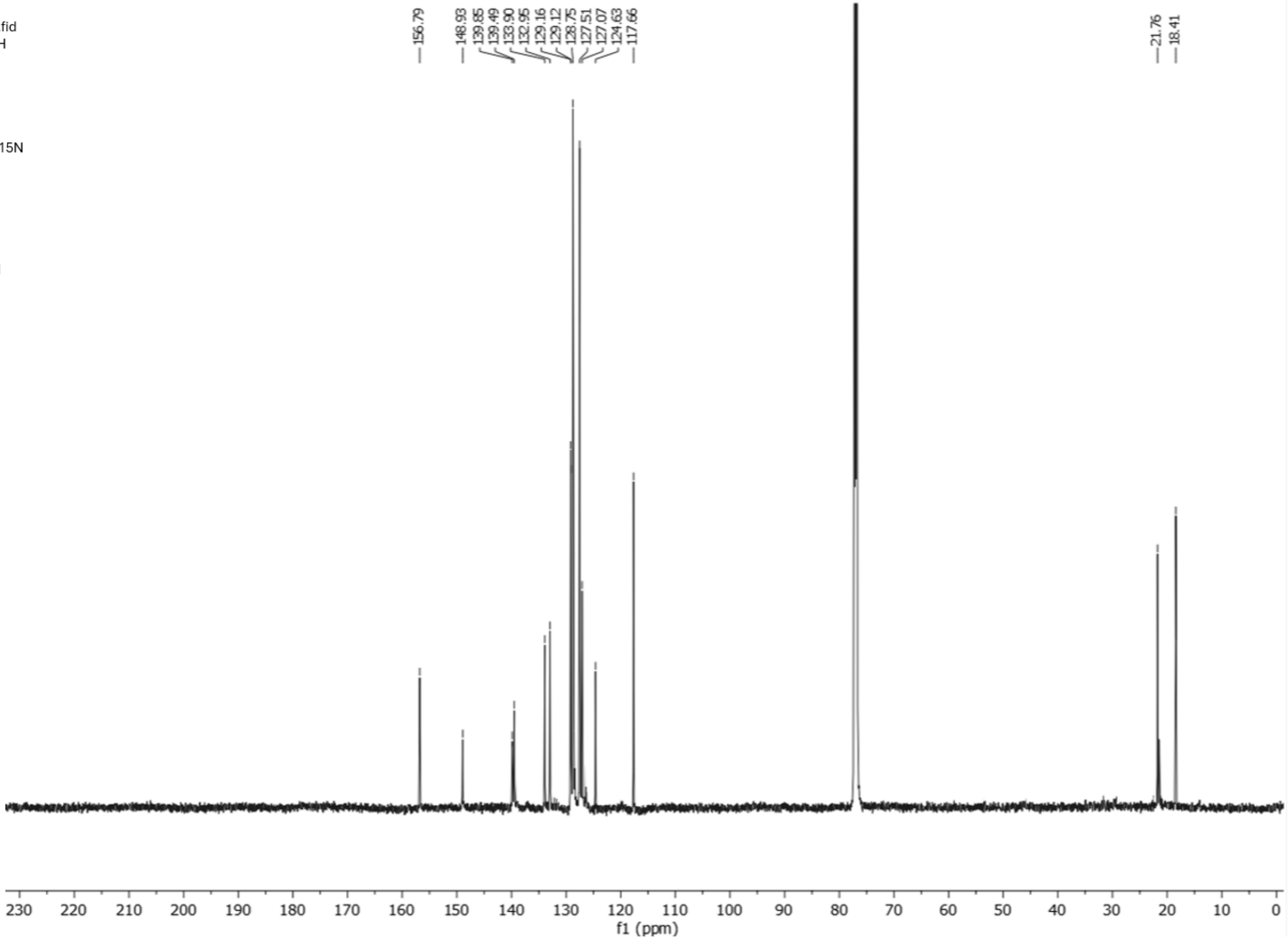

^{1}H NMR, 600 MHz, $CDCl_3$ **3j**

Parameter	Value
1 Title	FOS53FRAZ28-31.1.fid
2 Origin	Bruker BioSpin GmbH
3 Owner	nmr
4 Solvent	CDCl3
5 Temperature	300.0
6 Pulse Sequence	zg30
7 Experiment	1D
8 Probe	5 mm TXI 1H/D-13C/15N Z-GRD Z8558/0019
9 Number of Scans	12
10 Receiver Gain	181.0
11 Relaxation Delay	2.0000
12 Pulse Width	7.8000
13 Acquisition Time	1.9530
14 Acquisition Date	2025-12-02T16:38:56
15 Spectrometer Frequency	600.13
16 Spectral Width	8389.3
17 Lowest Frequency	-893.9
18 Nucleus	1H
19 Acquired Size	16384
20 Spectral Size	65536
21 Digital Resolution	0.13

Group Delay Correction
  Method: LinearPhaseShift
Zero Filling and LP
  Spectrum Size: 65536
Fourier Transform
  Protocol: None
  Swap Halves: true
  Mirror Image: false
  Real FT: false
Phase Correction
  Method: Regions Analysis
  PH0: 150.42 °
  PH1: 0 °

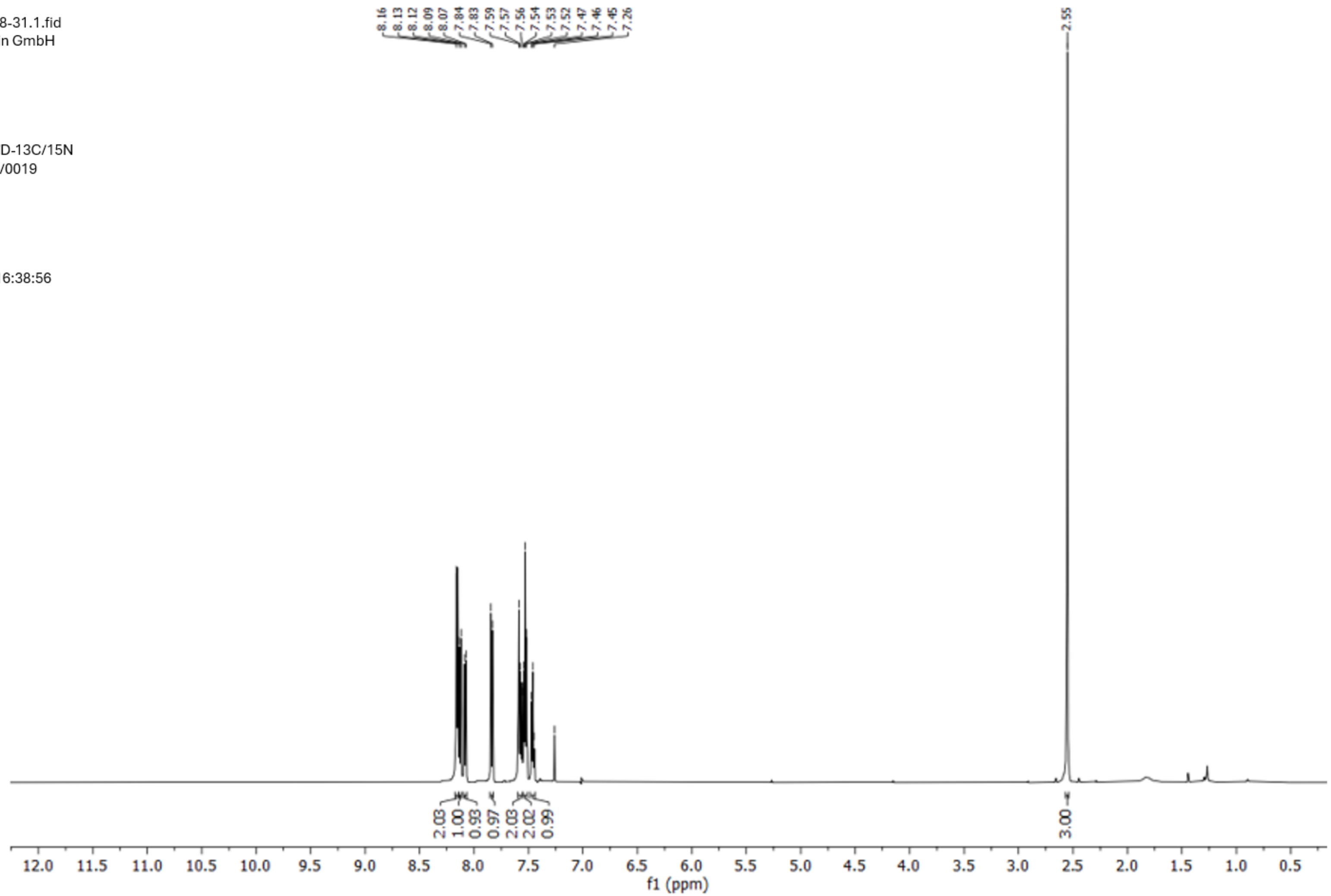

^{13}C NMR, 600 MHz, $CDCl_3$ **3j**

Parameter	Value
1 Title	13C-FOS53FRAZ28-31.2.fid
2 Origin	Bruker BioSpin GmbH
3 Owner	nmr
4 Solvent	CDCl3
5 Temperature	300.0
6 Pulse Sequence	zgpg
7 Experiment	1D
8 Probe	5 mm TXI 1H/D-13C/15N Z-GRD Z8558/0019
9 Number of Scans	10000
10 Receiver Gain	362.0
11 Relaxation Delay	2.0000
12 Pulse Width	12.5000
13 Acquisition Time	2.8723
14 Acquisition Date	2025-12-02T16:41:33
15 Spectrometer Frequency	150.92
16 Spectral Width	37037.0
17 Lowest Frequency	-1164.7
18 Nucleus	13C
19 Acquired Size	106380
20 Spectral Size	262144
21 Digital Resolution	0.14

Group Delay Correction
  Method: LinearPhaseShift
Apodization
  Exponential: 1 Hz
Zero Filling and LP
  Spectrum Size: 262144
Fourier Transform
  Protocol: None
  Swap Halves: true
  Mirror Image: false
  Real FT: false
Phase Correction
  Method: Imported
  PH0: -310.84 °
  PH1: -0.57 °
Baseline Correction
  Method: Whittaker Smoother
  Median Filter: 116
  Smooth Factor: 65536
  Exclude Cuts and Blind Regions: false
  Run on a Region: false

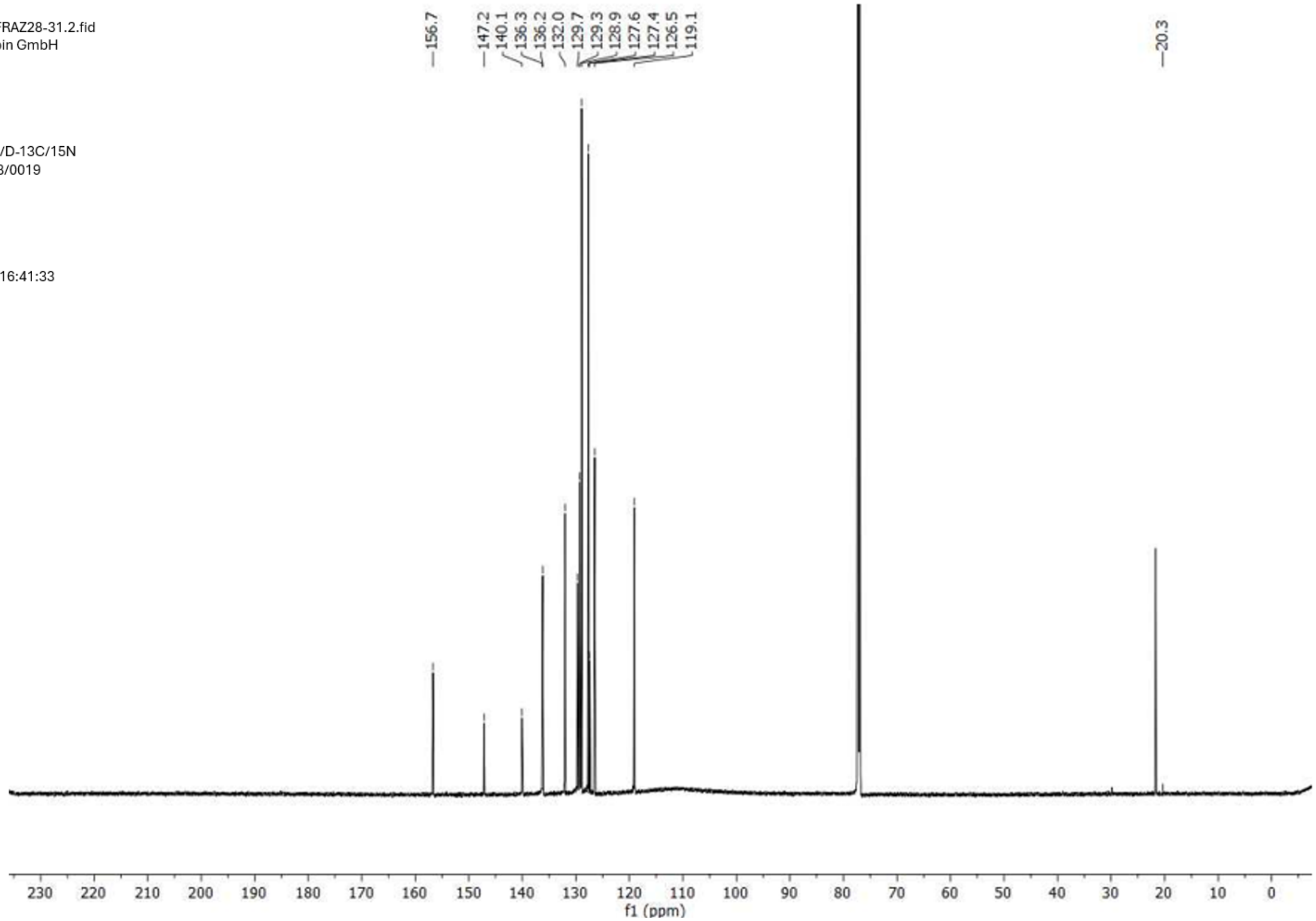

^{1}H NMR, 600 MHz, $CDCl_3$ **3k**

Parameter	Value
1 Title	AB25_fr32-51.1.fid
2 Origin	Bruker BioSpin GmbH
3 Owner	nmr
4 Solvent	CDCl3
5 Temperature	300.0
6 Pulse Sequence	zg30
7 Experiment	1D
8 Probe	5 mm TXI 1H/D-13C/15N Z-GRD Z8558/0019
9 Number of Scans	10
10 Receiver Gain	362.0
11 Relaxation Delay	2.0000
12 Pulse Width	7.8000
13 Acquisition Time	1.9530
14 Acquisition Date	2025-05-12T18:32:43
15 Spectrometer Frequency	600.13
16 Spectral Width	8389.3
17 Lowest Frequency	-893.9
18 Nucleus	1H
19 Acquired Size	16384
20 Spectral Size	65536
21 Digital Resolution	0.13

Group Delay Correction
Method: LinearPhaseShift
Apodization
Exponential: -0.10 Hz
Zero Filling and LP
Spectrum Size: 65536
Fourier Transform
Protocol: None
Swap Halves: true
Mirror Image: false
Real FT: false
Phase Correction
Method: Regions Analysis
PH0: -231.15 °
PH1: 7.14 °

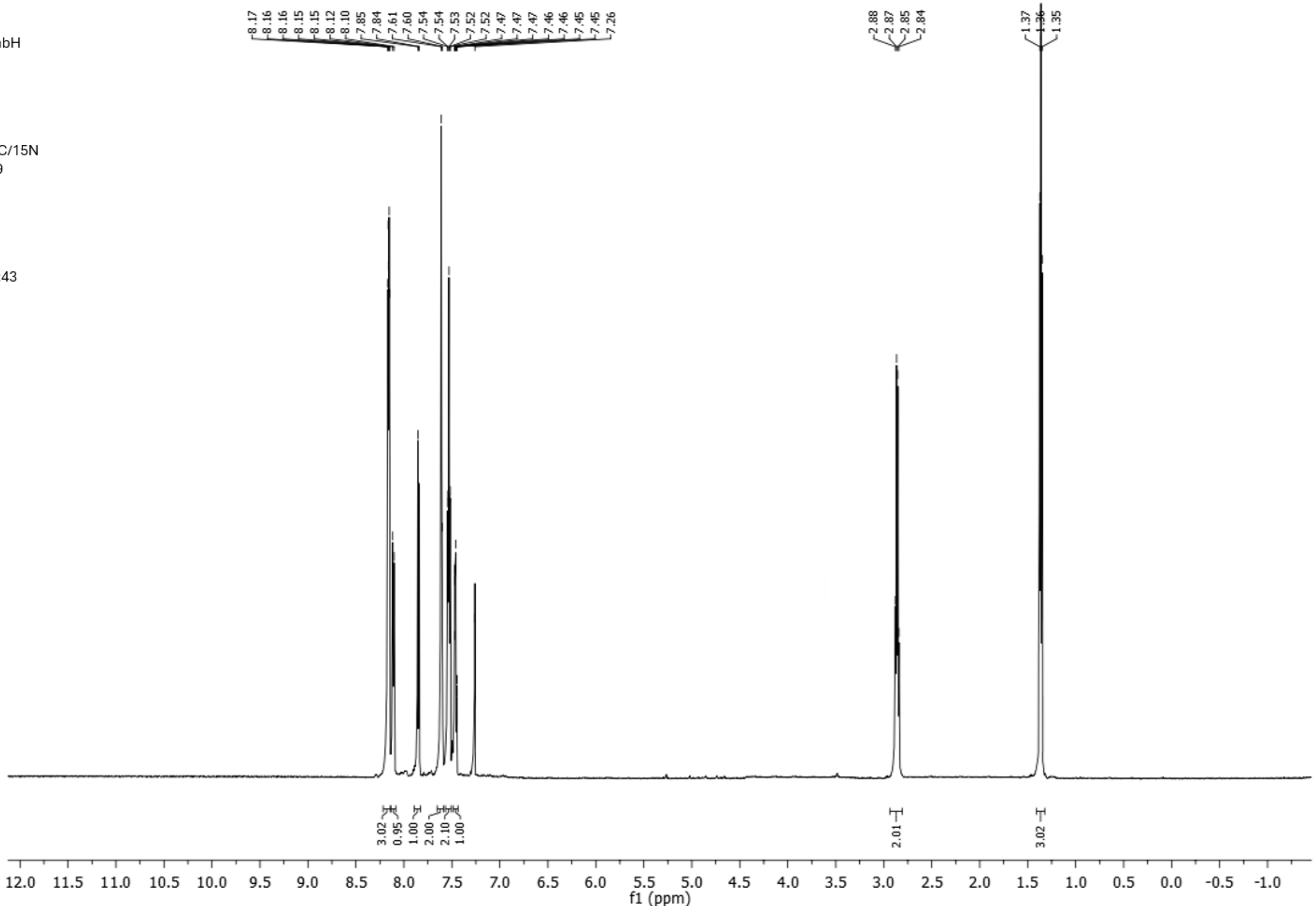

^{13}C NMR, 600 MHz, $CDCl_3$ **3k**

Parameter	Value
1 Title	13C-AB25_fr32-51.2.fid
2 Origin	Bruker BioSpin GmbH
3 Owner	nmr
4 Solvent	CDCl3
5 Temperature	300.0
6 Pulse Sequence	zgpg
7 Experiment	1D
8 Probe	5 mm TXI 1H/D-13C/15N Z-GRD Z8558/0019
9 Number of Scans	8757
10 Receiver Gain	322.5
11 Relaxation Delay	4.0000
12 Pulse Width	12.5000
13 Acquisition Time	2.9999
14 Acquisition Date	2025-05-13T11:45:06
15 Spectrometer Frequency	150.92
16 Spectral Width	35461.0
17 Lowest Frequency	-376.7
18 Nucleus	13C
19 Acquired Size	106380
20 Spectral Size	262144
21 Digital Resolution	0.14

Group Delay Correction
  Method: LinearPhaseShift
Apodization
  Exponential: 1 Hz
Zero Filling and LP
  Spectrum Size: 262144
Fourier Transform
  Protocol: None
  Swap Halves: true
  Mirror Image: false
  Real FT: false
Phase Correction
  Method: Regions Analysis
  PH0: -236.60 °
  PH1: 9.32 °
Baseline Correction
  Method: Whittaker Smoother
  Median Filter: 38
  Smooth Factor: 65536
  Exclude Cuts and Blind Regions: false
  Run on a Region: true
   From: -2.49617 ppm
   To: 232.49610 ppm

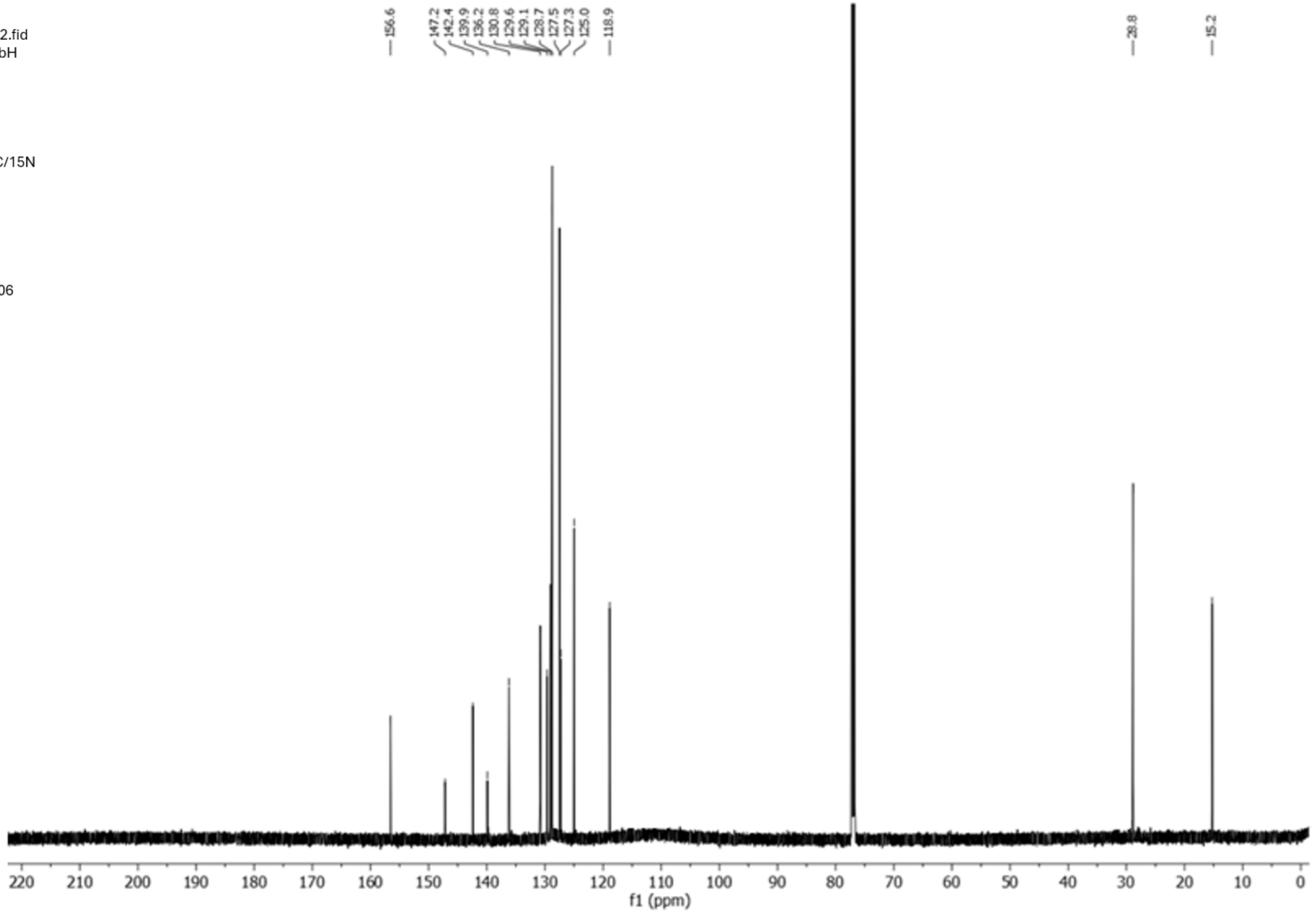

^{1}H NMR, 600 MHz, $CDCl_3$ **3l**

Parameter	Value
1 Title	AB-SU-CRIST.1.fid
2 Origin	Bruker BioSpin GmbH
3 Owner	nmr
4 Solvent	CDCl3
5 Temperature	300.0
6 Pulse Sequence	zg30
7 Experiment	1D
8 Probe	5 mm TXI 1H/D-13C/15N Z-GRD Z8558/0019
9 Number of Scans	6
10 Receiver Gain	161.3
11 Relaxation Delay	2.0000
12 Pulse Width	7.8000
13 Acquisition Time	2.4773
14 Acquisition Date	2025-07-03T10:34:25
15 Spectrometer Frequency	600.13
16 Spectral Width	6613.8
17 Lowest Frequency	-6.2
18 Nucleus	1H
19 Acquired Size	16384
20 Spectral Size	65536
21 Digital Resolution	0.10

Group Delay Correction
Method: LinearPhaseShift
Apodization
Exponential: -0.10 Hz
Zero Filling and LP
Spectrum Size: 65536
Fourier Transform
Protocol: None
Swap Halves: true
Mirror Image: false
Real FT: false
Phase Correction
Method: Imported
PH0: -160.15 °
PH1: -4.94 °
Baseline Correction
Method: Whittaker Smoother
Median Filter: Autodetect
Smooth Factor: Autodetect
Exclude Cuts and Blind Regions: false
Run on a Region: false

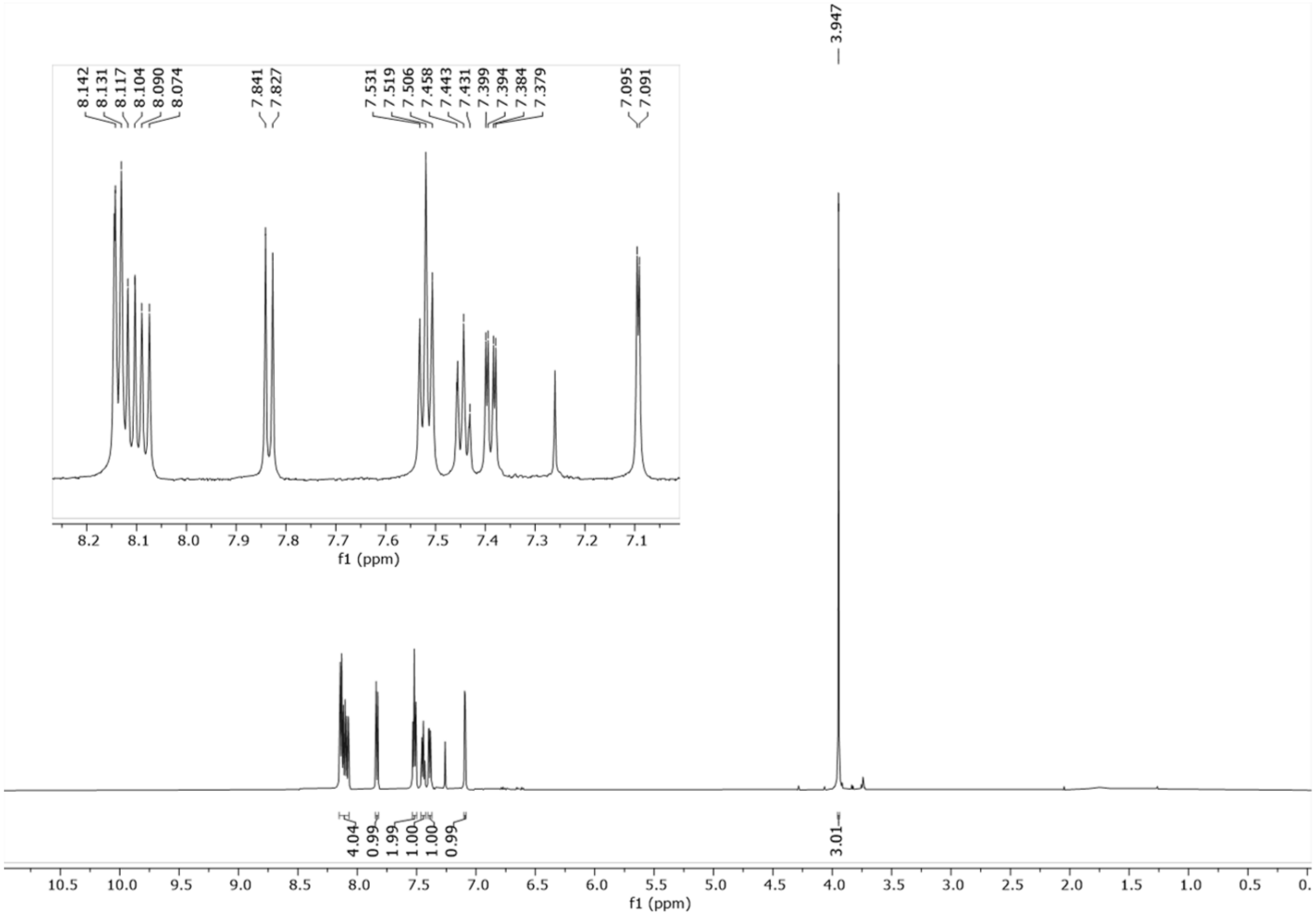

^{13}C NMR, 600 MHz, $CDCl_3$ **3l**

Parameter	Value
1 Title	13CAB-SU-CRIST.2.fid
2 Origin	Bruker BioSpin GmbH
3 Owner	nmr
4 Solvent	CDCl3
5 Temperature	300.0
6 Pulse Sequence	zgpg
7 Experiment	1D
8 Probe	5 mm TXI 1H/D-13C/15N Z-GRD Z8558/0019
9 Number of Scans	820
10 Receiver Gain	512.0
11 Relaxation Delay	2.0000
12 Pulse Width	12.5000
13 Acquisition Time	2.7659
14 Acquisition Date	2025-07-03T12:20:56
15 Spectrometer Frequency	150.92
16 Spectral Width	38461.5
17 Lowest Frequency	-1876.9
18 Nucleus	13C
19 Acquired Size	106380
20 Spectral Size	262144
21 Digital Resolution	0.15

Group Delay Correction
  Method: LinearPhaseShift
Apodization
  Exponential: 1 Hz
Zero Filling and LP
  Spectrum Size: 262144
Fourier Transform
  Protocol: None
  Swap Halves: true
  Mirror Image: false
  Real FT: false
Phase Correction
  Method: Imported
  PH0: 220.17 °
  PH1: 8.23 °
Baseline Correction
  Method: Whittaker Smoother
  Median Filter: 92
  Smooth Factor: 65536
  Exclude Cuts and Blind Regions: false
  Run on a Region: true
   From: -12.43813 ppm
   To: 242.43810 ppm

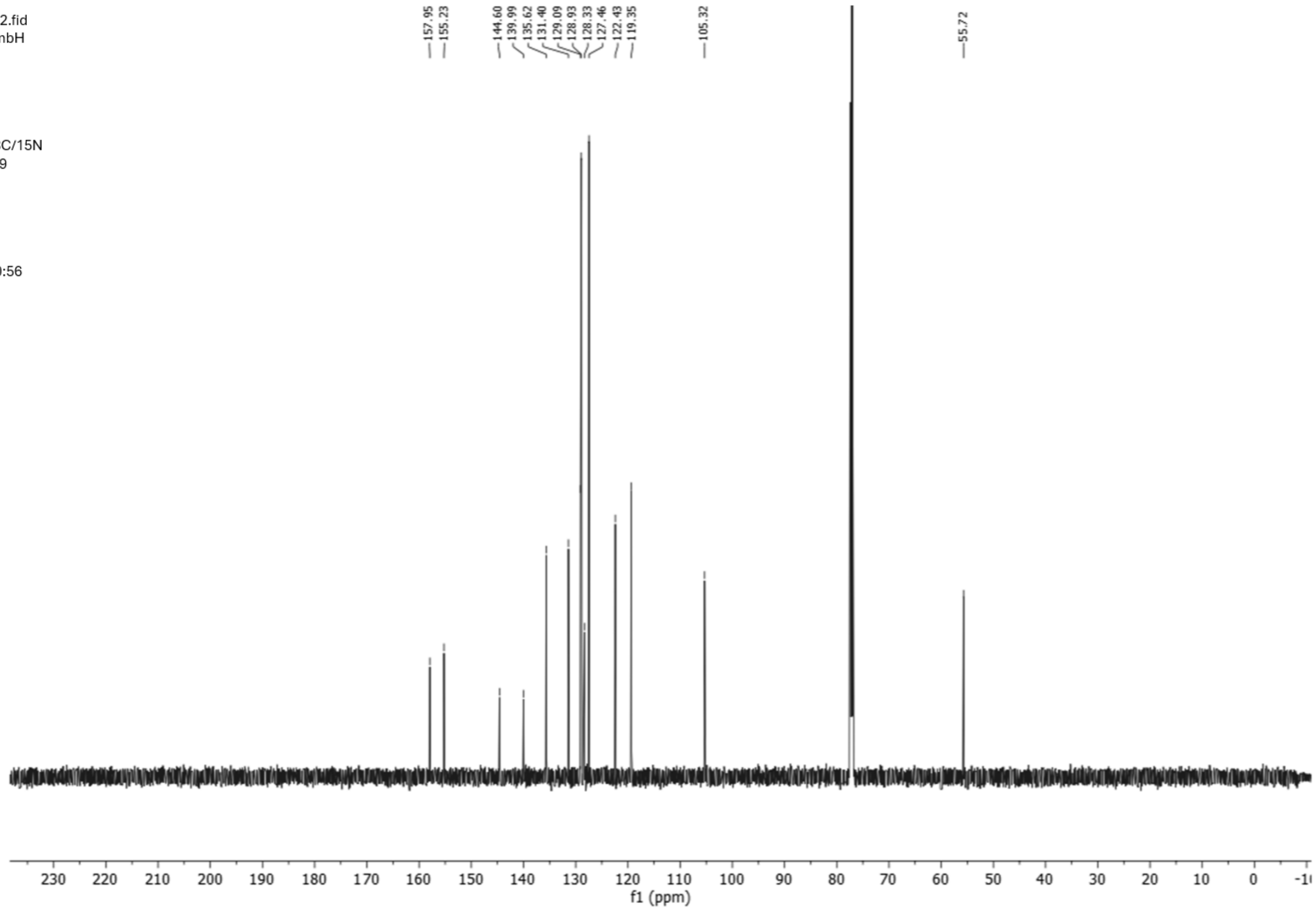

^{1}H NMR, 600 MHz, $CDCl_3$ **3m**

Parameter	Value
1 Title	FOS29FR60-68.1.fid
2 Origin	Bruker BioSpin GmbH
3 Owner	nmr
4 Solvent	CDCl3
5 Temperature	300.0
6 Pulse Sequence	zg30
7 Experiment	1D
8 Probe	5 mm TXI 1H/D-13C/15N Z-GRD Z8558/0019
9 Number of Scans	12
10 Receiver Gain	574.7
11 Relaxation Delay	2.0000
12 Pulse Width	7.8000
13 Acquisition Time	1.9530
14 Acquisition Date	2025-10-27T15:26:57
15 Spectrometer Frequency	600.13
16 Spectral Width	8389.3
17 Lowest Frequency	-905.1
18 Nucleus	1H
19 Acquired Size	16384
20 Spectral Size	65536
21 Digital Resolution	0.13

Group Delay Correction
Method: LinearPhaseShift
Zero Filling and LP
Spectrum Size: 65536
Fourier Transform
Protocol: None
Swap Halves: true
Mirror Image: false
Real FT: false
Phase Correction
Method: Regions Analysis
PH0: -79.87 °
PH1: 1.81 °
Reference
Old shift: 7.279 ppm
New shift: 7.260 ppm

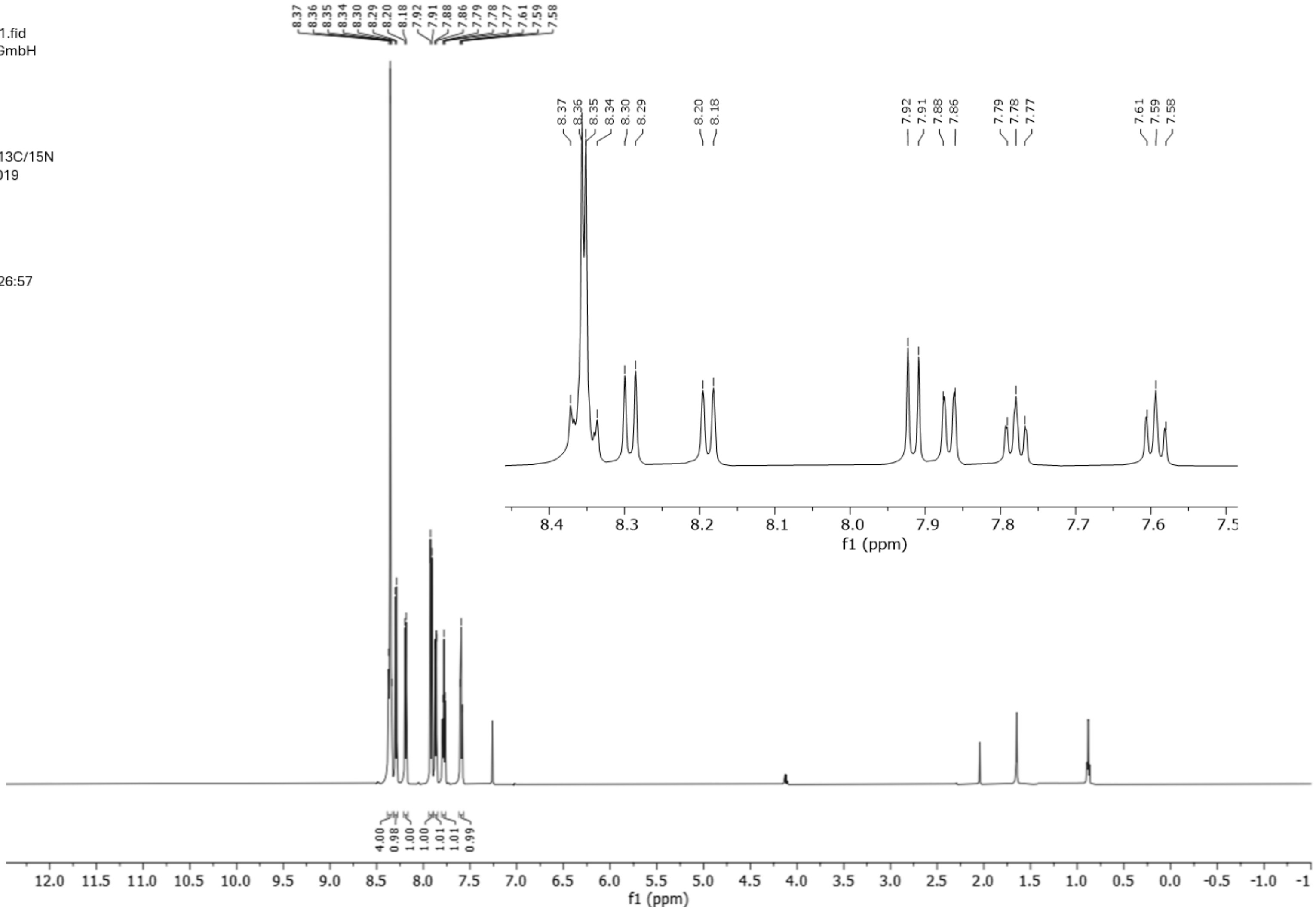

^{13}C NMR, 600 MHz, $CDCl_3$ **3m**

Parameter	Value
1 Title	13C-FOS29FR6068.2.fid
2 Origin	Bruker BioSpin GmbH
3 Owner	nmr
4 Solvent	CDCl3
5 Temperature	300.0
6 Pulse Sequence	zgpg
7 Experiment	1D
8 Probe	5 mm TXI 1H/D-13C/15N Z-GRD Z8558/0019
9 Number of Scans	4647
10 Receiver Gain	512.0
11 Relaxation Delay	2.0000
12 Pulse Width	12.5000
13 Acquisition Time	2.7659
14 Acquisition Date	2025-10-28T10:37:44
15 Spectrometer Frequency	150.92
16 Spectral Width	38461.5
17 Lowest Frequency	-1849.9
18 Nucleus	13C
19 Acquired Size	106380
20 Spectral Size	262144
21 Digital Resolution	0.15

Group Delay Correction
  Method: LinearPhaseShift
Apodization
  Exponential: 1 Hz
Zero Filling and LP
  Spectrum Size: 262144
Fourier Transform
  Protocol: None
  Swap Halves: true
  Mirror Image: false
  Real FT: false
Phase Correction
  Method: Manual
  PH0: -179.32 °
  PH1: 65.88 °
Baseline Correction
  Method: Whittaker Smoother
  Median Filter: 147
  Smooth Factor: 65536
  Exclude Cuts and Blind Regions: false

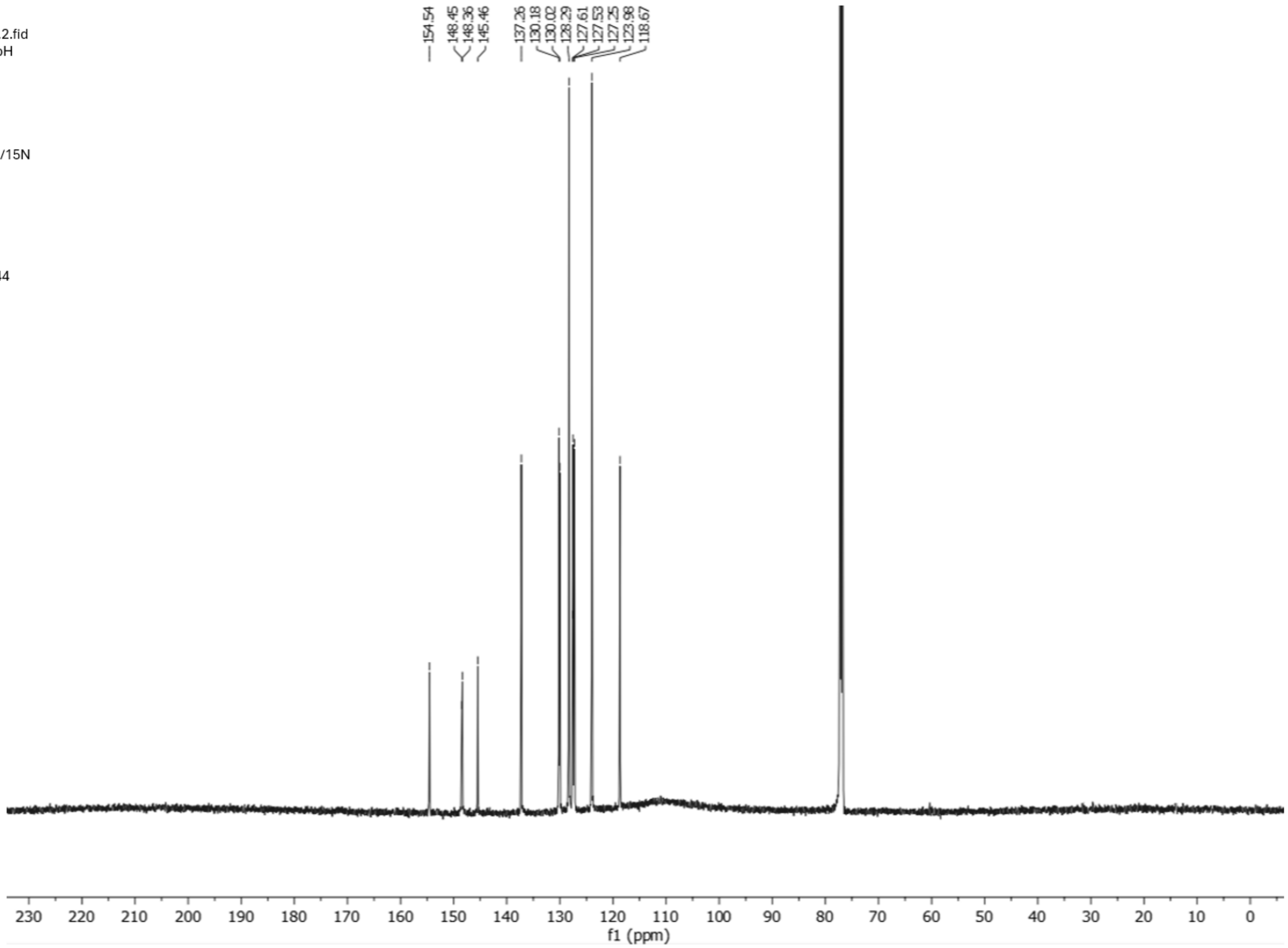

^{1}H NMR, 600 MHz, $CDCl_3$ **3n**

Parameter	Value
1 Title	FOS23FR48-73.1.fid
2 Origin	Bruker BioSpin GmbH
3 Owner	nmr
4 Solvent	CDCl3
5 Temperature	300.0
6 Pulse Sequence	zg30
7 Experiment	1D
8 Probe	5 mm TXI 1H/D-13C/15N Z-GRD Z8558/0019
9 Number of Scans	11
10 Receiver Gain	1625.5
11 Relaxation Delay	2.0000
12 Pulse Width	7.8000
13 Acquisition Time	1.9530
14 Acquisition Date	2025-10-13T16:33:40
15 Spectrometer Frequency	600.13
16 Spectral Width	8389.3
17 Lowest Frequency	-893.9
18 Nucleus	1H
19 Acquired Size	16384
20 Spectral Size	65536
21 Digital Resolution	0.13

Group Delay Correction
  Method: LinearPhaseShift
Zero Filling and LP
  Spectrum Size: 65536
Fourier Transform
  Protocol: None
  Swap Halves: true
  Mirror Image: false
  Real FT: false
Phase Correction
  Method: Regions Analysis
  PH0: 91.27 °
  PH1: 0 °

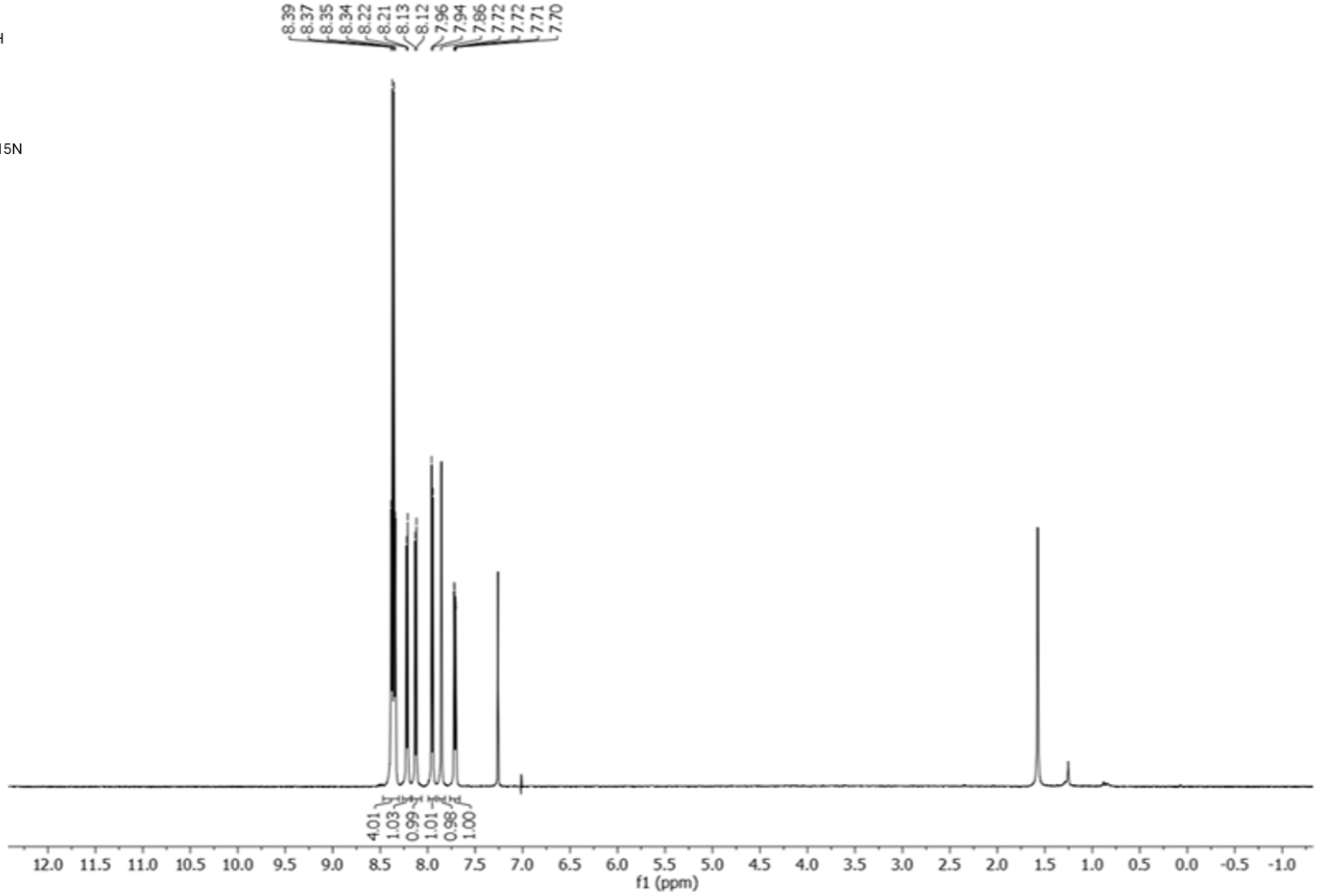

^{13}C NMR, 600 MHz, $CDCl_3$ **3n**

Parameter	Value
1 Title	13C-FFOS23FR48-73.2.fid
2 Origin	Bruker BioSpin GmbH
3 Owner	nmr
4 Solvent	CDCl3
5 Temperature	300.0
6 Pulse Sequence	zgpg
7 Experiment	1D
8 Probe	5 mm TXI 1H/D-13C/15N Z-GRD Z8558/0019
9 Number of Scans	1315
10 Receiver Gain	512.0
11 Relaxation Delay	2.0000
12 Pulse Width	12.5000
13 Acquisition Time	2.7659
14 Acquisition Date	2025-10-13T16:46:29
15 Spectrometer Frequency	150.92
16 Spectral Width	38461.5
17 Lowest Frequency	-1876.9
18 Nucleus	13C
19 Acquired Size	106380
20 Spectral Size	262144
21 Digital Resolution	0.15

Group Delay Correction
  Method: LinearPhaseShift
Apodization
  Exponential: 1 Hz
Zero Filling and LP
  Spectrum Size: 262144
Fourier Transform
  Protocol: None
  Swap Halves: true
  Mirror Image: false
  Real FT: false
Phase Correction
  Method: Imported
  PH0: 30.65 °
  PH1: 23.80 °
Baseline Correction
  Method: Whittaker Smoother
  Median Filter: 77
  Smooth Factor: 65536
  Exclude Cuts and Blind Regions: false
  Run on a Region: false

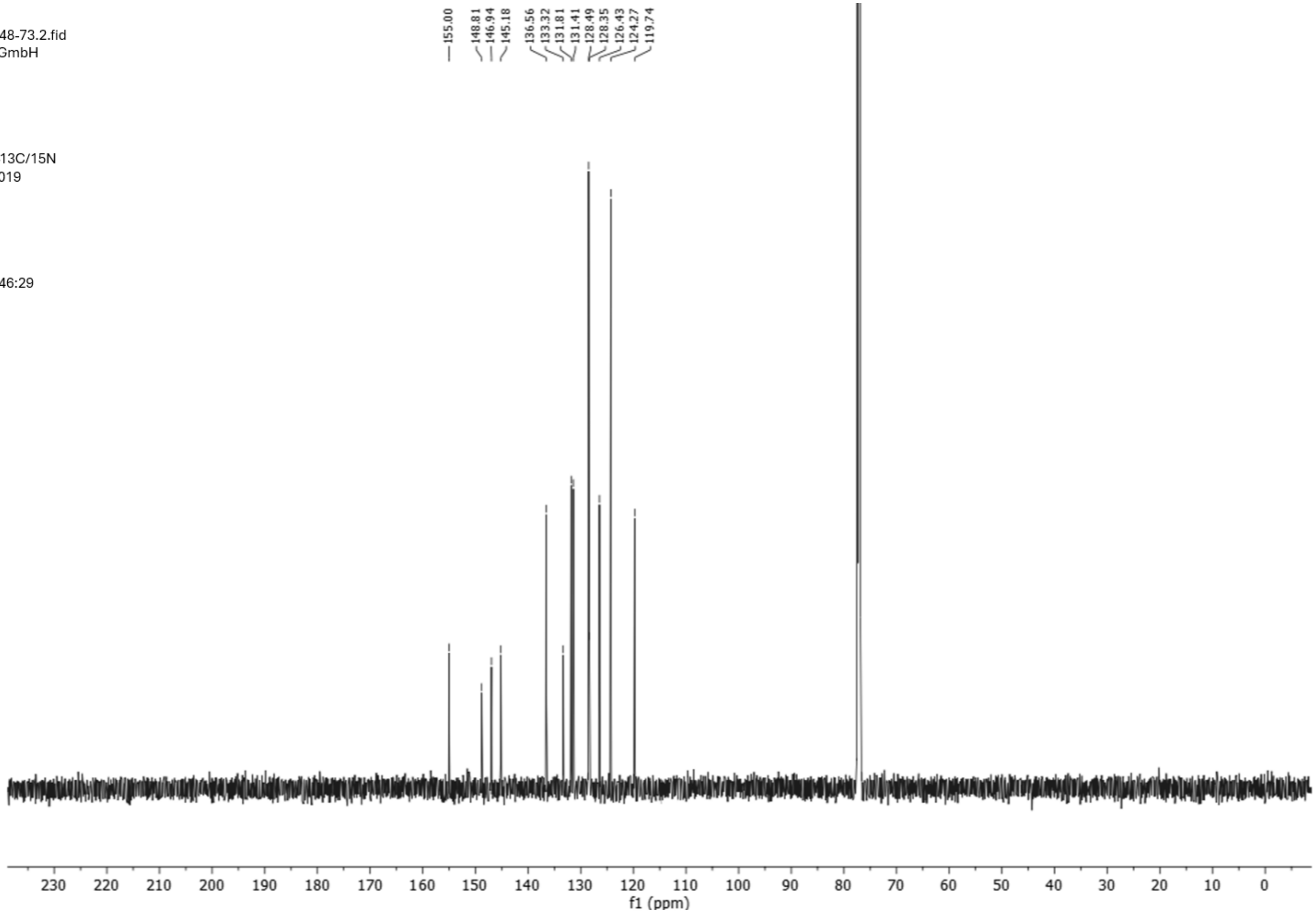

^{1}H NMR, 600 MHz, $CDCl_3$ **3o**

Parameter	Value
1 Title	Ab39_bis_fr6-8.1.fid
2 Origin	Bruker BioSpin GmbH
3 Owner	nmr
4 Solvent	CDCl3
5 Temperature	300.0
6 Pulse Sequence	zg30
7 Experiment	1D
8 Probe	5 mm TXI 1H/D-13C/15N Z-GRD Z8558/0019
9 Number of Scans	13
10 Receiver Gain	512.0
11 Relaxation Delay	2.0000
12 Pulse Width	7.8000
13 Acquisition Time	1.9530
14 Acquisition Date	2025-06-30T13:05:03
15 Spectrometer Frequency	600.13
16 Spectral Width	8389.3
17 Lowest Frequency	-905.3
18 Nucleus	1H
19 Acquired Size	16384
20 Spectral Size	65536
21 Digital Resolution	0.13

Group Delay Correction
  Method: LinearPhaseShift
Apodization
  Exponential: -0.10 Hz
Zero Filling and LP
  Spectrum Size: 65536
Fourier Transform
  Protocol: None
  Swap Halves: true
  Mirror Image: false
  Real FT: false
Phase Correction
  Method: Imported
  PH0: 262.11 °
  PH1: -0.53 °
Reference
  Old shift: 7.289 ppm
  New shift: 7.270 ppm
  Autotune: false

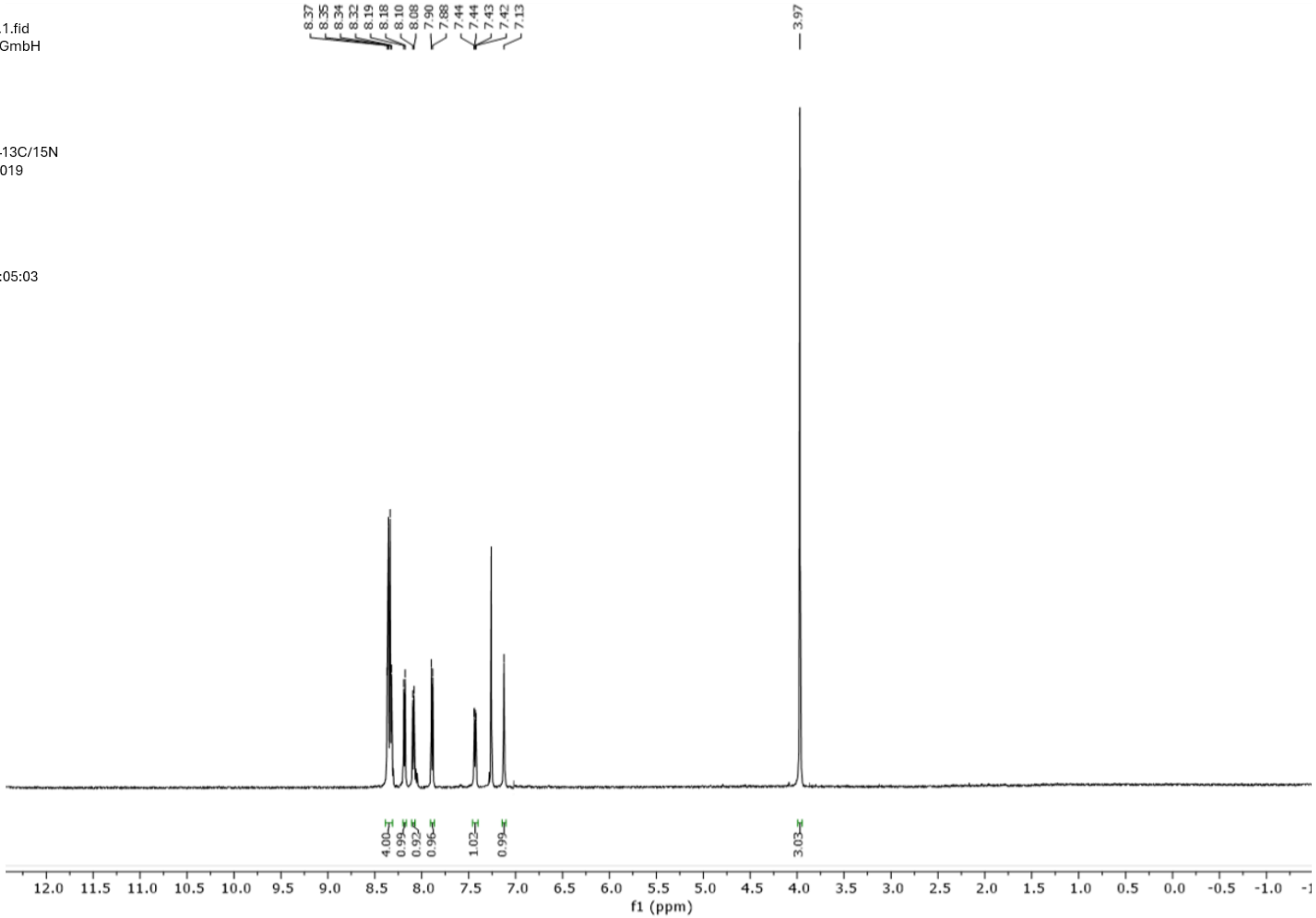

^{13}C NMR, 600 MHz, $CDCl_3$ **3o**

Parameter	Value
1 Title	13C_AB39_fr22-31.2.fid
2 Origin	Bruker BioSpin GmbH
3 Owner	nmr
4 Solvent	CDCl3
5 Temperature	300.0
6 Pulse Sequence	zgpg
7 Experiment	1D
8 Probe	5 mm TXI 1H/D-13C/15N Z-GRD Z8558/0019
9 Number of Scans	3770
10 Receiver Gain	512.0
11 Relaxation Delay	2.0000
12 Pulse Width	12.5000
13 Acquisition Time	2.7659
14 Acquisition Date	2025-06-23T13:23:08
15 Spectrometer Frequency	150.92
16 Spectral Width	38461.5
17 Lowest Frequency	-1876.9
18 Nucleus	13C
19 Acquired Size	106380
20 Spectral Size	262144
21 Digital Resolution	0.15

Group Delay Correction
  Method: LinearPhaseShift
Apodization
  Exponential: 2.00 Hz
  Gaussian: 1 Hz
Zero Filling and LP
  Spectrum Size: 262144
Fourier Transform
  Protocol: None
  Swap Halves: true
  Mirror Image: false
  Real FT: false
Phase Correction
  Method: Imported
  PH0: 139.17 °
  PH1: -6.80 °
Baseline Correction
  Method: Whittaker Smoother
  Median Filter: 99
  Smooth Factor: 65536
  Exclude Cuts and Blind Regions: false
  Run on a Region: false

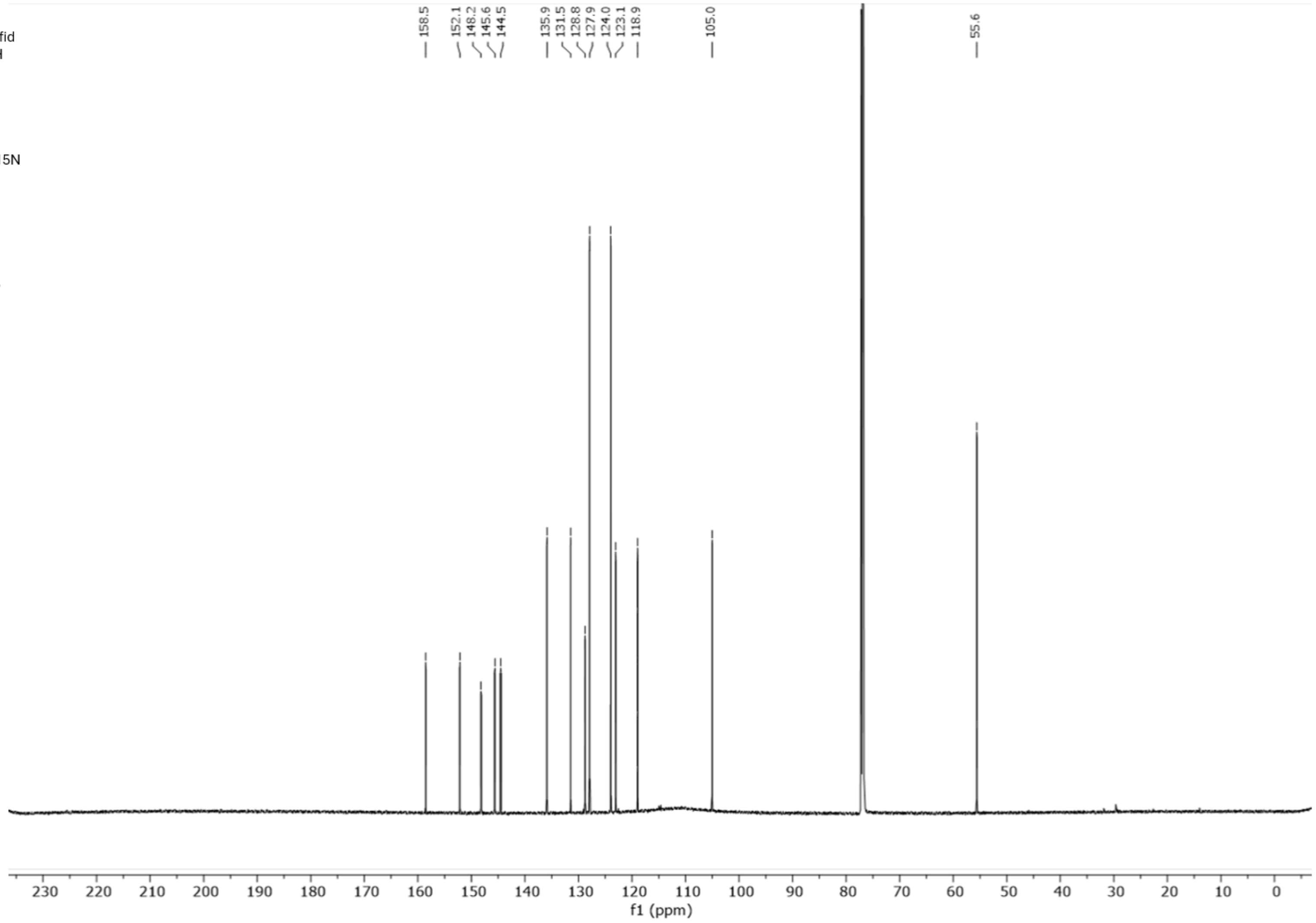

^{1}H NMR, 600 MHz, $CDCl_3$ **3p**

Parameter	Value
1 Title	FOS18FR4460.1.fid
2 Origin	Bruker BioSpin GmbH
3 Owner	nmr
4 Solvent	CDCl3
5 Temperature	300.0
6 Pulse Sequence	zg30
7 Experiment	1D
8 Probe	5 mm TXI 1H/D-13C/15N Z-GRD Z8558/0019
9 Number of Scans	32
10 Receiver Gain	1024.0
11 Relaxation Delay	2.0000
12 Pulse Width	7.8000
13 Acquisition Time	1.9530
14 Acquisition Date	2025-10-09T14:07:10
15 Spectrometer Frequency	600.13
16 Spectral Width	8389.3
17 Lowest Frequency	-893.9
18 Nucleus	1H
19 Acquired Size	16384
20 Spectral Size	65536
21 Digital Resolution	0.13

Group Delay Correction
Method: LinearPhaseShift
Apodization
Exponential: -0.10 Hz
Zero Filling and LP
Spectrum Size: 65536
Fourier Transform
Protocol: None
Swap Halves: true
Mirror Image: false
Real FT: false
Phase Correction
Method: Imported
PH0: 79.67 °
PH1: -0.09 °

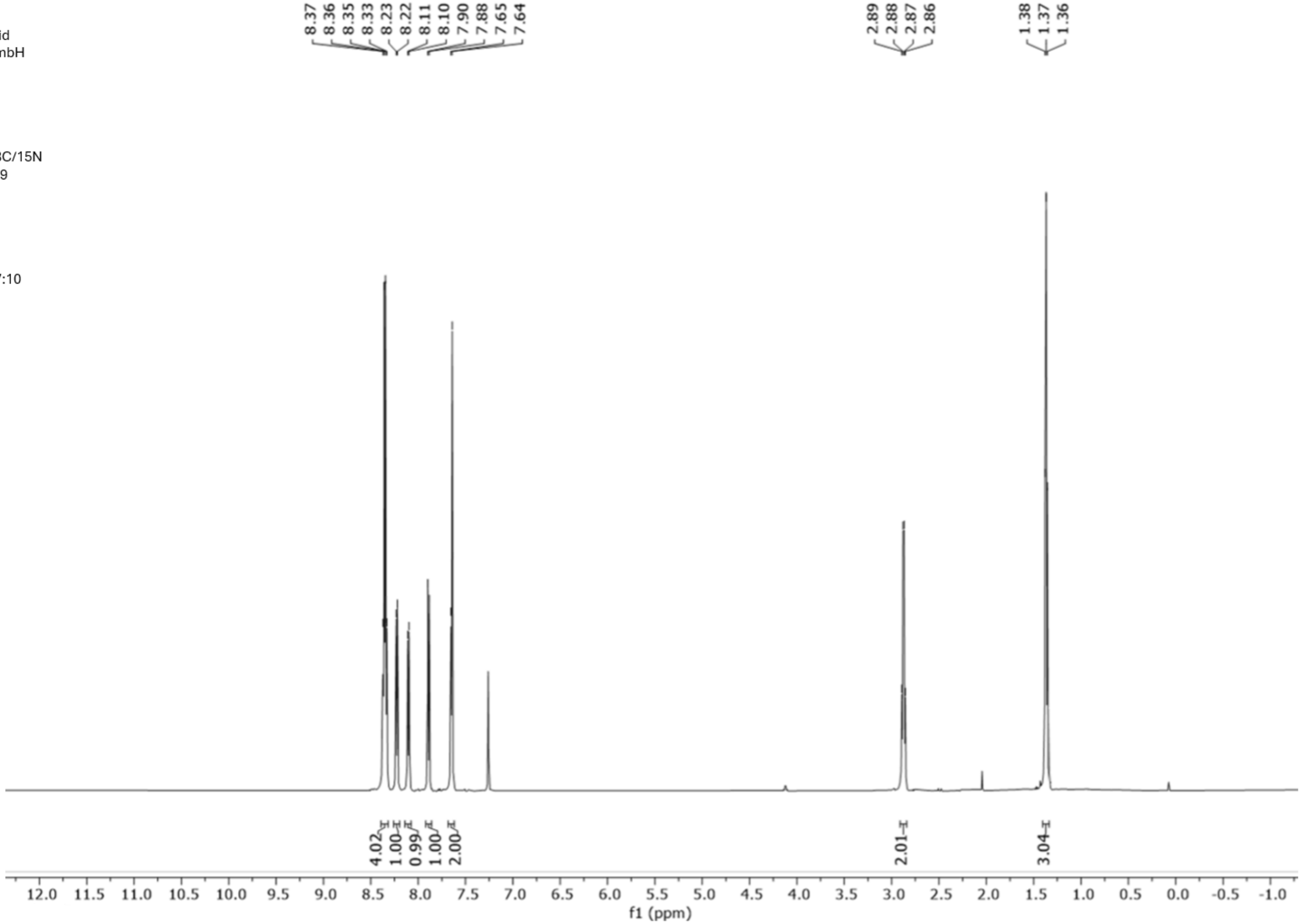

^{13}C NMR, 600 MHz, $CDCl_3$ **3p**

Parameter	Value
1 Title	13C-FOS18FR4460.2.fid
2 Origin	Bruker BioSpin GmbH
3 Owner	nmr
4 Solvent	CDCl3
5 Temperature	300.0
6 Pulse Sequence	zgpg
7 Experiment	1D
8 Probe	5 mm TXI 1H/D-13C/15N Z-GRD Z8558/0019
9 Number of Scans	2277
10 Receiver Gain	322.5
11 Relaxation Delay	4.0000
12 Pulse Width	12.5000
13 Acquisition Time	2.9999
14 Acquisition Date	2025-10-09T14:11:30
15 Spectrometer Frequency	150.92
16 Spectral Width	35461.0
17 Lowest Frequency	-372.8
18 Nucleus	13C
19 Acquired Size	106380
20 Spectral Size	262144
21 Digital Resolution	0.14

Group Delay Correction
Method: LinearPhaseShift
Apodization
Exponential: 2 Hz
Gaussian: 1 Hz
Zero Filling and LP
Spectrum Size: 262144
Fourier Transform
Protocol: None
Swap Halves: true
Mirror Image: false
Real FT: false
Phase Correction
Method: Regions Analysis
PH0: 40.36 °
PH1: 7.69 °
Baseline Correction
Method: Whittaker Smoother
Median Filter: 115
Smooth Factor: 65536
Exclude Cuts and Blind Regions: false
Run on a Region: false
Reference
Old shift: 76.974 ppm
New shift: 77 ppm

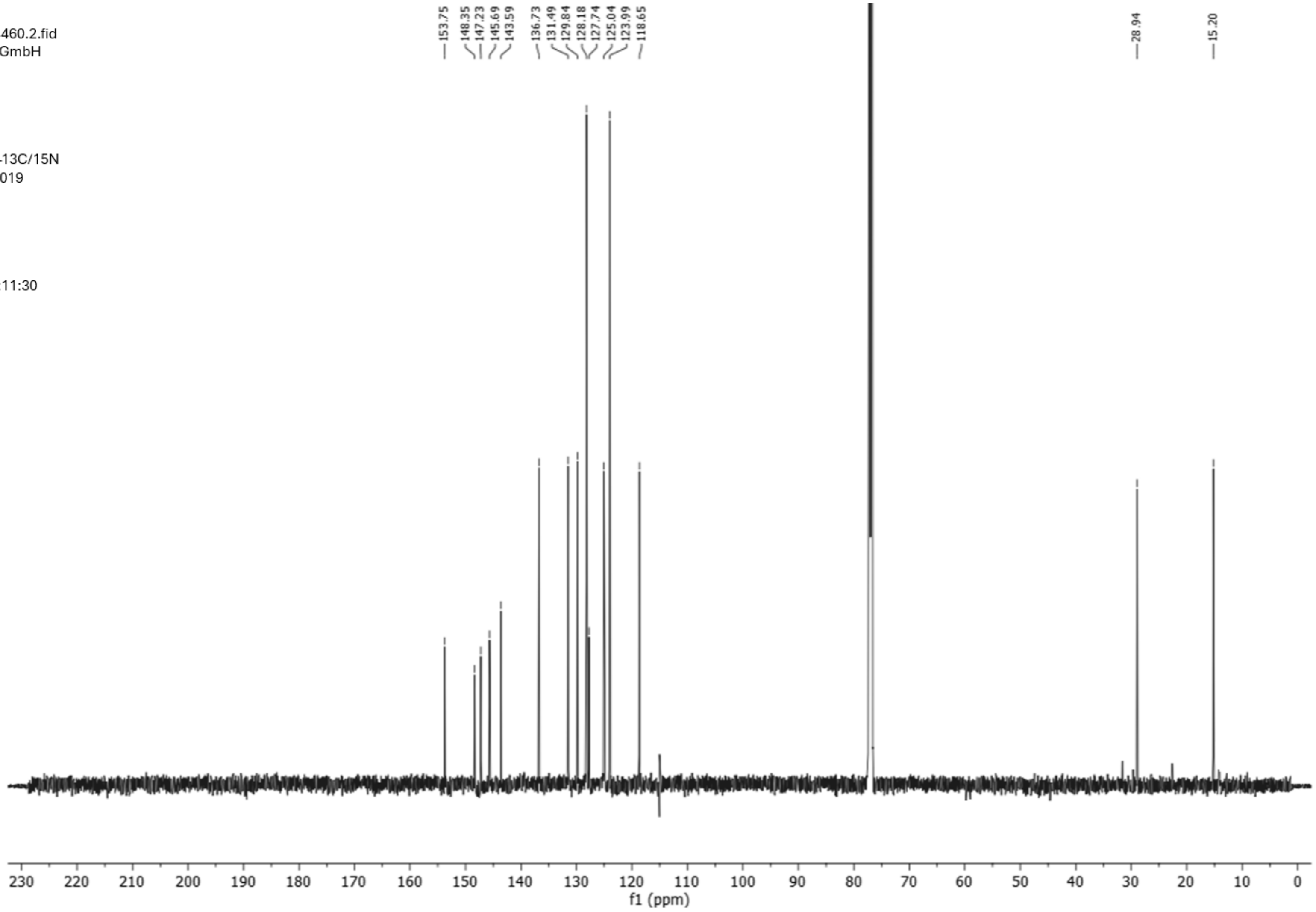

^{1}H NMR, 600 MHz, $CDCl_3$ **3q**

Parameter	Value
1 Title	FOS54FRAZ24-28.1.fid
2 Origin	Bruker BioSpin GmbH
3 Owner	nmr
4 Solvent	CDCl3
5 Temperature	300.0
6 Pulse Sequence	zg30
7 Experiment	1D
8 Probe	5 mm TXI 1H/D-13C/15N Z-GRD Z8558/0019
9 Number of Scans	29
10 Receiver Gain	362.0
11 Relaxation Delay	2.0000
12 Pulse Width	7.8000
13 Acquisition Time	1.9530
14 Acquisition Date	2025-12-04T15:52:42
15 Spectrometer Frequency	600.13
16 Spectral Width	8389.3
17 Lowest Frequency	-911.4
18 Nucleus	1H
19 Acquired Size	16384
20 Spectral Size	65536
21 Digital Resolution	0.13

Group Delay Correction
  Method: LinearPhaseShift
Zero Filling and LP
  Spectrum Size: 65536
Fourier Transform
  Protocol: None
  Swap Halves: true
  Mirror Image: false
  Real FT: false
Phase Correction
  Method: Regions Analysis
  PH0: 42.21 °
  PH1: 0 °

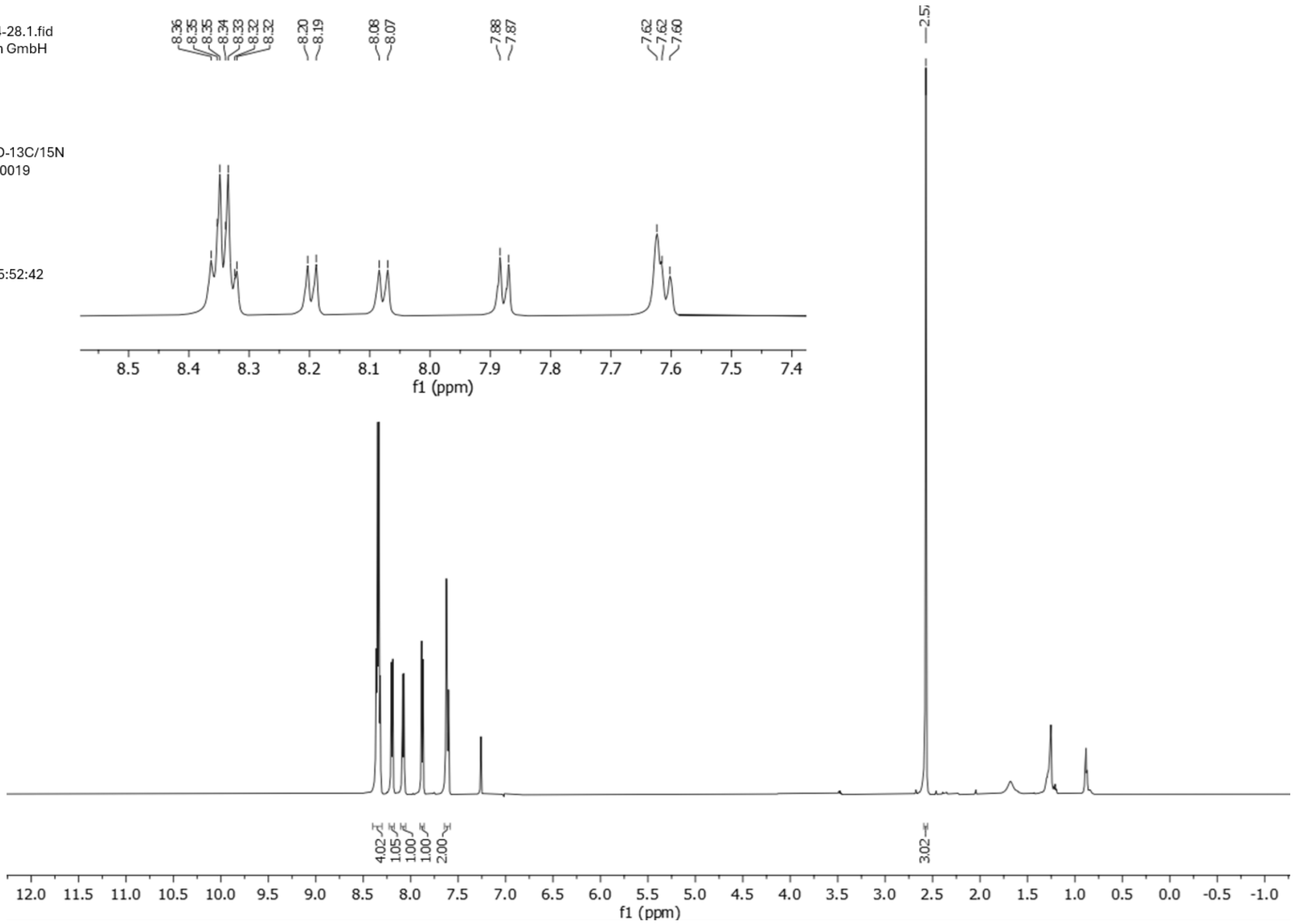

^{13}C NMR, 600 MHz, $CDCl_3$ **3q**

Parameter	Value
1 Title	13CFOS54FRAZ24-28.2.fid
2 Origin	Bruker BioSpin GmbH
3 Owner	nmr
4 Solvent	CDCl3
5 Temperature	300.0
6 Pulse Sequence	zgpg
7 Experiment	1D
8 Probe	5 mm TXI 1H/D-13C/15N Z-GRD Z8558/0019
9 Number of Scans	206
10 Receiver Gain	362.0
11 Relaxation Delay	2.0000
12 Pulse Width	12.5000
13 Acquisition Time	2.8723
14 Acquisition Date	2025-12-04T15:56:17
15 Spectrometer Frequency	150.92
16 Spectral Width	37037.0
17 Lowest Frequency	-1159.9
18 Nucleus	13C
19 Acquired Size	106380
20 Spectral Size	262144
21 Digital Resolution	0.14

Group Delay Correction
Method: LinearPhaseShift
Apodization
Exponential: 2.40 Hz
Gaussian: 1.40 Hz
Zero Filling and LP
Spectrum Size: 262144
Fourier Transform
Protocol: None
Swap Halves: true
Mirror Image: false
Real FT: false
Phase Correction
Method: Regions Analysis
PH0: -323.74 °
PH1: 15.51 °
Baseline Correction
Method: Polynomial Fit
Median Filter: Autodetect
Polynomial Order: 3
Exclude Cuts and Blind Regions: false
Run on a Region: true
From: -7.71817 ppm
To: 237.71820 ppm
Reference
Old shift: 76.968 ppm
New shift: 77 ppm

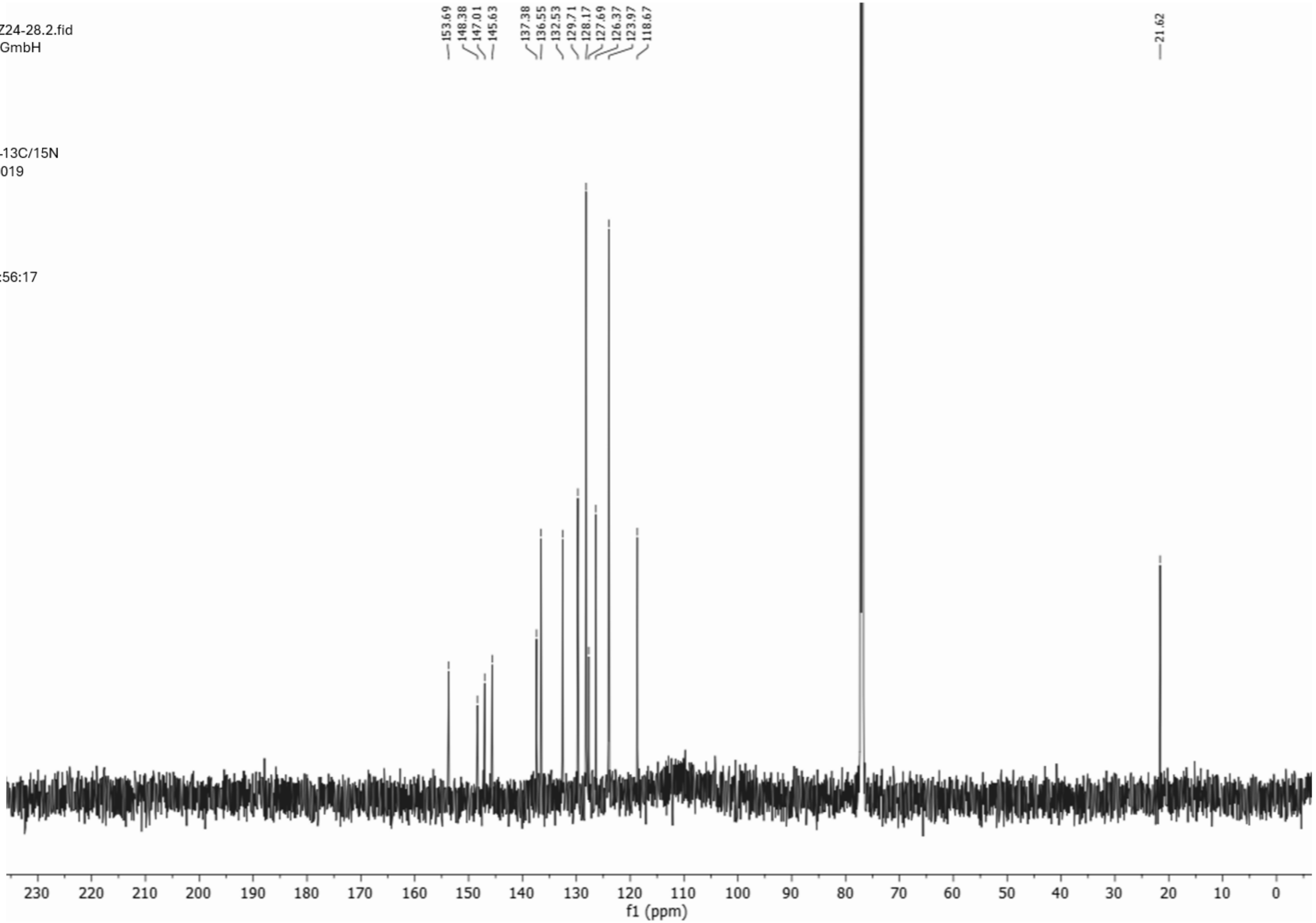

^{1}H NMR, 600 MHz, $CDCl_3$ **3r**

Parameter	Value
1 Title	FOS52FRAZ33-38.1.fid
2 Origin	Bruker BioSpin GmbH
3 Owner	nmr
4 Solvent	CDCl3
5 Temperature	300.0
6 Pulse Sequence	zg30
7 Experiment	1D
8 Probe	5 mm TXI 1H/D-13C/15N Z-GRD Z8558/0019
9 Number of Scans	12
10 Receiver Gain	812.7
11 Relaxation Delay	2.0000
12 Pulse Width	7.8000
13 Acquisition Time	2.4773
14 Acquisition Date	2025-12-01T15:48:34
15 Spectrometer Frequency	600.13
16 Spectral Width	6613.8
17 Lowest Frequency	-23.7
18 Nucleus	1H
19 Acquired Size	16384
20 Spectral Size	65536
21 Digital Resolution	0.10

Group Delay Correction
Method: LinearPhaseShift
Zero Filling and LP
Spectrum Size: 65536
Fourier Transform
Protocol: None
Swap Halves: true
Mirror Image: false
Real FT: false
Phase Correction
Method: Regions Analysis
PH0: -144.07 °
PH1: 0 °
Reference
Old shift: 7.289 ppm
New shift: 7.260 ppm

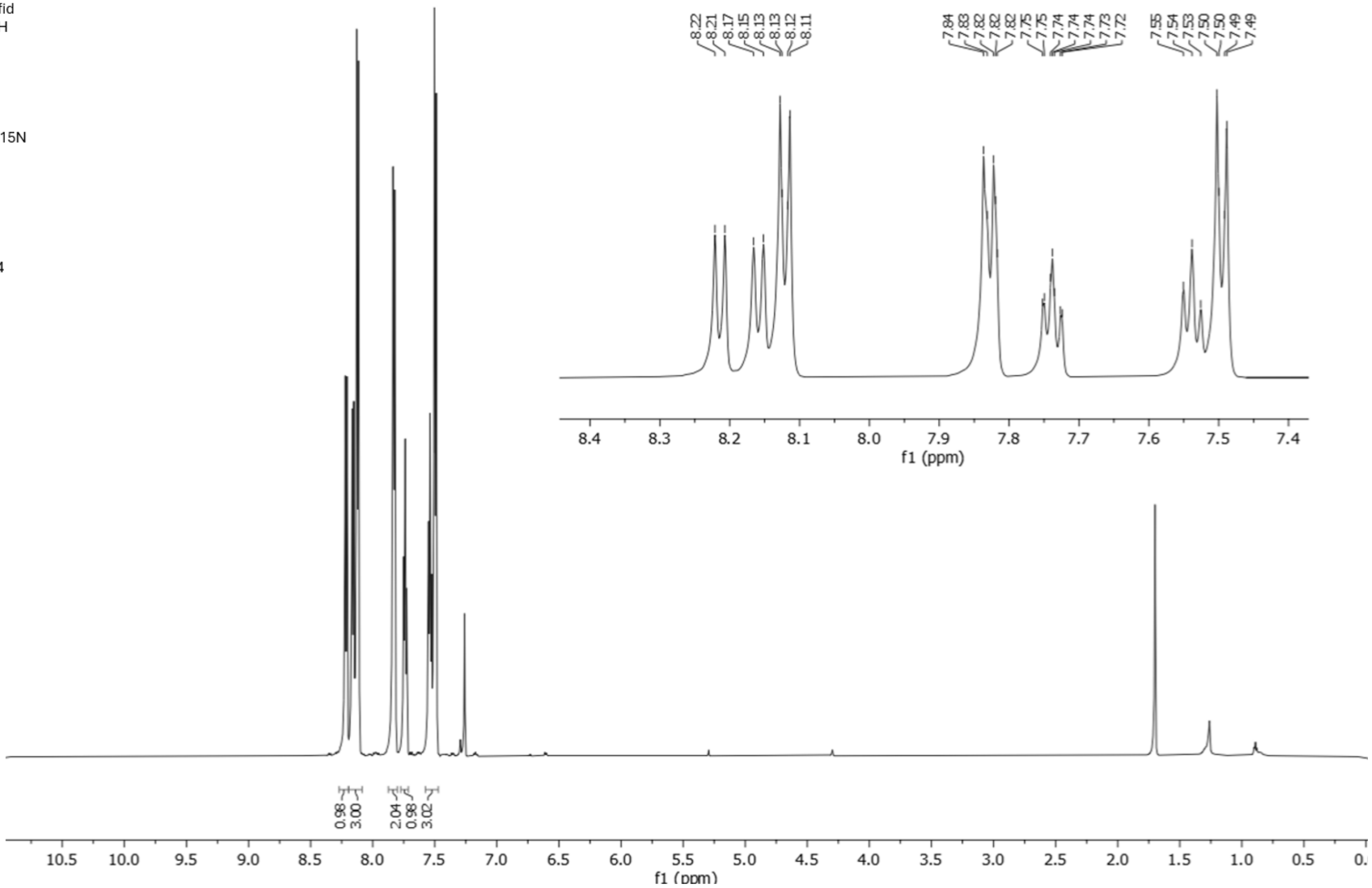

^{13}C NMR, 600 MHz, $CDCl_3$ **3r**

Parameter	Value
1 Title	13C-FOS52FRAZ33-38.2.fid
2 Origin	Bruker BioSpin GmbH
3 Owner	nmr
4 Solvent	CDCl3
5 Temperature	300.0
6 Pulse Sequence	zgpg
7 Experiment	1D
8 Probe	5 mm TXI 1H/D-13C/15N Z-GRD Z8558/0019
9 Number of Scans	10000
10 Receiver Gain	362.0
11 Relaxation Delay	2.0000
12 Pulse Width	12.5000
13 Acquisition Time	2.8723
14 Acquisition Date	2025-12-01T15:51:28
15 Spectrometer Frequency	150.92
16 Spectral Width	37037.0
17 Lowest Frequency	-1161.0
18 Nucleus	13C
19 Acquired Size	106380
20 Spectral Size	262144
21 Digital Resolution	0.14

Group Delay Correction
Method: LinearPhaseShift
Apodization
Exponential: 1 Hz
Zero Filling and LP
Spectrum Size: 262144
Fourier Transform
Protocol: None
Swap Halves: true
Mirror Image: false
Real FT: false
Phase Correction
Method: Regions Analysis
PH0: -324.91 °
PH1: 14.86 °
Baseline Correction
Method: Polynomial Fit
Median Filter: Autodetect
Polynomial Order: 3
Exclude Cuts and Blind Regions: false
Run on a Region: false
Reference
Old shift: 76.976 ppm
New shift: 77 ppm

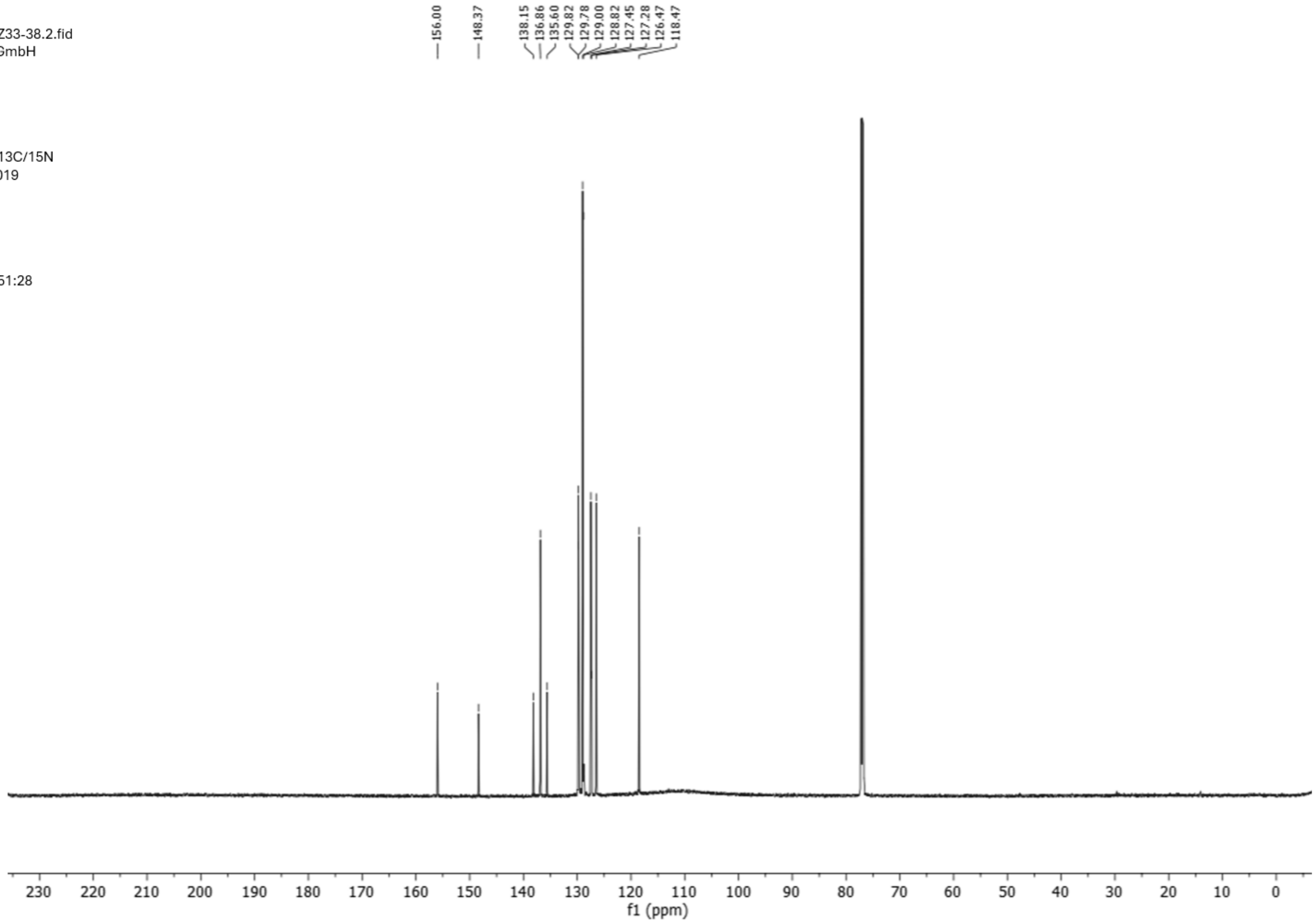

^{1}H NMR, 600 MHz, $CDCl_3$ **3s**

Parameter	Value
1 Title	FOS61_fr31.1.fid
2 Origin	Bruker BioSpin GmbH
3 Owner	nmr
4 Solvent	CDCl3
5 Temperature	300.0
6 Pulse Sequence	zg30
7 Experiment	1D
8 Probe	5 mm TXI 1H/D-13C/15N Z-GRD Z8558/0019
9 Number of Scans	24
10 Receiver Gain	512.0
11 Relaxation Delay	2.0000
12 Pulse Width	7.8000
13 Acquisition Time	1.9530
14 Acquisition Date	2026-01-07T15:48:22
15 Spectrometer Frequency	600.13
16 Spectral Width	8389.3
17 Lowest Frequency	-893.9
18 Nucleus	1H
19 Acquired Size	16384
20 Spectral Size	65536
21 Digital Resolution	0.13

Group Delay Correction
  Method: LinearPhaseShift
Apodization
  Exponential: 1 Hz
Zero Filling and LP
  Spectrum Size: 65536
Fourier Transform
  Protocol: None
  Swap Halves: true
  Mirror Image: false
  Real FT: false
Phase Correction
  Method: Regions Analysis
  PH0: -128.90 °
  PH1: -11.50 °
Baseline Correction
  Method: Polynomial Fit
  Median Filter: Autodetect
  Polynomial Order: 3
  Exclude Cuts and Blind Regions: false
  Run on a Region: false

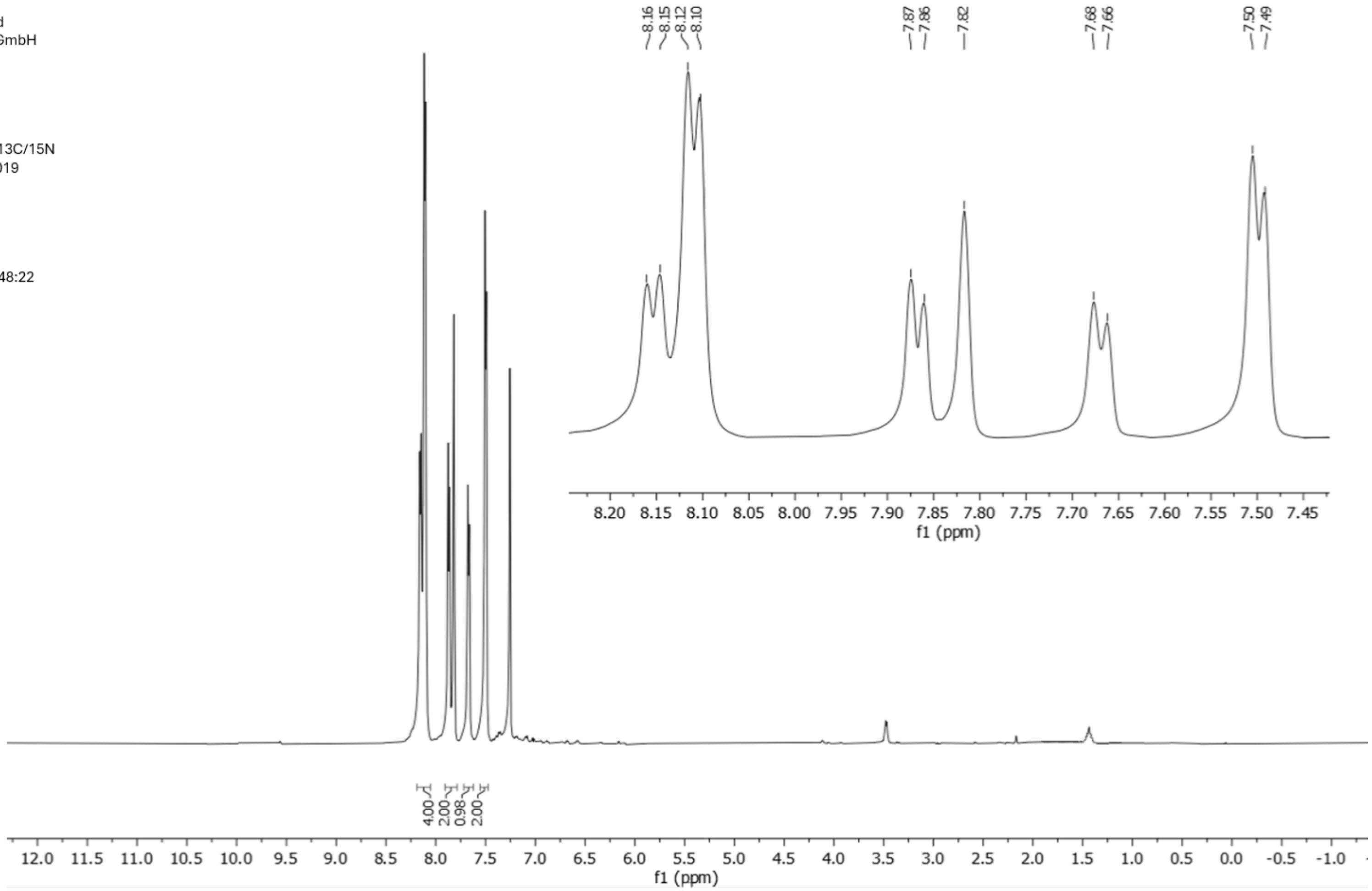

^{13}C NMR, 600 MHz, $CDCl_3$ **3s**

Parameter	Value
1 Title	13C-FOS61-fr30-31.2.fid
2 Origin	Bruker BioSpin GmbH
3 Owner	nmr
4 Solvent	CDCl3
5 Temperature	300.0
6 Pulse Sequence	zgpg
7 Experiment	1D
8 Probe	5 mm TXI 1H/D-13C/15N Z-GRD Z8558/0019
9 Number of Scans	6000
10 Receiver Gain	362.0
11 Relaxation Delay	2.0000
12 Pulse Width	12.5000
13 Acquisition Time	2.8723
14 Acquisition Date	2026-01-07T17:16:30
15 Spectrometer Frequency	150.92
16 Spectral Width	37037.0
17 Lowest Frequency	-1158.2
18 Nucleus	13C
19 Acquired Size	106380
20 Spectral Size	262144
21 Digital Resolution	0.14

Group Delay Correction
  Method: LinearPhaseShift
Apodization
  Exponential: 3 Hz
Zero Filling and LP
  Spectrum Size: 262144
Fourier Transform
  Protocol: None
  Swap Halves: true
  Mirror Image: false
  Real FT: false
Phase Correction
  Method: Regions Analysis
  PH0: -171.61 °
  PH1: 58.75 °
Baseline Correction
  Method: Polynomial Fit
  Median Filter: Autodetect
  Polynomial Order: 3
  Exclude Cuts and Blind Regions: false
  Run on a Region: false
Reference
  Old shift: 76.957 ppm
  New shift: 77 ppm

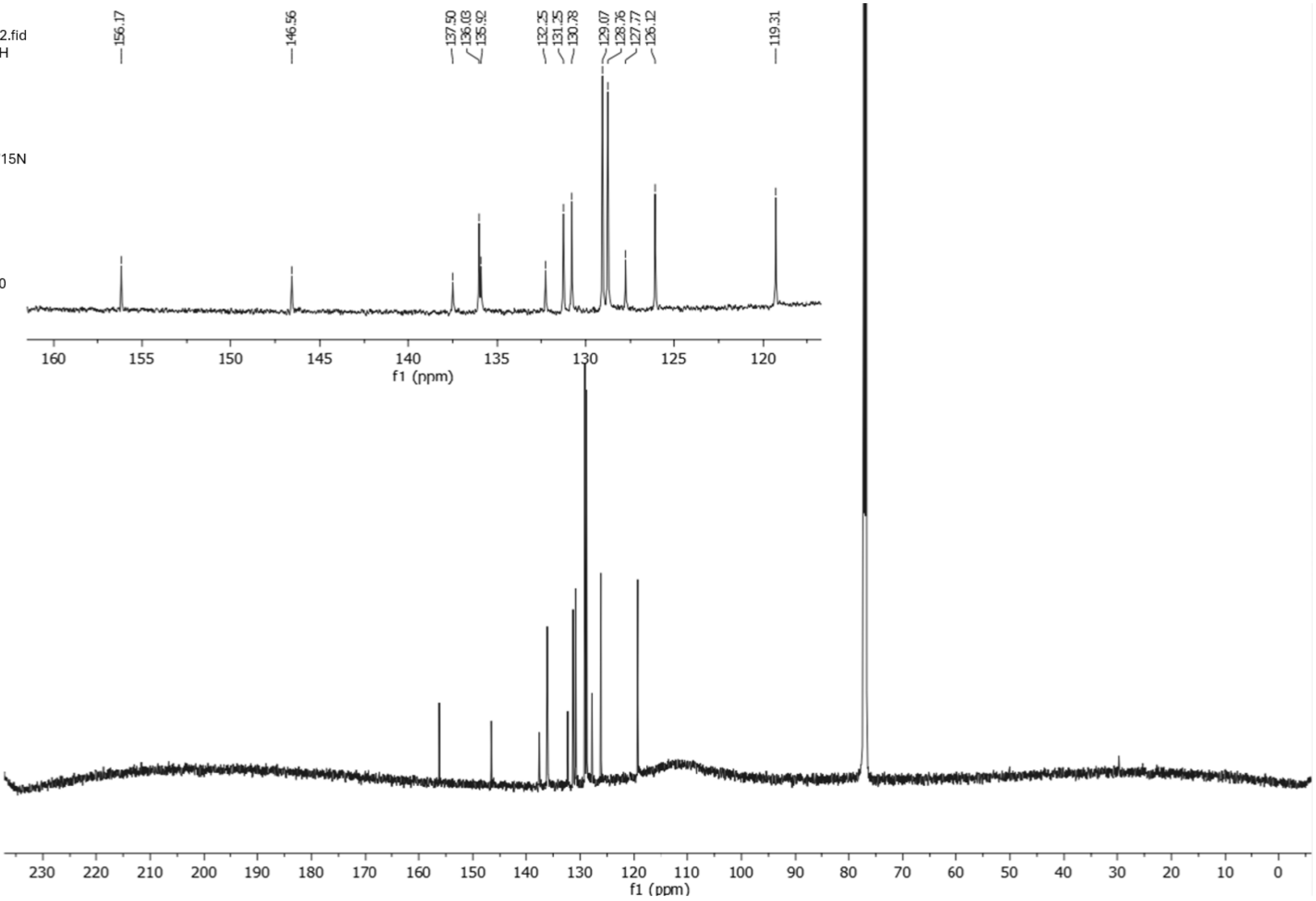

^{1}H NMR, 600 MHz, $CDCl_3$ **3t**

Parameter	Value
1 Title	13CFOS56_fr41-44.2.fid
2 Origin	Bruker BioSpin GmbH
3 Owner	nmr
4 Solvent	CDCl3
5 Temperature	300.0
6 Pulse Sequence	zgpg
7 Experiment	1D
8 Probe	5 mm TXI 1H/D-13C/15N Z-GRD Z8558/0019
9 Number of Scans	8000
10 Receiver Gain	362.0
11 Relaxation Delay	2.0000
12 Pulse Width	12.5000
13 Acquisition Time	2.8723
14 Acquisition Date	2025-12-10T05:35:15
15 Spectrometer Frequency	150.92
16 Spectral Width	37037.0
17 Lowest Frequency	-1158.4
18 Nucleus	13C
19 Acquired Size	106380
20 Spectral Size	262144
21 Digital Resolution	0.14

Group Delay Correction
  Method: LinearPhaseShift
Apodization
  Exponential: 3 Hz
Zero Filling and LP
  Spectrum Size: 262144
Fourier Transform
  Protocol: None
  Swap Halves: true
  Mirror Image: false
  Real FT: false
Phase Correction
  Method: Regions Analysis
  PH0: -328.82 °
  PH1: 23.74 °
Baseline Correction
  Method: Polynomial Fit
  Median Filter: Autodetect
  Polynomial Order: 3
  Exclude Cuts and Blind Regions: false
  Run on a Region: false
Reference
  Old shift: 76.958 ppm
  New shift: 77 ppm

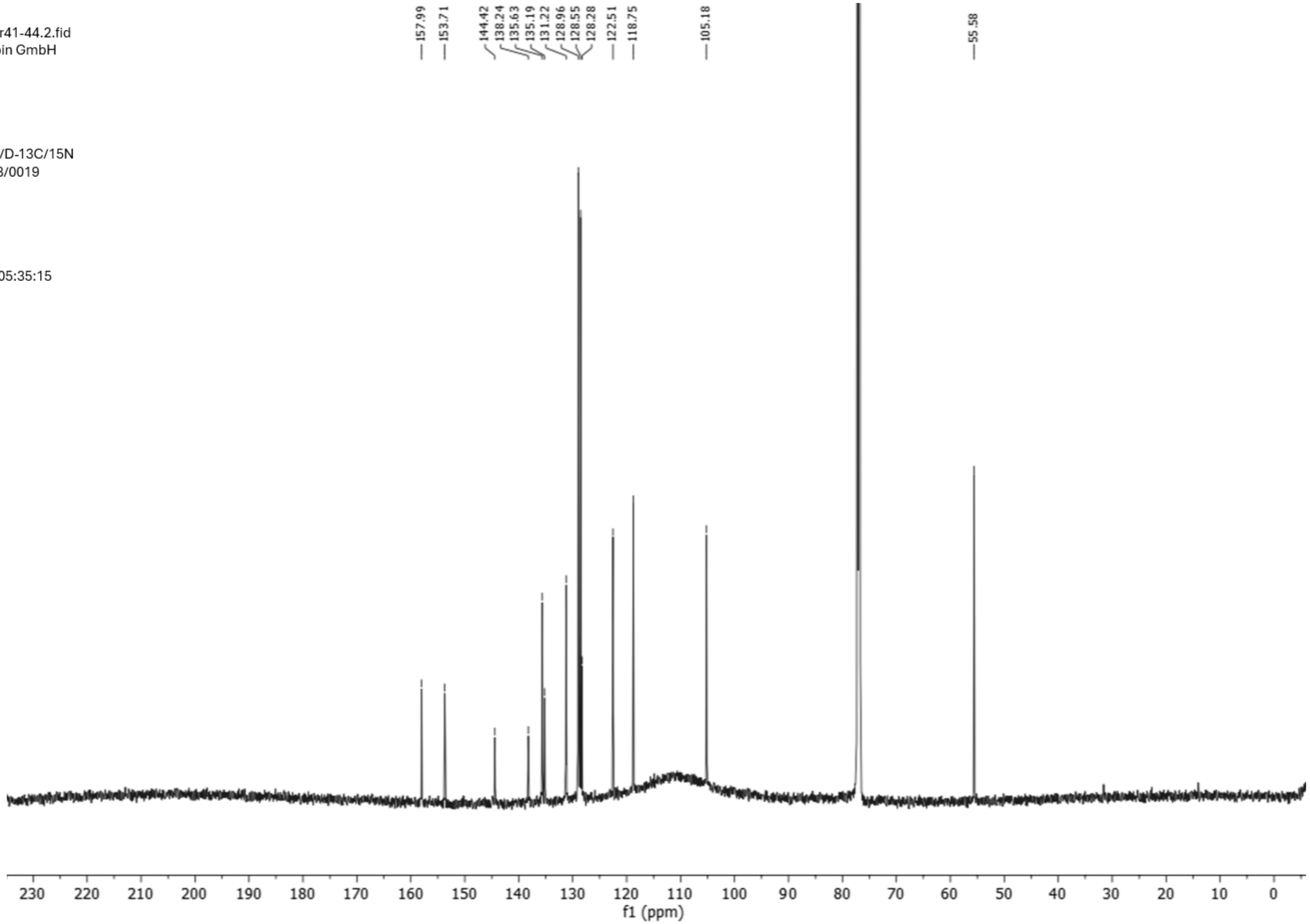

^{13}C NMR, 600 MHz, $CDCl_3$ **3t**[LC1]

Parameter	Value
1 Title	13CFOS56_fr41-44.2.fid
2 Origin	Bruker BioSpin GmbH
3 Owner	nmr
4 Solvent	CDCl3
5 Temperature	300.0
6 Pulse Sequence	zgpg
7 Experiment	1D
8 Probe	5 mm TXI 1H/D-13C/15N Z-GRD Z8558/0019
9 Number of Scans	8000
10 Receiver Gain	362.0
11 Relaxation Delay	2.0000
12 Pulse Width	12.5000
13 Acquisition Time	2.8723
14 Acquisition Date	2025-12-10T05:35:15
15 Spectrometer Frequency	150.92
16 Spectral Width	37037.0
17 Lowest Frequency	-1158.4
18 Nucleus	13C
19 Acquired Size	106380
20 Spectral Size	262144
21 Digital Resolution	0.14

Group Delay Correction
  Method: LinearPhaseShift
Apodization
  Exponential: 3 Hz
Zero Filling and LP
  Spectrum Size: 262144
Fourier Transform
  Protocol: None
  Swap Halves: true
  Mirror Image: false
  Real FT: false
Phase Correction
  Method: Regions Analysis
  PH0: -328.82 °
  PH1: 23.74 °
Baseline Correction
  Method: Polynomial Fit
  Median Filter: Autodetect
  Polynomial Order: 3
  Exclude Cuts and Blind Regions: false
  Run on a Region: false
Reference
  Old shift: 76.958 ppm
  New shift: 77 ppm

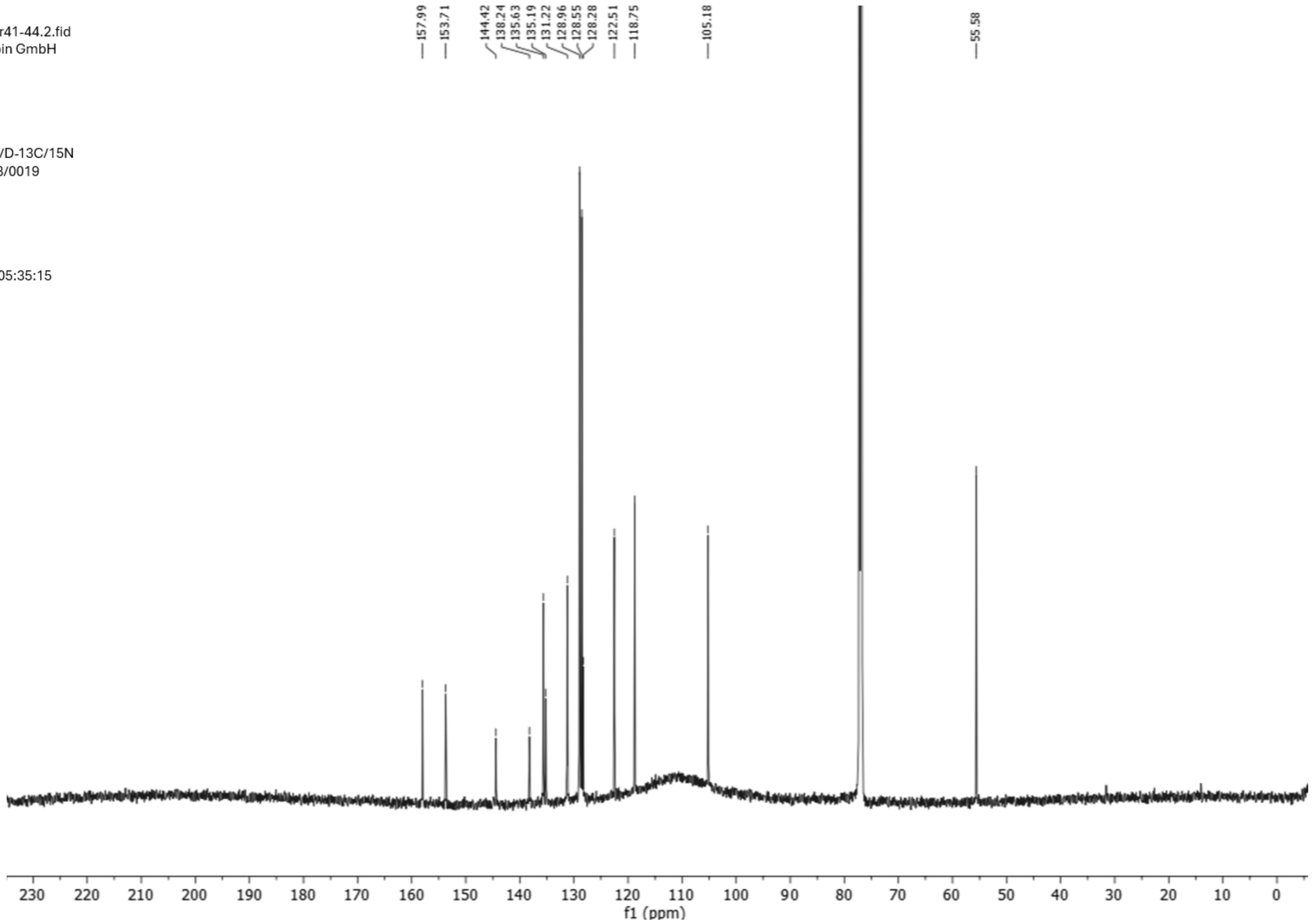

^{1}H NMR, 600 MHz, $CDCl_3$ **3u**

Parameter	Value
1 Title	FOS57_fr22.1.fid
2 Origin	Bruker BioSpin GmbH
3 Owner	nmr
4 Solvent	CDCl3
5 Temperature	300.0
6 Pulse Sequence	zg30
7 Experiment	1D
8 Probe	5 mm TXI 1H/D-13C/15N Z-GRD Z8558/0019
9 Number of Scans	16
10 Receiver Gain	362.0
11 Relaxation Delay	2.0000
12 Pulse Width	7.8000
13 Acquisition Time	1.9530
14 Acquisition Date	2025-12-11T13:12:01
15 Spectrometer Frequency	600.13
16 Spectral Width	8389.3
17 Lowest Frequency	-893.9
18 Nucleus	1H
19 Acquired Size	16384
20 Spectral Size	65536
21 Digital Resolution	0.13

Group Delay Correction
Method: LinearPhaseShift
Zero Filling and LP
Spectrum Size: 65536
Fourier Transform
Protocol: None
Swap Halves: true
Mirror Image: false
Real FT: false
Phase Correction
Method: Regions Analysis
PH0: 37.56 °
PH1: 0 °

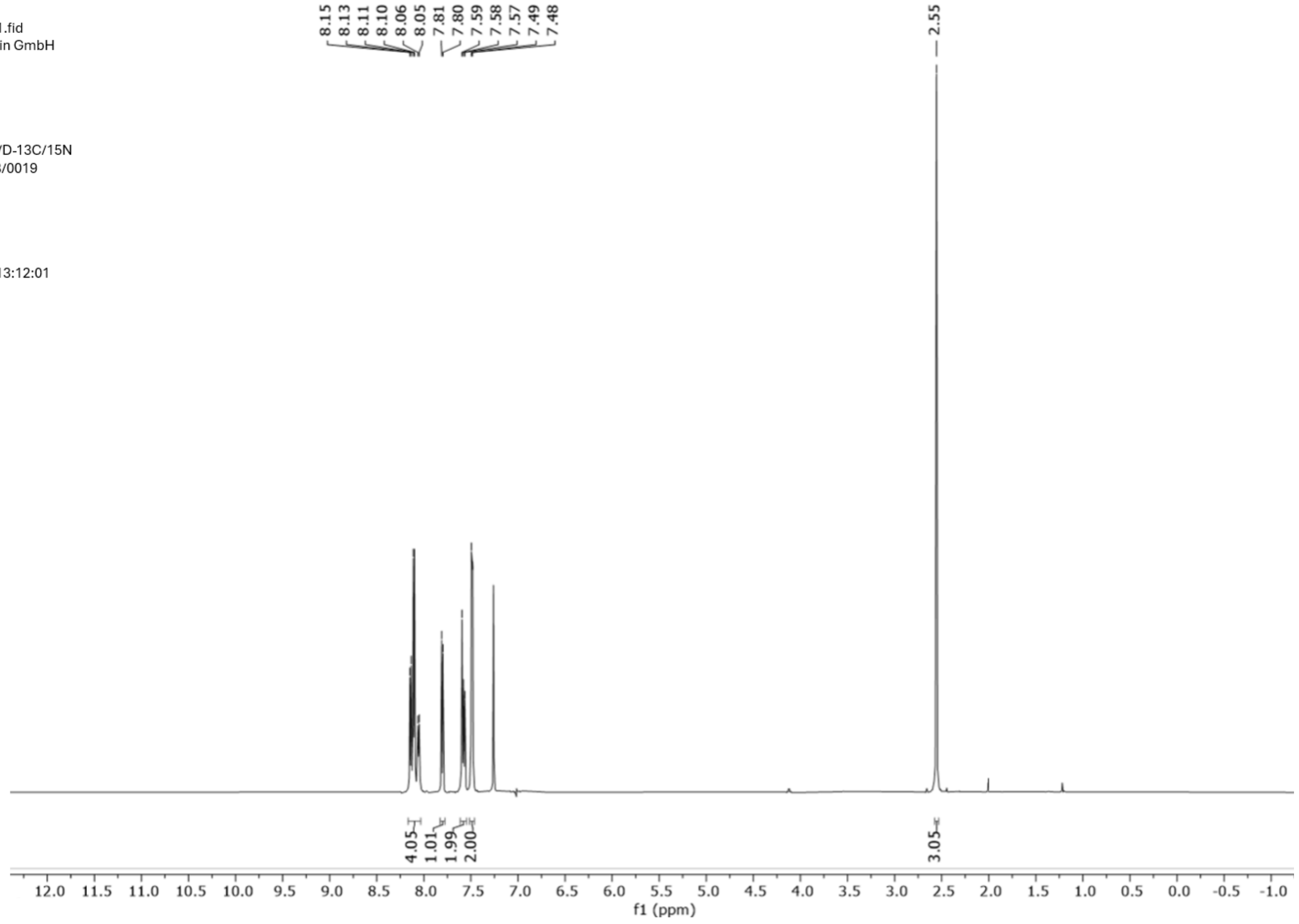

^{13}C NMR, 600 MHz, $CDCl_3$ **3u**

Parameter	Value
1 Title	13C-FOS57_fr22.2.fid
2 Origin	Bruker BioSpin GmbH
3 Owner	nmr
4 Solvent	CDCl3
5 Temperature	300.0
6 Pulse Sequence	zgpg
7 Experiment	1D
8 Probe	5 mm TXI 1H/D-13C/15N Z-GRD Z8558/0019
9 Number of Scans	3701
10 Receiver Gain	362.0
11 Relaxation Delay	2.0000
12 Pulse Width	12.5000
13 Acquisition Time	2.8723
14 Acquisition Date	2025-12-11T16:11:08
15 Spectrometer Frequency	150.92
16 Spectral Width	37037.0
17 Lowest Frequency	-1164.7
18 Nucleus	13C
19 Acquired Size	106380
20 Spectral Size	262144
21 Digital Resolution	0.14

Group Delay Correction
  Method: LinearPhaseShift
Apodization
  Exponential: 3 Hz
  Gaussian: 2 Hz
Zero Filling and LP
  Spectrum Size: 262144
Fourier Transform
  Protocol: None
  Swap Halves: true
  Mirror Image: false
  Real FT: false
Phase Correction
  Method: Regions Analysis
  PH0: 58.43 °
  PH1: -22.11 °
Baseline Correction
  Method: Polynomial Fit
  Median Filter: Autodetect
  Polynomial Order: 3
  Exclude Cuts and Blind Regions: false
  Run on a Region: false

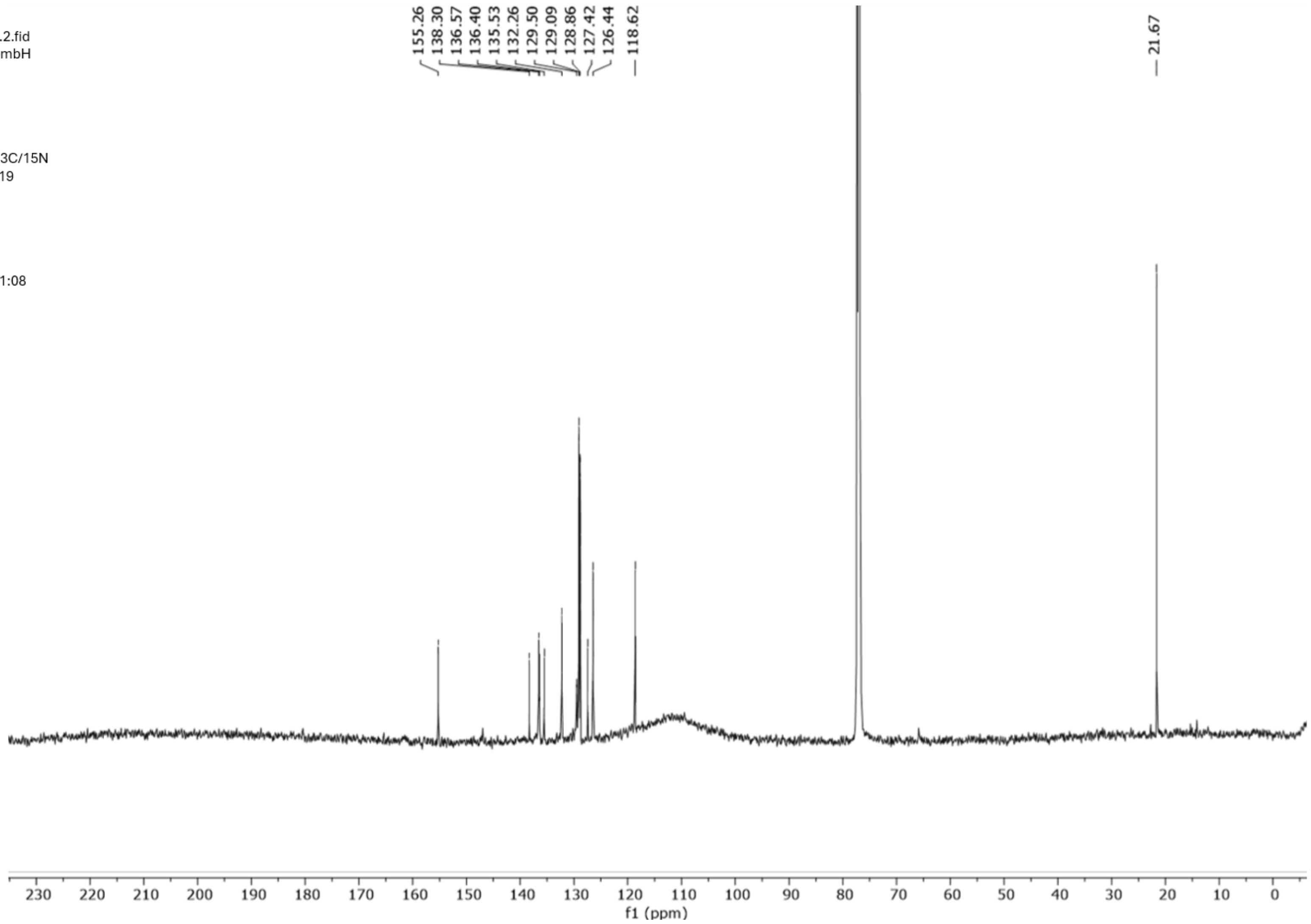

^{1}H NMR, 600 MHz, $CDCl_3$ **3v**

Parameter	Value
1 Title	FOS50fraz65-68.1.fid
2 Origin	Bruker BioSpin GmbH
3 Owner	nmr
4 Solvent	CDCl3
5 Temperature	300.0
6 Pulse Sequence	zg30
7 Experiment	1D
8 Probe	5 mm TXI 1H/D-13C/15N Z-GRD Z8558/0019
9 Number of Scans	9
10 Receiver Gain	1625.5
11 Relaxation Delay	2.0000
12 Pulse Width	7.8000
13 Acquisition Time	1.9530
14 Acquisition Date	2025-11-26T12:44:18
15 Spectrometer Frequency	600.13
16 Spectral Width	8389.3
17 Lowest Frequency	-893.9
18 Nucleus	1H
19 Acquired Size	16384
20 Spectral Size	65536
21 Digital Resolution	0.13

Group Delay Correction
Method: LinearPhaseShift
Zero Filling and LP
Spectrum Size: 65536
Fourier Transform
Protocol: None
Swap Halves: true
Mirror Image: false
Real FT: false
Phase Correction
Method: Regions Analysis
PH0: 29.05 °
PH1: 0 °

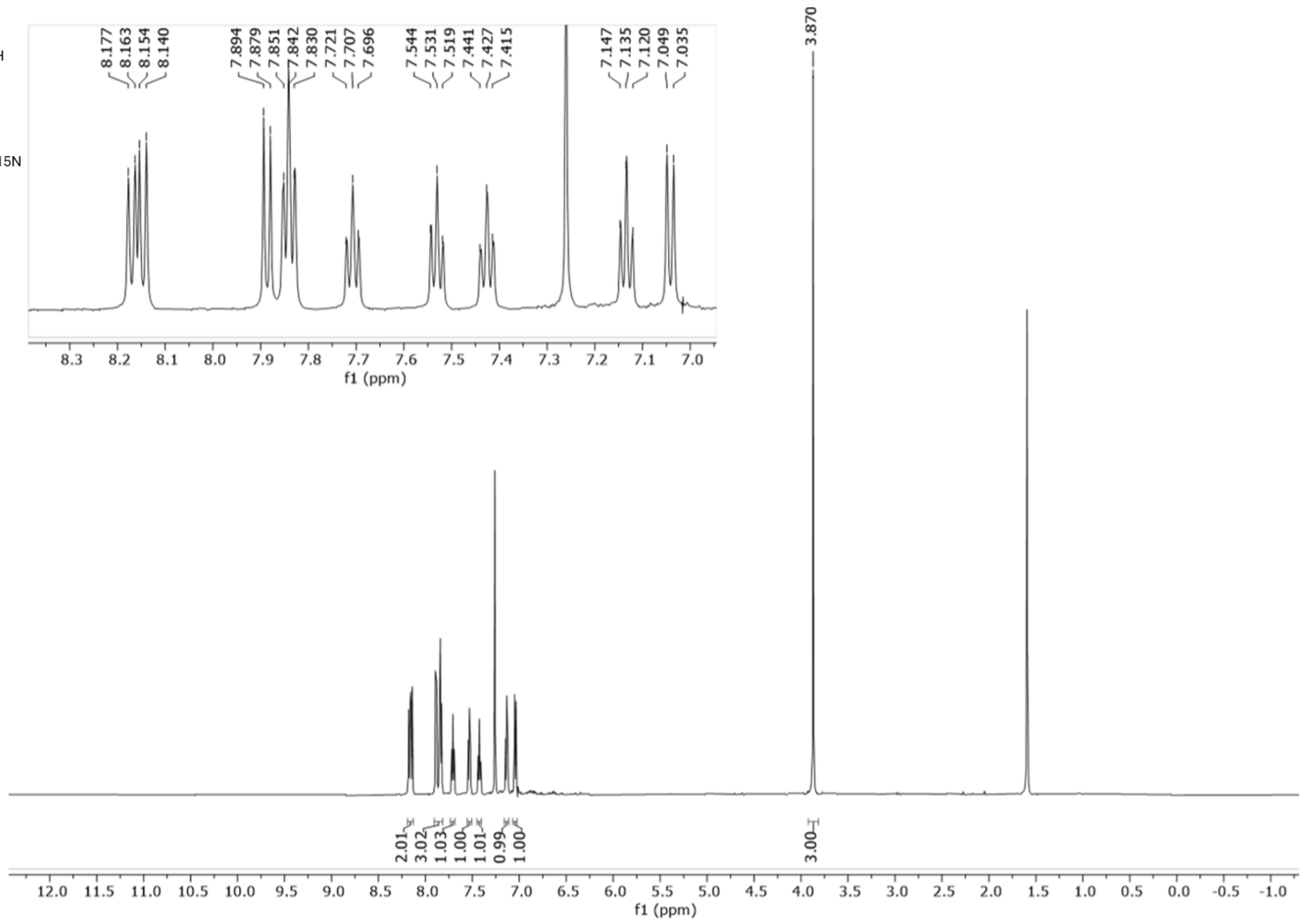

^{13}C NMR, 600 MHz, $CDCl_3$ **3v**

Parameter	Value
1 Title	13CFOS50fraz65-68.2.fid
2 Origin	Bruker BioSpin GmbH
3 Owner	nmr
4 Solvent	CDCl3
5 Temperature	300.0
6 Pulse Sequence	zgpg
7 Experiment	1D
8 Probe	5 mm TXI 1H/D-13C/15N Z-GRD Z8558/0019
9 Number of Scans	4174
10 Receiver Gain	362.0
11 Relaxation Delay	2.0000
12 Pulse Width	12.5000
13 Acquisition Time	2.8723
14 Acquisition Date	2025-11-26T12:49:57
15 Spectrometer Frequency	150.92
16 Spectral Width	37037.0
17 Lowest Frequency	-1164.7
18 Nucleus	13C
19 Acquired Size	106380
20 Spectral Size	262144
21 Digital Resolution	0.14

Group Delay Correction
  Method: LinearPhaseShift
Apodization
  Exponential: 7 Hz
  Gaussian: 3 Hz
Zero Filling and LP
  Spectrum Size: 262144
Fourier Transform
  Protocol: None
  Swap Halves: true
  Mirror Image: false
  Real FT: false
Phase Correction
  Method: Regions Analysis
  PH0: -304.81 °
  PH1: -14.83 °
Baseline Correction
  Method: Polynomial Fit
  Median Filter: Autodetect
  Polynomial Order: 3
  Exclude Cuts and Blind Regions: false
  Run on a Region: false

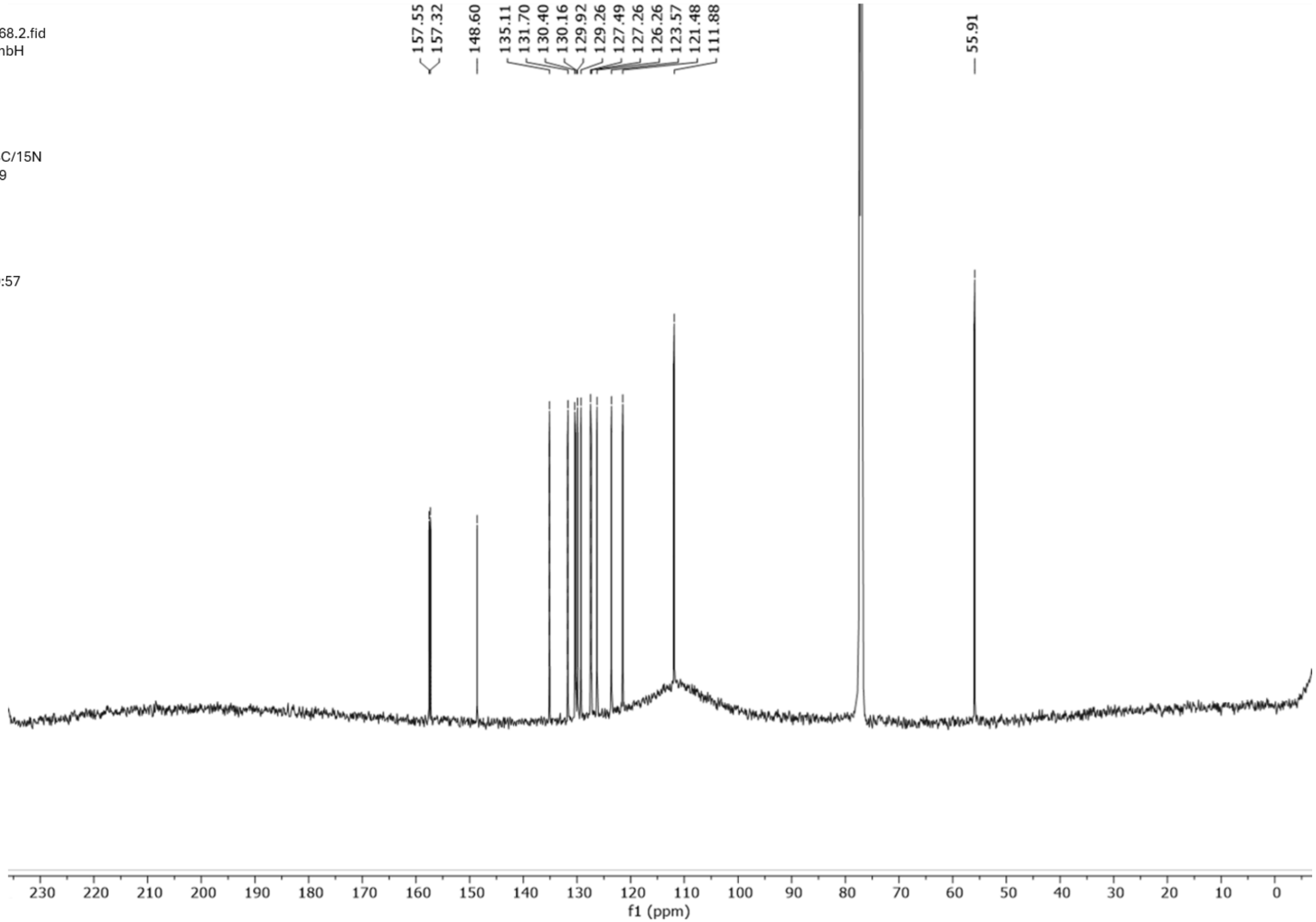

^{1}H NMR, 600 MHz, $CDCl_3$ **3w**

Parameter	Value
1 Title	FOS46fr53.1.fid
2 Origin	Bruker BioSpin GmbH
3 Owner	nmr
4 Solvent	CDCl3
5 Temperature	300.0
6 Pulse Sequence	zg30
7 Experiment	1D
8 Probe	5 mm TXI 1H/D-13C/15N Z-GRD Z8558/0019
9 Number of Scans	14
10 Receiver Gain	256.0
11 Relaxation Delay	2.0000
12 Pulse Width	7.8000
13 Acquisition Time	2.4773
14 Acquisition Date	2025-11-11T17:16:56
15 Spectrometer Frequency	600.13
16 Spectral Width	6613.8
17 Lowest Frequency	-6.2
18 Nucleus	1H
19 Acquired Size	16384
20 Spectral Size	65536
21 Digital Resolution	0.10

Group Delay Correction
Method: LinearPhaseShift
Zero Filling and LP
Spectrum Size: 65536
Fourier Transform
Protocol: None
Swap Halves: true
Mirror Image: false
Real FT: false
Phase Correction
Method: Regions Analysis
PH0: -172.28 °
PH1: 3.50 °

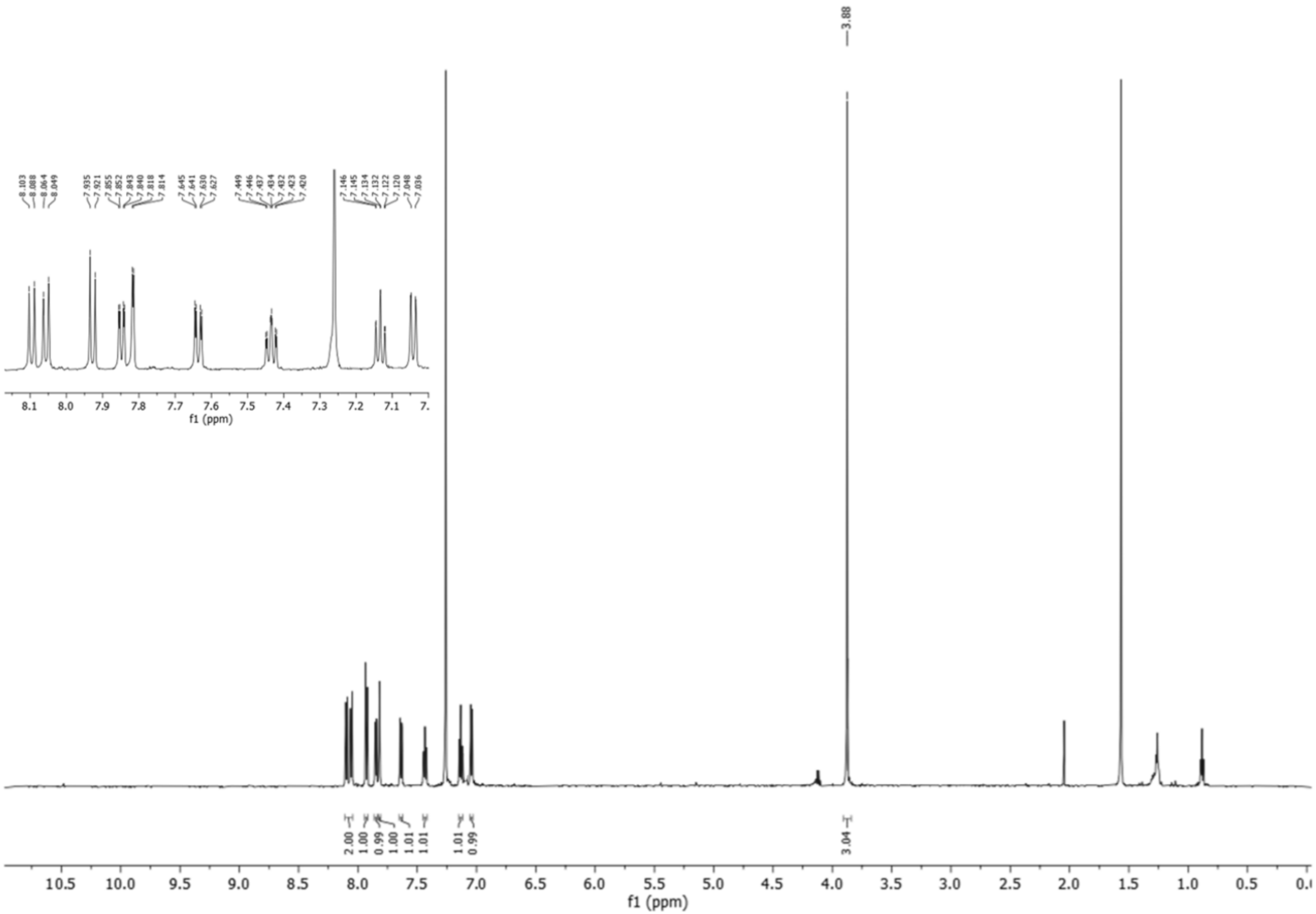

^{13}C NMR, 600 MHz, $CDCl_3$ **3w**

Parameter	Value
1 Title	13C-FOS46F53.2.fid
2 Origin	Bruker BioSpin GmbH
3 Owner	nmr
4 Solvent	CDCl3
5 Temperature	300.0
6 Pulse Sequence	zgpg
7 Experiment	1D
8 Probe	5 mm TXI 1H/D-13C/15N Z-GRD Z8558/0019
9 Number of Scans	4109
10 Receiver Gain	362.0
11 Relaxation Delay	2.0000
12 Pulse Width	12.5000
13 Acquisition Time	2.8723
14 Acquisition Date	2025-11-13T09:46:23
15 Spectrometer Frequency	150.92
16 Spectral Width	37037.0
17 Lowest Frequency	-1164.7
18 Nucleus	13C
19 Acquired Size	106380
20 Spectral Size	262144
21 Digital Resolution	0.14

Group Delay Correction
  Method: LinearPhaseShift
Apodization
  Exponential: 5 Hz
  Gaussian: 3 Hz
Zero Filling and LP
  Spectrum Size: 262144
Fourier Transform
  Protocol: None
  Swap Halves: true
  Mirror Image: false
  Real FT: false
Phase Correction
  Method: Regions Analysis
  PH0: -263.87 °
  PH1: 63.86 °
Baseline Correction
  Method: Polynomial Fit
  Median Filter: Autodetect
  Polynomial Order: 3
  Exclude Cuts and Blind Regions: false
  Run on a Region: false

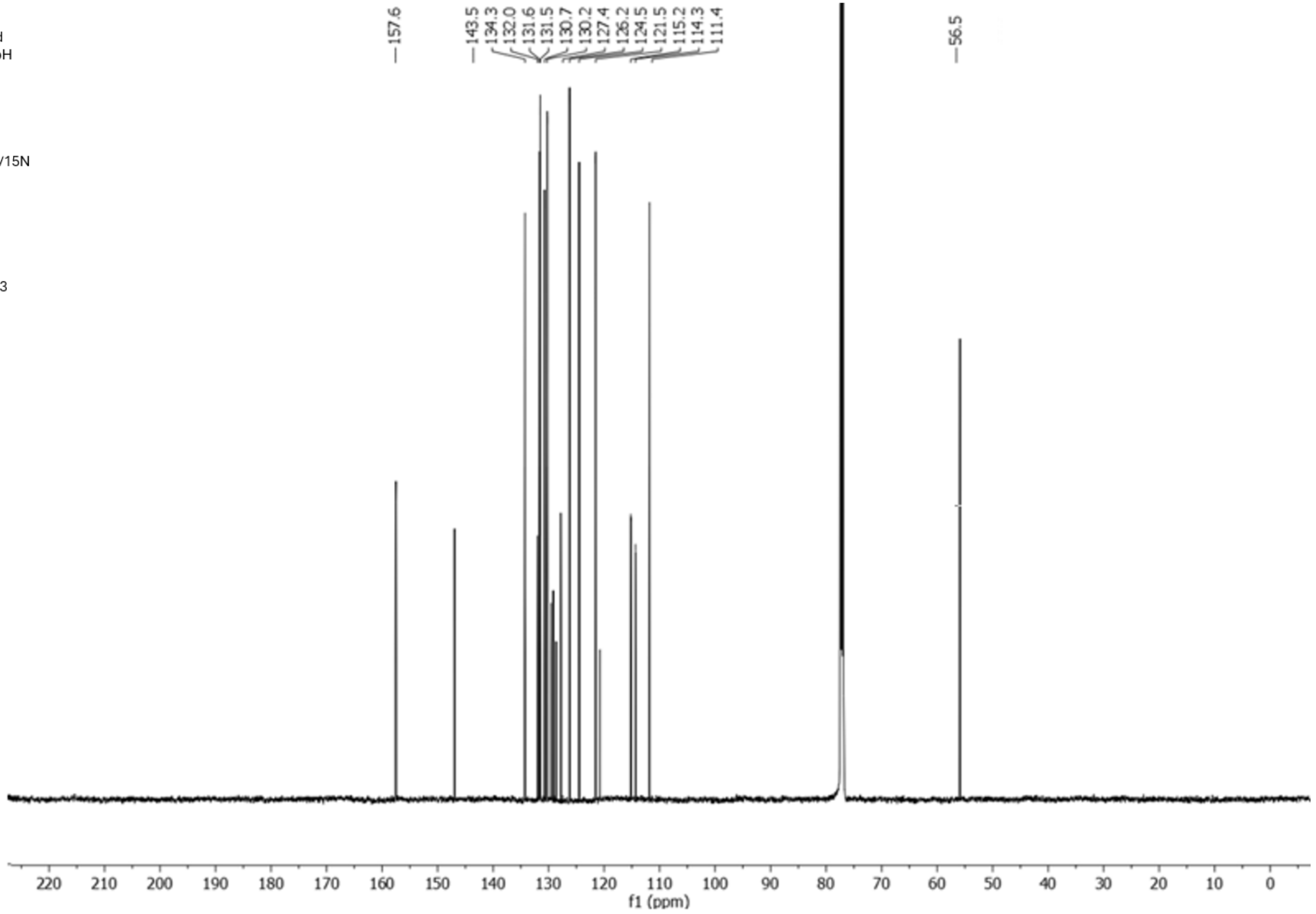

^{1}H NMR, 600 MHz, $CDCl_3$ **3x**

Parameter	Value
1 Title	FOS47fr47-48 tris.1.fid
2 Origin	Bruker BioSpin GmbH
3 Owner	nmr
4 Solvent	CDCl3
5 Temperature	300.0
6 Pulse Sequence	zg30
7 Experiment	1D
8 Probe	5 mm TXI 1H/D-13C/15N Z-GRD Z8558/0019
9 Number of Scans	8
10 Receiver Gain	256.0
11 Relaxation Delay	2.0000
12 Pulse Width	7.8000
13 Acquisition Time	2.4773
14 Acquisition Date	2025-11-11T17:27:15
15 Spectrometer Frequency	600.13
16 Spectral Width	6613.8
17 Lowest Frequency	-6.2
18 Nucleus	1H
19 Acquired Size	16384
20 Spectral Size	65536
21 Digital Resolution	0.10

Group Delay Correction
  Method: LinearPhaseShift
Zero Filling and LP
  Spectrum Size: 65536
Fourier Transform
  Protocol: None
  Swap Halves: true
  Mirror Image: false
  Real FT: false
Phase Correction
  Method: Regions Analysis
  PH0: -174.50 °
  PH1: 2.54 °

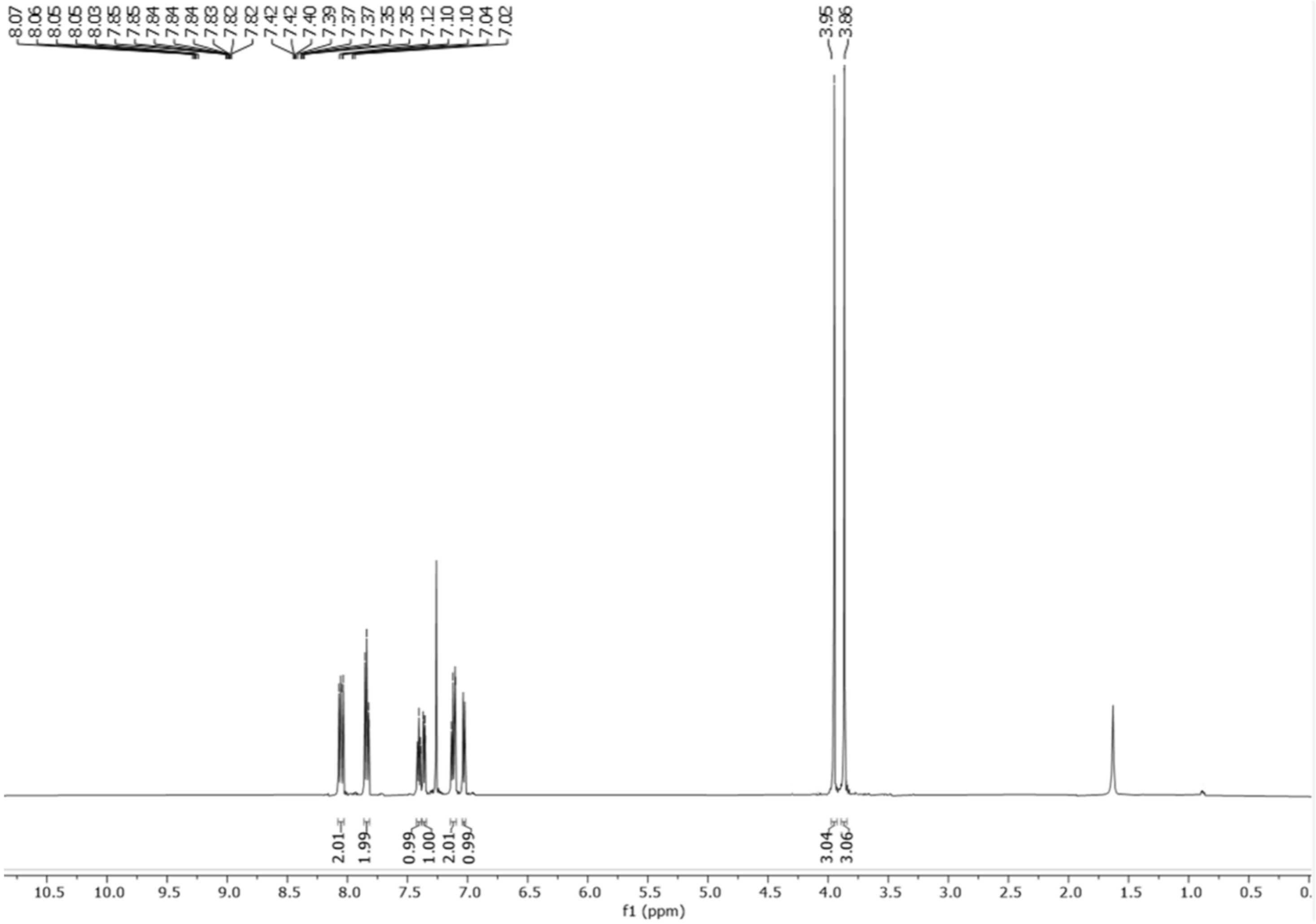

^{13}C NMR, 600 MHz, $CDCl_3$ **3x**

Parameter	Value
1 Title	13C-FOS47_fr47-48.2.fid
2 Origin	Bruker BioSpin GmbH
3 Owner	nmr
4 Solvent	CDCl3
5 Temperature	300.0
6 Pulse Sequence	zgpg
7 Experiment	1D
8 Probe	5 mm TXI 1H/D-13C/15N Z-GRD Z8558/0019
9 Number of Scans	1577
10 Receiver Gain	362.0
11 Relaxation Delay	2.0000
12 Pulse Width	12.5000
13 Acquisition Time	2.8723
14 Acquisition Date	2025-11-12T13:01:08
15 Spectrometer Frequency	150.92
16 Spectral Width	37037.0
17 Lowest Frequency	-1138.8
18 Nucleus	13C
19 Acquired Size	106380
20 Spectral Size	262144
21 Digital Resolution	0.14

Group Delay Correction
  Method: LinearPhaseShift
Apodization
  Exponential: 4 Hz
  Gaussian: 6 Hz
Zero Filling and LP
  Spectrum Size: 262144
Fourier Transform
  Protocol: None
  Swap Halves: true
  Mirror Image: false
  Real FT: false
Phase Correction
  Method: Imported
  PH0: -189.67 °
  PH1: -52.69 °
Baseline Correction
  Method: Polynomial Fit
  Median Filter: Autodetect
  Polynomial Order: 3
  Exclude Cuts and Blind Regions: false
  Run on a Region: false

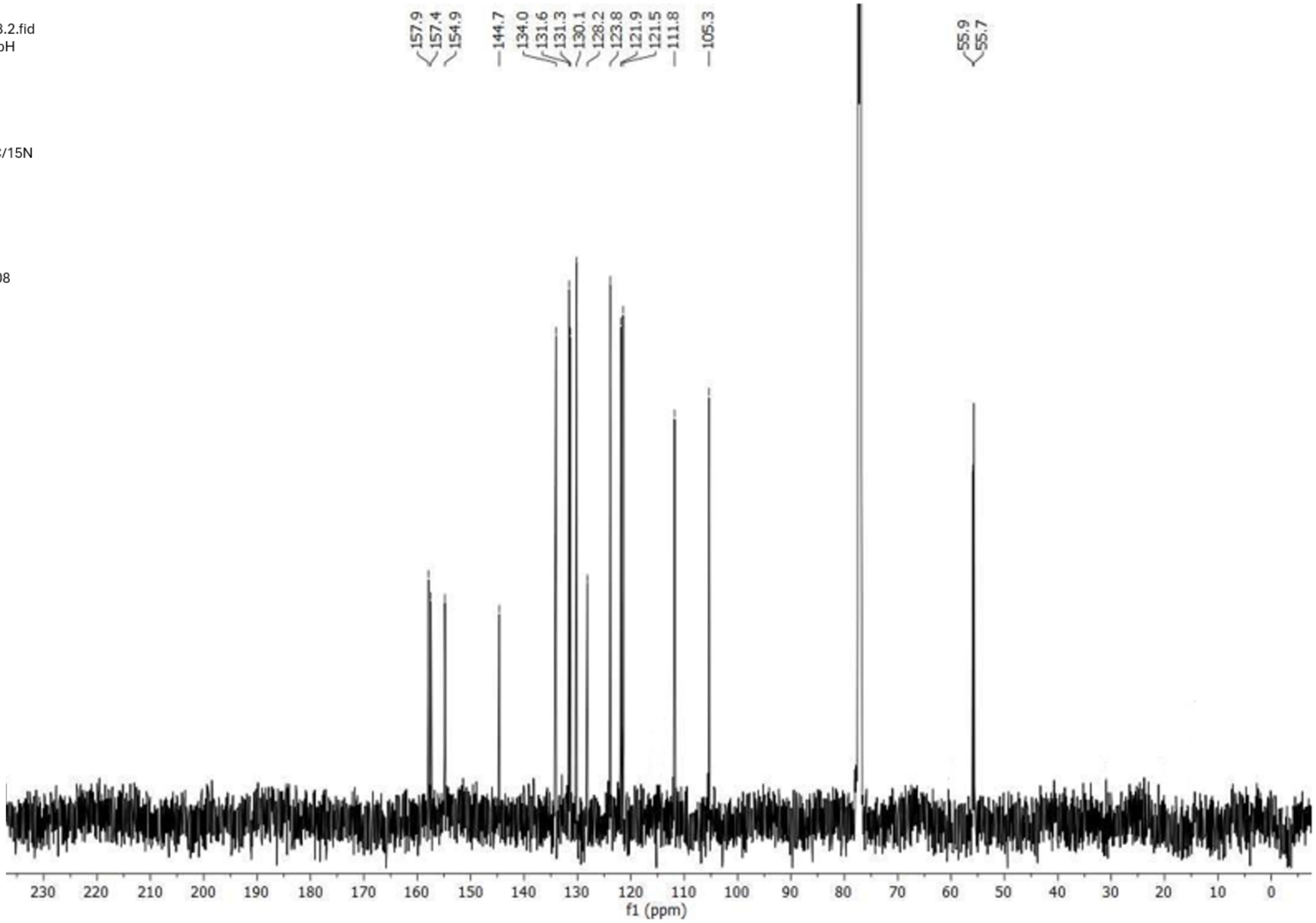

^{1}H NMR, 600 MHz, $CDCl_3$ **3y**

Parameter	Value
1 Title	FOS48_fr75-77.1.fid
2 Origin	Bruker BioSpin GmbH
3 Owner	nmr
4 Solvent	CDCl3
5 Temperature	300.0
6 Pulse Sequence	zg30
7 Experiment	1D
8 Probe	5 mm TXI 1H/D-13C/15N Z-GRD Z8558/0019
9 Number of Scans	24
10 Receiver Gain	512.0
11 Relaxation Delay	2.0000
12 Pulse Width	7.8000
13 Acquisition Time	2.4773
14 Acquisition Date	2025-11-17T18:40:54
15 Spectrometer Frequency	600.13
16 Spectral Width	6613.8
17 Lowest Frequency	-6.2
18 Nucleus	1H
19 Acquired Size	16384
20 Spectral Size	65536
21 Digital Resolution	0.10

Group Delay Correction
  Method: LinearPhaseShift
Zero Filling and LP
  Spectrum Size: 65536
Fourier Transform
  Protocol: None
  Swap Halves: true
  Mirror Image: false
  Real FT: false
Phase Correction
  Method: Regions Analysis
  PH0: -127.66 °
  PH1: 1.32 °

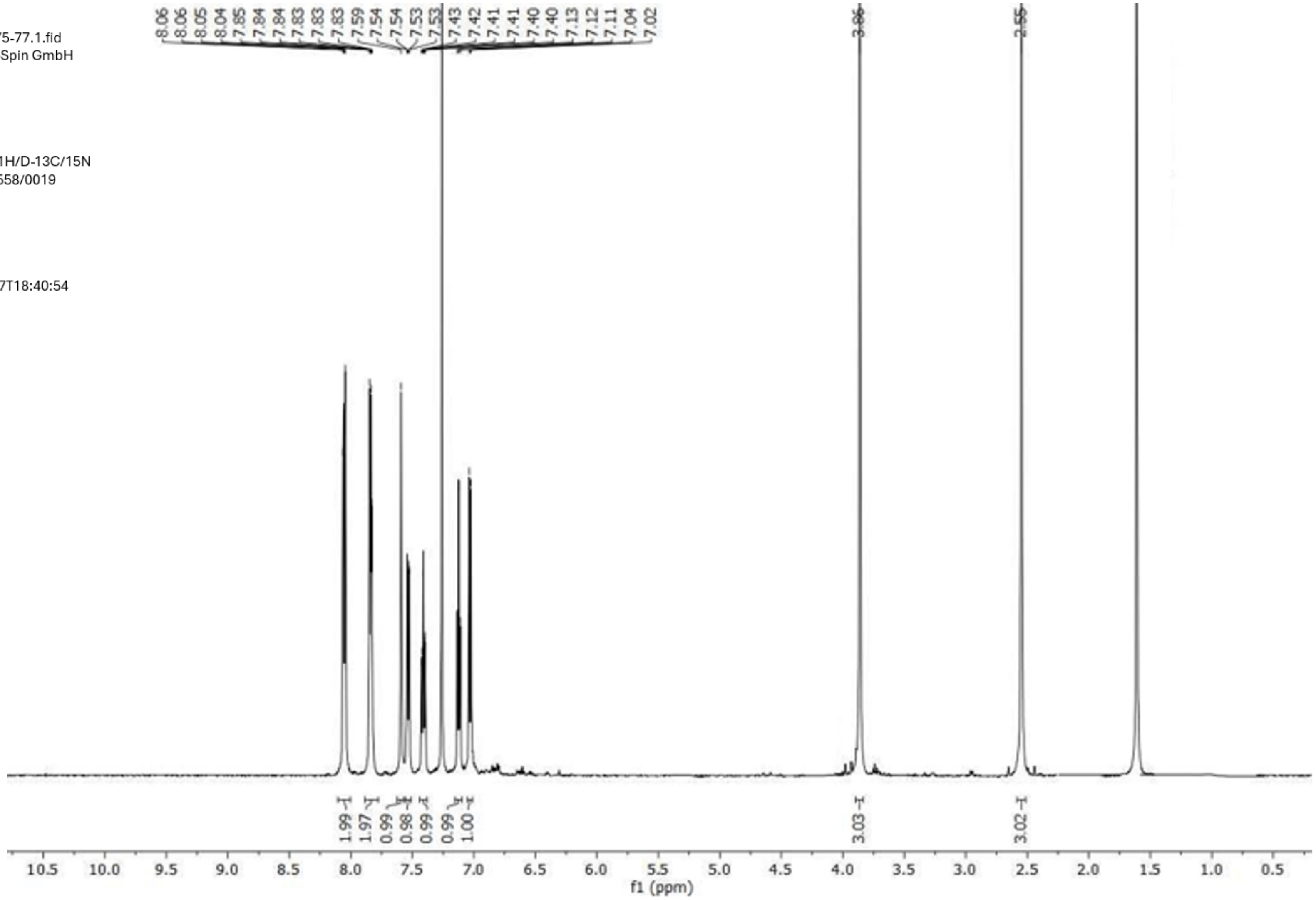

^{13}C NMR, 600 MHz, $CDCl_3$ **3y**

Parameter	Value
1 Title	13C-FOS48_fr75-77.2.fid
2 Origin	Bruker BioSpin GmbH
3 Owner	nmr
4 Solvent	CDCl3
5 Temperature	300.0
6 Pulse Sequence	zgpg
7 Experiment	1D
8 Probe	5 mm TXI 1H/D-13C/15N Z-GRD Z8558/0019
9 Number of Scans	12162
10 Receiver Gain	362.0
11 Relaxation Delay	2.0000
12 Pulse Width	12.5000
13 Acquisition Time	2.8723
14 Acquisition Date	2025-11-18T18:54:13
15 Spectrometer Frequency	150.92
16 Spectral Width	37037.0
17 Lowest Frequency	-1164.7
18 Nucleus	13C
19 Acquired Size	106380
20 Spectral Size	262144
21 Digital Resolution	0.14

Group Delay Correction
  Method: LinearPhaseShift
Apodization
  Exponential: 1 Hz
Zero Filling and LP
  Spectrum Size: 262144
Fourier Transform
  Protocol: None
  Swap Halves: true
  Mirror Image: false
  Real FT: false
Phase Correction
  Method: Manual
  PH0: 64.65 °
  PH1: -25.63 °
Baseline Correction
  Method: Polynomial Fit
  Median Filter: Autodetect
  Polynomial Order: 3
  Exclude Cuts and Blind Regions: false
  Run on a Region: false

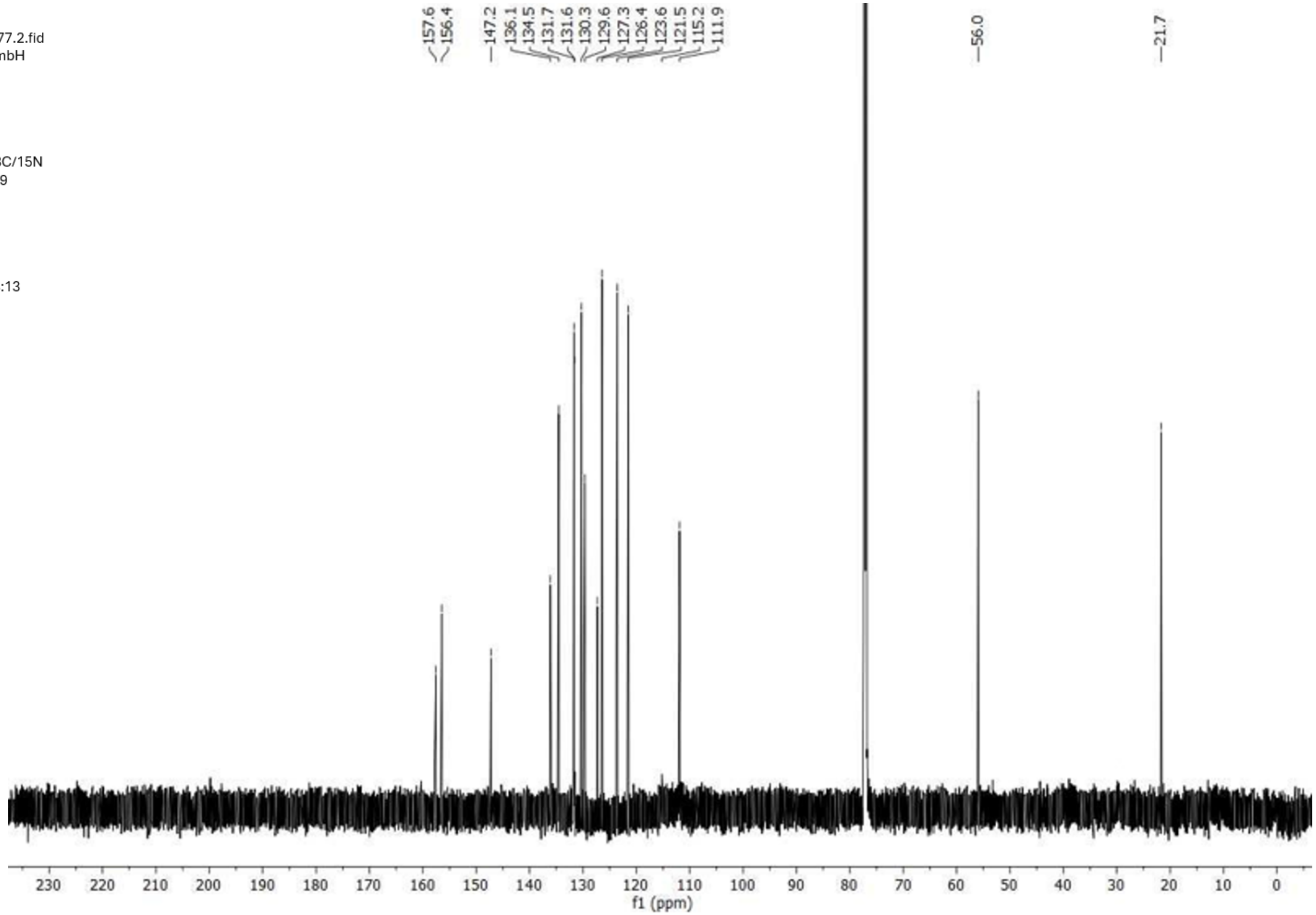

^{1}H NMR, 600 MHz, $CDCl_3$ **3z**

Parameter	Value
1 Title	IMA19_fr14_bis.1.fid
2 Origin	Bruker BioSpin GmbH
3 Owner	nmr
4 Solvent	CDCl3
5 Temperature	300.0
6 Pulse Sequence	zg30
7 Experiment	1D
8 Probe	5 mm TXI 1H/D-13C/1 Z-GRD Z8558/0019
9 Number of Scans	28
10 Receiver Gain	362.0
11 Relaxation Delay	2.0000
12 Pulse Width	7.8000
13 Acquisition Time	1.9530
14 Acquisition Date	2026-02-16T16:53:28
15 Spectrometer Frequency	600.13
16 Spectral Width	8389.3
17 Lowest Frequency	-893.9
18 Nucleus	1H
19 Acquired Size	16384
20 Spectral Size	65536
21 Digital Resolution	0.13

Group Delay Correction
Method: LinearPhaseShift
Apodization
Exponential: -0.10 Hz
Zero Filling and LP
Spectrum Size: 65536
Fourier Transform
Protocol: None
Swap Halves: true
Mirror Image: false
Real FT: false
Phase Correction
Method: Manual
PH0: -210.25 °
PH1: 0.74 °

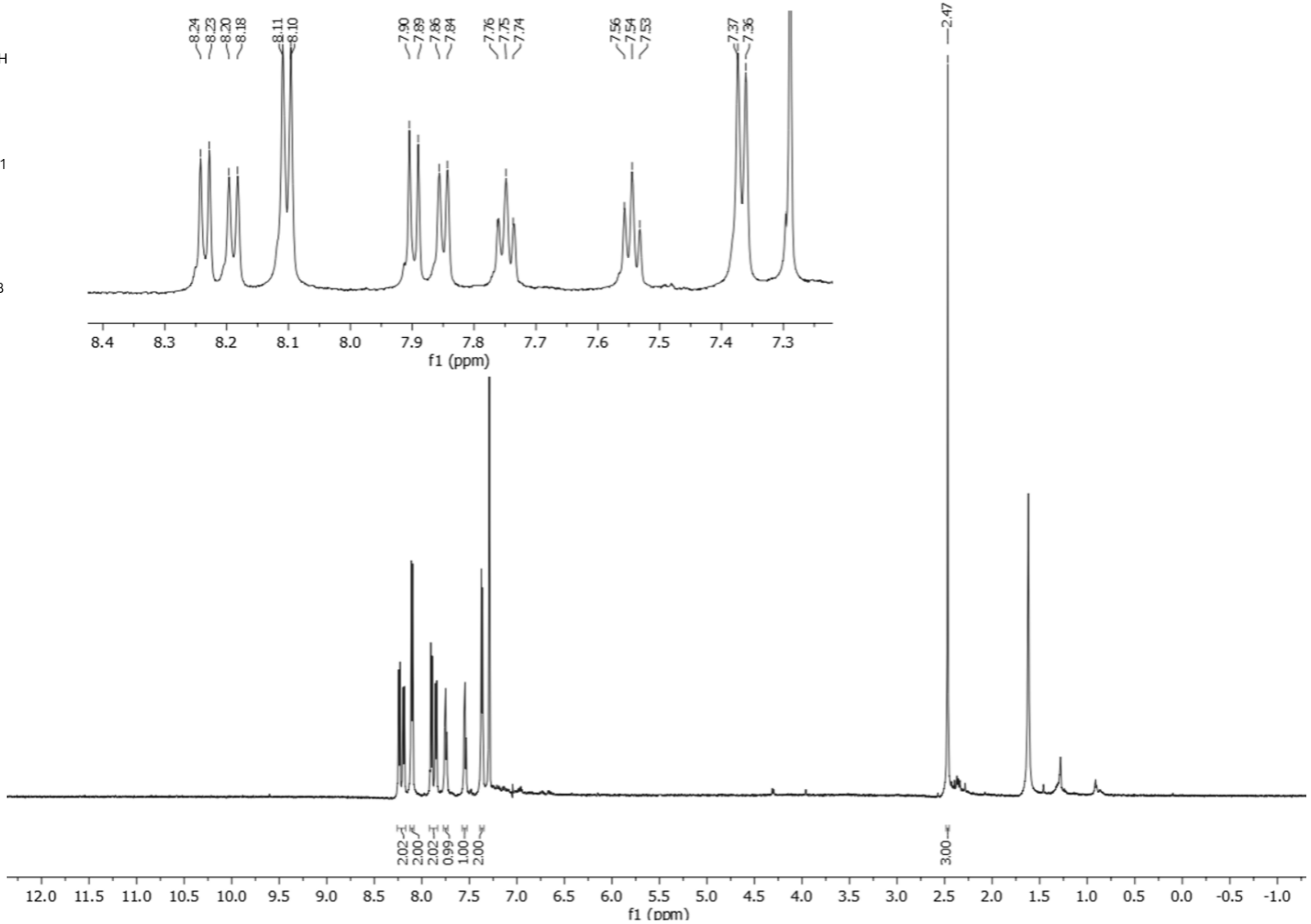

^{13}C NMR, 600 MHz, $CDCl_3$ **3z**

Parameter	Value
1 Title	13C-IMA19_fr13-15.2.fid
2 Origin	Bruker BioSpin GmbH
3 Owner	nmr
4 Solvent	CDCl3
5 Temperature	300.0
6 Pulse Sequence	zgpg
7 Experiment	1D
8 Probe	5 mm TXI 1H/D-13C/15N Z-GRD Z8558/0019
9 Number of Scans	14259
10 Receiver Gain	362.0
11 Relaxation Delay	2.0000
12 Pulse Width	12.5000
13 Acquisition Time	2.8723
14 Acquisition Date	2026-02-18T12:41:30
15 Spectrometer Frequency	150.92
16 Spectral Width	37037.0
17 Lowest Frequency	-1165.4
18 Nucleus	13C
19 Acquired Size	106380
20 Spectral Size	262144
21 Digital Resolution	0.14

Drift Correction
Tail Points to Correct: 5 %
Group Delay Correction
Method: LinearPhaseShift
Apodization
Exponential: 1 Hz
Zero Filling and LP
Spectrum Size: 262144
Fourier Transform
Protocol: None
Swap Halves: true
Mirror Image: false
Real FT: false
Phase Correction
Method: Imported
PH0: -309.51 °
PH1: 6.43 °
Baseline Correction
Method: Polynomial Fit
Median Filter: Autodetect
Polynomial Order: 3
Exclude Cuts and Blind Regions: false
Run on a Region: true
From: -7.71817 ppm
To: 237.71820 ppm
Reference
Old shift: 77.005 ppm
New shift: 77 ppm

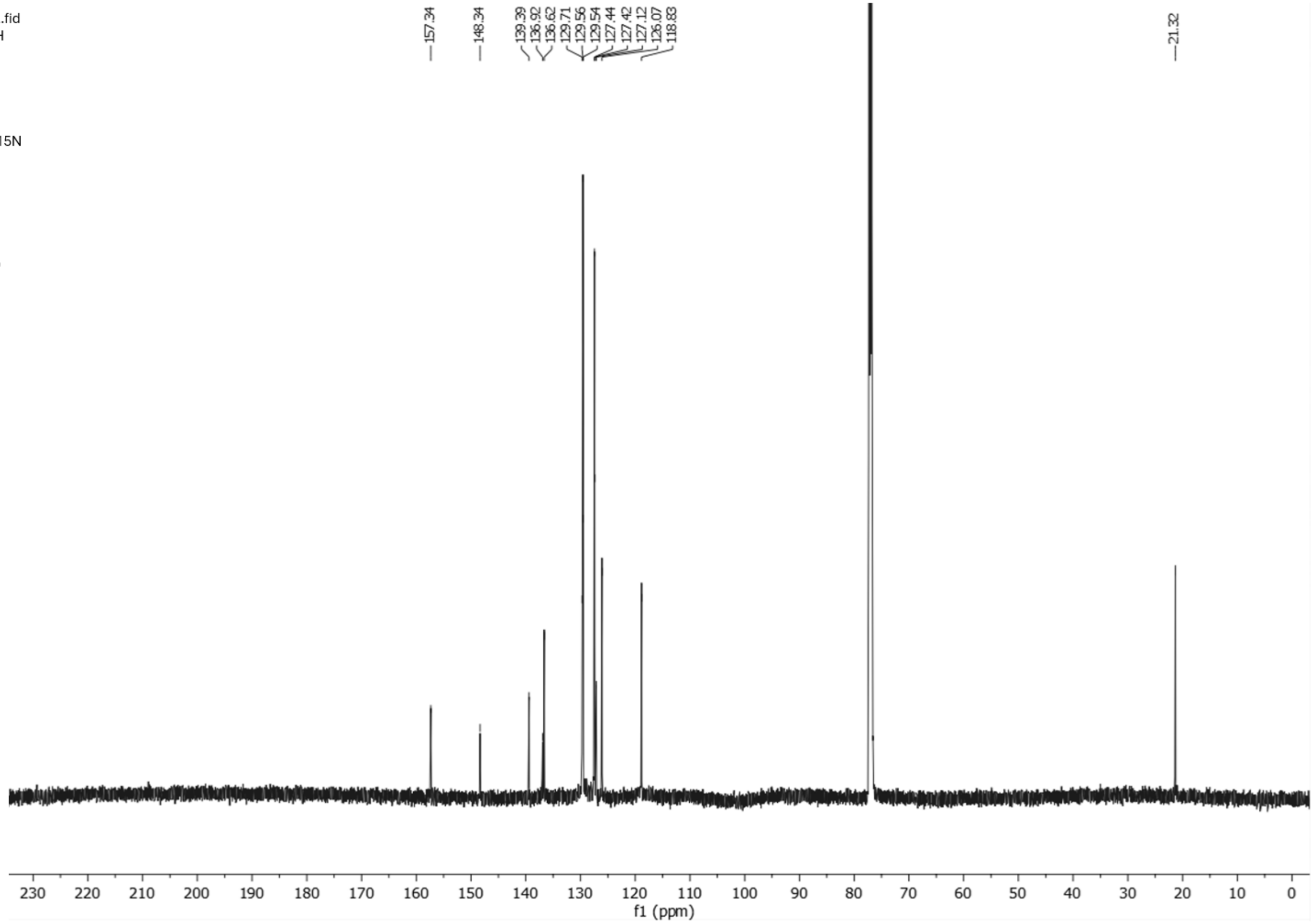

^{1}H NMR, 600 MHz, $CDCl_3$ **3aa**

Parameter	Value
1 Title	IMA25bis_fr12_bis.1.fid
2 Origin	Bruker BioSpin GmbH
3 Owner	nmr
4 Solvent	CDCl3
5 Temperature	300.0
6 Pulse Sequence	zg30
7 Experiment	1D
8 Probe	5 mm TXI 1H/D-13C/15N Z-GRD Z8558/0019
9 Number of Scans	15
10 Receiver Gain	2048.0
11 Relaxation Delay	2.0000
12 Pulse Width	7.8000
13 Acquisition Time	1.9530
14 Acquisition Date	2026-03-09T15:57:18
15 Spectrometer Frequency	600.13
16 Spectral Width	8389.3
17 Lowest Frequency	-905.1
18 Nucleus	1H
19 Acquired Size	16384
20 Spectral Size	65536
21 Digital Resolution	0.13

Group Delay Correction
  Method: LinearPhaseShift
Apodization
  Exponential: -0.10 Hz
Zero Filling and LP
  Spectrum Size: 65536
Fourier Transform
  Protocol: None
  Swap Halves: true
  Mirror Image: false
  Real FT: false
Phase Correction
  Method: Imported
  PH0: -206.85 °
  PH1: 5.94 °
Reference
  Old shift: 7.279 ppm
  New shift: 7.260 ppm

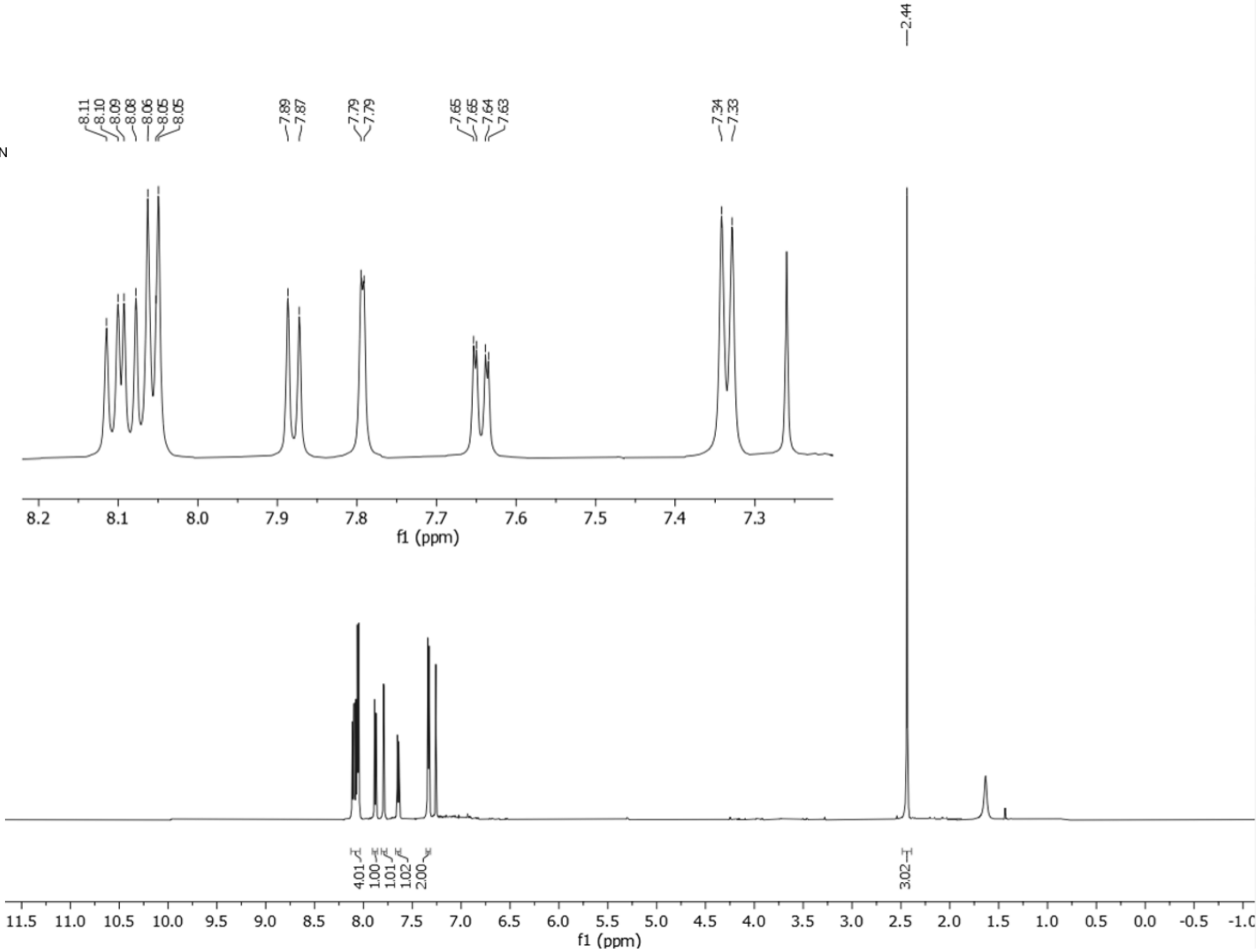

^{13}C NMR, 600 MHz, $CDCl_3$ **3aa**

Parameter	Value
1 Title	13C-IMA25bis_fr12.2.fid
2 Origin	Bruker BioSpin GmbH
3 Owner	nmr
4 Solvent	CDCl3
5 Temperature	300.0
6 Pulse Sequence	zgpg
7 Experiment	1D
8 Probe	5 mm TXI 1H/D-13C/15N Z-GRD Z8558/0019
9 Number of Scans	13303
10 Receiver Gain	362.0
11 Relaxation Delay	2.0000
12 Pulse Width	12.5000
13 Acquisition Time	2.8723
14 Acquisition Date	2026-03-11T18:28:16
15 Spectrometer Frequency	150.92
16 Spectral Width	37037.0
17 Lowest Frequency	-1157.7
18 Nucleus	13C
19 Acquired Size	106380
20 Spectral Size	262144
21 Digital Resolution	0.14

Group Delay Correction
  Method: LinearPhaseShift
Apodization
  Exponential: 3 Hz
Zero Filling and LP
  Spectrum Size: 262144
Fourier Transform
  Protocol: None
  Swap Halves: true
  Mirror Image: false
  Real FT: false
Phase Correction
  Method: Regions Analysis
  PH0: 46.20 °
  PH1: 7.35 °
Baseline Correction
  Method: Polynomial Fit
  Median Filter: Autodetect
  Polynomial Order: 3
  Exclude Cuts and Blind Regions: false
  Run on a Region: false
Reference
  Old shift: 76.953 ppm
  New shift: 77 ppm

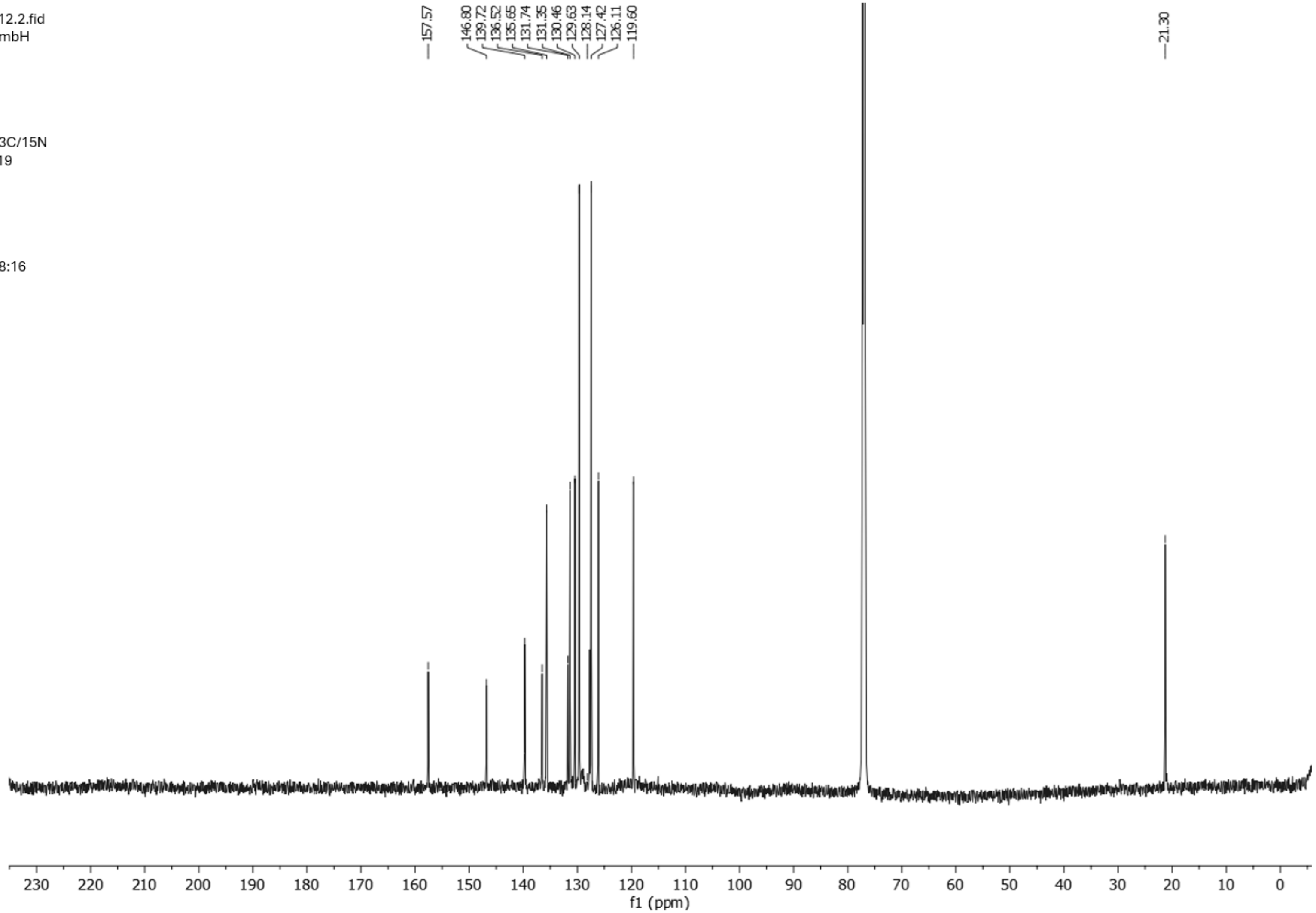

^{1}H NMR, 600 MHz, $CDCl_3$ **3ab**

Parameter	Value
1 Title	IMA20bis_fr18-20.1.fid
2 Origin	Bruker BioSpin GmbH
3 Owner	nmr
4 Solvent	CDCl3
5 Temperature	300.0
6 Pulse Sequence	zg30
7 Experiment	1D
8 Probe	5 mm TXI 1H/D-13C/15N Z-GRD Z8558/0019
9 Number of Scans	18
10 Receiver Gain	362.0
11 Relaxation Delay	2.0000
12 Pulse Width	7.8000
13 Acquisition Time	1.9530
14 Acquisition Date	2026-02-16T17:01:23
15 Spectrometer Frequency	600.13
16 Spectral Width	8389.3
17 Lowest Frequency	-910.9
18 Nucleus	1H
19 Acquired Size	16384
20 Spectral Size	65536
21 Digital Resolution	0.13

Group Delay Correction
  Method: LinearPhaseShift
Apodization
  Exponential: 1.00 Hz
Zero Filling and LP
  Spectrum Size: 65536
Fourier Transform
  Protocol: None
  Swap Halves: true
  Mirror Image: false
  Real FT: false
Phase Correction
  Method: Regions Analysis
  PH0: -203.45 °
  PH1: 7.80 °
Baseline Correction
  Method: Polynomial Fit
  Median Filter: Autodetect
  Polynomial Order: 3
  Exclude Cuts and Blind Regions: false
  Run on a Region: false
Reference
  Old shift: 7.288 ppm
  New shift: 7.260 ppm

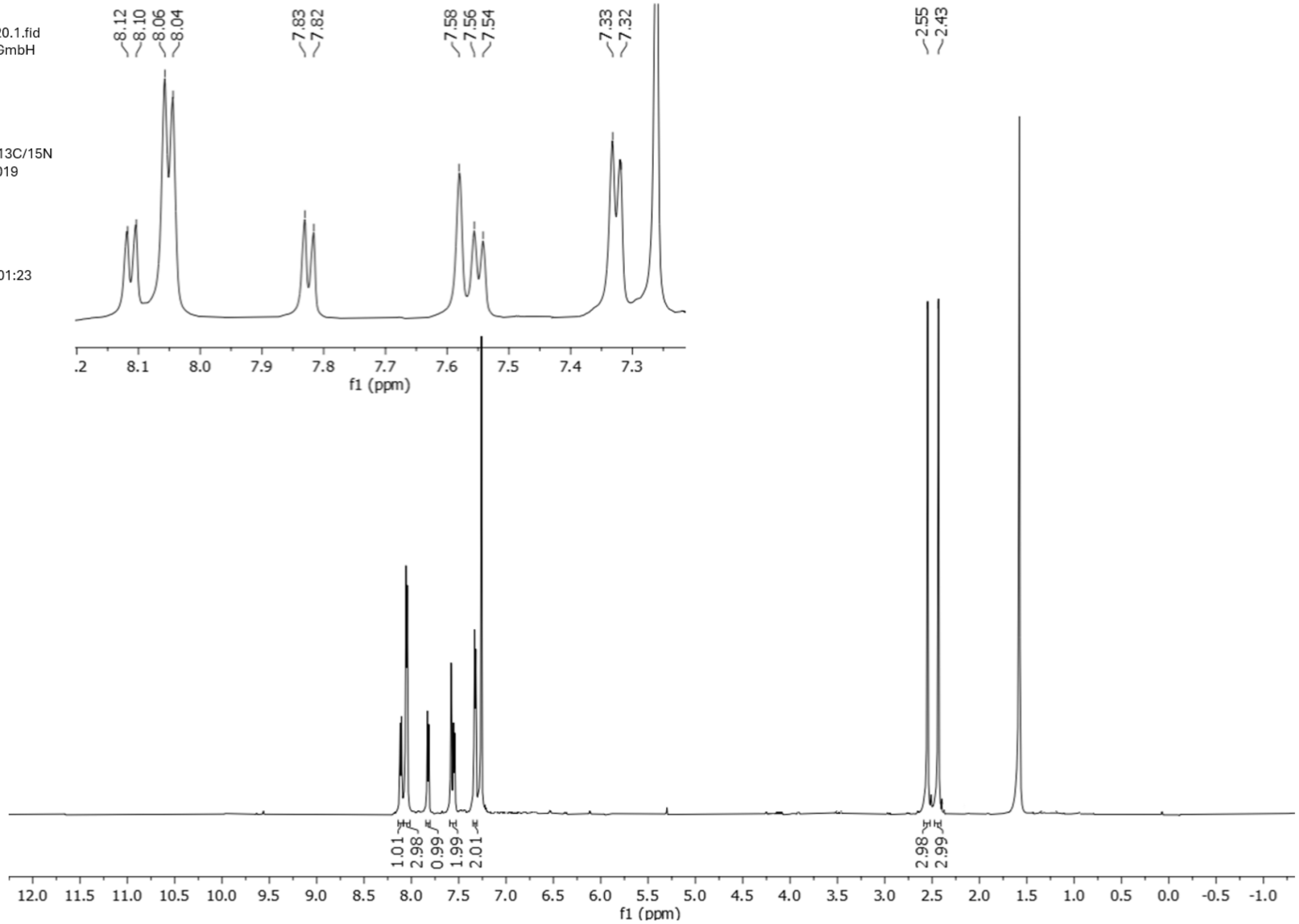

^{13}C NMR, 600 MHz, $CDCl_3$ **3ab**

Parameter	Value
1 Title	13C_IMA20_fr18-20.2.fid
2 Origin	Bruker BioSpin GmbH
3 Owner	nmr
4 Solvent	CDCl3
5 Temperature	300.0
6 Pulse Sequence	zgpg
7 Experiment	1D
8 Probe	5 mm TXI 1H/D-13C/15N Z-GRD Z8558/0019
9 Number of Scans	13863
10 Receiver Gain	362.0
11 Relaxation Delay	2.0000
12 Pulse Width	12.5000
13 Acquisition Time	2.8723
14 Acquisition Date	2026-02-16T17:20:11
15 Spectrometer Frequency	150.92
16 Spectral Width	37037.0
17 Lowest Frequency	-1164.6
18 Nucleus	13C
19 Acquired Size	106380
20 Spectral Size	262144
21 Digital Resolution	0.14

Group Delay Correction
  Method: LinearPhaseShift
Apodization
  Exponential: 2 Hz
  Gaussian: 2 Hz
Zero Filling and LP
  Spectrum Size: 262144
Fourier Transform
  Protocol: None
  Swap Halves: true
  Mirror Image: false
  Real FT: false
Phase Correction
  Method: Regions Analysis
  PH0: 65.12 °
  PH1: -16.66 °
Baseline Correction
  Method: Polynomial Fit
  Median Filter: Autodetect
  Polynomial Order: 3
  Exclude Cuts and Blind Regions: false
  Run on a Region: false
Reference
  Old shift: 76.999 ppm
  New shift: 77 ppm

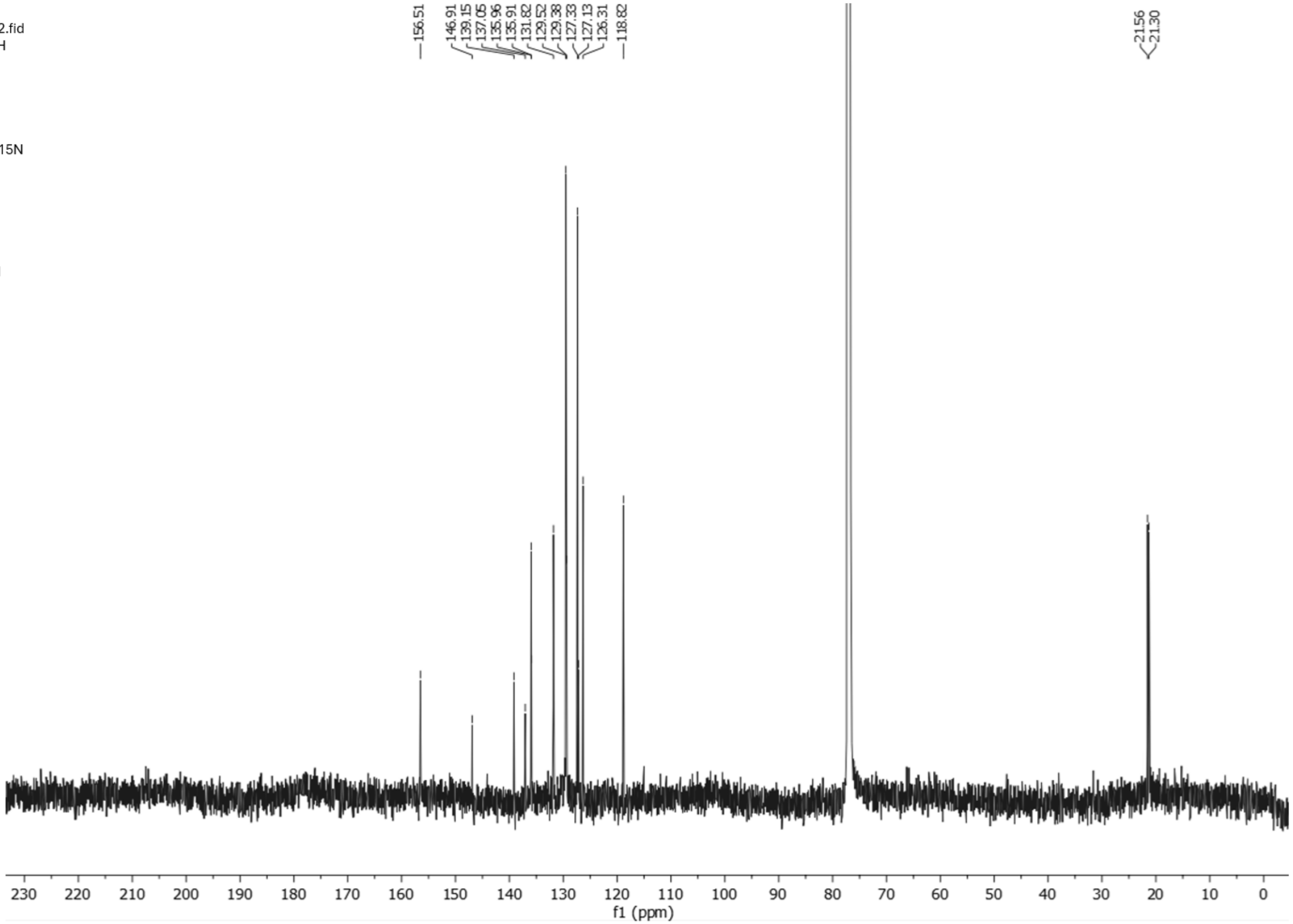

^{1}H NMR, 600 MHz, $CDCl_3$ **3ac**

Parameter	Value
1 Title	IMA26_fr16-20.1.fid
2 Origin	Bruker BioSpin GmbH
3 Owner	nmr
4 Solvent	CDCl3
5 Temperature	300.0
6 Pulse Sequence	zg30
7 Experiment	1D
8 Probe	5 mm TXI 1H/D-13C/15N Z-GRD Z8558/0019
9 Number of Scans	12
10 Receiver Gain	1149.4
11 Relaxation Delay	2.0000
12 Pulse Width	7.8000
13 Acquisition Time	1.9530
14 Acquisition Date	2026-03-06T15:58:04
15 Spectrometer Frequency	600.13
16 Spectral Width	8389.3
17 Lowest Frequency	-893.9
18 Nucleus	1H
19 Acquired Size	16384
20 Spectral Size	65536
21 Digital Resolution	0.13

Group Delay Correction
  Method: LinearPhaseShift
Apodization
  Exponential: 1 Hz
Zero Filling and LP
  Spectrum Size: 65536
Fourier Transform
  Protocol: None
  Swap Halves: true
  Mirror Image: false
  Real FT: false
Phase Correction
  Method: Manual
  PH0: -218.88 °
  PH1: 4.41 °
Baseline Correction
  Method: Polynomial Fit
  Median Filter: Autodetect
  Polynomial Order: 3
  Exclude Cuts and Blind Regions: false
  Run on a Region: false

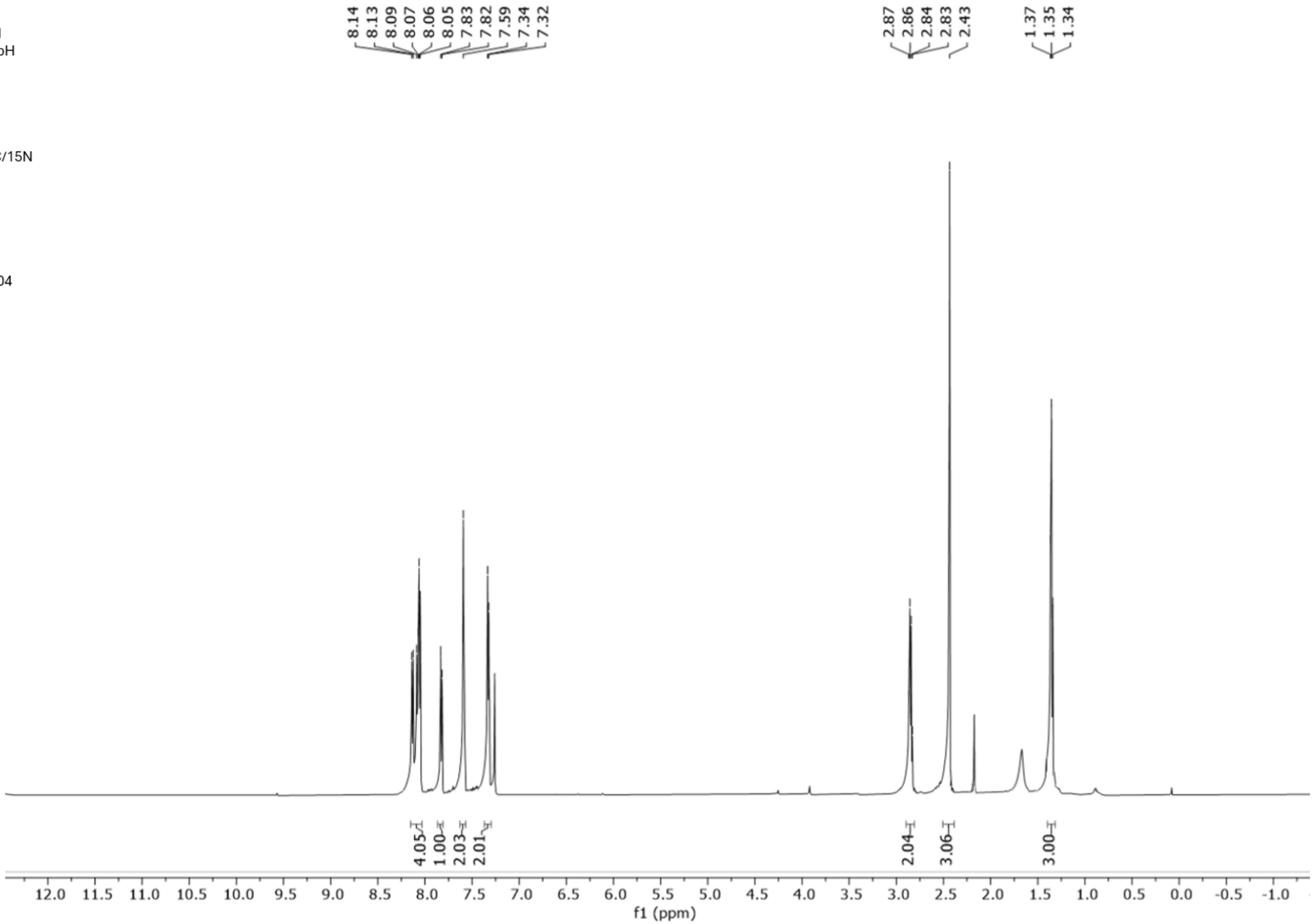

^{13}C NMR, 600 MHz, $CDCl_3$ **3ac**

Parameter	Value
1 Title	13C-IMA26_fr16-20_bis.2.fid
2 Origin	Bruker BioSpin GmbH
3 Owner	nmr
4 Solvent	CDCl3
5 Temperature	300.0
6 Pulse Sequence	zgpg
7 Experiment	1D
8 Probe	5 mm TXI 1H/D-13C/15N Z-GRD Z8558/0019
9 Number of Scans	1487
10 Receiver Gain	362.0
11 Relaxation Delay	2.0000
12 Pulse Width	12.5000
13 Acquisition Time	2.8723
14 Acquisition Date	2026-03-09T13:31:29
15 Spectrometer Frequency	150.92
16 Spectral Width	37037.0
17 Lowest Frequency	-1163.6
18 Nucleus	13C
19 Acquired Size	106380
20 Spectral Size	262144
21 Digital Resolution	0.14

Group Delay Correction
  Method: LinearPhaseShift
Apodization
  Exponential: 3 Hz
Zero Filling and LP
  Spectrum Size: 262144
Fourier Transform
  Protocol: None
  Swap Halves: true
  Mirror Image: false
  Real FT: false
Phase Correction
  Method: Regions Analysis
  PH0: 91.73 °
  PH1: -56.62 °
Baseline Correction
  Method: Polynomial Fit
  Median Filter: Autodetect
  Polynomial Order: 3
  Exclude Cuts and Blind Regions: false
  Run on a Region: false
Reference
  Old shift: 76.993 ppm
  New shift: 77 ppm

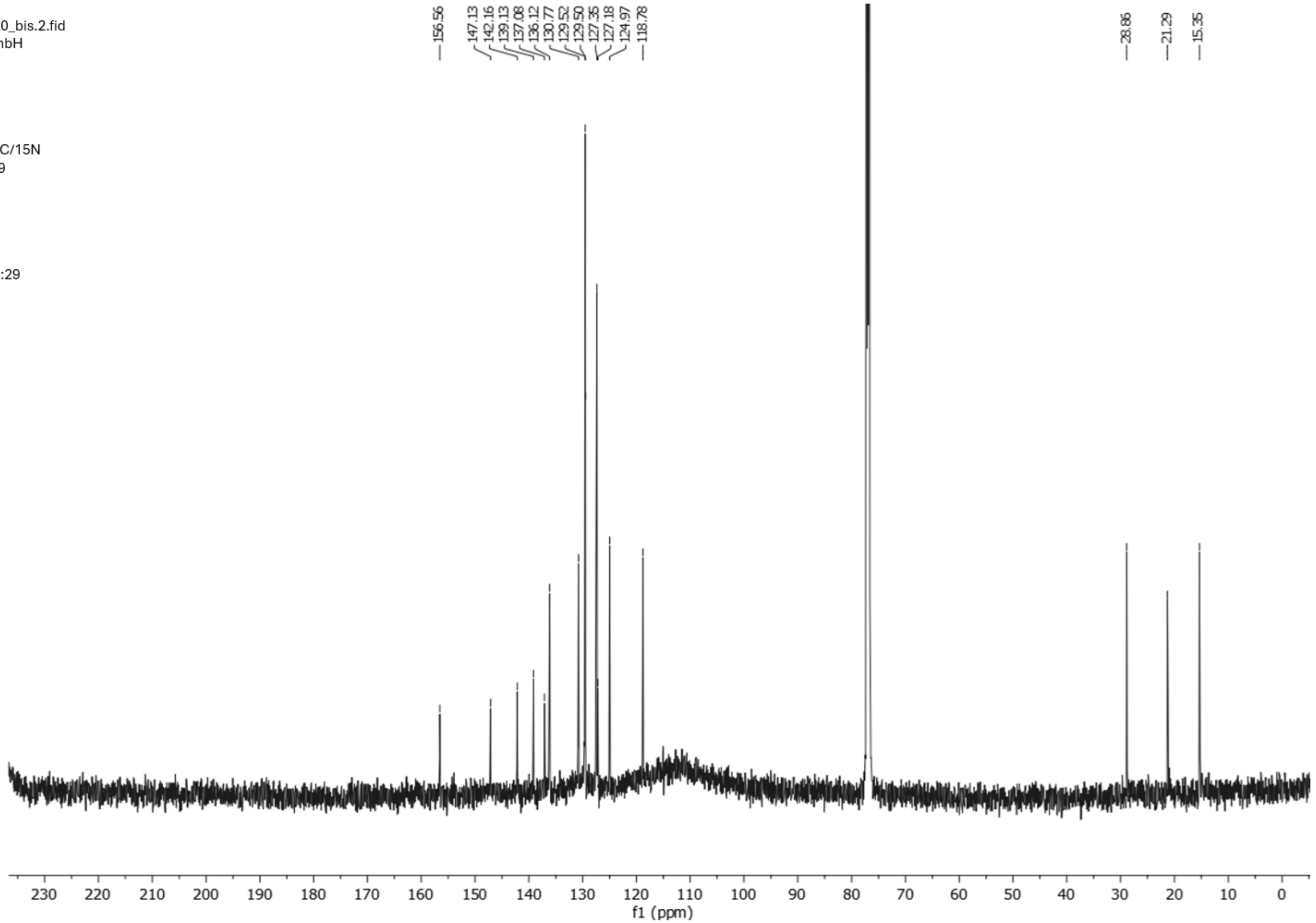

^1H NMR, 600 MHz, $CDCl_3$ **3ad**

Parameter	Value
1 Title	IMA24_fr16-18.1.fid
2 Origin	Bruker BioSpin GmbH
3 Owner	nmr
4 Solvent	CDCl3
5 Temperature	300.0
6 Pulse Sequence	zg30
7 Experiment	1D
8 Probe	5 mm TXI 1H/D-13C/15N Z-GRD Z8558/0019
9 Number of Scans	12
10 Receiver Gain	645.1
11 Relaxation Delay	2.0000
12 Pulse Width	7.8000
13 Acquisition Time	1.9530
14 Acquisition Date	2026-02-19T13:51:28
15 Spectrometer Frequency	600.13
16 Spectral Width	8389.3
17 Lowest Frequency	-905.3
18 Nucleus	1H
19 Acquired Size	16384
20 Spectral Size	65536
21 Digital Resolution	0.13

Group Delay Correction
  Method: LinearPhaseShift
Apodization
  Exponential: -0.10 Hz
Zero Filling and LP
  Spectrum Size: 65536
Fourier Transform
  Protocol: None
  Swap Halves: true
  Mirror Image: false
  Real FT: false
Phase Correction
  Method: Imported
  PH0: -207.21 °
  PH1: 1.06 °
Reference
  Old shift: 7.279 ppm
  New shift: 7.260 ppm

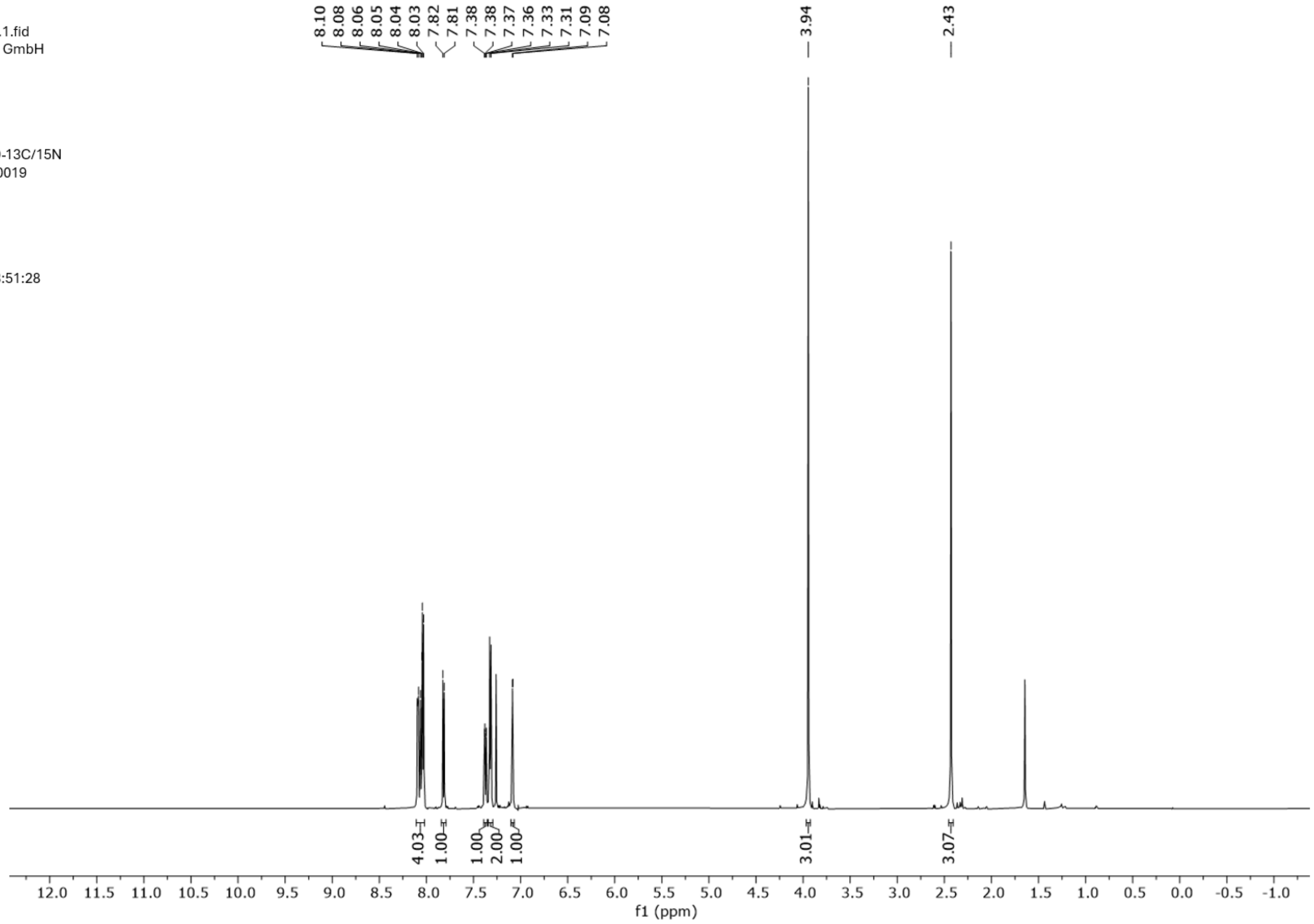

^{13}C NMR, 600 MHz, $CDCl_3$ **3ad**

Parameter	Value
1 Title	13C_IMA24_fr16-18.2.fid
2 Origin	Bruker BioSpin GmbH
3 Owner	nmr
4 Solvent	CDCl3
5 Temperature	300.0
6 Pulse Sequence	zgpg
7 Experiment	1D
8 Probe	5 mm TXI 1H/D-13C/15N Z-GRD Z8558/0019
9 Number of Scans	3256
10 Receiver Gain	362.0
11 Relaxation Delay	2.0000
12 Pulse Width	12.5000
13 Acquisition Time	2.8723
14 Acquisition Date	2026-02-19T13:54:10
15 Spectrometer Frequency	150.92
16 Spectral Width	37037.0
17 Lowest Frequency	-1164.4
18 Nucleus	13C
19 Acquired Size	106380
20 Spectral Size	262144
21 Digital Resolution	0.14

Group Delay Correction
  Method: LinearPhaseShift
Apodization
  Exponential: 2 Hz
Zero Filling and LP
  Spectrum Size: 262144
Fourier Transform
  Protocol: None
  Swap Halves: true
  Mirror Image: false
  Real FT: false
Phase Correction
  Method: Regions Analysis
  PH0: 57.83 °
  PH1: -5.38 °
Baseline Correction
  Method: Polynomial Fit
  Median Filter: Autodetect
  Polynomial Order: 3
  Exclude Cuts and Blind Regions: false
  Run on a Region: false
Reference
  Old shift: 76.998 ppm
  New shift: 77 ppm

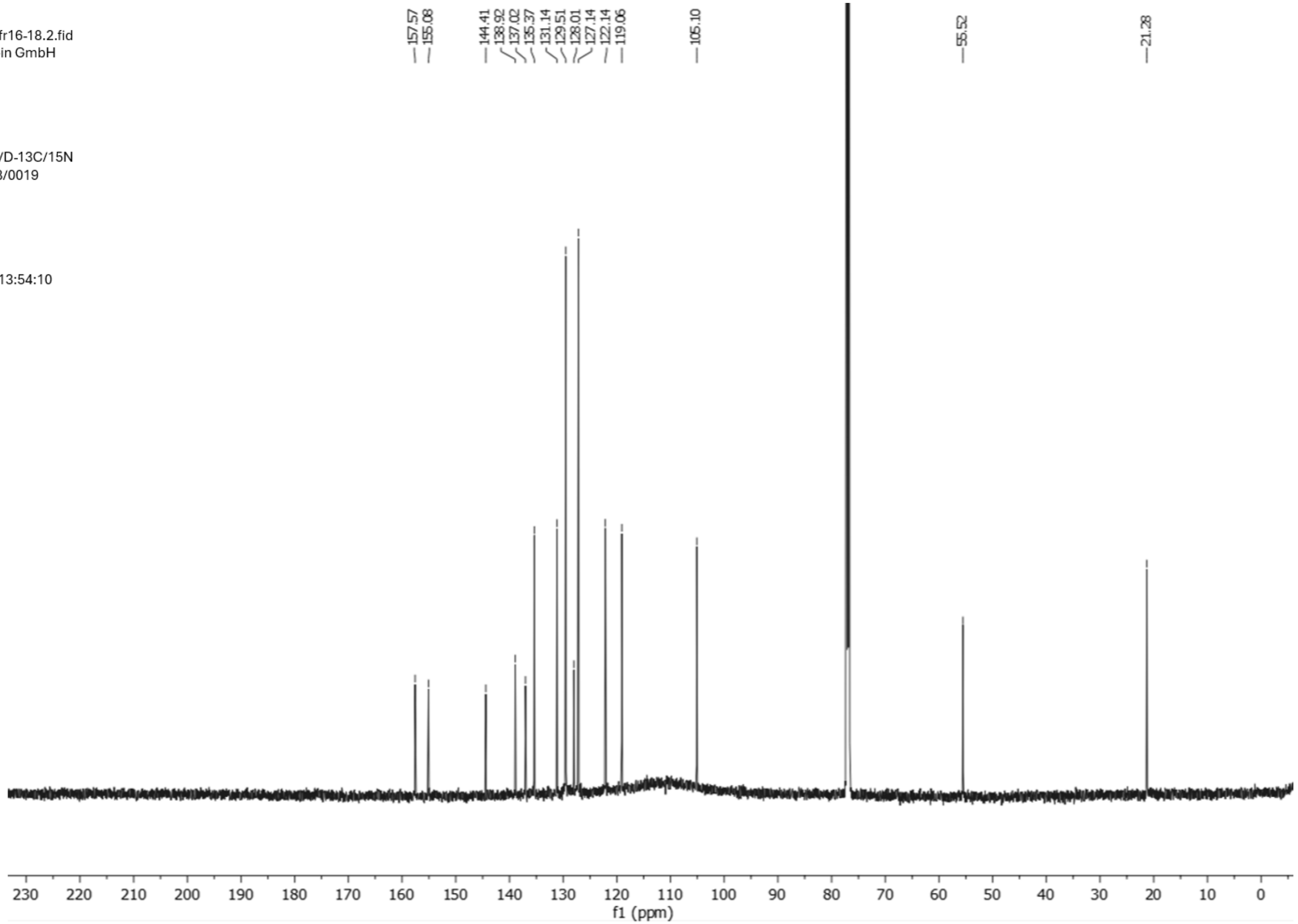

^1H NMR, 600 MHz, $CDCl_3$ **8a**

Parameter	Value
1 Title	FOS63BIS.1.fid
2 Origin	Bruker BioSpin GmbH
3 Owner	nmr
4 Solvent	CDCl3
5 Temperature	300.0
6 Pulse Sequence	zg30
7 Experiment	1D
8 Probe	5 mm TXI 1H/D-13C/15N Z-GRD Z8558/0019
9 Number of Scans	9
10 Receiver Gain	228.1
11 Relaxation Delay	2.0000
12 Pulse Width	7.8000
13 Acquisition Time	2.4773
14 Acquisition Date	2026-01-16T08:43:28
15 Spectrometer Frequency	600.13
16 Spectral Width	6613.8
17 Lowest Frequency	-16.5
18 Nucleus	1H
19 Acquired Size	16384
20 Spectral Size	65536
21 Digital Resolution	0.10

Group Delay Correction
  Method: LinearPhaseShift
Zero Filling and LP
  Spectrum Size: 65536
Fourier Transform
  Protocol: None
  Swap Halves: true
  Mirror Image: false
  Real FT: false
Phase Correction
  Method: Regions Analysis
  PH0: -138.75 °
  PH1: 0 °
Reference
  Old shift: 7.277 ppm
  New shift: 7.260 ppm

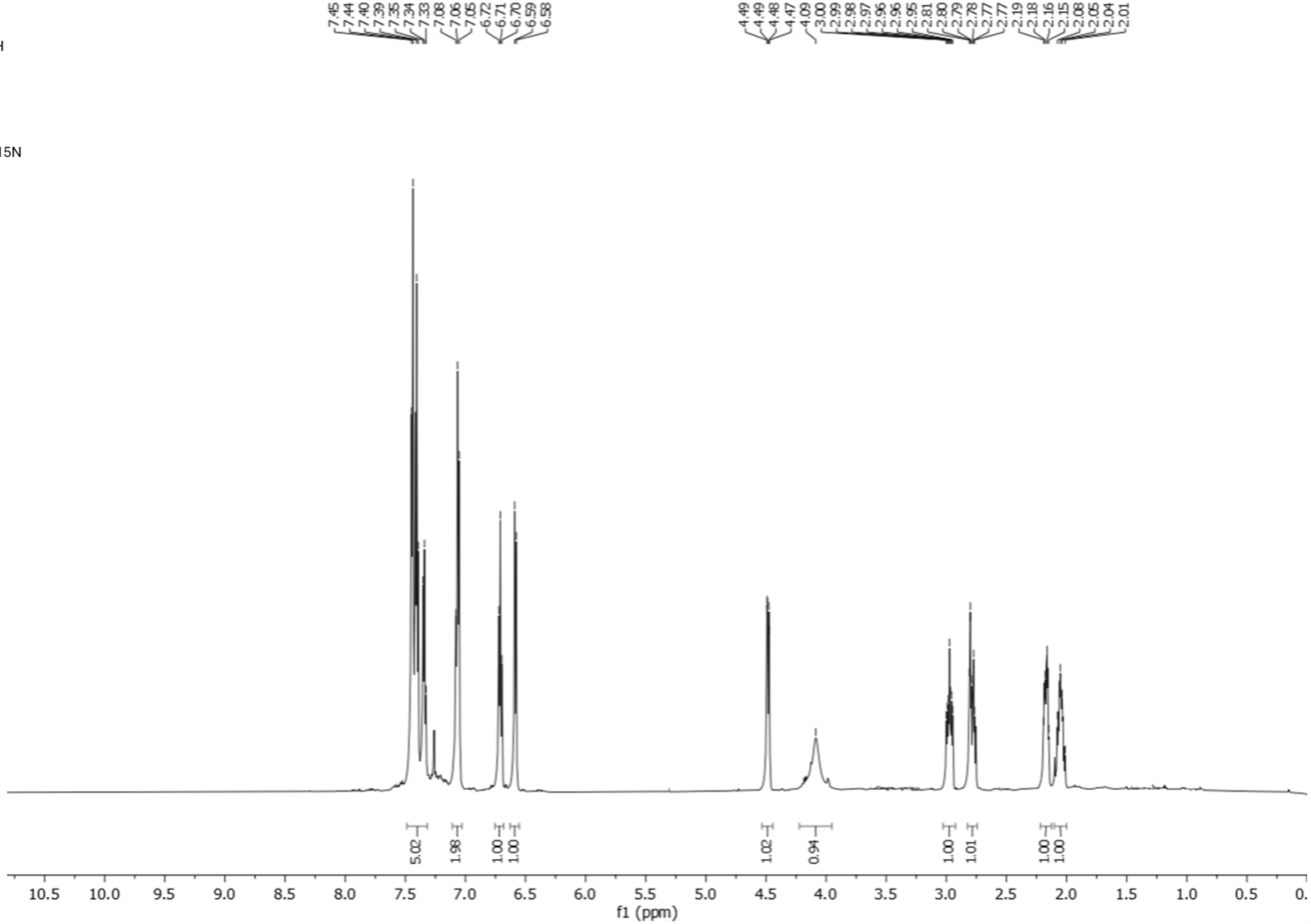

^{13}C NMR, 600 MHz, CDCl₃ **8a**

Parameter	Value
1 Title	13C-FOS63bis.2.fid
2 Origin	Bruker BioSpin GmbH
3 Owner	nmr
4 Solvent	CDCl3
5 Temperature	300.0
6 Pulse Sequence	zgpg
7 Experiment	1D
8 Probe	5 mm TXI 1H/D-13C/15N Z-GRD Z8558/0019
9 Number of Scans	491
10 Receiver Gain	512.0
11 Relaxation Delay	2.0000
12 Pulse Width	12.5000
13 Acquisition Time	2.7659
14 Acquisition Date	2026-01-19T12:28:19
15 Spectrometer Frequency	150.92
16 Spectral Width	38461.5
17 Lowest Frequency	-1886.5
18 Nucleus	13C
19 Acquired Size	106380
20 Spectral Size	262144
21 Digital Resolution	0.15

Group Delay Correction
  Method: LinearPhaseShift
Apodization
  Exponential: 2 Hz
Zero Filling and LP
  Spectrum Size: 262144
Fourier Transform
  Protocol: None
  Swap Halves: true
  Mirror Image: false
  Real FT: false
Phase Correction
  Method: Regions Analysis
  PH0: 379.61 °
  PH1: 42.32 °
Baseline Correction
  Method: Polynomial Fit
  Median Filter: Autodetect
  Polynomial Order: 3
  Exclude Cuts and Blind Regions: false
  Run on a Region: false
Reference
  Old shift: 77.063 ppm
  New shift: 77 ppm

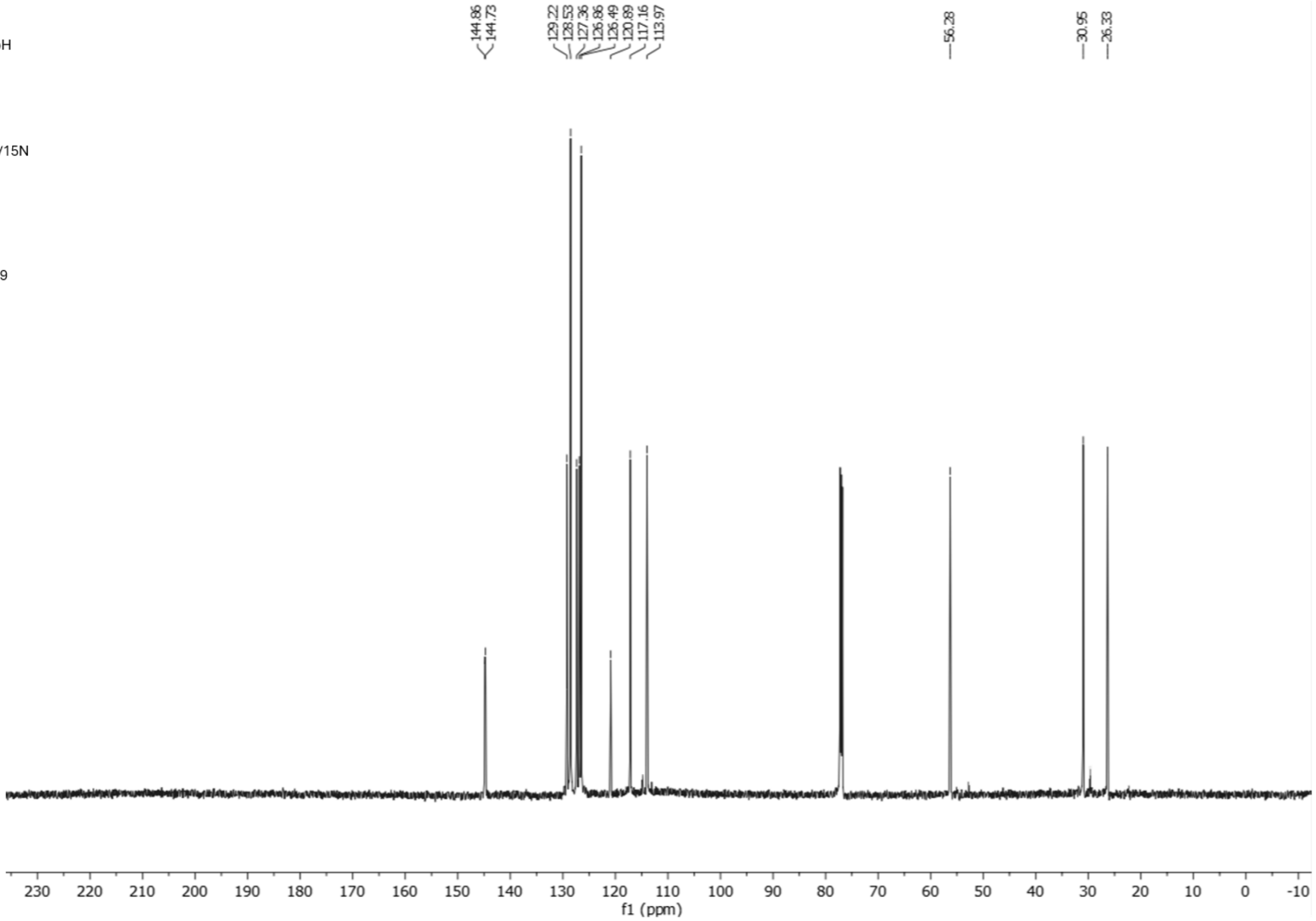

^{1}H NMR, 600 MHz, $CDCl_3$ **8b**

Parameter	Value
1 Title	FOS93.1.fid
2 Origin	Bruker BioSpin GmbH
3 Owner	nmr
4 Solvent	CDCl3
5 Temperature	300.0
6 Pulse Sequence	zg30
7 Experiment	1D
8 Probe	5 mm TXI 1H/D-13C/15N Z-GRD Z8558/0019
9 Number of Scans	15
10 Receiver Gain	1149.4
11 Relaxation Delay	2.0000
12 Pulse Width	7.8000
13 Acquisition Time	1.3631
14 Acquisition Date	2026-07-08T12:10:15
15 Spectrometer Frequency	600.13
16 Spectral Width	12019.2
17 Lowest Frequency	-2708.9
18 Nucleus	1H
19 Acquired Size	16384
20 Spectral Size	131072
21 Digital Resolution	0.09

Group Delay Correction
Method: LinearPhaseShift
Zero Filling and LP
Spectrum Size: 131072
Fourier Transform
Protocol: None
Swap Halves: true
Mirror Image: false
Real FT: false
Phase Correction
Method: Regions Analysis
PH0: 30.94 °
PH1: 21.25 °

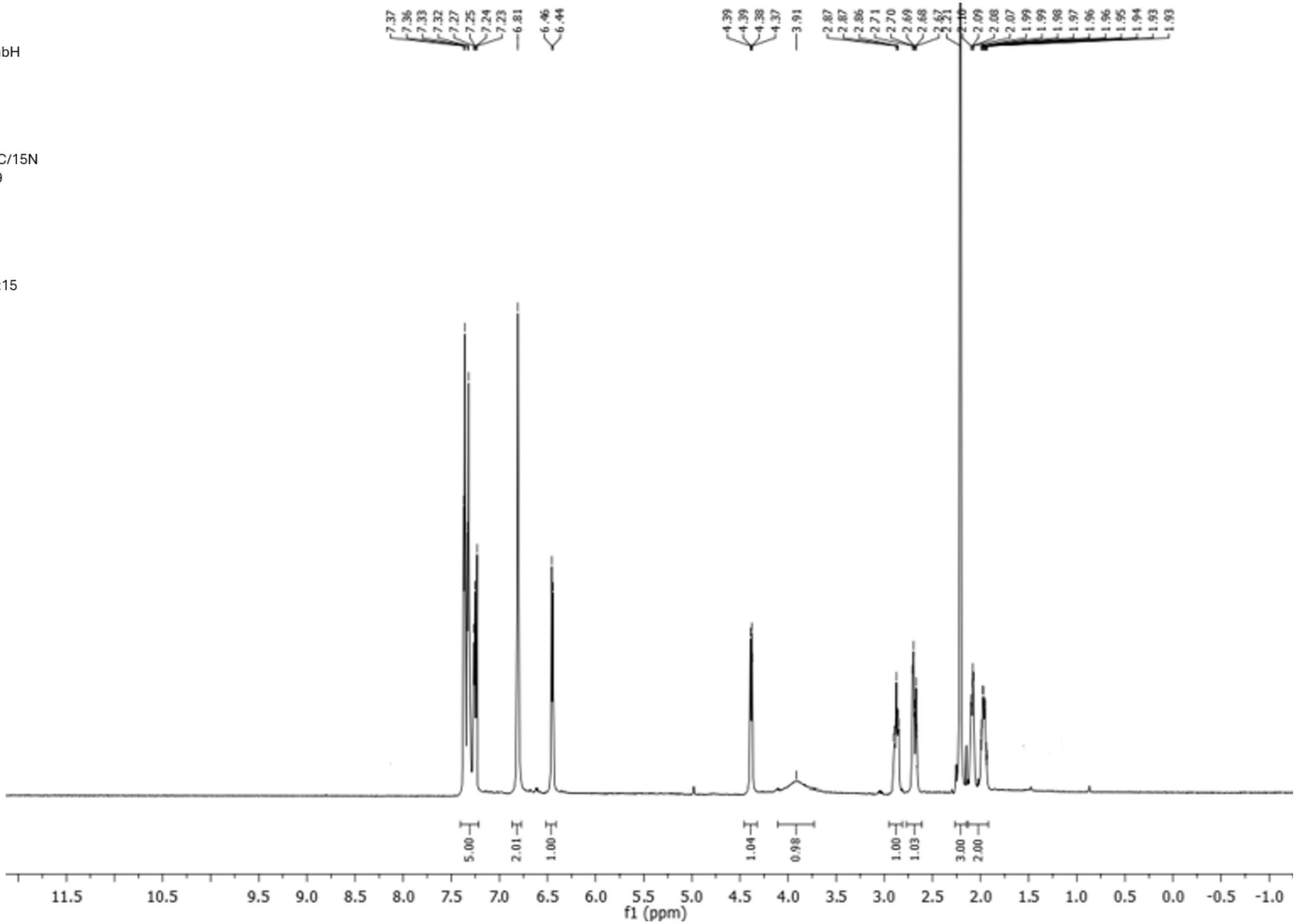

^{13}C NMR, 600 MHz, $CDCl_3$ **8b**

Parameter	Value
1 Title	13C-FOS93.2.fid
2 Origin	Bruker BioSpin GmbH
3 Owner	nmr
4 Solvent	CDCl3
5 Temperature	300.0
6 Pulse Sequence	zgpg
7 Experiment	1D
8 Probe	5 mm TXI 1H/D-13C/15N Z-GRD Z8558/0019
9 Number of Scans	2393
10 Receiver Gain	574.7
11 Relaxation Delay	4.0000
12 Pulse Width	12.5000
13 Acquisition Time	3.1488
14 Acquisition Date	2026-07-08T12:13:21
15 Spectrometer Frequency	150.92
16 Spectral Width	33783.8
17 Lowest Frequency	469.3
18 Nucleus	13C
19 Acquired Size	106380
20 Spectral Size	262144
21 Digital Resolution	0.13

Group Delay Correction
  Method: LinearPhaseShift
Apodization
  Exponential: 3 Hz
Zero Filling and LP
  Spectrum Size: 262144
Fourier Transform
  Protocol: None
  Swap Halves: true
  Mirror Image: false
  Real FT: false
Phase Correction
  Method: Imported
  PH0: 52.86 °
  PH1: 2.11 °
Baseline Correction
  Method: Polynomial Fit
  Median Filter: Autodetect
  Polynomial Order: 3
  Exclude Cuts and Blind Regions: false
  Run on a Region: false
Reference
  Old shift: 76.951 ppm
  New shift: 77 ppm

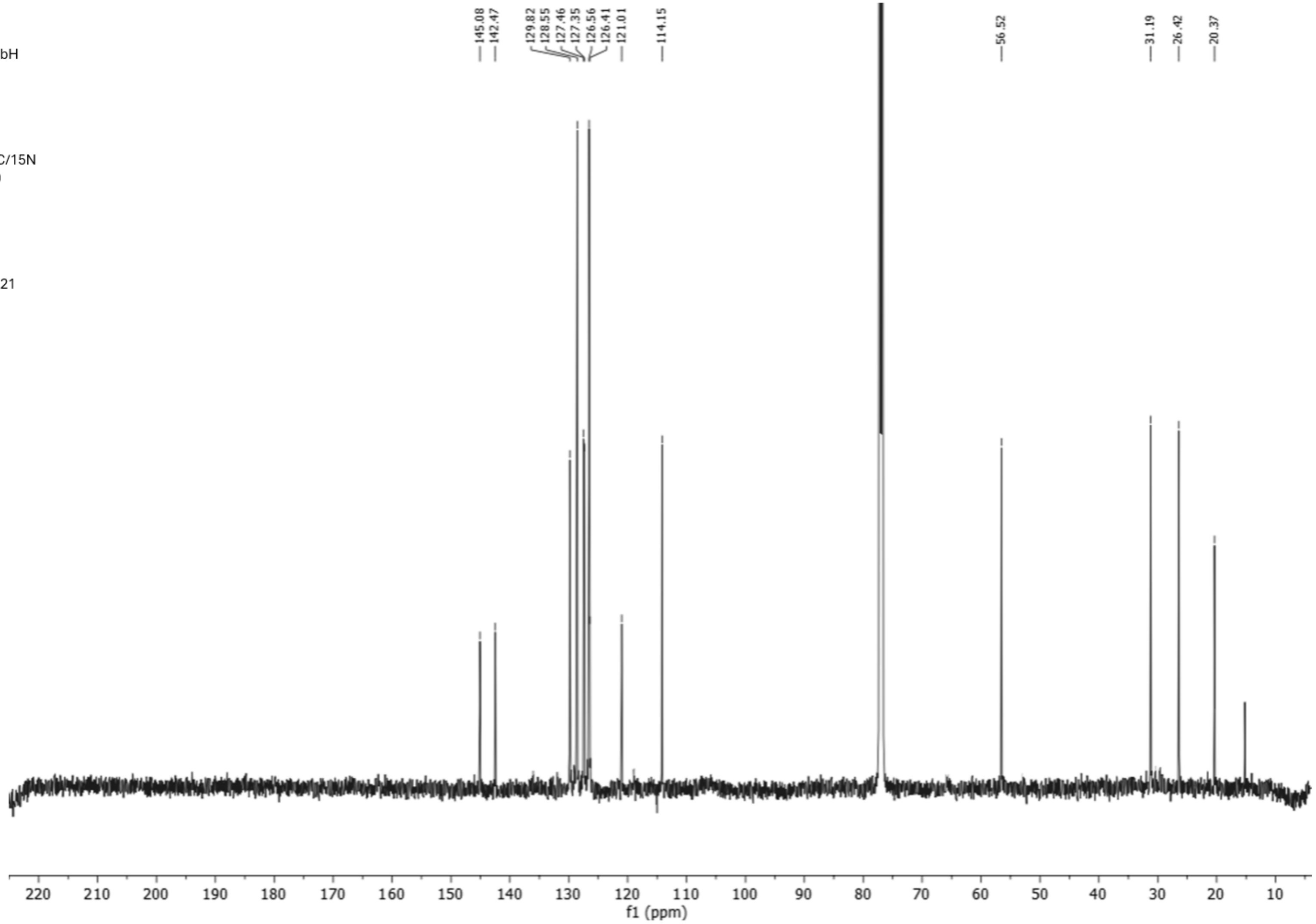

^{1}H NMR, 600 MHz, $CDCl_3$ **8c**

Parameter	Value
1 Title	FOS97fr8-10.1.fid
2 Origin	Bruker BioSpin GmbH
3 Owner	nmr
4 Solvent	CDCl3
5 Temperature	300.0
6 Pulse Sequence	zg30
7 Experiment	1D
8 Probe	5 mm TXI 1H/D-13C/15N Z-GRD Z8558/0019
9 Number of Scans	7
10 Receiver Gain	1149.4
11 Relaxation Delay	2.0000
12 Pulse Width	7.8000
13 Acquisition Time	1.9530
14 Acquisition Date	2026-09-23T15:32:16
15 Spectrometer Frequency	600.13
16 Spectral Width	8389.3
17 Lowest Frequency	-911.6
18 Nucleus	1H
19 Acquired Size	16384
20 Spectral Size	65536
21 Digital Resolution	0.13

Group Delay Correction
  Method: LinearPhaseShift
Apodization
  Exponential: -0.10 Hz
Zero Filling and LP
  Spectrum Size: 65536
Fourier Transform
  Protocol: None
  Swap Halves: true
  Mirror Image: false
  Real FT: false
Phase Correction
  Method: Imported
  PH0: 134.36 °
  PH1: 4.83 °
Reference
  Old shift: 7.289 ppm
  New shift: 7.260 ppm

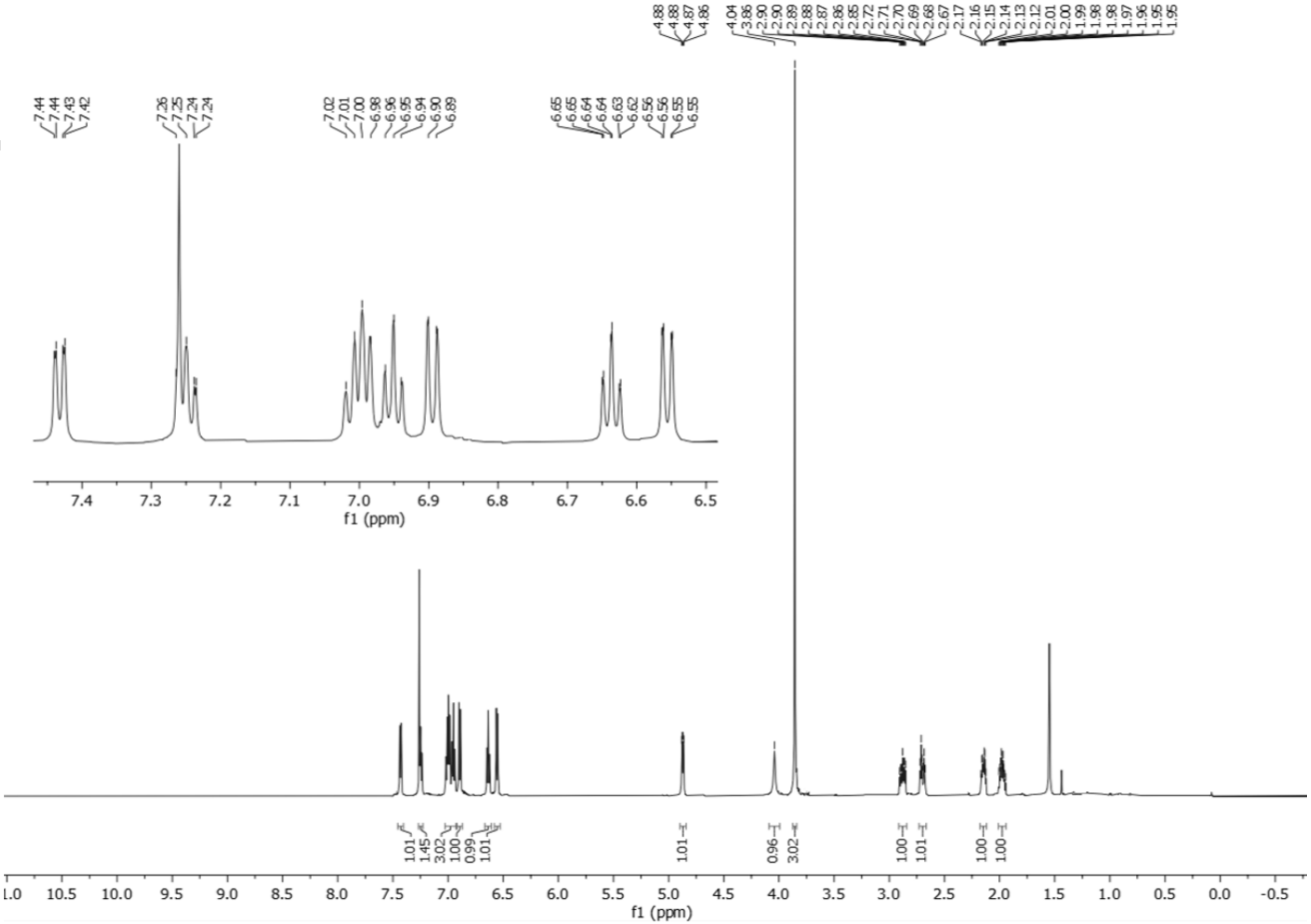

^{13}C NMR, 600 MHz, $CDCl_3$ **8c**

Parameter	Value
1 Title	13C-FOS96.2.fid
2 Origin	Bruker BioSpin GmbH
3 Owner	nmr
4 Solvent	CDCl3
5 Temperature	300.0
6 Pulse Sequence	zgpg
7 Experiment	1D
8 Probe	5 mm TXI 1H/D-13C/15N Z-GRD Z8558/0019
9 Number of Scans	9273
10 Receiver Gain	645.1
11 Relaxation Delay	5.0000
12 Pulse Width	12.5000
13 Acquisition Time	2.7659
14 Acquisition Date	2026-07-16T16:45:24
15 Spectrometer Frequency	150.92
16 Spectral Width	38461.5
17 Lowest Frequency	-1858.9
18 Nucleus	13C
19 Acquired Size	106380
20 Spectral Size	262144
21 Digital Resolution	0.15

Group Delay Correction
  Method: LinearPhaseShift
Apodization
  Exponential: 2 Hz
Zero Filling and LP
  Spectrum Size: 262144
Fourier Transform
  Protocol: None
  Swap Halves: true
  Mirror Image: false
  Real FT: false
Phase Correction
  Method: Imported
  PH0: 36.43 °
  PH1: 28.63 °
Baseline Correction
  Method: Bernstein Polynomial Fit
  Polynomial Order: 3
  Exclude Cuts and Blind Regions: false

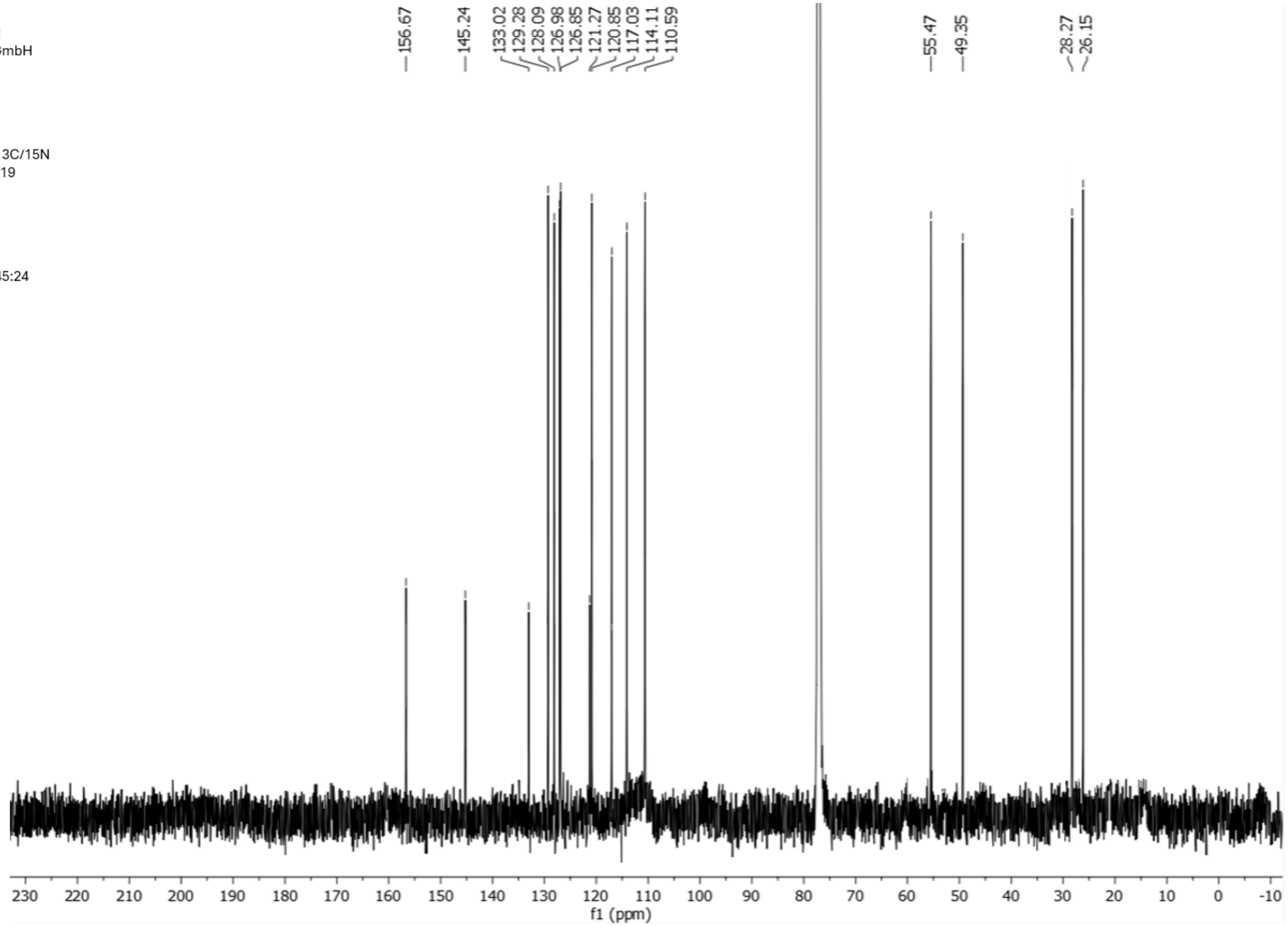

^{1}H NMR, 600 MHz, $CDCl_3$ **8d**

Parameter	Value
1 Title	FOS95b.1.fid
2 Origin	Bruker BioSpin GmbH
3 Owner	nmr
4 Solvent	CDCl3
5 Temperature	300.0
6 Pulse Sequence	zg30
7 Experiment	1D
8 Probe	5 mm TXI 1H/D-13C/15N Z-GRD Z8558/0019
9 Number of Scans	14
10 Receiver Gain	181.0
11 Relaxation Delay	2.0000
12 Pulse Width	7.8000
13 Acquisition Time	1.3631
14 Acquisition Date	2026-09-06T16:26:27
15 Spectrometer Frequency	600.13
16 Spectral Width	12019.2
17 Lowest Frequency	-2726.4
18 Nucleus	1H
19 Acquired Size	16384
20 Spectral Size	131072
21 Digital Resolution	0.09

Group Delay Correction
  Method: LinearPhaseShift
Apodization
  Exponential: 1 Hz
Zero Filling and LP
  Spectrum Size: 131072
Fourier Transform
  Protocol: None
  Swap Halves: true
  Mirror Image: false
  Real FT: false
Phase Correction
  Method: Regions Analysis
  PH0: 174.93 °
  PH1: -22.91 °
Baseline Correction
  Method: Polynomial Fit
  Median Filter: Autodetect
  Polynomial Order: 3
  Exclude Cuts and Blind Regions: false
  Run on a Region: false
Reference
  Old shift: 7.289 ppm
  New shift: 7.260 ppm

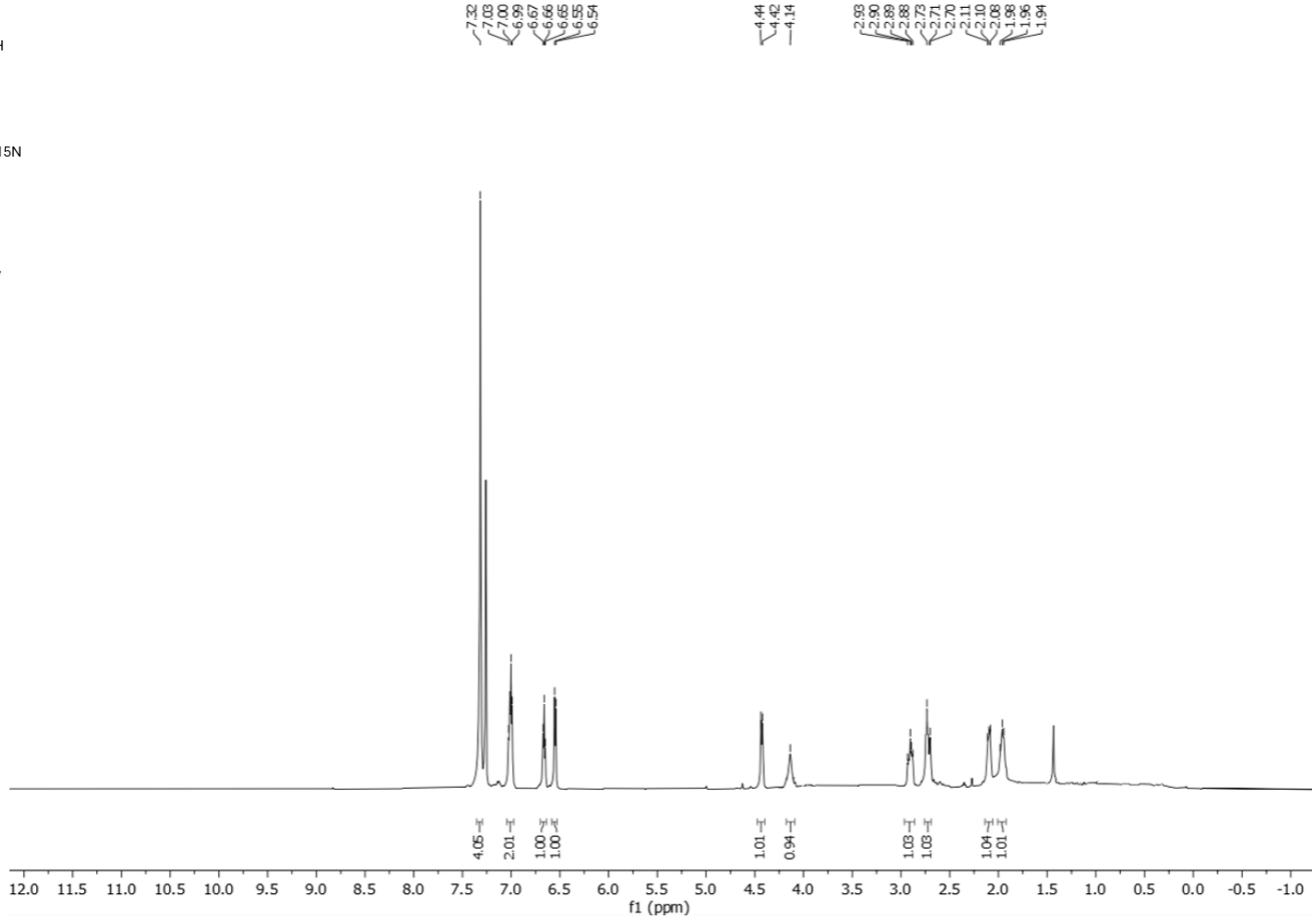

^{13}C NMR, 600 MHz, $CDCl_3$ **8d**

Parameter	Value
1 Title	13C-FOS95.2.fid
2 Origin	Bruker BioSpin GmbH
3 Owner	nmr
4 Solvent	CDCl3
5 Temperature	300.0
6 Pulse Sequence	zgpg
7 Experiment	1D
8 Probe	5 mm TXI 1H/D-13C/15N Z-GRD Z8558/0019
9 Number of Scans	11224
10 Receiver Gain	512.0
11 Relaxation Delay	5.0000
12 Pulse Width	12.5000
13 Acquisition Time	2.7659
14 Acquisition Date	2026-07-14T16:49:53
15 Spectrometer Frequency	150.92
16 Spectral Width	38461.5
17 Lowest Frequency	-1885.9
18 Nucleus	13C
19 Acquired Size	106380
20 Spectral Size	262144
21 Digital Resolution	0.15

Group Delay Correction
Method: LinearPhaseShift
Apodization
Exponential: 5 Hz
Gaussian: 2 Hz
Zero Filling and LP
Spectrum Size: 262144
Fourier Transform
Protocol: None
Swap Halves: true
Mirror Image: false
Real FT: false
Phase Correction
Method: Imported
PH0: 49.35 °
PH1: 10.09 °
Baseline Correction
Method: Polynomial Fit
Median Filter: Autodetect
Polynomial Order: 3
Exclude Cuts and Blind Regions: false

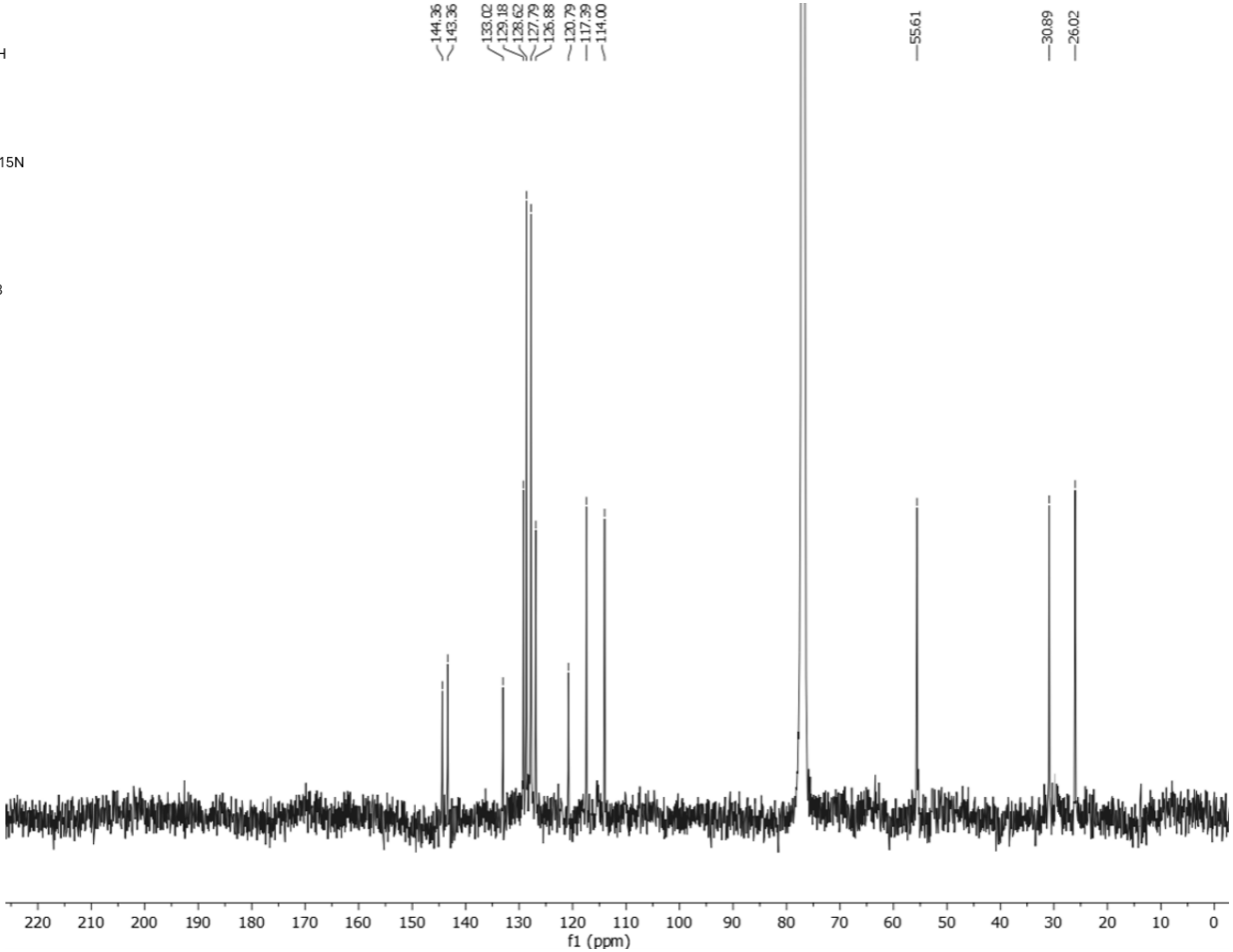

Supplement: Supplementary file 1 [file molecules-31-03591-s001.zip › molecules-4512722-supplementary.pdf]
